# Optimization of extracellular vesicle extraction from hepatic tissue interstitial fluid and analysis of their ncRNA expression profiles

**DOI:** 10.1371/journal.pone.0355303

**Published:** 2026-08-03

**Authors:** Shubo Liu, Yuxuan Fu, Hua Guo, Hailin Li, Lei Zhang, Shuangshuang Du, Chunyue Guo, Cheng Lou, Jinjuan Zhang, Chengjun Lu, Yingtang Gao

**Affiliations:** 1 The Third Central Clinical College of Tianjin Medical University, Tianjin, China; 2 Tianjin Key Laboratory of Extracorporeal Life Support for Critical Diseases, Tianjin Institute of Hepatobiliary Disease, The Third Central Hospital of Tianjin, Tianjin, China; 3 School of Medicine, Nankai University, Tianjin, China; 4 Department of Hepatobiliary Surgery, Nankai University Affiliated Third Center Hospital, Tianjin, China; CRCL: Centre de Recherche en Cancerologie de Lyon, FRANCE

## Abstract

**Background:**

To address the limited tissue specificity of extracellular vesicles (EVs) derived from blood and other body fluids, this study isolated small EVs (sEVs) from the tissue interstitial fluid (TIF) of hepatocellular carcinoma (HCC) and adjacent tissues. The expression profiles of non-coding RNAs (ncRNAs) were analyzed to identify more specific diagnostic biomarkers.

**Methods:**

An optimized protocol for TIF-sEV extraction was established, which combined enzymatic digestion (Collagenase D and DNase I) with differential and ultracentrifugation. The isolated sEVs were characterized using nanoparticle tracking analysis (NTA), transmission electron microscopy (TEM), and western blotting (WB). The expression of 33 candidate ncRNAs (11 lncRNAs from TCGA-LIHC and 22 miRNAs from TCGA-LIHC and GSE302990) in TIF-sEVs was analyzed by qRT-PCR and integrated with clinical parameters via LASSO regression.

**Results:**

The optimal extraction conditions were determined to be digestion with Collagenase D (2 mg/mL) and DNase I (40 U/mL) for 30 minutes. The obtained EVs exhibited typical morphology, a particle size below 200 nm, and expressed canonical EV marker proteins (ALIX, CD63) as well as the hepatocyte-specific membrane protein ASGPR. qRT-PCR analysis revealed specific enrichment of six lncRNAs (e.g., AL031985, TMCC1-AS1) and eleven miRNAs (e.g., miR-1224-5p, miR-483-5p) in TIF-sEVs. LASSO regression applied to all 33 candidate ncRNAs and clinical parameters identified a combined diagnostic signature comprising ALB, PLT, DBIL, lncRNA GAS5, and miR-194-5p, which achieved an area under the curve (AUC) of 0.960.

**Conclusions:**

This study establishes an efficient and stable system for the isolation and characterization of TIF-sEVs from liver tissue. Furthermore, it identifies multiple non-coding RNAs (ncRNAs) that are enriched in TIF-sEVs, thereby providing potential novel diagnostic biomarkers for the differential diagnosis and prognosis evaluation of HCC.

## 1 Introduction

Extracellular vesicles (EVs), a class of phospholipid bilayer vesicles actively secreted by cells, have emerged as highly promising sources of disease biomarkers due to their stable presence in various body fluids and their capacity to carry bioactive molecules, such as proteins and nucleic acids, which reflect the state of parental cells [1–4]. The diagnostic value of biofluid-derived EVs (Bf-EVs) has been evaluated across various diseases [5–8]. For instance, in HCC, the level of LC3B-positive EVs in patient plasma is significantly elevated [7], and specific miRNAs, such as miR-122-5p, within serum EVs have demonstrated diagnostic potential [8].

However, the application of Bf-EVs faces several challenges. Due to their diverse sources and lack of tissue specificity, accurately tracing their organ origins remains difficult, which limits the specificity and sensitivity of related biomarkers [3,9–11]. To address this issue, research focus has gradually shifted toward the tumor microenvironment closer to the primary lesion, specifically the tissue interstitial fluid (TIF). TIF is the fluid present in the interstitium of tissues and serves as the main component of extracellular fluid, forming the immediate internal environment for cell survival. It contains abundant EVs secreted by local cells, which may retain the specific molecular fingerprints of their originating tissues at an earlier stage, thereby providing direct evidence for the tissue tracing of Bf-EVs [12–15]. This strategy is emerging as an ideal bridge connecting the precision of ‘tissue biopsy’ with the convenience of ‘liquid biopsy,’ demonstrating significant potential across multiple disease areas. Examples include the identification of miR-483-5p as a diagnostic biomarker through high-throughput sequencing of HCC tissue interstitial fluid and subsequent validation [16], proteins derived from skin interstitial fluid EVs have been utilized for point-of-care detection of melanoma [17], the use of dermal interstitial fluid EV miRNAs as markers for burn depth assessment [18], SERS spectral features of EVs in bronchoalveolar lavage fluid have been applied to diagnose non-small cell lung cancer [19]; miRNAs and proteins in peritoneal fluid-derived EVs have been employed for ovarian cancer diagnosis [20,21]; and proteomics of of cerebrospinal fluid EVs have been used in the differential diagnosis of neurodegenerative diseases [22,23], multiple sclerosis [24], and medulloblastoma [25].

To date, the sampling methods for TIF-EVs from various tissues exhibit significant variability, and no unified standards have been established. For instance, brain tissues are obtained through microdialysis [12,22–25], while skin tissues are collected using microneedles [17]. In contrast, visceral tissues, such as the liver, are acquired through surgical resection or minimally invasive procedures, followed by enzymatic digestion and differential/ultracentrifugation [16,26,27]. Specifically, TIF-EVs from HCC tissues are isolated via enzymatic digestion combined with differential/ultracentrifugation [16,26,27]. Although this method has been characterized through transmission electron microscopy (TEM), nanoparticle tracking analysis (NTA), and Western blotting (WB), confirming its adherence to the fundamental requirements of the MISEV2023 guidelines, the specific operational procedures have yet to be standardized. Furthermore, research into its diagnostic biomarkers remains in the preliminary exploratory stage [16]. Therefore, the present study aims to refine the isolation method for liver tissue-derived TIF-sEVs and to explore the non-coding RNAs they contain, with the goal of providing potential biomarkers for the differential diagnosis and precise intervention of HCC.

## 2 Results

### 2.1 Isolation and characterization of TIF-sEVs

**Fig 1:**
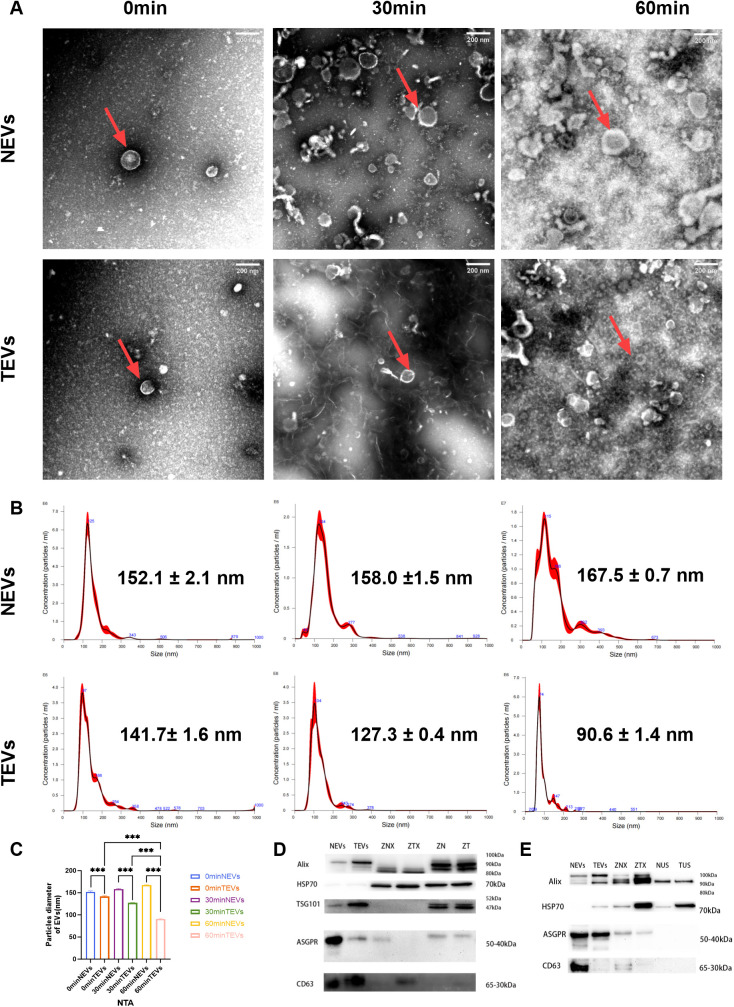
Characterization of TIF-EVs derived from adjacent non-tumor and tumor tissues. (A) TEM images of EVs under different enzyme incubation durations; (B) NTA size distribution of EVs under different enzyme incubation durations; (C) NTA particle diameter analysis of NEVs and TEVs under different enzyme incubation durations; (D) Western Blot results after 30-minute enzyme incubation; (E) Western Blot results after 60-minute enzyme incubation. Legend for Western Blot figures: NEVs: Protein from non-tumor adjacent tissue-derived TIF-sEVs. TEVs: Protein from tumor tissue-derived TIF-sEVs. ZNX: Protein from non-tumor adjacent tissue pellet after EV extraction. ZTX: Protein from tumor tissue pellet after EV extraction. NUS: Protein from supernatant after ultracentrifugation pellet of non-tumor adjacent tissue EVs. TUS: Protein from supernatant after ultracentrifugation pellet of tumor tissue EVs. ZN: Protein from non-tumor adjacent tissue. ZT: Protein from tumor tissue.

TEM results demonstrated that EVs isolated from the tissue interstitial fluid of non-tumor adjacent tissue (NEVs) and tumor tissue (TEVs), analyzed under various digestion times (0 min, 30 min, and 60 min) and centrifugation repetitions (one differential centrifugation step versus repeated differential centrifugation), exhibited the classic ‘cup-shaped’ morphology. The majority of particles within the fields of view were relatively uniform in size, consistent with the characteristics of EVs, thereby confirming the successful extraction of tissue interstitial fluid-derived small extracellular vesicles (TIF-sEVs) (Fig 1A and S1A-S1D Fig). Specifically, the 0-min and 30-min groups displayed morphologically typical EVs with clean backgrounds and minimal impurities, whereas the 60-min group exhibited a cluttered background with numerous protein aggregates, lipoproteins, and other non-vesicular structures alongside EVs. Furthermore, TIF-sEVs subjected to repeated differential centrifugation contained fewer impurities compared to those processed with a single centrifugation step. Finally, to assess the storage stability of TIF-sEVs, this study compared the morphological integrity of samples stored at 4°C versus −80°C for one week. The results indicated that TIF-sEVs stored at −80°C exhibited significantly fewer background contaminants, such as protein aggregates and non-vesicular structures, than those stored at 4°C, suggesting that storage at −80°C is more conducive to maintaining the morphological purity and stability of TIF-sEVs.

NTA analysis revealed that the average particle diameters of both NEVs and TEVs extracted at different digestion times (0 min, 30 min, and 60 min) were consistently below 200 nm, which aligns with the characteristics of sEVs and confirms the successful extraction of TIF-sEVs (Fig 1B-1C). The average particle diameter of NEVs exhibited a non-significant increasing trend with longer digestion times. In contrast, the average particle diameter of TEVs decreased significantly as digestion time increased. Notably, the average particle diameter of NEVs was significantly larger than that of TEVs at all digestion time points. Furthermore, particle concentration progressively increased with digestion time, with statistically significant differences observed between most groups, except for the comparison between the 0-min TEVs group and the 30-min group (Table 1).

**Table 1:** Characterization of TIF-EVs: Total protein concentration and particle properties.

	0 min	30 min	60 min
**Particles concentration of NEVs(particles/ml)**	3.95 × 10^11^ ± 2.03 × 10^8C^	1.86 × 10^12^ ± 6.93 × 10^10B^	2.14 × 10^13^ ± 1.09 × 10^12A^
**Particles concentration of TEVs(particles/ml)**	8.55 × 10^10^ ± 3.57 × 10^8C†^	2.38 × 10^12^ ± 4.91 × 10^10B†^	2.26 × 10^13^ ± 6.44 × 10^11A†^
**Protein concentration of NEVs(mg/ml)**	0.32 ± 0.01^c^	1.52 ± 0.41^b^	2.45 ± 0.34^a^
**Protein concentration of TEVs(mg/ml)**	0.30 ± 0.01^b^	1.09 ± 0.47^b^	1.94 ± 0.52^a^

Caption. Within the same row, different lowercase letter superscripts (a, b, c) indicate significant differences (p < 0.05). Within the same column, the cross symbol (†) signifies a statistically significant difference (determined by independent samples t-test, p < 0.01) between New Energy Vehicles (NEVs) and Traditional Energy Vehicles (TEVs) at that treatment concentration.

BCA assay results indicated that the total protein concentration of TIF-sEVs displayed an increasing trend with prolonged digestion time, with statistically significant differences noted between groups. However, no significant difference was found between NEVs and TEVs (Table 1).

Western Blot results demonstrated that under same loading amount (Fig 1D-1E and S1E Fig), the positive markers CD63, CD9, TSG101, and Alix were all detected in TIF-sEVs. Among these, CD9 exhibited a pronounced signal in the 30-minute TEVs, while a weaker signal was observed in the 60-minute TEVs. TSG101 predominantly appeared as a 47 kDa band at 30 minutes, whereas both 52 kDa and 47 kDa bands were observed at 60 minutes. Alix was primarily present as a 100 kDa subunit in TIF-sEVs, displaying a pattern distinct from the three molecular weight forms (100, 90, and 80 kDa) observed in tissue and the two forms (100 and 90 kDa) observed in cells. The negative marker Calnexin was detectable in NEVs and 60-minute TEVs, albeit with weaker signal intensity than that of the positive markers. The 0-minute control group was essentially negative, confirming that simple mechanical disruption does not release detectable EVs. Regarding source markers, both hepatocyte markers ASGPR and ALB were positive, with ASGPR exhibiting markedly higher signal intensity compared to the cell control. The signals for the biliary epithelial marker CK19 and the vascular endothelial marker CD34 were both weaker than those observed in cells. These results demonstrate that the isolated vesicles conform to the characteristics of EVs and suggest that hepatocytes are one of their principal sources.

Based on the characterization analysis, TIF-sEVs derived from paracancerous tissues and tumor tissues exhibit differences in particle size, concentration, and cargo content. This indicates that TIF-sEVs possess high tissue specificity and demonstrate potential as biomarkers for liver cancer. Additionally, after a comprehensive consideration of vesicle yield and purity, the final protocol for subsequent TIF-sEVs experiments was established to include a 30-minute enzymatic digestion, one round of differential centrifugation, and storage at −80°C.

### 2.2 Expression characteristics of lncRNA molecules

#### 2.2.1 Analysis of lncRNA CT values.

The detection of lncRNAs in TIF-sEVs, tissues, and US was conducted (Table 2). The CT value results indicated that five lncRNAs, namely AL158166, TMCC1-AS1, CERNA2, LINC00622, and AL031985, exhibited CT values that were 2–6 cycles lower in TIF-sEVs compared to tissues, suggesting their enrichment in vesicles. Conversely, the reference gene GAPDH and six lncRNAs (GAS5, H19, LINC00839, SNHG1, LINC03067, and ST8SIA6-AS1) displayed CT values that were 1–7 cycles higher in TIF-sEVs than in tissues. Furthermore, with the exception of cases where LINC03067 was undetected in some NEVs, and the TUS of LINC03067 and NUS of ST8SIA6-AS1 were undetected (rendering CT values incomparable), the CT values of other lncRNAs in TIF-sEVs were at least 4 cycles lower than those in US. This finding suggests the presence of free lncRNAs or other Non-Vesicular Extracellular Particles (NVEPs), such as exomeres or supermeres, in US. It can be speculated that five lncRNA molecules, including AL031985 and AL158166, may be selectively packaged and enriched in TIF-sEVs, categorizing them as TIF-sEVs-enriched ncRNAs.

**Table 2:** Quartile statistics of lncRNA expression CT values across sample groups.

lncRNA	NEVs(22)	TEVs(21)	ZN(24)	ZT(24)	NUS(11)	TUS(9)	NEVs-ZN	TEVs-ZT	NEVs-NUS	TEVs-TUS
**GAPDH**	18.83(17.65,20.70)	19.31(18.13,20.21)	17.92(16.20,18.79)	15.63(14.64,17.09)	23.90(23.11,25.10)	24.58(23.08,27.51)	0.91	3.68	−5.07	−5.27
**AL158166**	24.99(24.33,27.17)	25.57(24.42,26.81)	31.94(31.38,32.70)	31.75(30.86,32.94)	29.96(28.64,31.02)	30.56(29.63,33.48)	−6.95	−6.18	−4.97	−4.99
**TMCC1-AS1**	26.43(25.16,28.16)	26.90(25.48,28.69)	33.20(31.48,34.38)	31.36(29.68,33.21)	31.29(29.84,33.12)	31.47(30.55,35.15)	−6.77	−4.46	−4.86	−4.57
**CERNA2**	23.82(22.23,25.65)	24.32(22.73,25.64)	30.21(29.75,31.10)	30.22(29.02,30.85)	28.16(26.97,30.16)	29.19(27.73,31.81)	−6.39	−5.9	−4.34	−4.87
**LINC00622**	21.34(19.93,24.70)	21.76(20.53,23.80)	25.97(24.64,27.24)	25.43(24.46,26.27)	27.45(25.43,29.09)	27.83(26.12,30.71)	−4.63	−3.67	−6.11	−6.07
**AL031985**	22.23(21.07,23.50)	22.90(21.94,24.01)	25.92(25.04,26.40)	25.53(24.64,25.90)	26.86(26.22,27.79)	27.38(26.34,29.18)	−3.69	−2.63	−4.63	−4.48
**GAS5**	23.70(22.62,24.92)	23.54(22.73,24.49)	23.65(22.12,24.24)	21.48(20.52,23.52)	27.92(27.42,28.70)	28.27(27.67,30.55)	0.05	2.06	−4.22	−4.73
**H19**	26.88(24.77,28.53)	27.67(26.79,29.09)	23.09(21.85,23.47)	24.75(22.53,27.28)	45.00(36.97,45.00)	45.00(35.32,45.00)	3.79	2.92	−18.12	−17.33
**LINC00839**	35.79(34.90,37.82)	35.74(34.10,42.21)	30.80(30.04,32.05)	29.67(28.35,31.20)	45.00(44.15,45.00)	45.00(40.74,45.00)	4.99	6.07	−9.21	−9.26
**LINC03067**	45.00(41.75,45.00)	41.65(39.74,45.00)	38.56(37.79,39.67)	37.56(35.92,38.58)	45.00(45.00,45.00)	Undet	6.44	4.09	0	–
**SNHG1**	35.56(33.99,36.47)	34.10(32.11,36.65)	27.49(26.75,28.30)	26.34(24.41,27.58)	45.00(45.00,45.00)	41.14(38.07,45.00)	8.07	7.76	−9.44	−7.04
**ST8SIA6-AS1**	45.00(36.71,45.00)	34.43(33.10,45.00)	30.87(29.77,35.84)	25.06(22.61,31.81)	Undet	45.00(45.00,45.00)	14.13	9.37	–	−10.57

#### 2.2.2 Relative expression levels of LncRNAs.

**Fig 2:**
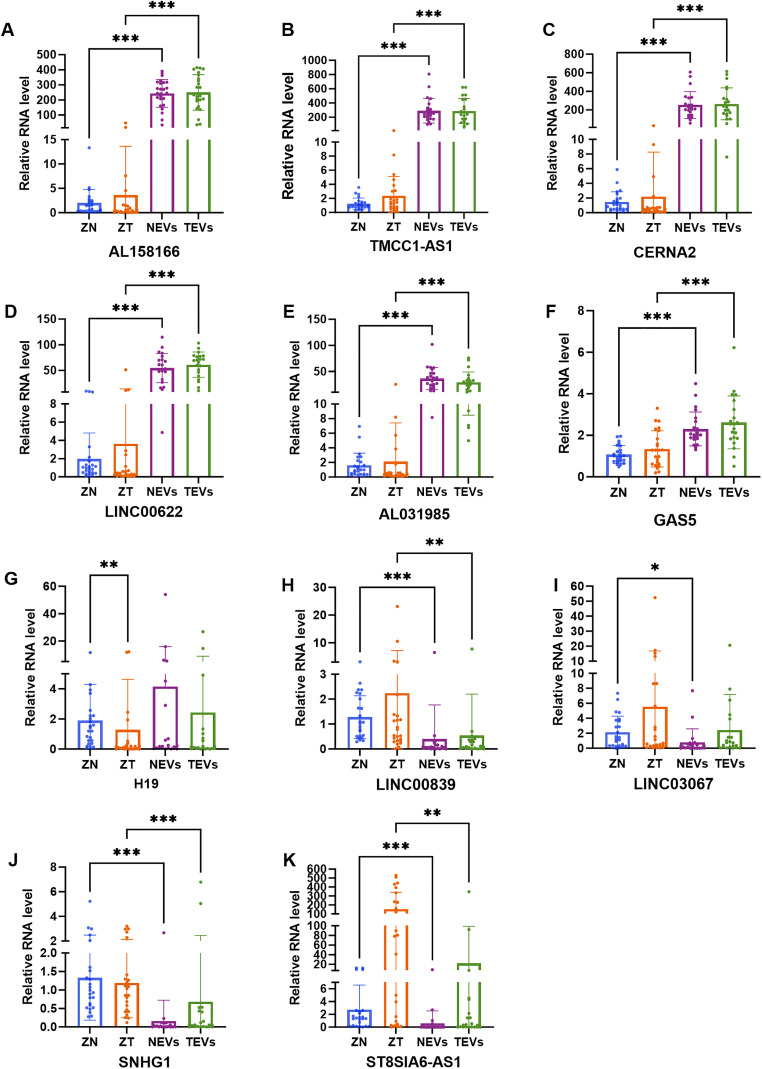
Relative expression levels of lncRNAs in tissue and TIF-EVs. A-G: lncRNAs enriched in TIF-EVs; H-K: lncRNAs not enriched in TIF-EVs.

For relative quantification analysis, the CT values of samples without amplification signals were set to the maximum cycle threshold of 45. The results indicated that the relative expression levels of seven lncRNAs (AL031985, AL158166, CERNA2, TMCC1-AS1, LINC00622, GAS5, and H19) in TIF-sEVs were higher than those in tissues, with statistically significant differences observed for all except H19 (Fig 2A-2G). This finding further suggests that these lncRNAs may be specifically enriched in TIF-sEVs. Conversely, four lncRNAs, including LINC00839, LINC03067, SNHG1, and ST8SIA6-AS1, exhibited high expression levels in tissues (Fig 2H-2K). Except for LINC03067, which showed no significant difference between TEVs and tumor tissues (ZT), the others displayed statistically significant differences. Additionally, the relative expression level of H19 in adjacent non-tumor tissues (ZN) was significantly higher than that in ZT (Fig 2G). In paired t-tests, ST8SIA6-AS1 was significantly higher in ZT than in ZN, while both ST8SIA6-AS1 and SNHG1 were significantly higher in TEVs than in NEVs. However, these differences did not reach statistical significance after multiple comparison correction. Based on this speculation, seven lncRNA molecules, including AL031985 and AL158166, may be selectively packaged and enriched in TIF-sEVs, categorizing them as TIF-sEVs-enriched ncRNAs. Furthermore, H19 may exhibit lower expression levels in HCC, while ST8SIA6-AS1 and SNHG1 may demonstrate higher expression levels in HCC, representing HCC-specific ncRNAs.

### 2.3 Expression characteristics of miRNA molecules

#### 2.3.1 Analysis of miRNA CT values.

Detection of miRNAs in TIF-sEVs and tissues (Table 3) revealed that the CT value results indicated four miRNAs (miR-1224-5p, miR-4306, miR-2114-5p, and miR-483-5p) exhibited CT values that were 2–11 cycles lower in TIF-sEVs compared to tissues. Additionally, three miRNAs (miR-628-5p, miR-1269a, and miR-885-3p) showed only a 0.14–4 cycle reduction in CT values in TIF-sEVs from the paracancerous group compared to their corresponding tissues. In contrast, the reference gene U6 and the remaining 15 miRNAs (including miR-16-5p, miR-21-3p, and miR-21-5p) exhibited CT values that were 0.42–11 cycles higher in TIF-sEVs than in tissues. This suggests that four miRNA molecules, including miR-1224-5p and miR-483-5p, may be selectively packaged and enriched in TIF-sEVs, thus belonging to the category of TIF-sEVs-enriched ncRNAs.

**Table 3:** Quartile statistics of miRNA expression CT values across sample groups.

miRNA	NEVs(18)	TEVs(17)	ZN(8)	ZT(8)	NEVs-ZN	TEVs-ZT
**U6**	19.23(16.82,20.86)	20.56(15.16,25.12)	12.93(12.79, 13.24)	12.76(12.28,13.80)	6.30	7.80
**miR-1224-5p**	24.13(23.41,25.28)	25.17(24.03,28.80)	35.43(34.55,36.29)	35.27(33.44,35.51)	−11.30	−10.10
**miR-1269a**	32.07(30.99,32.93)	31.54(29.60,34.29)	37.04(35.62,37.24)	31.32(28.23,32.88)	−4.97	0.22
**miR-4306**	31.90(31.47,33.57)	33.37(30.71,36.84)	36.44(34.59,37.30)	36.19(35.18,37.31)	−4.54	−2.82
**miR-483-5p**	24.37(23.08,25.78)	28.86(26.19,33.76)	27.62(27.32,28.02)	31.28(27.39,33.31)	−3.25	−2.42
**miR-2114-5p**	30.12(28.94,31.47)	30.75(29.10,33.97)	32.17(31.26,32.93)	32.72(29.77,33.30)	−2.05	−1.97
**miR-628-5p**	28.39(27.82,29.96)	31.32(28.40,33.86)	29.59(28.70,29.90)	29.97(29.51,30.49)	−1.20	1.35
**miR-885-3p**	29.32(27.67,31.02)	32.17(27.40,35.29)	29.44(29.14,30.12)	31.69(29.21,32.72)	−0.12	0.48
**miR-130a-3p**	32.33(29.23,34.82)	35.32(31.30,38.42)	31.91(30.97,32.58)	33.01(32.46,34.15)	0.42	2.31
**miR-148b-5p**	32.46(31.47,33.91)	34.61(31.41,37.08)	32.01(31.68,32.59)	31.82(31.37,32.38)	0.45	2.79
**miR-197-3p**	25.88(24.60,27.59)	28.20(24.23,30.89)	24.17(23.56,24.57)	24.14(23.75,24.88)	1.71	4.06
**miR-885-5p**	24.88(23.02,26.68)	29.37(23.59,31.29)	22.64(22.36,22.86)	24.61(22.35,25.88)	2.24	4.76
**miR-214-3p**	25.07(23.57,26.97)	27.49(25.10,30.78)	22.29(21.52,23.09)	24.46(23.48,26.35)	2.78	3.03
**miR-452-5p**	31.48(30.42,33.96)	32.28(30.22,36.47)	27.70(26.51,29.15)	27.49(24.64,29.02)	3.78	4.79
**miR-21-3p**	29.72(28.50,31.44)	31.42(28.00,34.70)	24.44(23.58,24.86)	22.81(21.53,24.01)	5.28	8.61
**miR-451a**	23.23(20.78,25.58)	26.51(24.87,31.06)	17.59(15.97,18.25)	18.64(17.02,19.99)	5.64	7.87
**miR-122-3p**	24.31(21.47,26.30)	29.12(23.93,31.72)	18.59(18.41,18.94)	19.72(19.42,20.68)	5.72	9.40
**miR-192-5p**	27.43(25.23,28.33)	27.46(26.17,29.60)	21.53(21.15,21.92)	22.43(21.27,24.52)	5.90	5.03
**miR-194-5p**	25.99(23.68,27.01)	28.99(24.13,32.78)	19.65(19.31,20.11)	20.52(19.31,22.76)	6.34	8.47
**miR-142-5p**	31.26(29.48,34.03)	34.95(31.34,37.09)	23.93(23.08,24.79)	25.28(24.47,26.67)	7.33	9.67
**miR-16-5p**	24.06(21.72,26.04)	25.90(22.53,29.65)	15.34(14.38,16.08)	15.55(14.99,15.88)	8.72	10.35
**miR-122-5p**	20.34(17.49,21.42)	24.15(19.68,27.89)	11.59(11.17,12.08)	13.05(12.68,13.93)	8.75	11.10
**miR-21-5p**	23.53(21.78,24.96)	24.90(21.82,26.14)	14.56(13.84,15.35)	13.09(12.41,13.71)	8.97	11.81

#### 2.3.2 Relative expression levels of miRNAs.

**Fig 3:**
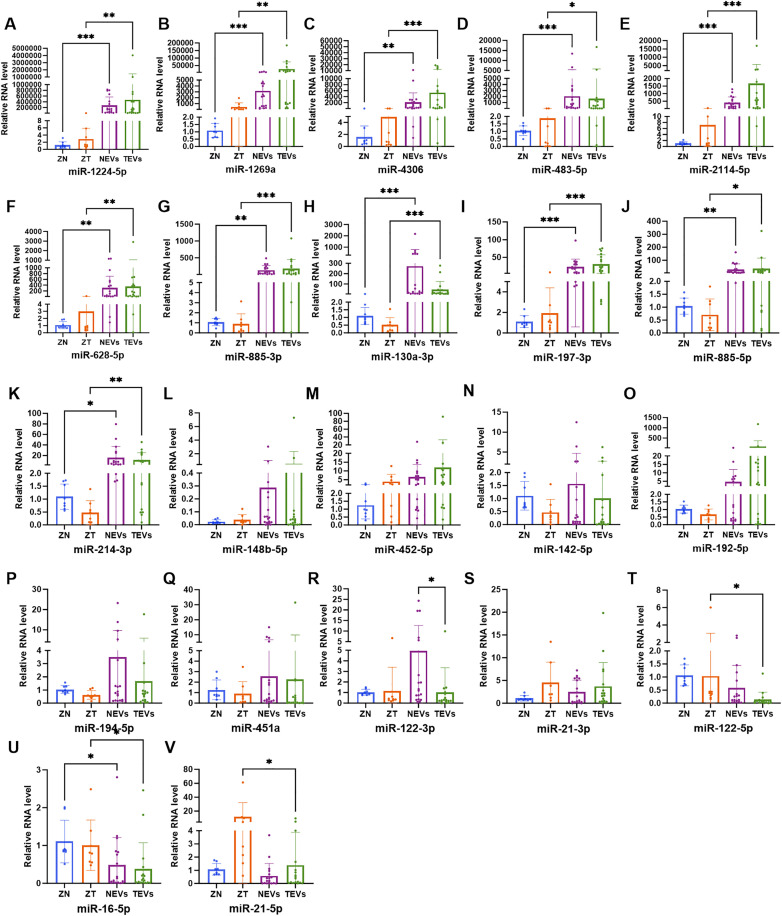
Relative expression levels of miRNAs in tissue and TIF-EVs. A-Q: miRNAs enriched in TIF-EVs; R-S: miRNAs enriched in TIF-EVs only in the non-tumor adjacent group; T-V: miRNAs not enriched in TIF-EVs.

The relative quantitative analysis indicated that 17 miRNAs exhibited elevated relative expression levels in TIF-sEVs compared to tissues (Fig 3A-3Q). Among these, 11 miRNAs—miR-1224-5p, miR-1269a, miR-4306, miR-483-5p, miR-2114-5p, miR-628-5p, miR-885-3p, miR-130a-3p, miR-197-3p, miR-885-5p, and miR-214-3p—demonstrated statistically significant differences (Fig 3A-3K). It is hypothesized that these miRNAs may be relatively enriched in TIF-sEVs. miR-122-3p and miR-21-3p showed higher relative expression levels exclusively in NEVs compared to ZN (Fig 3R-3S). In contrast, three miRNAs, namely miR-16-5p, miR-21-5p, and miR-122-5p (Fig 3T-3V), exhibited higher relative expression levels in tissues, with these three miRNAs demonstrating statistically significant differences in the TEVs versus ZT comparison. However, in the NEVs versus ZN comparison, only miR-16-5p displayed a statistically significant difference. Furthermore, the relative expression of miR-122-3p in NEVs was significantly higher than that in TEVs (Fig 3R). Based on this hypothesis, 11 miRNA molecules, including miR-1224-5p and miR-483-5p, may be selectively packaged and enriched in TIF-sEVs, categorizing them as TIF-sEVs-enriched ncRNAs. Additionally, miR-122-3p may exhibit lower expression levels in HCC, suggesting its role as an HCC-specific ncRNA.

### 2.4 Receiver operating characteristic (ROC) curve analysis

**Fig 4:**
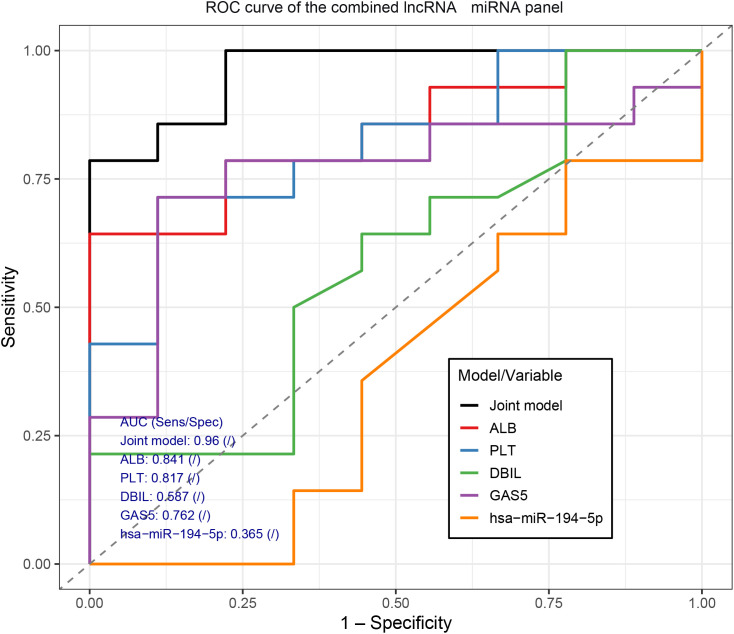
ROC performance analysis of a combined model for predicting HCC based on LASSO regression.

To preliminarily evaluate diagnostic efficacy, 23 patients were categorized into HCC and non-HCC groups. LASSO regression was performed on 33 candidate ncRNAs and clinical parameters. The final model retained five key variables: albumin (ALB), platelets (PLT), direct bilirubin (DBIL), lncRNA GAS5, and miRNA hsa-194-5p (Fig 4). This composite indicator demonstrated a significantly improved predictive performance for HCC, yielding an area under the curve (AUC) of 0.960 (95% confidence interval 0.89–1.00). Bootstrap testing further substantiated the superiority of the joint model, as its diagnostic performance (AUC = 0.960) was markedly higher than that of the best single biomarker, miR-483-5p (AUC = 0.714, D = 2.15, P = 0.032). At the optimal threshold of 0.628, the joint model reached a sensitivity of 78.6% and a specificity of 100.0%. The individual AUC values for the five variables in the model were as follows: ALB 0.841, PLT 0.817, DBIL 0.587, GAS5 0.762, and hsa-miR-194-5p 0.365. The individual diagnostic performances of all non-coding RNAs and the corresponding ROC curves are detailed in the Supplementary Materials (S1 File. ROC analyses for ncRNA).

## 3 Discussion

HCC is a malignancy with high global incidence and mortality rates. Differentialdiagnosis and precise treatment are key to improving patient prognosis [28]. Although advances in neoadjuvant and conversion therapies have improved outcomes for patients with intermediate to advanced stages, there is still a lack of widely accepted biomarkers that can effectively guide clinical decision-making to accurately identify beneficiaries, leading to a degree of empirical and uncertain treatment strategies [29–31]. Therefore, there is an urgent need to identify highly efficient and specific biomarkers to achieve individualized precision therapy. In this context, EVs, as nanoscale carriers with a phospholipid bilayer structure, have emerged as a highly promising source of biomarkers due to their high stability [2,30], minimally or non-invasive accessibility from Bf-EVs, and ability to carry bioactive molecules such as proteins and nucleic acids that reflect the state of their parental cells [3,5–11].

However, Bf-EVs (e.g., plasma EVs) originate from various cells throughout the body, resulting in low tissue specificity and susceptibility to background signal interference during detection, which limits their specificity and sensitivity. In contrast, TIF is rich in EVs directly released by local cells, offering closer proximity to the primary lesion. Their molecular features more directly and earlier reflect the pathophysiological state of the tissue, avoiding the “dilution” of signals by systemic background encountered in blood tests, making them an ideal source for discovering highly specific biomarkers. Currently, TIF-EVs have demonstrated diagnostic potential in various fields, including HCC, cutaneous melanoma, burn assessment, and neurodegenerative diseases [16–18,22–25].

Therefore, this study focuses on hepatic tissue interstitial fluid, aiming to establish a method for isolating and characterizing TIF-EVs applicable to clinical liver tissue samples, and to screen for potential HCC diagnostic biomarkers by detecting ncRNAs via qRT-PCR. Based on literature [13,16,20,21] and preliminary experiments, this study optimized conditions such as sample selection, enzyme types, concentrations, digestion time, centrifugation, and filtration. The final protocol involved digestion with Collagenase D (final concentration 2 mg/mL) and DNase I (final concentration 40 U/mL) at 37°C for 30 minutes, followed by differential centrifugation, filtration through a 0.22 µm filter, and ultracentrifugation at 120,000 × g for 2 hours.

The method used in this study was further compared with those reported in the literature [13,16,26,27]. Regarding sample selection, Chen J et al. [26] and Wang X et al. [27] reported no significant differences between TIF-sEVs isolated from tissues stored at −80°C and fresh tissues, although the total protein content was slightly lower in the former. To ensure TIF-sEVs were in optimal condition for subsequent analyses, this study used fresh tissues obtained directly from the operating room for TIF-sEV extraction. The results showed that the particle concentration and total protein content of TIF-sEVs per unit weight of liver tissue were higher than those reported in the literature [26,27].

Regarding enzymatic digestion conditions, Chen J et al. [26] and Wang X et al. [27] reported optimized conditions for extracting TIF-EVs from HCC tissue as digestion with Collagenase D (4 mg/mL) and DNase I (80 U/mL) for 20 minutes. Lin J et al. [16] used a combination of Collagenase I (100 U/mL), Collagenase IV (50 U/mL), Hyaluronidase (50 U/mL), and DNase I (0.1 mg/mL), with incubation at 37°C for 30–60 minutes to extract TIF-EVs from HCC and adjacent tissues. While all these methods can yield TIF-EVs, the conditions and yields vary significantly, indicating a need for further standardization and comparative evaluation. This study primarily referred to the conditions used by Crescitelli R et al. [13] for extracting melanoma TIF-EVs, which involved Collagenase D (2 mg/mL) and DNase I (40 U/mL) with a 30-minute incubation.

Regarding differential centrifugation, Chen J et al. [26] and Wang X et al. [27] noted that a white layer (suspected lipid layer), often appeared on the surface of the supernatant. To minimize its interference, they adopted a stepwise centrifugation strategy. This study further referenced the centrifugal forces used by Lin J et al. [16], establishing differential centrifugation steps (500 × g, 3,000 × g, and 16,500 × g), with each step repeated once for 20 minutes, which effectively removed the surface white material.

Regarding ultracentrifugation conditions, reported parameters in the literature vary. Lin J et al. [16] used 100,000 × g for 2 hours, Chen J et al. [26] used 100,000 × g for 70 minutes, and Wang X et al. [27] used 120,000 × g for 60 minutes, with the latter two including a repeat of the ultracentrifugation step after resuspension in PBS. Based on preliminary BCA and WB results showing no significant difference between one or two ultracentrifugation steps, this study ultimately adopted 120,000 × g for 2 hours.

Regarding pellet resuspension, Chen J et al. [26] resuspended TIF-EVs from 100 mg of HCC tissue in 100 µL of PBS, while Wang X et al. [27] resuspended TIF-EVs from 400 mg of HCC tissue in 50 µL of PBS. Both studies filtered the resuspended EVs through a 0.22 µm bacterial filter, with Chen J et al. [26] aiming to ensure sterility for subsequent cell function experiments. Considering the volume and concentration requirements for subsequent qRT-PCR analysis of TIF-sEVs in this study, and the practical difficulty of resuspending the TIF-EVs pellet from 400 mg of liver tissue in 50 µL PBS, the order of operations was adjusted. The supernatant after differential centrifugation was first transferred to sterile ultracentrifuge tubes through a 0.22 µm filter, and the volume was adjusted with PBS before ultracentrifugation. The final pellet was resuspended in 200 µL PBS. This method yielded higher particle concentrations and total protein concentrations as measured by NTA and BCA, compared to the optimal extraction yields reported in the literature [26,27].

In terms of Western blot analysis, this study not only utilized CD9, CD63, and TSG101, as seen in previous studies [16,26,27], but also examined additional EV markers, including Alix, HSP70, CD81. To trace the tissue origin of TIF-sEVs, we investigated hepatocyte markers ASGPR and ALB, the cholangiocyte marker CK19, and the vascular endothelial marker CD34. With equal loading, the EV marker protein CD63 were strongly positive in TIF-sEVs, suggesting higher abundance of the protein in TIF-sEVs than in tissue. Alix bands of different sizes were detected in different samples, suggesting possible phosphorylation or cleavage isoforms of this protein [32]. TSG101 predominantly appeared as a 47-kDa band in 30-minute TEVs, while both 52-kDa and 47-kDa bands were observed in 60-minute TEVs, indicating that digestion time may influence its molecular form. HSP70 expression in TIF-sEVs was significantly weaker than in tissue or even the ultracentrifugation supernatant (US), showing low specificity in TIF-sEV detection and was undetectable in some TIF-NEVs. Existing research has found that EVs released by tumor cells express the stress protein HSP70 on their membrane, whereas EVs from normal cells do not [33]. The expression of CD9 was influenced by both digestion time and vesicle type: the signal was clear in 30-minute TEVs, weakened in 60-minute TEVs, and was weaker in NEVs compared to TEVs, with some samples showing no detectable signal. CD9 was detected only in liver tissues pathologically diagnosed as hemangioma and cholangiocarcinoma, and their corresponding TIF-EVs. CD9 expression was influenced by both digestion time and vesicle type. The signal was prominent in 30-minute TEVs, diminished in 60-minute TEVs, and was weaker in NEVs compared to TEVs, with some samples exhibiting no detectable signal. Alvarez-Rodriguez M et al. also reported failure to detect EVs carrying CD9 (and CD44) in chicken semen [34]. This indicates that TIF-sEVs do not carry all EV protein markers, exhibiting a degree of heterogeneity. CD81, similar to literature reports [13], was almost undetectable, likely due to enzyme effects. With respect to source markers, both ASGPR and ALB exhibited positive signals, displaying stronger intensities than the cell control. In contrast, CK19 and CD34 signals were weaker than those observed in the cell control, indicating that hepatocytes represent a primary source of TIF-sEVs. Building on this observation, we further assessed the expression profile and potential diagnostic significance of ncRNAs in TIF-sEVs.

This study further evaluated the expression levels of 11 lncRNAs and 22 miRNAs in TIF-sEVs and tissues using qRT-PCR, comparing their CT values and relative quantification. Lower CT values provided direct evidence for high-concentration enrichment of ncRNAs in vesicles. Relative quantification further clarified the fold-change differences in expression between vesicles and tissues, identifying two molecular groups: those significantly enriched in TIF-sEVs (e.g., *TMCC1-AS1*, *miR-483-5p*) and those retained specifically in tissues (e.g., *SNHG1*, *miR-16-5p*). These results confirm potential distribution differences of ncRNAs between vesicles and tissues, suggesting diagnostic value in such differences.

Analyzing clinical diagnostic potential, TIF-sEV-enriched ncRNAs, due to their high specificity and secretory nature, are ideal candidate indicators for constructing liquid biopsy models. First, *AL031985* and *TMCC1-AS1* have been reported in EVs and show potential as prognostic markers for HCC [35]. Similarly, *AL158166* [36] and *miR-1224-5p* [37] have also been associated with HCC prognosis. *miR-4306* has also been reported to have potential as a prognostic marker for HCC [38]. This supports the possibility of TIF-EVs enriched ncRNAs serving as HCC biomarkers. Second, *miR-483-5p* [16] demonstrated highly consistent diagnostic value in this study and published literature. This study found that this molecule (previously identified through high-throughput sequencing in plasma and serum EVs in our laboratory as a differentially expressed molecule) had the highest single-indicator diagnostic efficacy among the tested ncRNAs (AUC = 0.714). This finding aligns with the conclusions of Lin et al. [16]: their work confirmed that *miR-483-5p* is significantly upregulated in HCC tissue TIF-sEVs and plasma EVs, promotes HCC cell proliferation, and exhibits good diagnostic performance for HCC in plasma EVs (AUC = 0.898), which improved to 0.949 when combined with alpha-fetoprotein (AFP). This consistency further validates the value of TIF-EV-enriched ncRNAs as HCC biomarkers. Third, miR-122-3p [39] was significantly enriched in non-tumor adjacent tissue TIF-sEVs, reflecting tumor microenvironment heterogeneity. Beyond individual biomarkers, the LASSO-derived combined model identified GAS5 and miR-194-5p as key ncRNA variables alongside clinical parameters.

The LASSO-derived model retained two ncRNA variables, GAS5 and miR-194-5p, alongside clinical parameters. GAS5 is a documented HCC tumor suppressor that inhibits proliferation and invasion via vimentin regulation [40], with its downregulation promoting drug resistance through PTEN/PI3K/AKT signaling [41]. Circulating GAS5 further shows dynamic fluctuation during sorafenib therapy, supporting its biomarker utility [42]. MiR-194-5p, identified from our prior serum sEV sequencing across the HBV-related disease spectrum, has been implicated in hepatocarcinogenesis through the MCM3AP-AS1/FOXA1 axis [43] and HCC immune evasion via the PCED1B-AS1/PD-L1/PD-L2 pathway [44]. In chronic hepatitis B, serum miR-194 correlates with viral load and treatment response through SOCS2/JAK-STAT modulation [45,46], while HBV itself drives host miRNA dysregulation targeting apoptosis genes [47]. The co-selection of these two ncRNAs likely reflects complementary pathobiological signals—GAS5 capturing tumor suppressor loss and miR-194-5p reflecting viral-driven oncogenesis—thereby enhancing diagnostic performance beyond individual biomarkers. Finally, a multi-indicator combined model constructed in this study based on LASSO regression, integrating vesicle ncRNAs and clinical parameters (e.g., ALB, PLT), achieved superior diagnostic performance (AUC = 0.960), highlighting the significant advantage of combining vesicle biomarkers with clinical parameters.

This study has certain limitations: First, at the methodological level, the enzymatic digestion method used to isolate EVs from tumor interstitial fluid has inherent drawbacks. The enzymatic digestion process may induce minor cellular damage and the release of artificial vesicles, complicating the rigorous distinction between ‘in situ pre-existing’ and ‘procedure-generated’ EVs at the single-particle level. Second, the sample size is relatively limited, and inter-individual biological variability may affect the generalizability and stability of the results. Finally, the enrichment status and diagnostic performance of the candidate molecules screened from TIF-sEVs in patient serum or plasma and their EVs have not been evaluated. Therefore, future research could simultaneously collect “tissue–TIF–serum–plasma” samples, conduct comparative multi-omics analyses, and validate findings through large-scale, multi-center cohorts. This would systematically assess the clinical translation potential of candidate biomarkers, providing new strategies for the differential diagnosis and precise monitoring of HCC.

## 4 Materials and methods

### 4.1 Patient information

Specimens were obtained from 23 patients who underwent surgical resection at the Department of Hepatobiliary Surgery of Tianjin Third Central Hospital from 01/04/202417/03/2025. Detailed clinical data were recorded (S2 File, S1-S4 Tables File), which included 14 cases pathologically confirmed as HCC, 5 as cholangiocarcinoma (CCA), 1 as focal nodular hyperplasia (FNH), 1 as inflammatory pseudotumor (IPT), and 2 as chronic hepatitis (CH). Paired non-tumor adjacent tissue and tumor tissue samples were collected from all cases. This study was approved by the Medical Ethics Committee of Tianjin Third Central Hospital (Approval No.: IRB2024-014-1). Given that this study exclusively utilizes discarded surgical tissue samples and de-identified clinical data, and poses no additional risk to the patients, the Ethics Committee has approved a waiver of individual patient informed consent.

### 4.2 Data sources

The candidate ncRNAs analyzed in this study were derived from two independent datasets.

(1) TCGA-LIHC cohort (https://portal.gdc.cancer.gov/)
RNA-seq data of 374 tumor tissues and 50 adjacent normal tissues were obtained from The Cancer Genome Atlas Liver Hepatocellular Carcinoma (TCGA-LIHC) project. Differential expression analysis and survival analysis identified 11 lncRNAs (AL031985.3, AL158166.2, CERNA2, GAS5, H19, LINC00622, LINC00839, LINC03067, SNHG1, ST8SIA6-AS1, and TMCC1-AS1) and 14 miRNAs (hsa-miR-122-3p, hsa-miR-122-5p, hsa-miR-16-5p, hsa-miR-21-3p, hsa-miR-21-5p, hsa-miR-130a-3p, hsa-miR-197-3p, hsa-miR-214-3p, hsa-miR-451a, hsa-miR-452-5p, hsa-miR-628-5p, hsa-miR-1269a, hsa-miR-1224-5p, and hsa-miR-2114-5p).
(2) In-house sequencing data (GEO: GSE302990. https://www.ncbi.nlm.nih.gov/geo/query/acc.cgi?acc=GSE302990)
Serum samples were collected from patients with HBV-related liver diseases, including chronic hepatitis B, cirrhosis, benign liver disease, and hepatocellular carcinoma. Small extracellular vesicles were isolated and subjected to whole-transcriptome sequencing. Differential abundance analysis across disease stages identified eight additional miRNAs: miR-142-5p, miR-148b-5p, miR-192-5p, miR-194-5p, miR-483-5p, miR-885-3p, miR-885-5p, and miR-4306. Raw sequencing data have been deposited in the Gene Expression Omnibus under accession number GSE302990.

In total, 33 candidate ncRNAs (11 lncRNAs and 22 miRNAs) were included in subsequent qRT‑PCR validation and LASSO regression analysis.

### 4.3 Isolation and purification of TIF-sEVs

**Fig 5:**
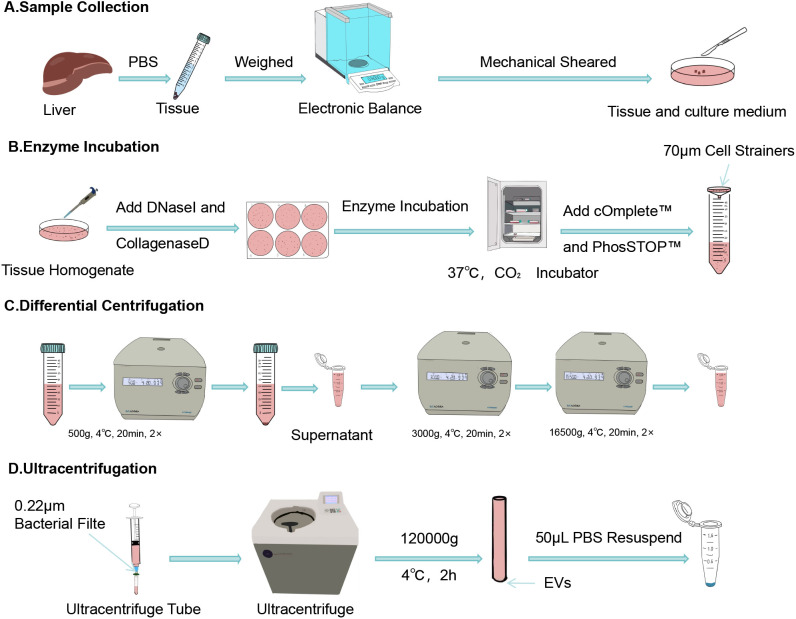
Schematic illustration of the isolation protocol for extracellular vesicles derived from interstitial fluid of liver tissue.

The extraction and purification of TIF-sEVs are depicted in Fig 5. In summary, 0.4 g of hepatocellular carcinoma tissue and the corresponding paired non-tumor adjacent tissue were weighed and minced into pieces measuring 1–2 mm^3^. Minimum Essential Medium (MEM) (Thermo Fisher Scientific, Waltham, MA, USA) was added at a ratio of 0.2 g of tissue per mL of medium. Subsequently, 20 µL of Collagenase D (Roche, Basel, Switzerland) and 20 µL of DNase I (Roche, Basel, Switzerland) were introduced per mL of the tissue-medium mixture, which was then homogenized and incubated at 37°C for 30 minutes in a CO_2_ incubator. The reaction was terminated by the addition of 40 µL of cOmplete™ Mini EDTA-free protease inhibitor cocktail (Roche) and 50 µL of PhosSTOP™ phosphatase inhibitor cocktail (Roche). All enzymes were prepared following the manufacturer’s instructions. Undigested tissue debris was removed by filtration through a Falcon® Cell Strainer (70 µm, Corning, NY, USA). The filtrate was subjected to sequential centrifugation at 4°C: first at 500 × g for 20 minutes, then at 3,000 × g for 20 minutes, and finally at 16,500 × g for 20 minutes. Each centrifugation step was performed twice to eliminate cell debris, apoptotic bodies, and large microvesicles. The supernatant was collected, filtered through a 0.22 µm filter (Millipore, Billerica, MA, USA), and subjected to ultracentrifugation at 120,000 × g for 2 hours at 4°C. The resulting pellet, which contained TIF-sEVs, fell within the size range defined for sEVs according to the MISEV2023 guidelines. Finally, the pellet was resuspended in 200 µL of Phosphate Buffered Saline without Ca^2+^ or Mg^2+^ (PBS, Precision Biomedicals Co. Ltd, China), aliquoted, and stored at −80°C.

### 4.4 Morphological observation by TEM

Thirty microliters of the TIF-sEV suspension were adsorbed onto a copper grid for 2 minutes at room temperature. The sample was then negatively stained with phosphotungstic acid (pH 6.8) for 5 minutes in the dark at room temperature, followed by drying for 10 minutes under an incandescent lamp. Images were acquired using a Hitachi HT7700 transmission electron microscope (Hitachi, Tokyo, Japan). This experiment was conducted at the Large-scale Instrument Public Service Platform of Tianjin Medical University.

### 4.5 NTA

TIF-sEVs samples were appropriately diluted with pre-chilled PBS and slowly injected into a Malvern Panalytical Nanosight LM10 instrument (Malvern Panalytical, Malvern, UK) using a 1 mL syringe. Videos were captured and analyzed to determine the particle size distribution and concentration. This experiment was conducted at the Large-scale Instrument Public Service Platform of Tianjin Medical University.

### 4.6 Protein extraction

Tissue samples were thoroughly ground, and a Western & IP cell lysis buffer (200 mg/mL, Beyotime Biotechnology, China), pre-supplemented with PMSF (Beyotime Biotechnology), was added. The mixture was incubated at 4°C for 30 minutes, with vortexing performed every 10 minutes. Following centrifugation at 4°C and 12,000 × g for 15 minutes, the supernatant was collected as the tissue protein lysate. For TIF-sEV and ultracentrifugation supernatant (US) samples, in order to avoid introducing additional liquid, proteins were released by directly adding 5 × Laemmli loading buffer (Thermo Fisher Scientific) instead of the conventional lysis buffer. Protein concentrations for all samples were determined using a BCA Protein Assay Kit (Beyotime Biotechnology, Shanghai, China).

### 4.7 WB analysis

Samples were mixed with 5 × Laemmli loading buffer at a 4:1 ratio to achieve a final concentration of 1 × . The mixtures were heated at 100°C for 10 minutes to facilitate denaturation. After cooling, 30 µg of total protein (as determined by BCA assay) was loaded per lane, alongside a PageRuler™ Prestained Protein Ladder (Thermo Fisher Scientific) serving as a molecular weight marker. Proteins were separated by electrophoresis on a 10% pre-cast PAGE gel (YESEN, Shanghai, China) under constant voltage conditions: 80 V for 30 minutes followed by 120 V for 60 minutes. Subsequently, proteins were transferred to a 0.45 µm PVDF membrane (Millipore) using the wet transfer method at a constant current of 200 mA for 60 minutes with Western transfer buffer (Beyotime Biotechnology). The membrane was then blocked with 5% skim milk in TBST (YESEN) at room temperature for 1 hour. Following this, it was incubated with primary antibodies diluted in TBST (S2 File, S1-S4 Tables File), overnight at 4°C. After washing with TBST (5 × 6 minutes), the membrane was incubated with Goat Anti-Rabbit IgG HRP (1:5000, Affinity, Jiangsu, China) at room temperature for 1 hour, followed by another wash with TBST (5 × 6 minutes). Finally, protein bands were visualized using BeyoECL Plus chemiluminescence reagent (Beyotime Biotechnology), and images were captured and analyzed using a FluorChem-M multi-gel imaging system (Protein Simple, CA, USA).

### 4.8 RNA extraction and quality control

RNA was extracted from TIF-sEV samples using a Viral Genomic DNA/RNA Rapid Extraction Kit (RA108, Biomed, Beijing, China). Total RNA from tissue samples was extracted using Trizol reagent (Takara, Dalian, China) in accordance with the manufacturer’s protocol. All extraction steps were conducted under RNase-free conditions to prevent contamination. The purity and concentration of RNA were evaluated using 1% agarose gel electrophoresis and a NanoDrop ND-2000 spectrophotometer (Life Technologies, Grand Island, NY, USA).

### 4.9 qRT-PCR detection of lncRNAs

cDNA for lncRNA analysis was synthesized using GoScript™ Reverse Transcriptase (Promega, WI, USA). Real-time quantitative PCR was performed using TB Green® Premix Ex Taq™ II FAST qPCR (CN830A, Takara) with SYBR Green detection. The cDNA synthesis steps were as follows: 2.5 µg of RNA was mixed with RNase-free H_2_O to achieve a final volume of 22 µL, followed by the addition of 2 µL of Oligo dT/pd(N)_6_ mixed primers. The mixture was heated at 70°C for 5 minutes and then placed on ice for 3 minutes. Subsequently, 8 µL of 5X RT Buffer, 2 µL of 10 mM dNTPs, 4.8 µL of 25 mM MgCl_2_, and 1.2 µL of reverse transcriptase were added, resulting in a total volume of 40 µL. The reaction conditions included incubation at 25°C for 5 minutes, 42°C for 60 minutes, and 70°C for 5 minutes. Synthesized cDNA templates were either stored at −20°C or used directly for qPCR. The qRT-PCR amplification mixture comprised 12.5 µL of 2 × TB Green Premix Ex Taq II FAST qPCR, 0.5 µL each of forward and reverse primers (S2 File, S1-S4 Tables File and S3 File ncRNA diff_expression_ survival_analysis), 2.0 µL of cDNA template, and 9.5 µL of sterile distilled water, totaling 25 µL. No-template controls (NTC) were included in each reaction to monitor for contamination. Amplification conditions consisted of pre-incubation at 25°C for 2 minutes and 95°C for 30 seconds, followed by 45 cycles of 95°C for 10 seconds and 62°C for 20 seconds, concluding with a melt curve analysis to confirm a single specific Tm peak. Specificity was further verified through agarose gel electrophoresis and sequencing of PCR products. GAPDH served as the reference gene, and the Ct values for each lncRNA were analyzed. Following validation of amplification efficiency, relative quantification was performed using the 2^(-ΔΔCt) method. All real-time PCR experiments were conducted on a ViiA 7 Dx Real-Time PCR System (Thermo Fisher Scientific).

### 4.10 qRT-PCR detection of mirnas

The miDETECT A Track miRNA RT-qPCR Starter Kit (C10712, RiboBio, Guangzhou, China) was employed for the detection of miRNA. The experimental procedure comprised the following steps: (1) Poly(A) tailing reaction: A mixture of 2 µL of 5 × Poly(A) Polymerase Buffer, 1 µL of Poly(A) Polymerase, and 7 µL of EVs RNA was incubated at 37°C for 1 hour. (2) Reverse transcription reaction: A combination of 4 µL of 5 × RTase Buffer, 4 µL of RTase mix, and 2 µL of miDETECT A Track™ Uni-RT Primer was added to 10 µL of the Poly(A) tailing product and incubated at 42°C for 60 minutes, followed by incubation at 72°C for 10 minutes. (3) qPCR reaction: A 20 µL mixture was prepared, consisting of 10 µL of 2 × SYBR Green Mix, 0.17 µL of a 20 µM miRNA-specific forward primer, 0.3 µL of a 10 µM miDETECT A Track™ Uni-Reverse Primer, 0.4 µL of 50 × Rox Dye II, 7.13 µL of RNase-free water, and 2 µL of cDNA template. The amplification conditions included a pre-incubation step at 25°C for 2 minutes and 95°C for 10 minutes, followed by 50 cycles of 95°C for 8 seconds and 61.5°C for 20 seconds, concluding with a melt curve analysis. U6 snRNA was utilized as the reference RNA. The Ct values for each miRNA (S2 File, S1-S4 Tables File and S3 File ncRNA diff_expression_ survival_analysis),were analyzed, and relative quantification was performed using the 2^(-ΔΔCt) method.

### 4.11 LASSO regression and diagnostic model development

ROC curves were utilized to assess the diagnostic performance of biomarkers for HCC. A total of twenty-three patients were categorized into HCC and non-HCC groups. The LASSO regression was applied for feature selection, with the optimal penalty parameter λ being determined through 10-fold cross-validation. A combined diagnostic logistic regression model was developed based on the selected variables, and the AUC along with its 95% confidence interval was computed. To ascertain whether the diagnostic performance of the combined model significantly surpassed that of individual biomarkers, the Bootstrap method (1,000 resampling iterations) was employed to evaluate the differences in AUC between the groups. All analyses were conducted using R software version 4.5.1.

### 4.12 Statistical analysis

All data were analyzed using GraphPad Prism version 10.4.1 and R version 4.5.1. The distribution characteristics of continuous variables were initially assessed for normality and log-normality using the Normality and Lognormality Tests, which included the Shapiro-Wilk and Kolmogorov-Smirnov tests. Data are presented as median (interquartile range) [M (Q1, Q3)]. For normally distributed data, one-way analysis of variance (ANOVA) with Tukey’s post-hoc test was employed for multi-group comparisons. In contrast, for non-normally distributed data, the Kruskal-Wallis test with Dunn’s post-hoc correction was utilized. The threshold for statistical significance was set at P < 0.05, with designations of *P < 0.05, **P < 0.01, and ***P < 0.001.

## Supporting information

S1 Fig Characterization of TIF-EVs. (A) Electron micrograph of TIF-EVs stored at 4°C for one week; (B) Electron micrograph of TIF-EVs stored at −80°C for one week; (C) Electron micrograph of TIF-EVs obtained after a single differential centrifugation step; (D) Electron micrograph of TIF-EVs obtained after repeating the differential centrifugation step once; (E) Western blot analysis of TIF-EVs digested with proteinase for 0, 30, and 60 min. NEVs (non-tumor adjacent tissue-derived TIF-sEVs) and TEVs (tumor tissue-derived TIF-sEVs) were analyzed, with Huh7-derived EVs serving as positive control and Huh7 cell lysate as negative control.
(TIF)

S1 File ROC analyses for ncRNAs. (PDF)

S2 File Supplementary tables (Tables S1–S4). (PDF)

S3 File Differential Expression Analysis for ncRNAs. (PDF)

S4 File Survival analysis for ncRNAs. (PDF)

S1 Text Raw images. (PDF)

## References

[1] Cheng L, Hill AF. Therapeutically harnessing extracellular vesicles. Nat Rev Drug Discov. 2022;21(5):379–99. doi: 10.1038/s41573-022-00410-w 35236964

[2] Welsh JA, Goberdhan DCI, O’Driscoll L, Buzas EI, Blenkiron C, Bussolati B, et al. Minimal information for studies of extracellular vesicles (MISEV2023): From basic to advanced approaches. J Extracell Vesicles. 2024;13(2):e12404. doi: 10.1002/jev2.12404 PMC10850029 38326288

[3] Su X, Wang H, Li Q, Chen Z. Extracellular Vesicles: A Review of Their Therapeutic Potentials, Sources, Biodistribution, and Administration Routes. Int J Nanomedicine. 2025;20:3175–99. doi: 10.2147/IJN.S502591 40098717 PMC11913029

[4] Zhou Y, Zhang Y, Gong H, Luo S, Cui Y. The Role of Exosomes and Their Applications in Cancer. Int J Mol Sci. 2021;22(22):12204. doi: 10.3390/ijms222212204 34830085 PMC8622108

[5] Khoo A, Govindarajan M, Qiu Z, Liu LY, Ignatchenko V, Waas M, et al. Prostate cancer reshapes the secreted and extracellular vesicle urinary proteomes. Nat Commun. 2024;15(1):5069. doi: 10.1038/s41467-024-49424-5 38871730 PMC11176296

[6] Grange C, Bussolati B. Extracellular vesicles in kidney disease. Nat Rev Nephrol. 2022;18(8):499–513. doi: 10.1038/s41581-022-00586-9 35641620 PMC9152665

[7] Chen Y-Q, Zheng L, Zhou J, Wang P, Wang L, Zhang Y, et al. Evaluation of plasma LC3B+extracellular vesicles as a potential novel diagnostic marker for hepatocellular carcinoma. Int Immunopharmacol. 2022;108:108760. doi: 10.1016/j.intimp.2022.108760 35398623

[8] Rui T, Zhang X, Guo J, Xiang A, Tang N, Liu J, et al. Serum-Exosome-Derived miRNAs Serve as Promising Biomarkers for HCC Diagnosis. Cancers (Basel). 2022;15(1):205. doi: 10.3390/cancers15010205 36612201 PMC9818484

[9] Jurj A, Paul D, Calin GA. Extracellular Vesicles in cancer: from isolation and characterization to metastasis, drug resistance, and clinical applications. BMC Cancer. 2025;25(1):1154. doi: 10.1186/s12885-025-14375-7 40629268 PMC12235885

[10] Li D, Gao Y, Wang C, Hu L. Proteomic and phosphoproteomic profiling of urinary small extracellular vesicles in hepatocellular carcinoma. Analyst. 2024;149(17):4378–87. doi: 10.1039/d4an00660g 38995156

[11] Shah R, Patel T, Freedman JE. Circulating Extracellular Vesicles in Human Disease. N Engl J Med. 2018;379(10):958–66. doi: 10.1056/NEJMra1704286 30184457

[12] Pait MC, Kaye SD, Su Y, Kumar A, Singh S, Gironda SC, et al. Novel method for collecting hippocampal interstitial fluid extracellular vesicles (EVISF) reveals sex-dependent changes in microglial EV proteome in response to Aβ pathology. J Extracell Vesicles. 2024;13(1):e12398. doi: 10.1002/jev2.12398 38191961 PMC10774707

[13] Crescitelli R, Lässer C, Jang SC, Cvjetkovic A, Malmhäll C, Karimi N, et al. Subpopulations of extracellular vesicles from human metastatic melanoma tissue identified by quantitative proteomics after optimized isolation. J Extracell Vesicles. 2020;9(1):1722433. doi: 10.1080/20013078.2020.1722433 32128073 PMC7034452

[14] Crescitelli R, Lässer C, Lötvall J. Isolation and characterization of extracellular vesicle subpopulations from tissues. Nat Protoc. 2021;16(3):1548–80. doi: 10.1038/s41596-020-00466-1 33495626

[15] Wang J, Li L, Zhang Z, Zhang X, Zhu Y, Zhang C, et al. Extracellular vesicles mediate the communication of adipose tissue with brain and promote cognitive impairment associated with insulin resistance. Cell Metab. 2022;34(9):1264-1279.e8. doi: 10.1016/j.cmet.2022.08.004 36070680

[16] Lin J, Lin W, Bai Y, Liao Y, Lin Q, Chen L, et al. Identification of exosomal hsa-miR-483-5p as a potential biomarker for hepatocellular carcinoma via microRNA expression profiling of tumor-derived exosomes. Exp Cell Res. 2022;417(2):113232. doi: 10.1016/j.yexcr.2022.113232 35659970

[17] Smith SM, Kumari A, Marvar JP, Onukwugha N-E, Kang Y-T, Nagrath S. Stellate silicon microneedles for rapid point-of-care melanoma exosome isolation and detection via a lateral flow assay. Biosens Bioelectron. 2025;285:117560. doi: 10.1016/j.bios.2025.117560 40403613

[18] Foessl I, Haudum CW, Vidakovic I, Prassl R, Franz J, Mautner SI, et al. miRNAs as Regulators of the Early Local Response to Burn Injuries. Int J Mol Sci. 2021;22(17):9209. doi: 10.3390/ijms22179209 34502118 PMC8430593

[19] Jonak ST, Liu Z, Liu J, Li T, D’Souza BV, Schiaffino JA, et al. Analyzing bronchoalveolar fluid derived small extracellular vesicles using single-vesicle SERS for non-small cell lung cancer detection. Sens Diagn. 2022;2(1):90–9. doi: 10.1039/d2sd00109h 36741247 PMC9850358

[20] Gorini F, Coada CA, Monesmith S, De Leo A, de Biase D, Dondi G, et al. Distinctive features of blood- and ascitic fluid-derived extracellular vesicles in ovarian cancer patients. Mol Med. 2025;31(1):143. doi: 10.1186/s10020-025-01177-7 40259212 PMC12010555

[21] Quiralte M, Barquín A, Yagüe-Fernández M, Navarro P, Grazioso TP, Sevillano-Fernández E, et al. Proteomic profiles of peritoneal fluid-derived small extracellular vesicles correlate with patient outcome in ovarian cancer. J Clin Invest. 2024;134(10):e176161. doi: 10.1172/JCI176161 38564289 PMC11093605

[22] Hirschberg Y, Valle-Tamayo N, Dols-Icardo O, Engelborghs S, Buelens B, Vandenbroucke RE, et al. Proteomic comparison between non-purified cerebrospinal fluid and cerebrospinal fluid-derived extracellular vesicles from patients with Alzheimer’s, Parkinson’s and Lewy body dementia. J Extracell Vesicles. 2023;12(12):e12383. doi: 10.1002/jev2.12383 38082559 PMC10714029

[23] Chatterjee M, Özdemir S, Kunadt M, Koel-Simmelink M, Boiten W, Piepkorn L, et al. C1q is increased in cerebrospinal fluid-derived extracellular vesicles in Alzheimer’s disease: A multi-cohort proteomics and immuno-assay validation study. Alzheimers Dement. 2023;19(11):4828–40. doi: 10.1002/alz.13066 37023079

[24] Bravo-Miana RDC, Arizaga-Echebarria JK, Sabas-Ortega V, Crespillo-Velasco H, Prada A, Castillo-Triviño T, et al. Tetraspanins, GLAST and L1CAM Quantification in Single Extracellular Vesicles from Cerebrospinal Fluid and Serum of People with Multiple Sclerosis. Biomedicines. 2024;12(10):2245. doi: 10.3390/biomedicines12102245 39457558 PMC11504864

[25] Reetz L, Ghanam J, Chetty VK, Barthel L, Tippelt S, Fleischhack G, et al. Cerebrospinal Fluid-Derived Small Extracellular Vesicles May Better Reflect Medulloblastoma Proteomes than Those from Blood Plasma. Int J Mol Sci. 2025;26(19):9279. doi: 10.3390/ijms26199279 41096549 PMC12524324

[26] Chen J, Jiao Z, Mo J, Huang D, Li Z, Zhang W, et al. Comparison of the Variability of Small Extracellular Vesicles Derived from Human Liver Cancer Tissues and Cultured from the Tissue Explants Based on a Simple Enrichment Method. Stem Cell Rev Rep. 2022;18(3):1067–77. doi: 10.1007/s12015-021-10264-1 34550537 PMC8942897

[27] Wang X, Chen J, Li Z, Huang D, Yi X, Wu J, et al. An Enrichment Method for Small Extracellular Vesicles Derived from Liver Cancer Tissue. J Vis Exp. 2023;192. doi: 10.3791/64499 36806033

[28] Bray F, Laversanne M, Sung H, Ferlay J, Siegel RL, Soerjomataram I, et al. Global cancer statistics 2022: GLOBOCAN estimates of incidence and mortality worldwide for 36 cancers in 185 countries. CA Cancer J Clin. 2024;74(3):229–63. doi: 10.3322/caac.21834 38572751

[29] Chan SL, Sun H-C, Xu Y, Zeng H, El-Serag HB, Lee JM, et al. The Lancet Commission on addressing the global hepatocellular carcinoma burden: comprehensive strategies from prevention to treatment. Lancet. 2025;406(10504):731–78. doi: 10.1016/S0140-6736(25)01042-6 40744051

[30] Bi X, Zhao H, Zhao H, Li G, Wang X, Chen B, et al. Consensus of Chinese Experts on Neoadjuvant and Conversion Therapies for Hepatocellular Carcinoma: 2023 Update. Liver Cancer. 2024;14(2):223–38. doi: 10.1159/000541249 40255878 PMC12005702

[31] Papaconstantinou D, Tsilimigras DI, Pawlik TM. Recurrent Hepatocellular Carcinoma: Patterns, Detection, Staging and Treatment. J Hepatocell Carcinoma. 2022;9:947–57. doi: 10.2147/JHC.S342266 36090786 PMC9450909

[32] Qiu X, Campos Y, van de Vlekkert D, Gomero E, Tanwar AC, Kalathur R, et al. Distinct functions of dimeric and monomeric scaffold protein Alix in regulating F-actin assembly and loading of exosomal cargo. J Biol Chem. 2022;298(10):102425. doi: 10.1016/j.jbc.2022.102425 36030822 PMC9531180

[33] Chanteloup G, Cordonnier M, Isambert N, Bertaut A, Hervieu A, Hennequin A, et al. Monitoring HSP70 exosomes in cancer patients’ follow up: a clinical prospective pilot study. J Extracell Vesicles. 2020;9(1):1766192. doi: 10.1080/20013078.2020.1766192 32595915 PMC7301715

[34] Alvarez-Rodriguez M, Ntzouni M, Wright D, Khan KI, López-Béjar M, Martinez CA, et al. Chicken seminal fluid lacks CD9- and CD44-bearing extracellular vesicles. Reprod Domest Anim. 2020;55(3):293–300. doi: 10.1111/rda.13617 31894881

[35] Su D, Zhang Z, Xu Z, Xia F, Yan Y. A prognostic exosome-related LncRNA risk model correlates with the immune microenvironment in liver cancer. Front Genet. 2022;13:965329. doi: 10.3389/fgene.2022.965329 36081999 PMC9445491

[36] Ye G-Q, Wang M-D, Diao Y-K, Li C, Yao L-Q, Gu L-H, et al. Deciphering the Role of Necroptosis-Related Long Non-coding RNAs in Hepatocellular Carcinoma: A Necroptosis-Related lncRNA-Based Signature to Predict the Prognosis of Hepatocellular Carcinoma. Appl Biochem Biotechnol. 2025;197(1):313–34. doi: 10.1007/s12010-024-05014-1 39115788

[37] Kudo M, Kang Y-K, Park J-W, Qin S, Inaba Y, Assenat E, et al. Regional Differences in Efficacy, Safety, and Biomarkers for Second-Line Axitinib in Patients with Advanced Hepatocellular Carcinoma: From a Randomized Phase II Study. Liver Cancer. 2018;7(2):148–64. doi: 10.1159/000484620 29888205 PMC5985413

[38] Cao W, Ren Y, Liu Y, Cao G, Chen Z, Wang F. KDM4A-AS1 Promotes Cell Proliferation, Migration, and Invasion via the miR-4306/STX6 Axis in Hepatocellular Carcinoma. Crit Rev Eukaryot Gene Expr. 2024;34(4):55–68. doi: 10.1615/CritRevEukaryotGeneExpr.2024051414 38505873

[39] Deng R, Cui X, Dong Y, Tang Y, Tao X, Wang S, et al. Construction of circRNA-Based ceRNA Network to Reveal the Role of circRNAs in the Progression and Prognosis of Hepatocellular Carcinoma. Front Genet. 2021;12:626764. doi: 10.3389/fgene.2021.626764 33719338 PMC7953168

[40] Chang L, Li C, Lan T, Wu L, Yuan Y, Liu Q, et al. Decreased expression of long non-coding RNA GAS5 indicates a poor prognosis and promotes cell proliferation and invasion in hepatocellular carcinoma by regulating vimentin. Mol Med Rep. 2016;13(2):1541–50. doi: 10.3892/mmr.2015.4716 26707238 PMC4732840

[41] Wang C, Ke S, Li M, Lin C, Liu X, Pan Q. Downregulation of LncRNA GAS5 promotes liver cancer proliferation and drug resistance by decreasing PTEN expression. Mol Genet Genomics. 2020;295(1):251–60. doi: 10.1007/s00438-019-01620-5 31705194

[42] Manganelli M, Grossi I, Ferracin M, Guerriero P, Negrini M, Ghidini M, et al. Longitudinal Circulating Levels of miR-23b-3p, miR-126-3p and lncRNA GAS5 in HCC Patients Treated with Sorafenib. Biomedicines. 2021;9(7):813. doi: 10.3390/biomedicines9070813 34356875 PMC8301380

[43] Wang Y, Yang L, Chen T, Liu X, Guo Y, Zhu Q, et al. A novel lncRNA MCM3AP-AS1 promotes the growth of hepatocellular carcinoma by targeting miR-194-5p/FOXA1 axis. Mol Cancer. 2019;18(1):28. doi: 10.1186/s12943-019-0957-7 30782188 PMC6381672

[44] Fan F, Chen K, Lu X, Li A, Liu C, Wu B. Dual targeting of PD-L1 and PD-L2 by PCED1B-AS1 via sponging hsa-miR-194-5p induces immunosuppression in hepatocellular carcinoma. Hepatol Int. 2021;15(2):444–58. doi: 10.1007/s12072-020-10101-6 33219943

[45] van der Ree MH, Jansen L, Kruize Z, van Nuenen AC, van Dort KA, Takkenberg RB, Reesink HW, Kootstra NA. Plasma MicroRNA Levels Are Associated With Hepatitis B e Antigen Status and Treatment Response in Chronic Hepatitis B Patients. J Infect Dis. 2017;215(9):1421–9. doi: 10.1093/infdis/jix140 28368488

[46] Chen Y, LijuanZhang, Ling L. miR-194-5p targets SOCS2 to predict pegIFNα treatment response in HBeAg-positive chronic hepatitis B patients. Virol J. 2026;23(1):68. doi: 10.1186/s12985-026-03090-9 41645267 PMC12973741

[47] Nielsen KO, Jacobsen KS, Mirza AH, Winther TN, Størling J, Glebe D, et al. Hepatitis B virus upregulates host microRNAs that target apoptosis-regulatory genes in an in vitro cell model. Exp Cell Res. 2018;371(1):92–103. doi: 10.1016/j.yexcr.2018.07.044 30059664

