# Supplementary figures and images for: Optimization of extracellular vesicle extraction from hepatic tissue interstitial fluid and analysis of their ncRNA expression profiles
Source: PLoS One. 2026 Aug 3;21(8):e0355303. doi: 10.1371/journal.pone.0355303 (PMC13432105; doi:10.1371/journal.pone.0355303)

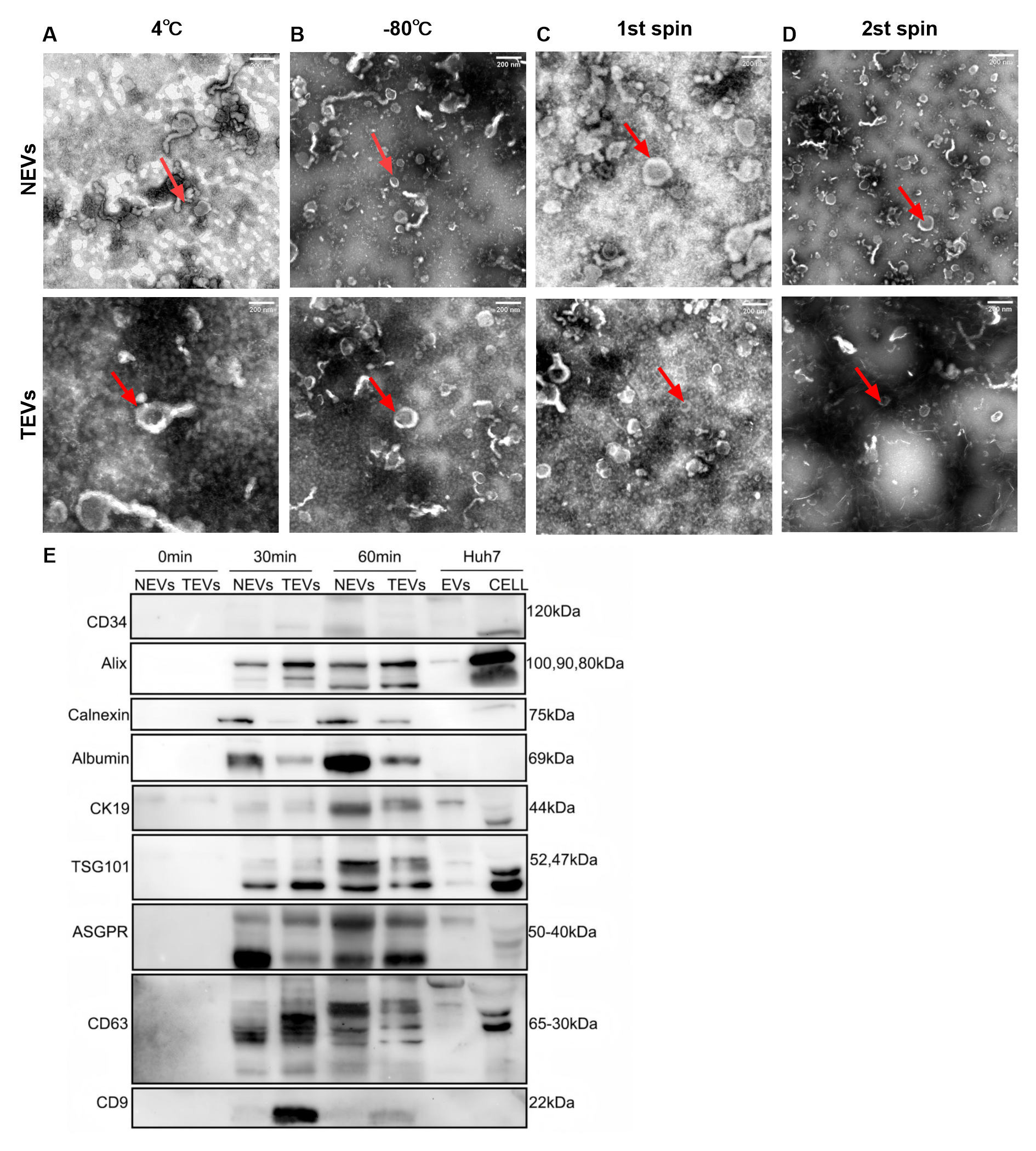

Supplement: S1 Fig — (A) Electron micrograph of TIF-EVs stored at 4°C for one week; (B) Electron micrograph of TIF-EVs stored at −80°C for one week; (C) Electron micrograph of TIF-EVs obtained after a single differential centrifugation step; (D) Electron micrograph of TIF-EVs obtained after repeating the differential centrifugation step once; (E) Western blot analysis of TIF-EVs digested with proteinase for 0, 30, and 60 min. NEVs (non-tumor adjacent tissue-derived TIF-sEVs) and TEVs (tumor tissue-derived TIF-sEVs) were analyzed, with Huh7-derived EVs serving as positive control and Huh7 cell lysate as negative control. (TIF) [file pone.0355303.s001.tif]
