# Supplementary material for: Optimization of extracellular vesicle extraction from hepatic tissue interstitial fluid and analysis of their ncRNA expression profiles
Source: PLoS One. 2026 Aug 3;21(8):e0355303. doi: 10.1371/journal.pone.0355303 (PMC13432105; doi:10.1371/journal.pone.0355303)
Supplement: S1 File — (PDF) [file pone.0355303.s002.pdf]

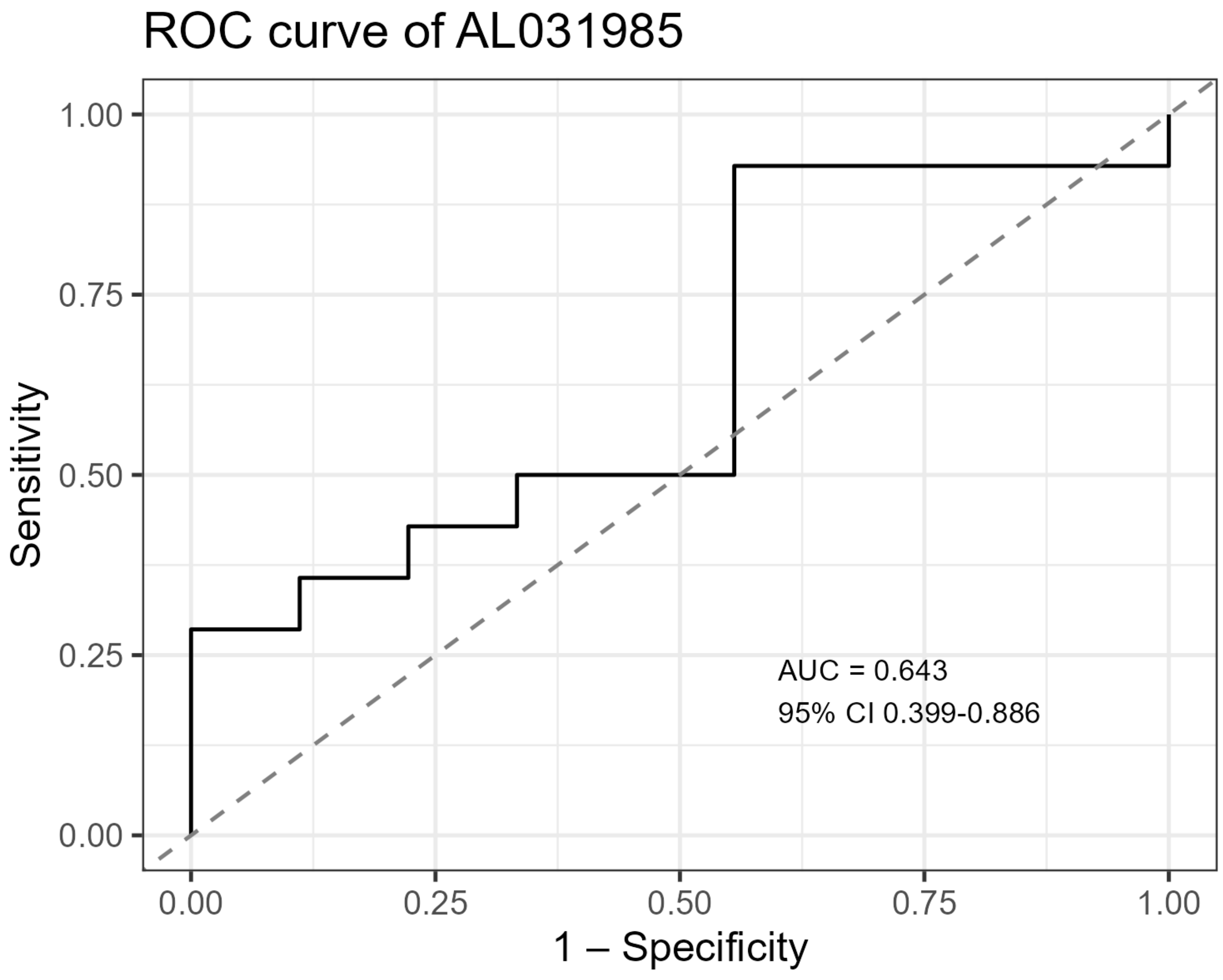

ROC curve of AL031985
Sensitivity
1.00
0.75
0.50
0.25
0.00
AUC = 0.643
95% CI 0.399-0.886
0.00
0.25
0.50
0.75
1.00
1 – Specificity

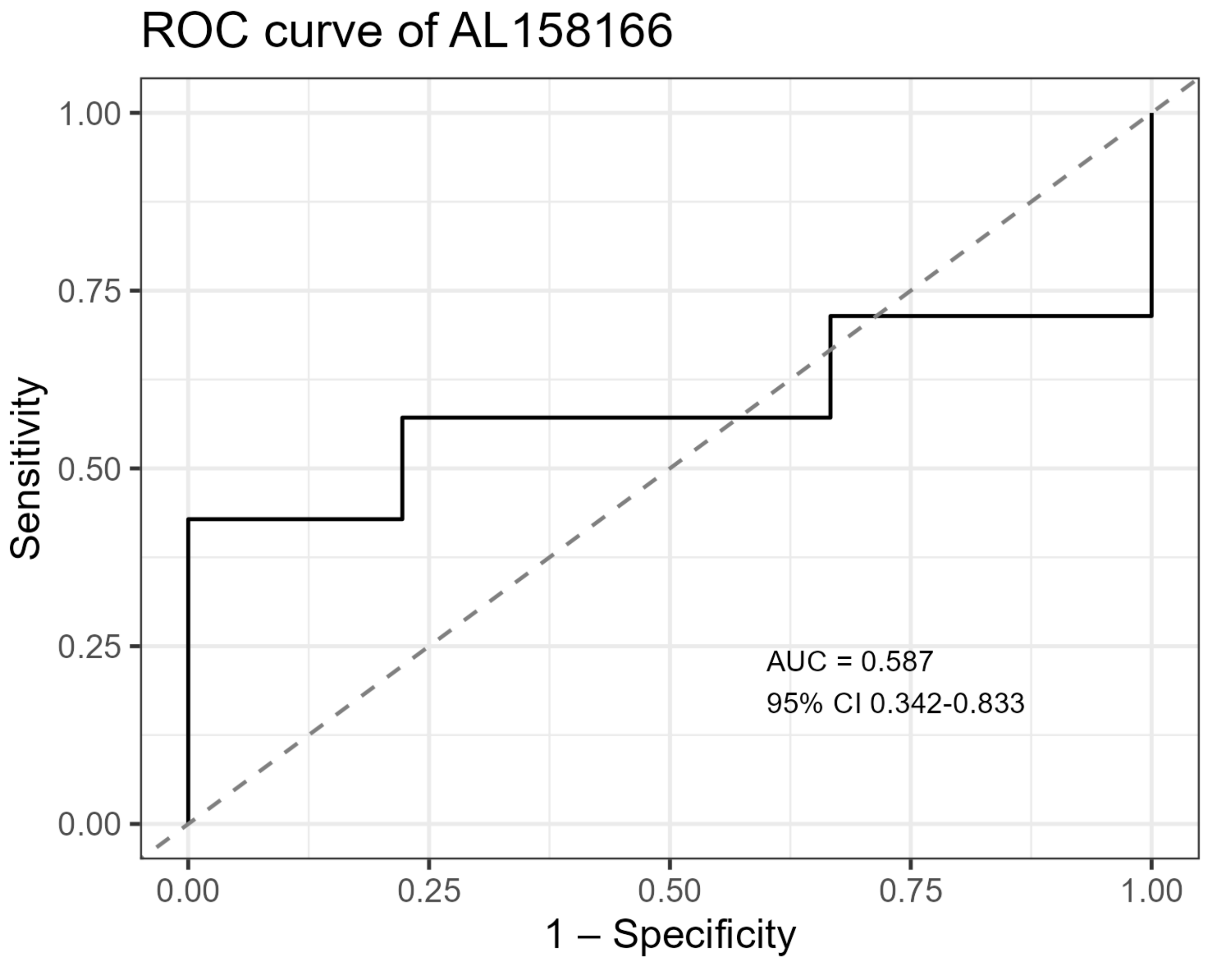

ROC curve of AL158166
Sensitivity
1.00
0.75
0.50
0.25
0.00
0.00
0.25
0.50
0.75
1.00
1 – Specificity
AUC = 0.587
95% CI 0.342-0.833

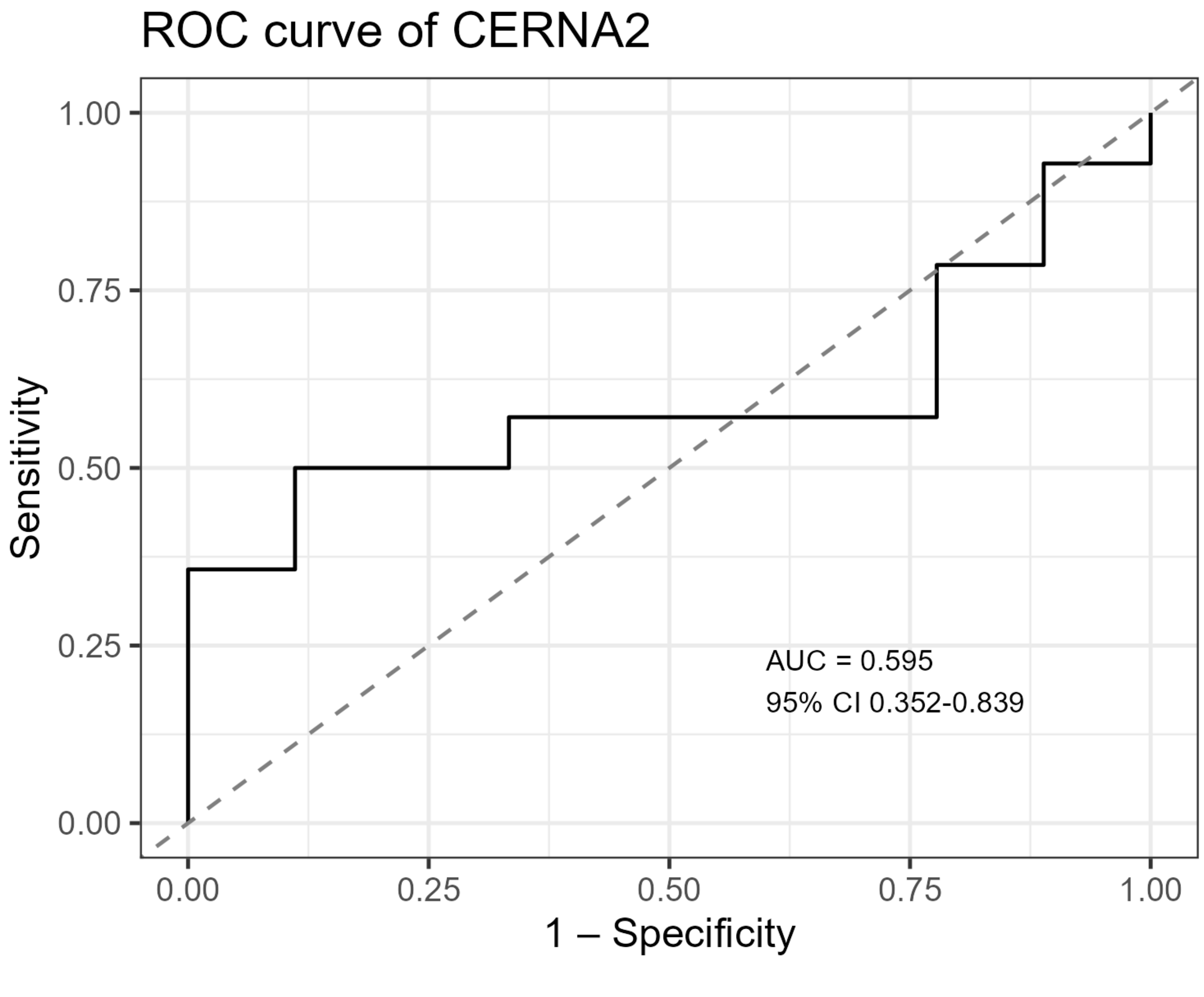
ROC curve of CERNA2
Sensitivity
1.00
0.75
0.50
0.25
0.00
AUC = 0.595
95% CI 0.352-0.839
0.00
0.25
0.50
0.75
1.00
1 – Specificity

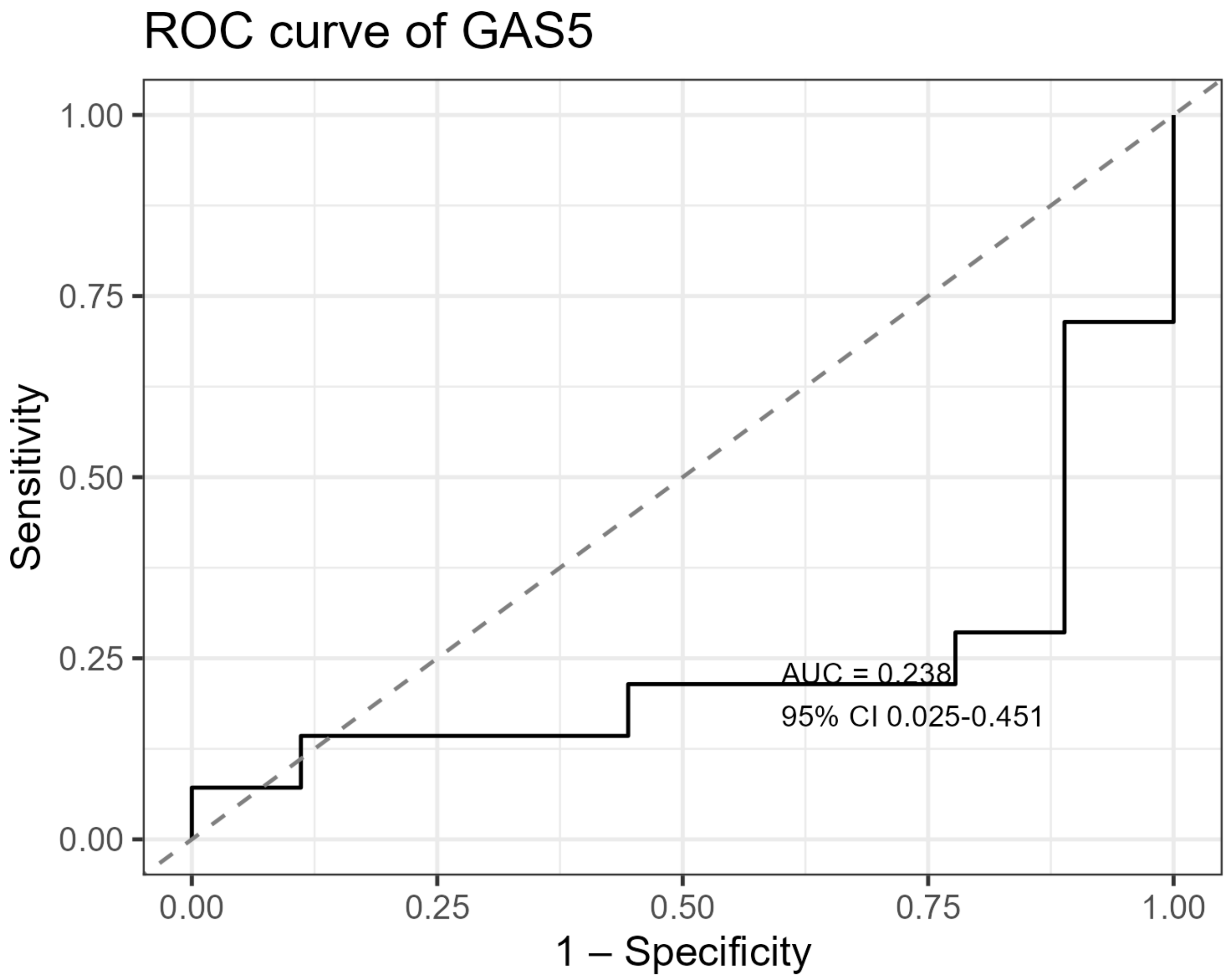

ROC curve of GAS5
Sensitivity
1.00
0.75
0.50
0.25
0.00
0.00
0.25
0.50
0.75
1.00
1 – Specificity
AUC = 0.238
95% CI 0.025-0.451

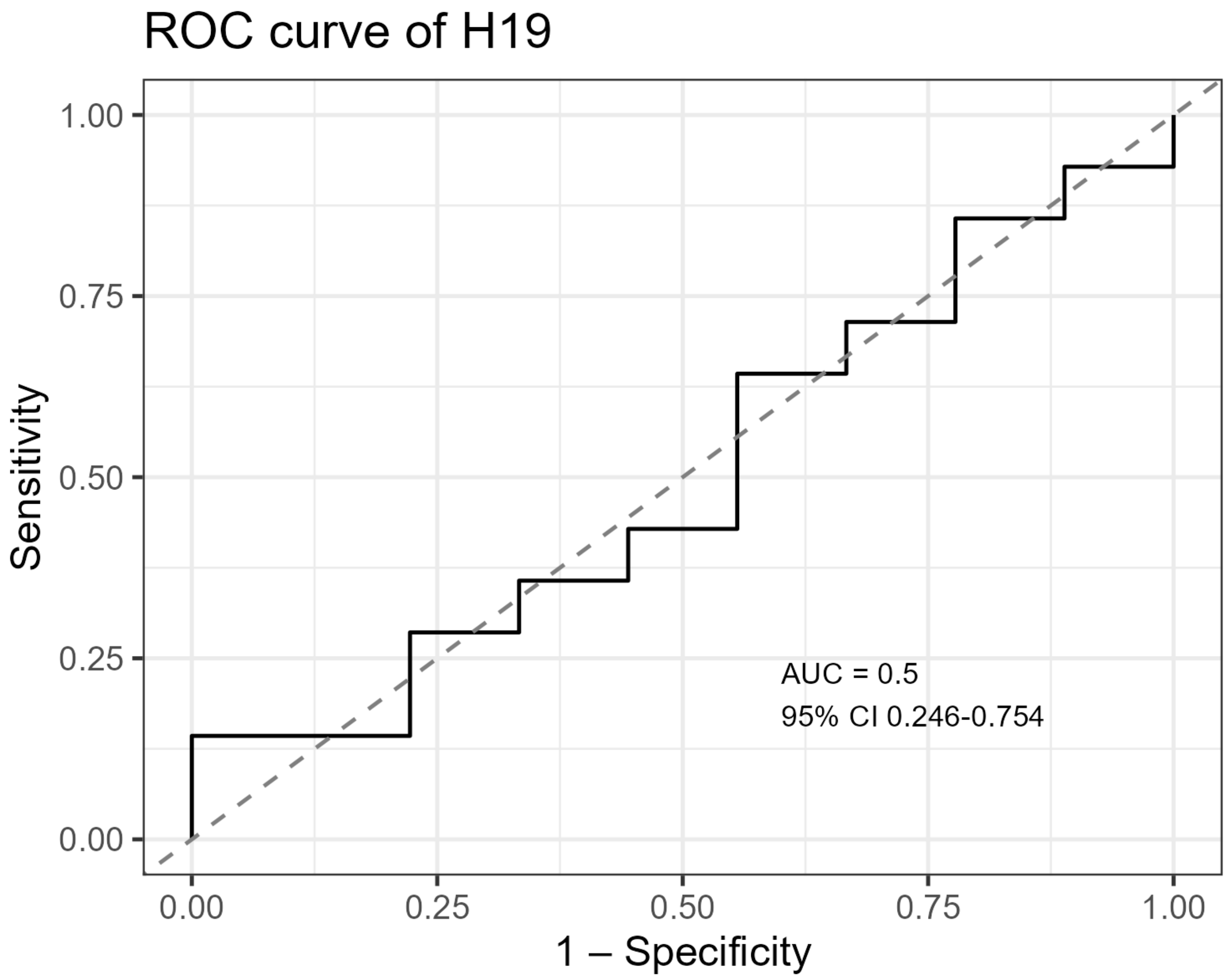
ROC curve of H19
Sensitivity
1.00
0.75
0.50
0.25
0.00
0.00
0.25
0.50
0.75
1.00
1 – Specificity
AUC = 0.5
95% CI 0.246-0.754

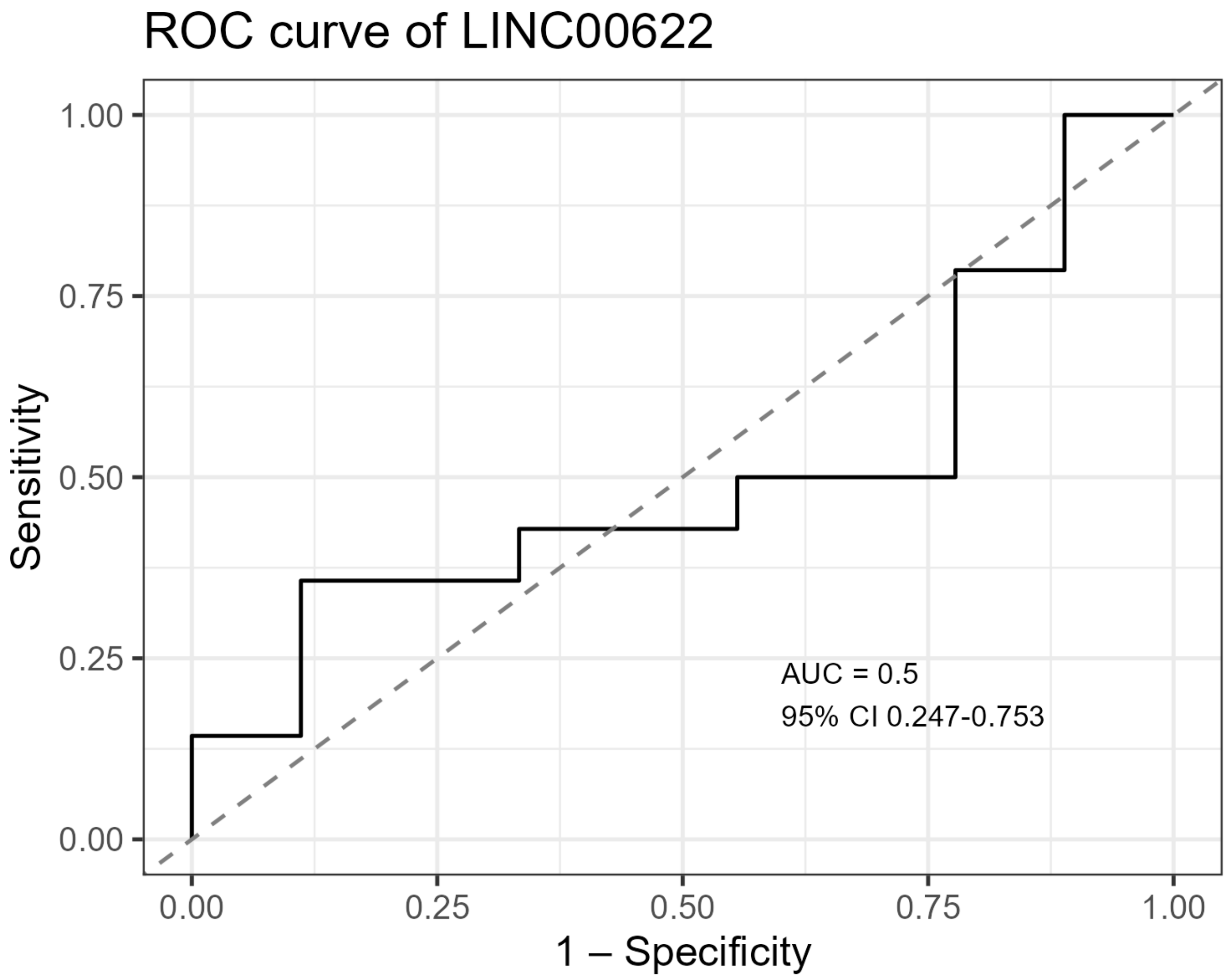
ROC curve of LINC00622
Sensitivity
1.00
0.75
0.50
0.25
0.00
0.00
0.25
0.50
0.75
1.00
1 – Specificity
AUC = 0.5
95% CI 0.247-0.753

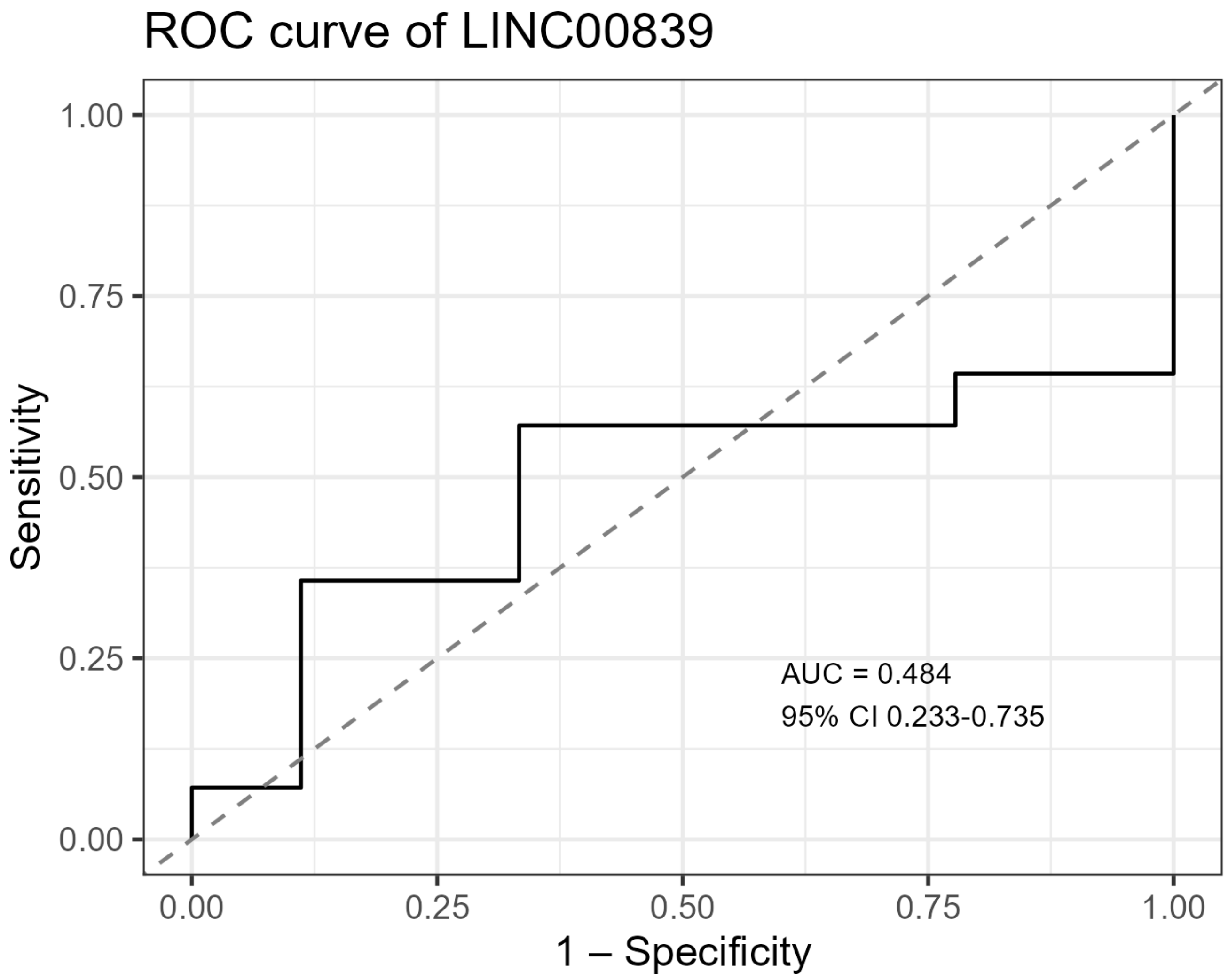

ROC curve of LINC00839
Sensitivity
1 – Specificity
1.00
0.75
0.50
0.25
0.00
0.00
0.25
0.50
0.75
1.00
AUC = 0.484
95% CI 0.233-0.735

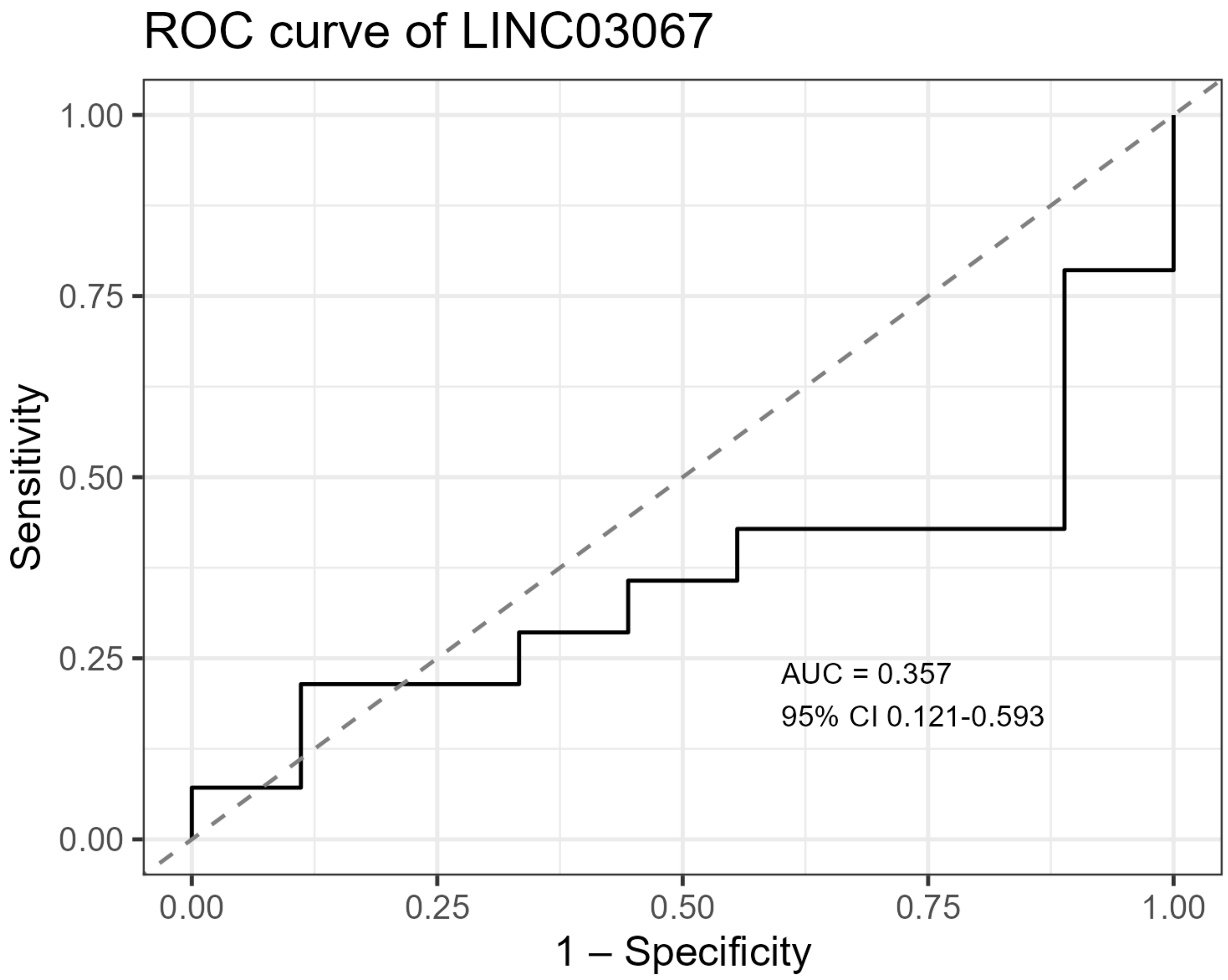
ROC curve of LINC03067
Sensitivity
1.00
0.75
0.50
0.25
0.00
0.00
0.25
0.50
0.75
1.00
1 – Specificity
AUC = 0.357
95% CI 0.121-0.593

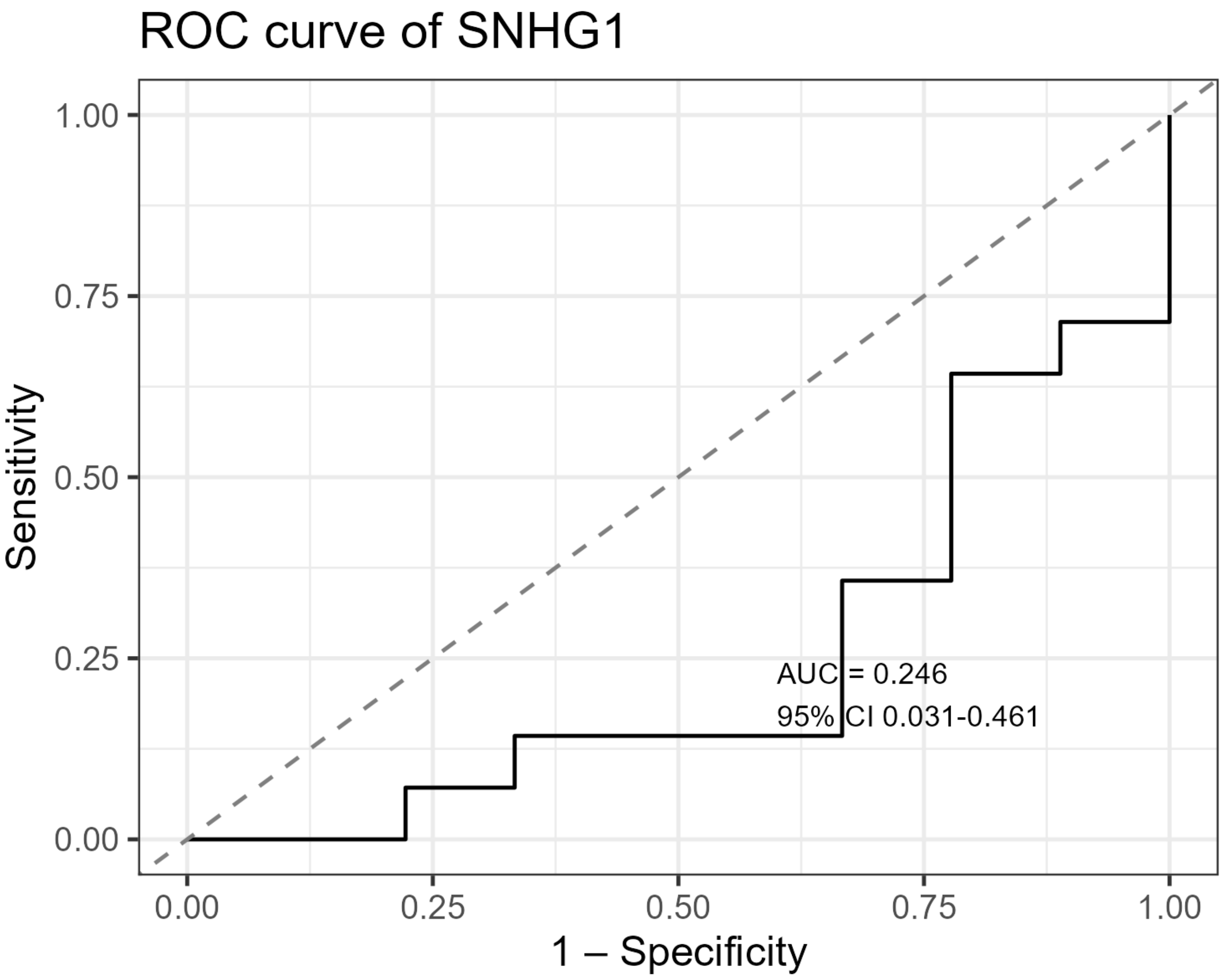

ROC curve of SNHG1
1.00
0.75
0.50
0.25
0.00
Sensitivity
AUC = 0.246
95% CI 0.031-0.461
0.00
0.25
0.50
0.75
1.00
1 – Specificity

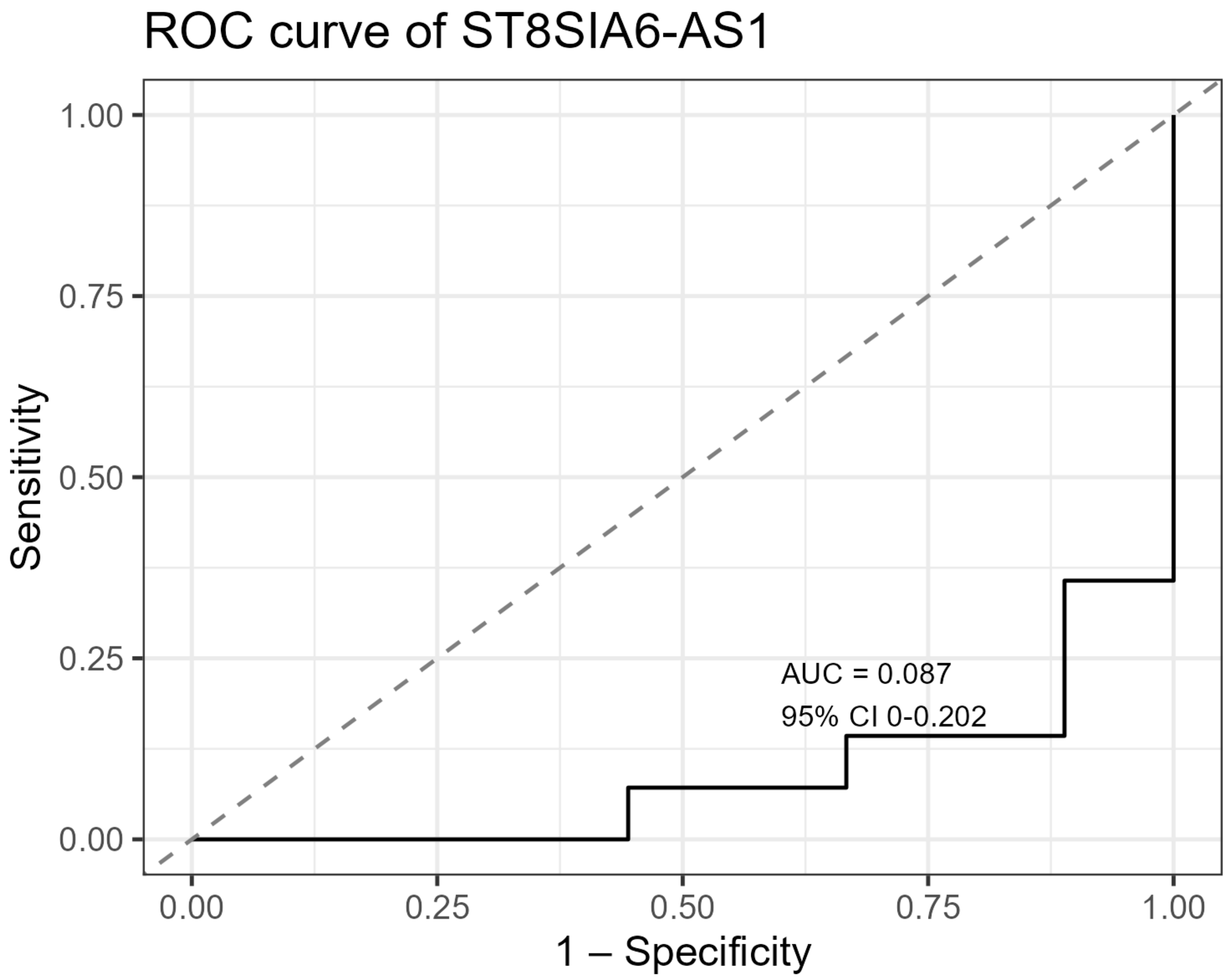
ROC curve of ST8SIA6-AS1
1.00
0.75
0.50
0.25
0.00
Sensitivity
AUC = 0.087
95% CI 0-0.202
0.00
0.25
0.50
0.75
1.00
1 – Specificity

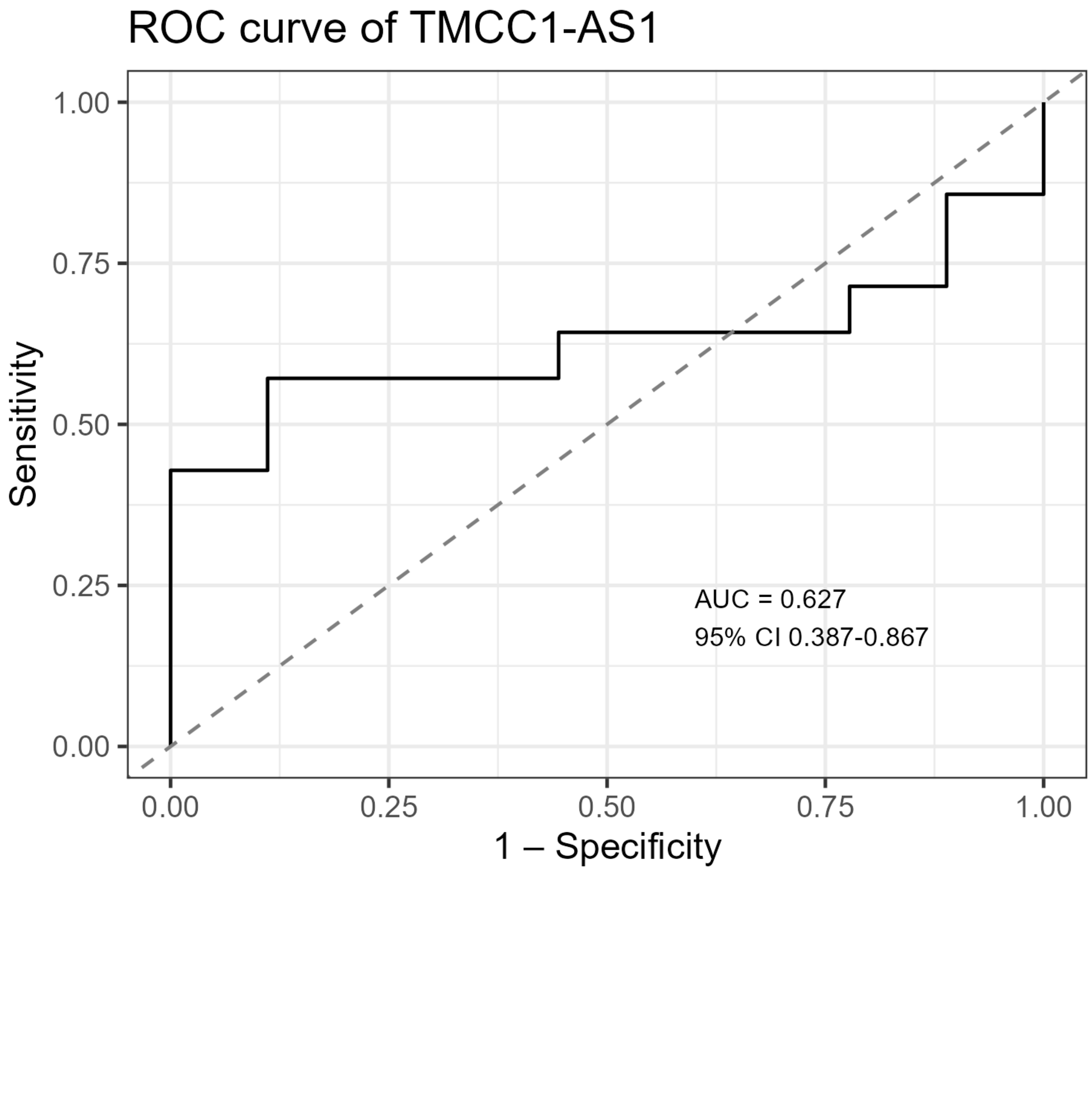
ROC curve of TMCC1-AS1
Sensitivity
1.00
0.75
0.50
0.25
0.00
0.00
0.25
0.50
0.75
1.00
1 – Specificity
AUC = 0.627
95% CI 0.387-0.867

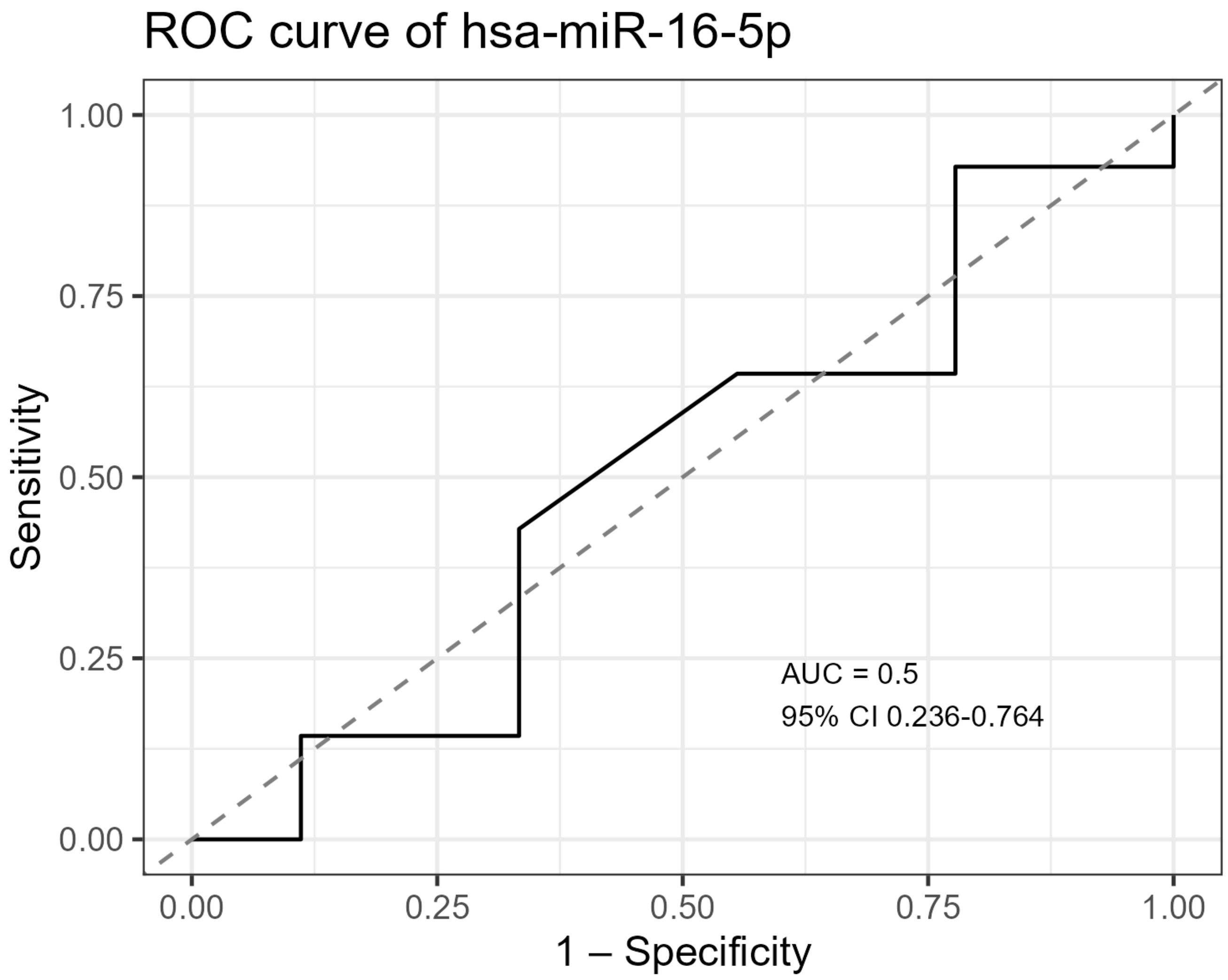
ROC curve of hsa-miR-16-5p
Sensitivity
1.00
0.75
0.50
0.25
0.00
0.00
0.25
0.50
0.75
1.00
1 – Specificity
AUC = 0.5
95% CI 0.236-0.764

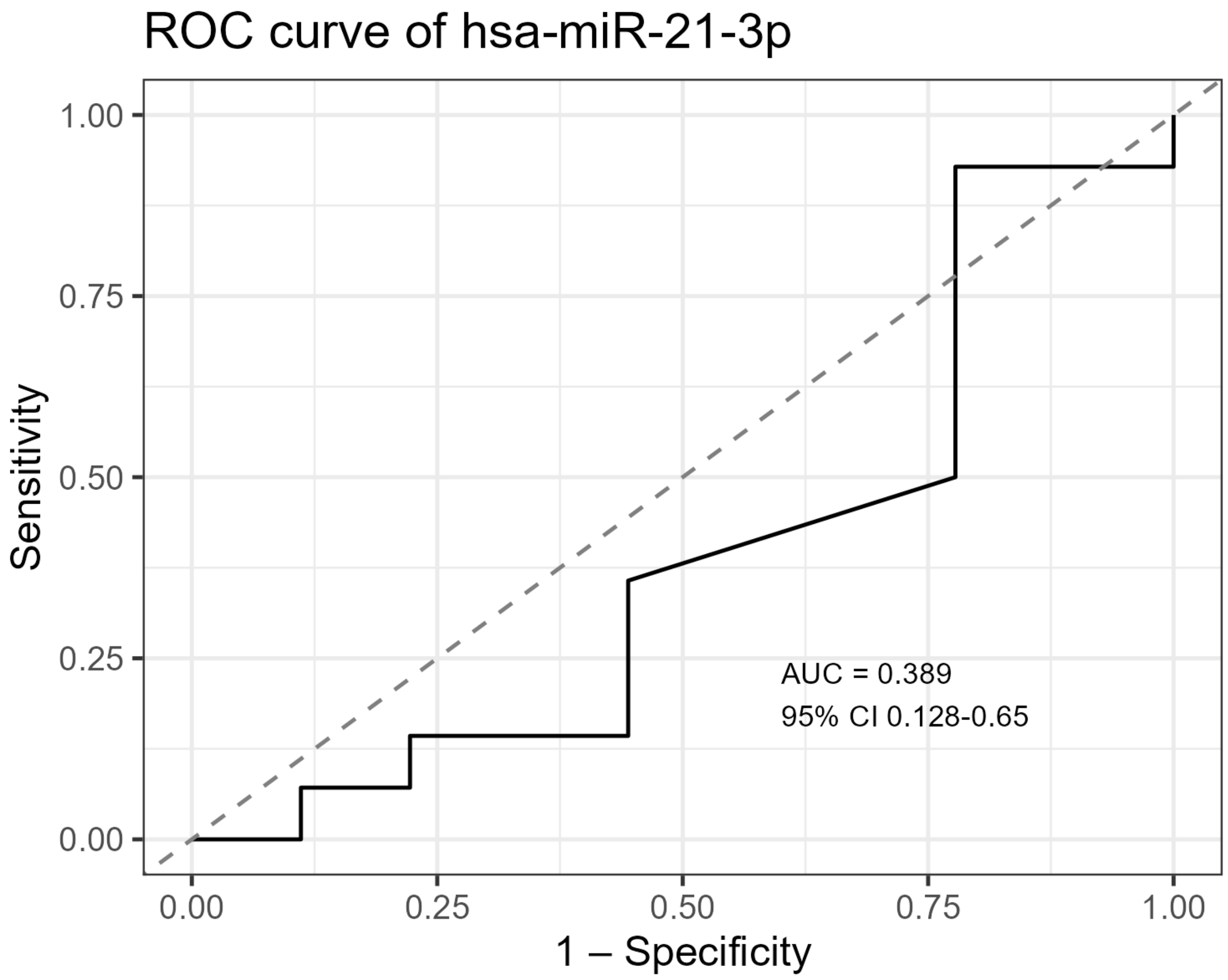

ROC curve of hsa-miR-21-3p
Sensitivity
1.00
0.75
0.50
0.25
0.00
0.00
0.25
0.50
0.75
1.00
1 – Specificity
AUC = 0.389
95% CI 0.128-0.65

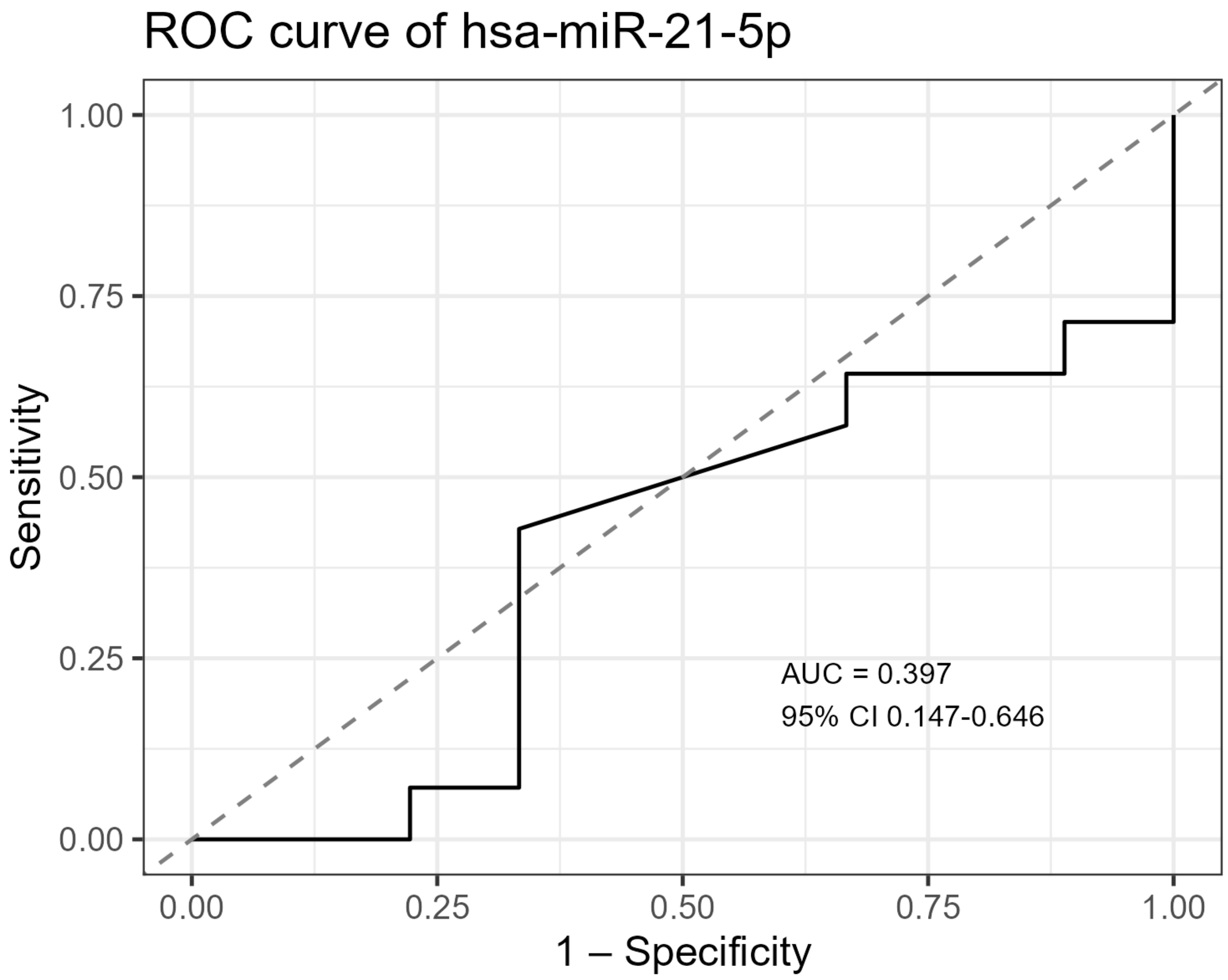
ROC curve of hsa-miR-21-5p
1.00
0.75
0.50
0.25
0.00
Sensitivity
AUC = 0.397
95% CI 0.147-0.646
0.00
0.25
0.50
0.75
1.00
1 – Specificity

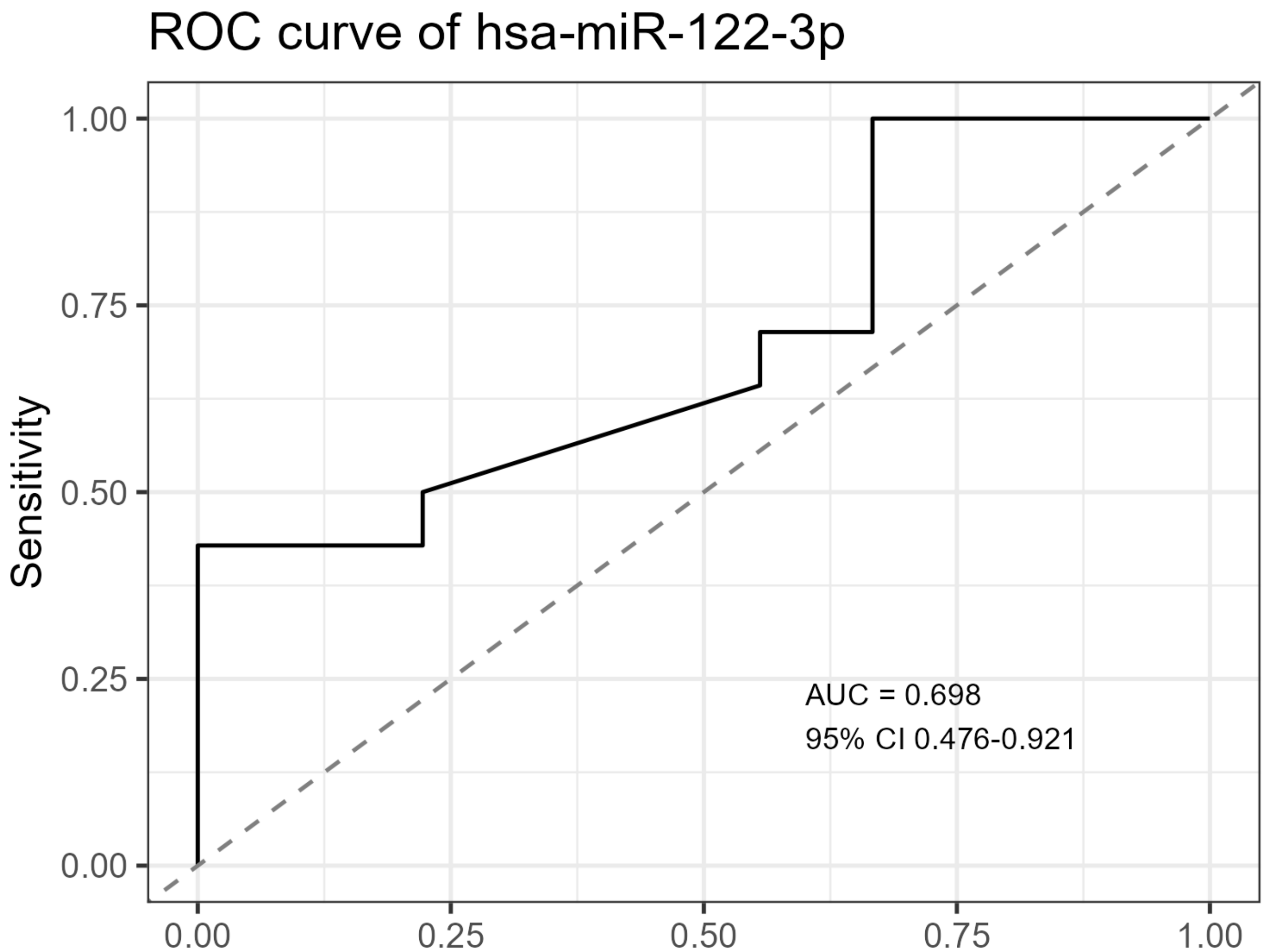
ROC curve of hsa-miR-122-3p
Sensitivity
1.00
0.75
0.50
0.25
0.00
0.00
0.25
0.50
0.75
1.00
1 – Specificity
AUC = 0.698
95% CI 0.476-0.921

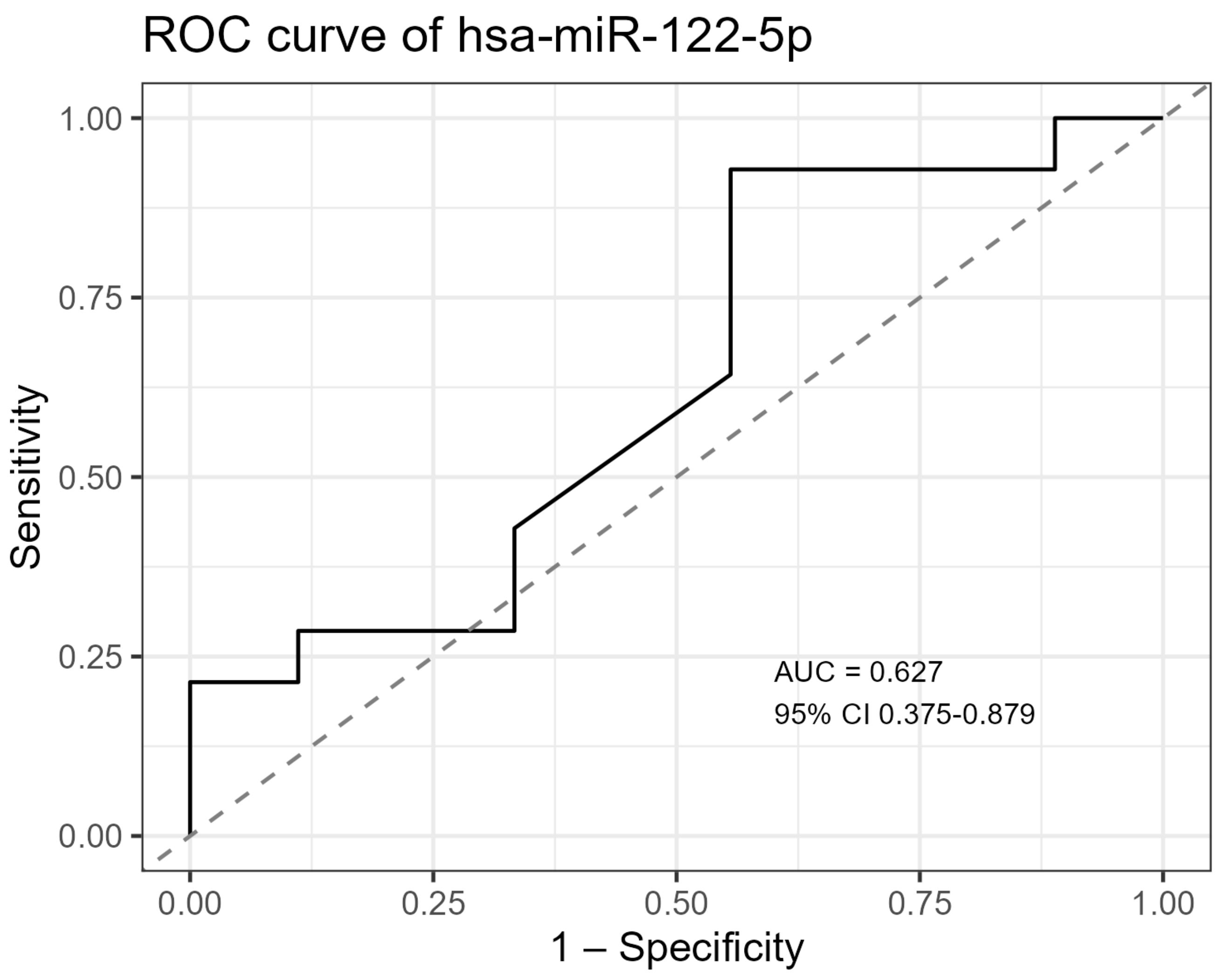

ROC curve of hsa-miR-122-5p
Sensitivity
1 – Specificity
1.00
0.75
0.50
0.25
0.00
0.00
0.25
0.50
0.75
1.00
AUC = 0.627
95% CI 0.375-0.879

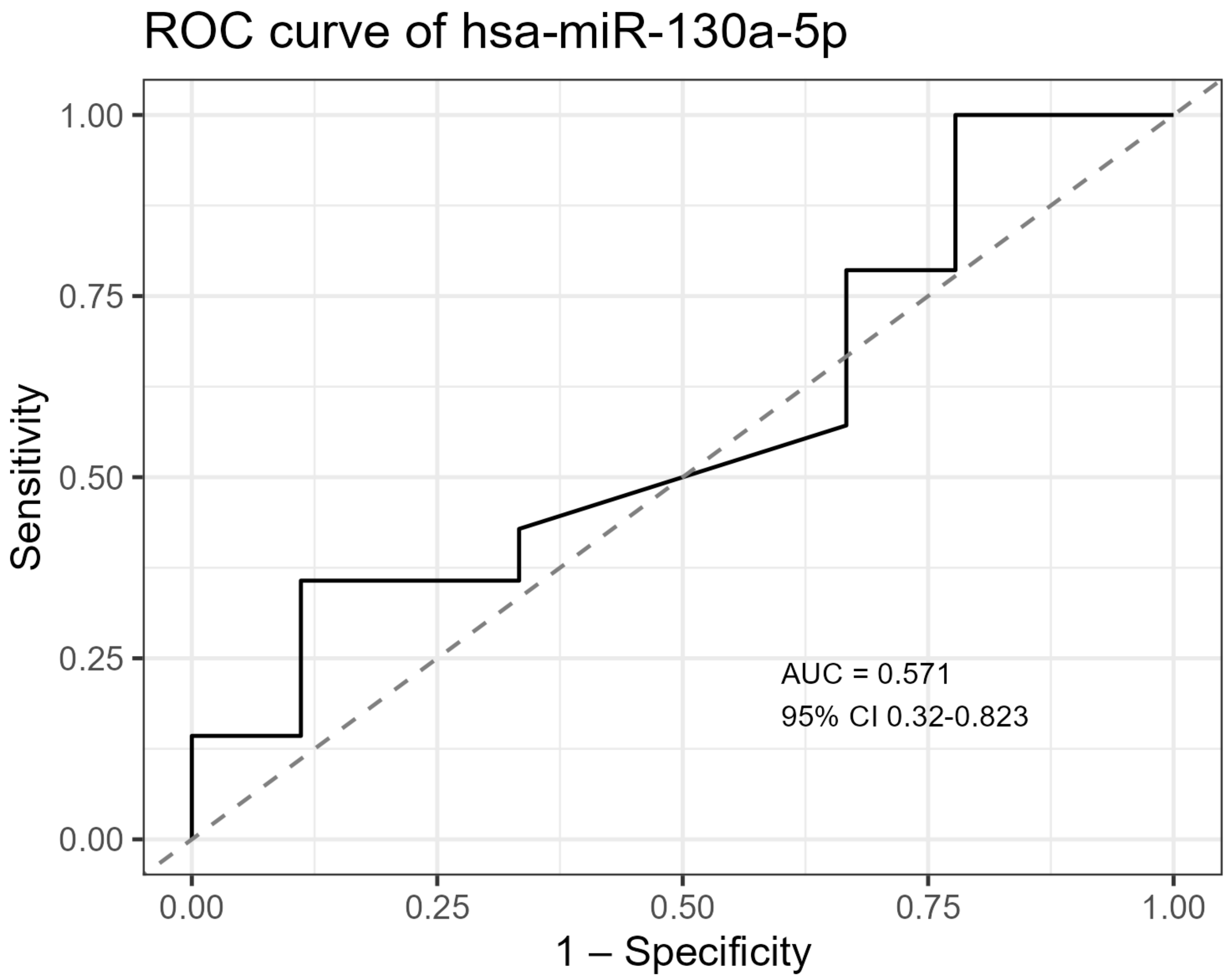
ROC curve of hsa-miR-130a-5p
Sensitivity
1.00
0.75
0.50
0.25
0.00
0.00
0.25
0.50
0.75
1.00
1 – Specificity
AUC = 0.571
95% CI 0.32-0.823

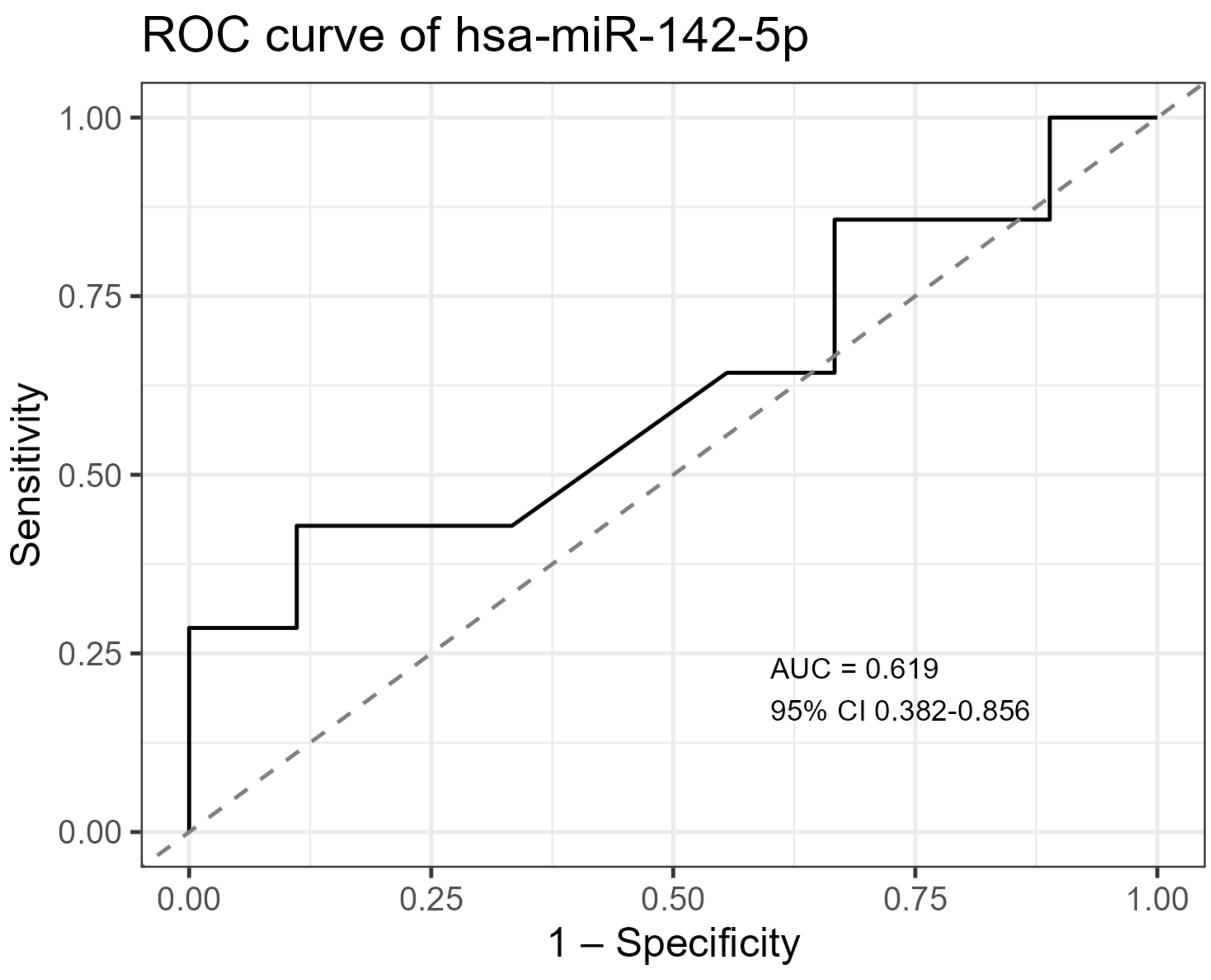
ROC curve of hsa-miR-142-5p
Sensitivity
1.00
0.75
0.50
0.25
0.00
0.00
0.25
0.50
0.75
1.00
1 – Specificity
AUC = 0.619
95% CI 0.382-0.856

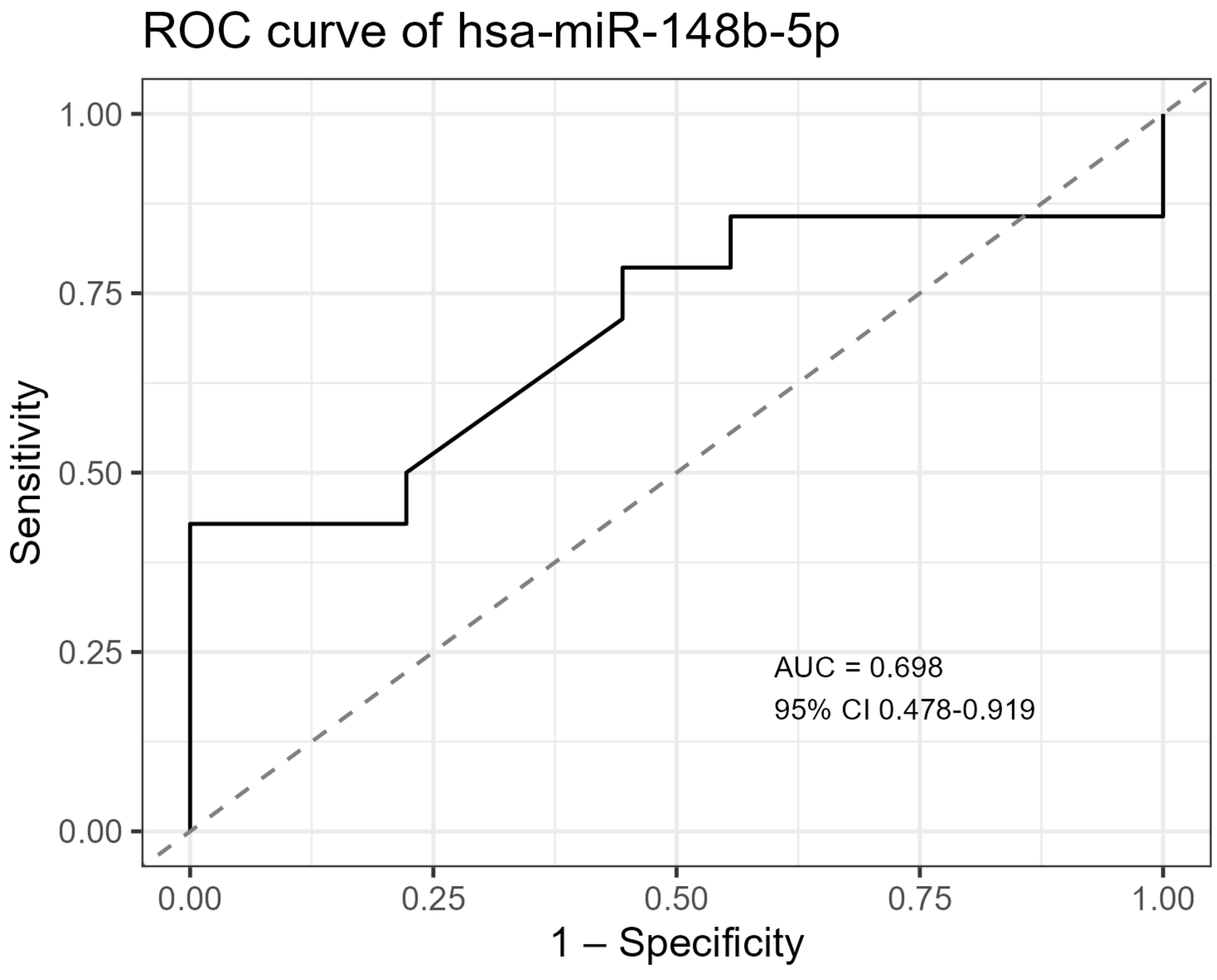
ROC curve of hsa-miR-148b-5p
1.00
0.75
0.50
0.25
0.00
Sensitivity
0.00
0.25
0.50
0.75
1.00
1 – Specificity
AUC = 0.698
95% CI 0.478-0.919

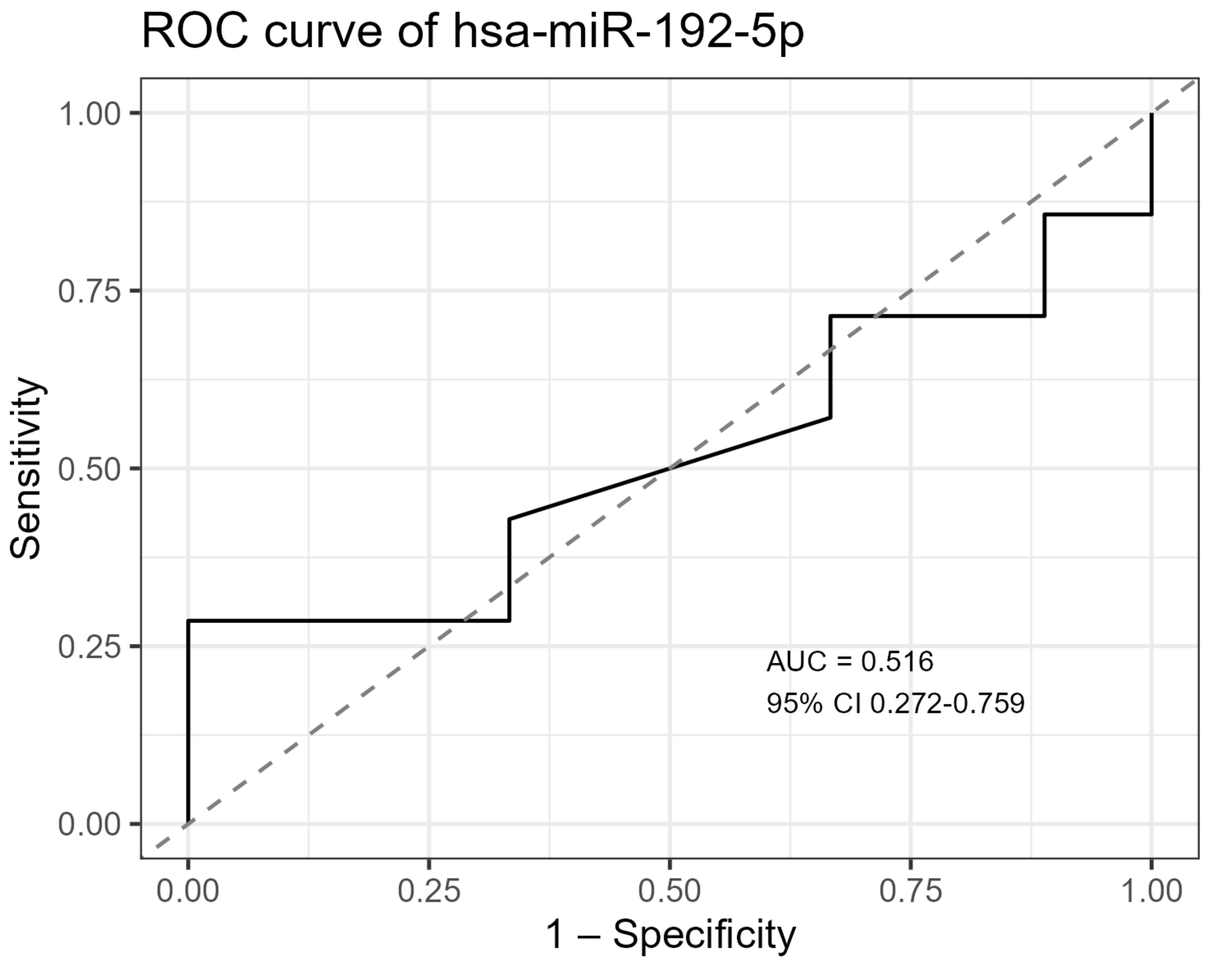
ROC curve of hsa-miR-192-5p
Sensitivity
1.00
0.75
0.50
0.25
0.00
AUC = 0.516
95% CI 0.272-0.759
0.00
0.25
0.50
0.75
1.00
1 – Specificity

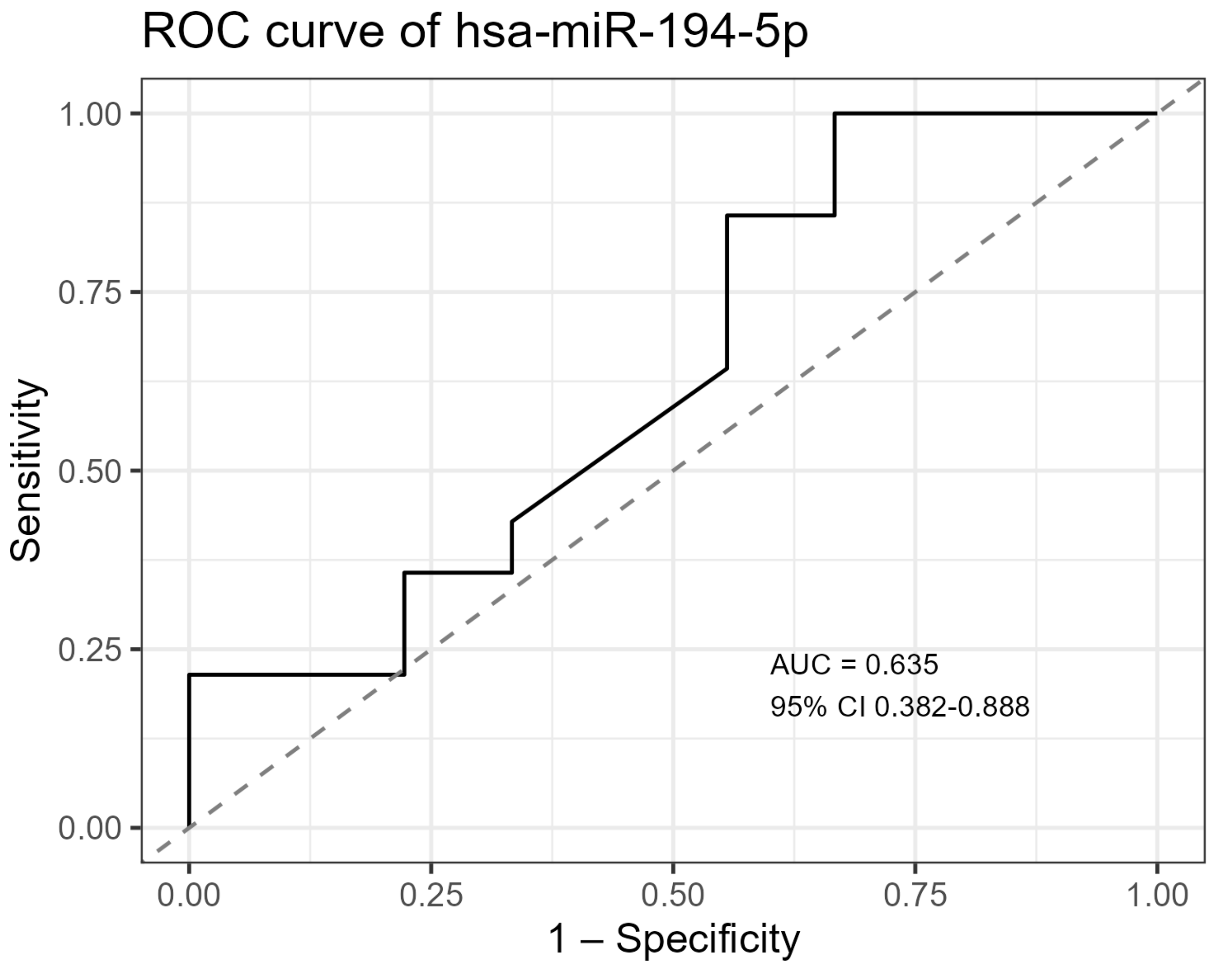
ROC curve of hsa-miR-194-5p
1.00
0.75
0.50
0.25
0.00
Sensitivity
AUC = 0.635
95% CI 0.382-0.888
0.00
0.25
0.50
0.75
1.00
1 – Specificity

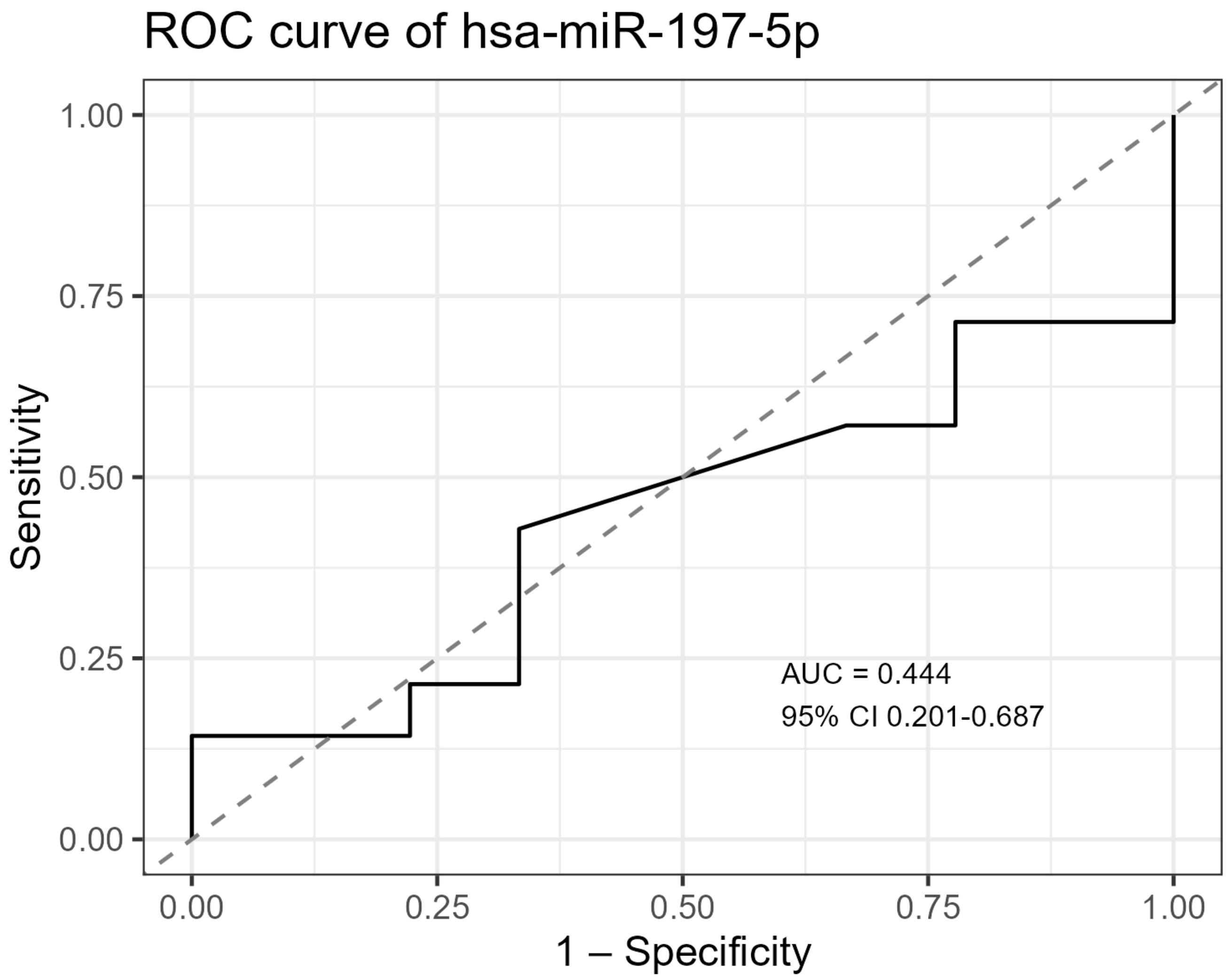
ROC curve of hsa-miR-197-5p
Sensitivity
1.00
0.75
0.50
0.25
0.00
0.00
0.25
0.50
0.75
1.00
1 – Specificity
AUC = 0.444
95% CI 0.201-0.687

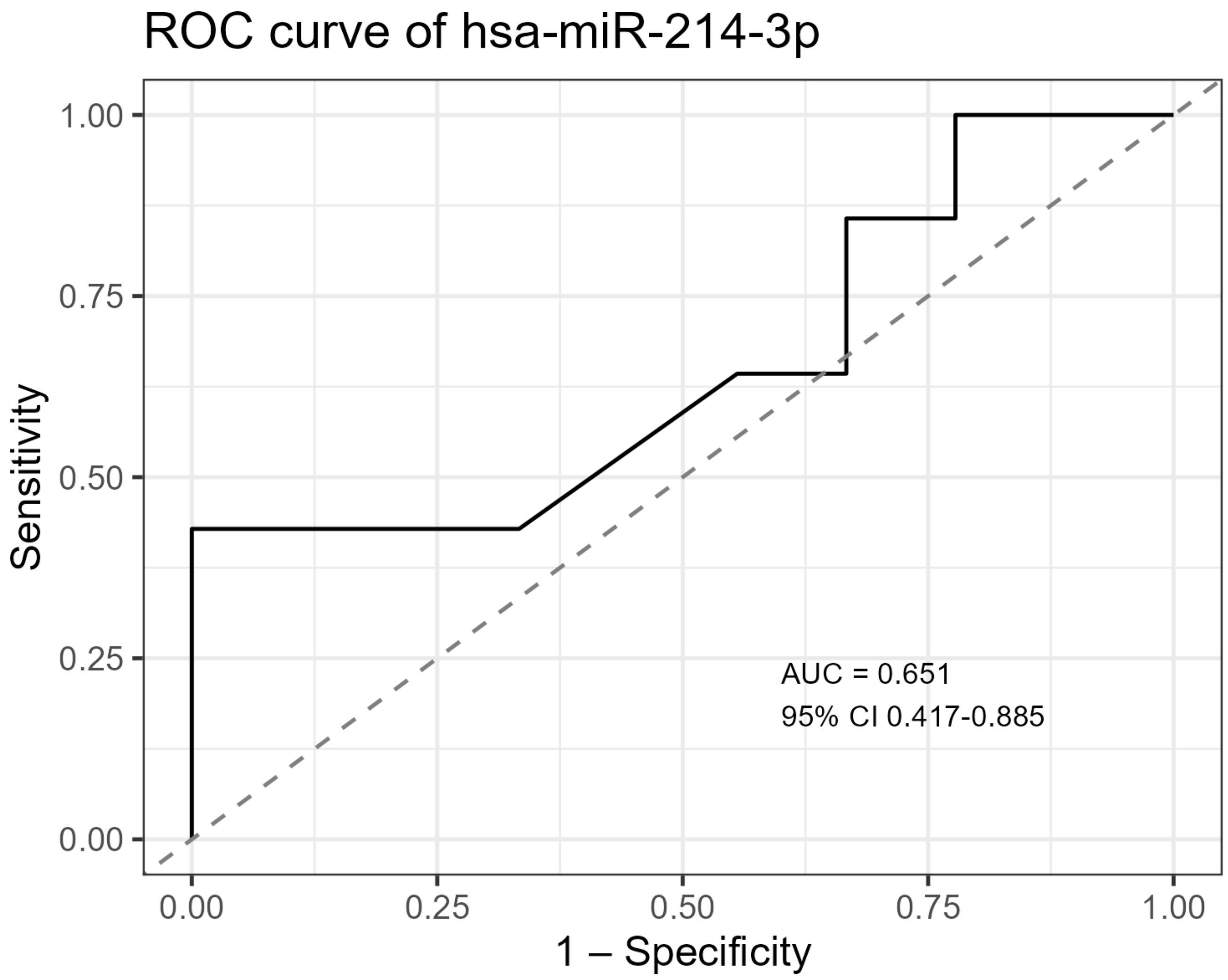

ROC curve of hsa-miR-214-3p
Sensitivity
1.00
0.75
0.50
0.25
0.00
0.00
0.25
0.50
0.75
1.00
1 – Specificity
AUC = 0.651
95% CI 0.417-0.885

ROC curve of hsa-miR-451a

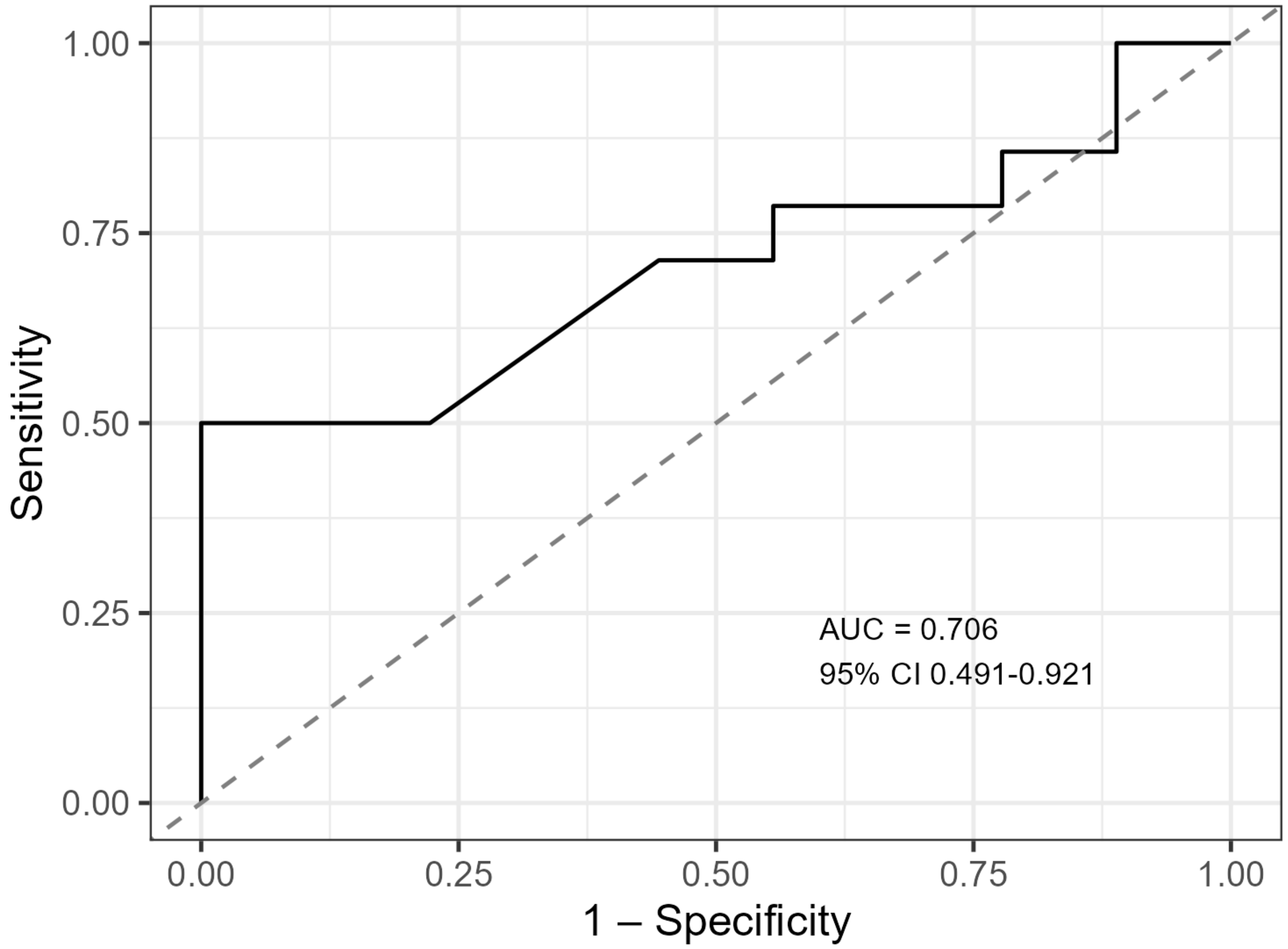

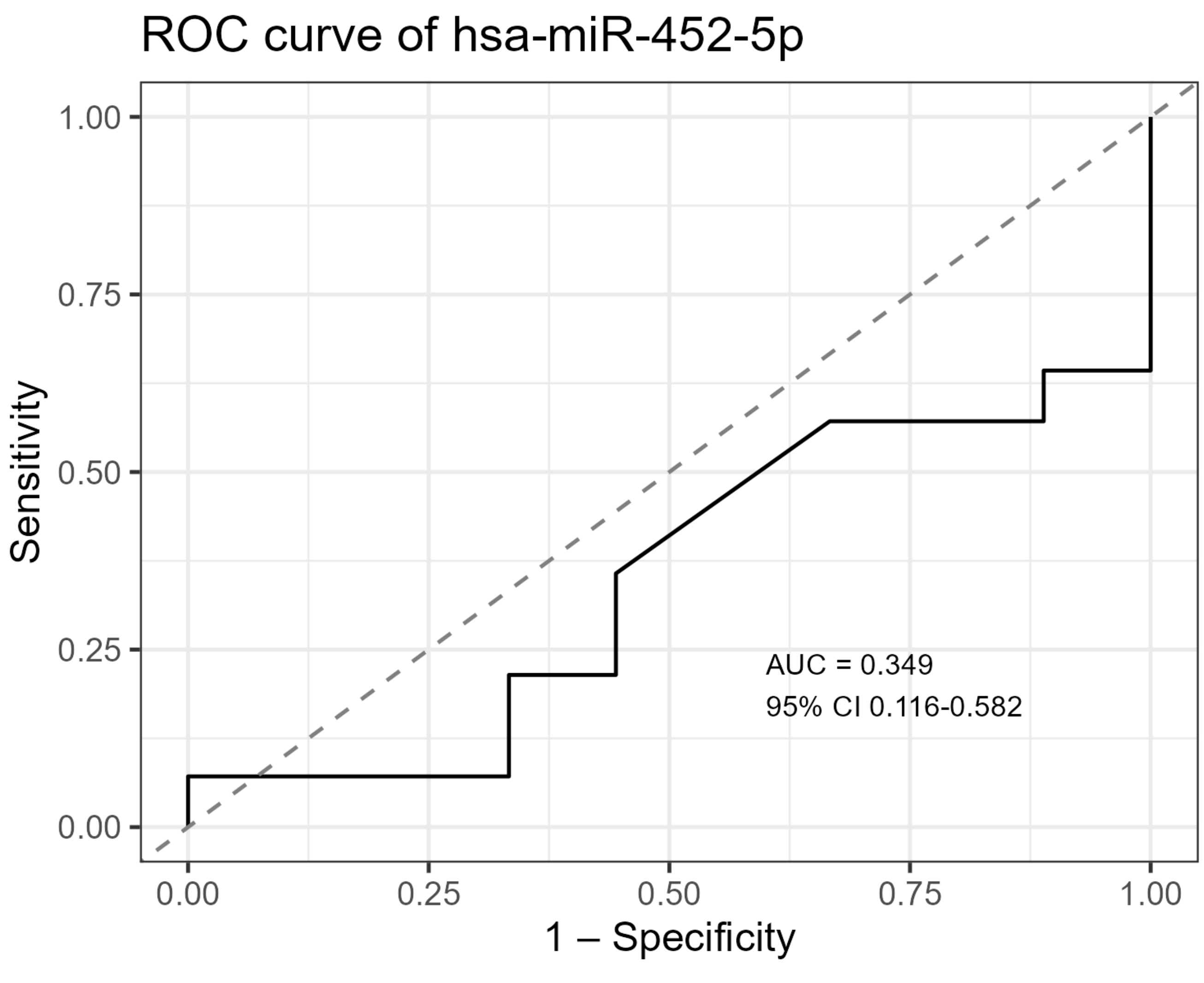
ROC curve of hsa-miR-452-5p
Sensitivity
1.00
0.75
0.50
0.25
0.00
0.00
0.25
0.50
0.75
1.00
1 – Specificity
AUC = 0.349
95% CI 0.116-0.582

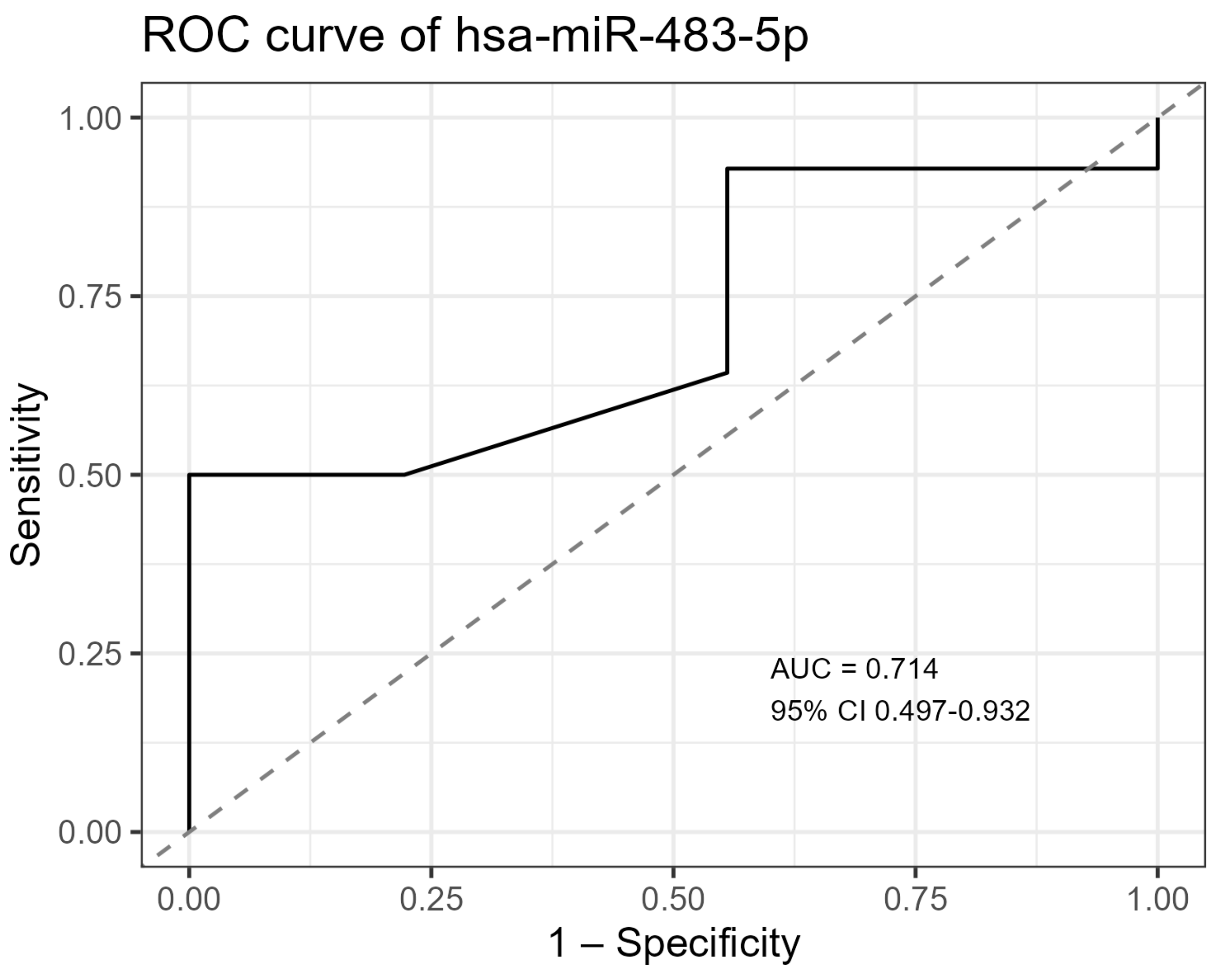
ROC curve of hsa-miR-483-5p
Sensitivity
1.00
0.75
0.50
0.25
0.00
0.00
0.25
0.50
0.75
1.00
1 – Specificity
AUC = 0.714
95% CI 0.497-0.932

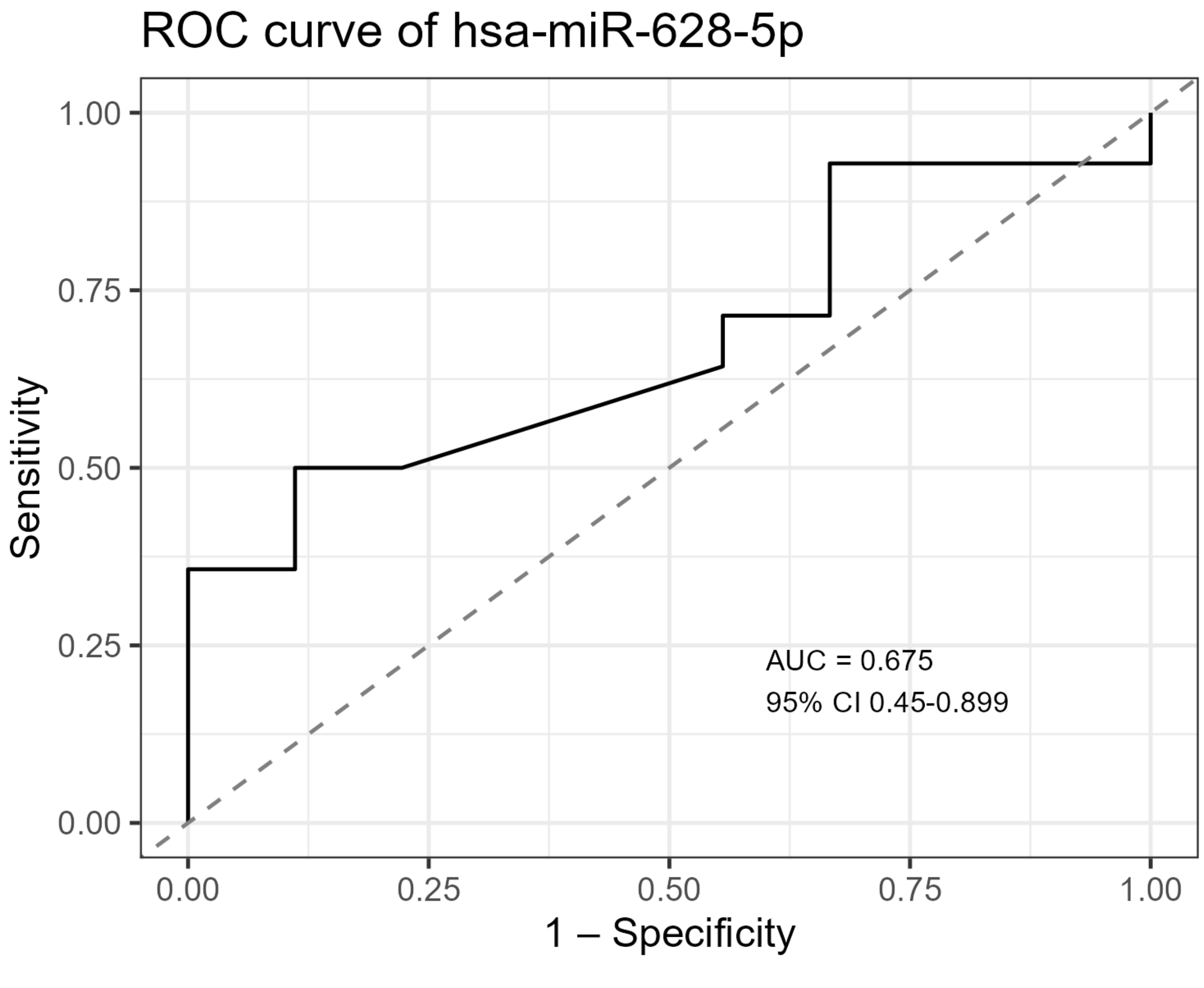

ROC curve of hsa-miR-628-5p
1.00
0.75
0.50
0.25
0.00
Sensitivity
0.00
0.25
0.50
0.75
1.00
1 – Specificity
AUC = 0.675
95% CI 0.45-0.899

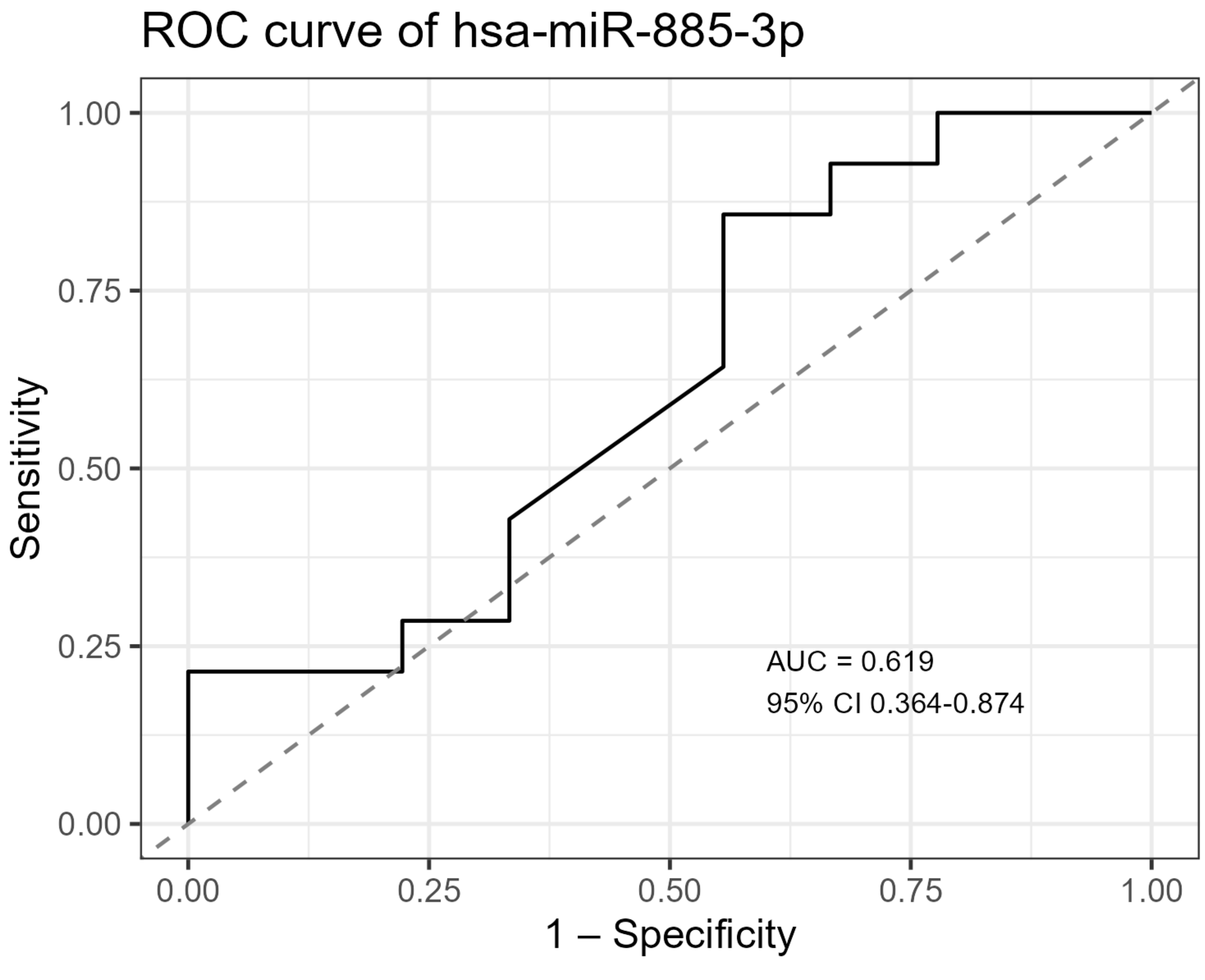
ROC curve of hsa-miR-885-3p
Sensitivity
1.00
0.75
0.50
0.25
0.00
AUC = 0.619
95% CI 0.364-0.874
0.00
0.25
0.50
0.75
1.00
1 – Specificity

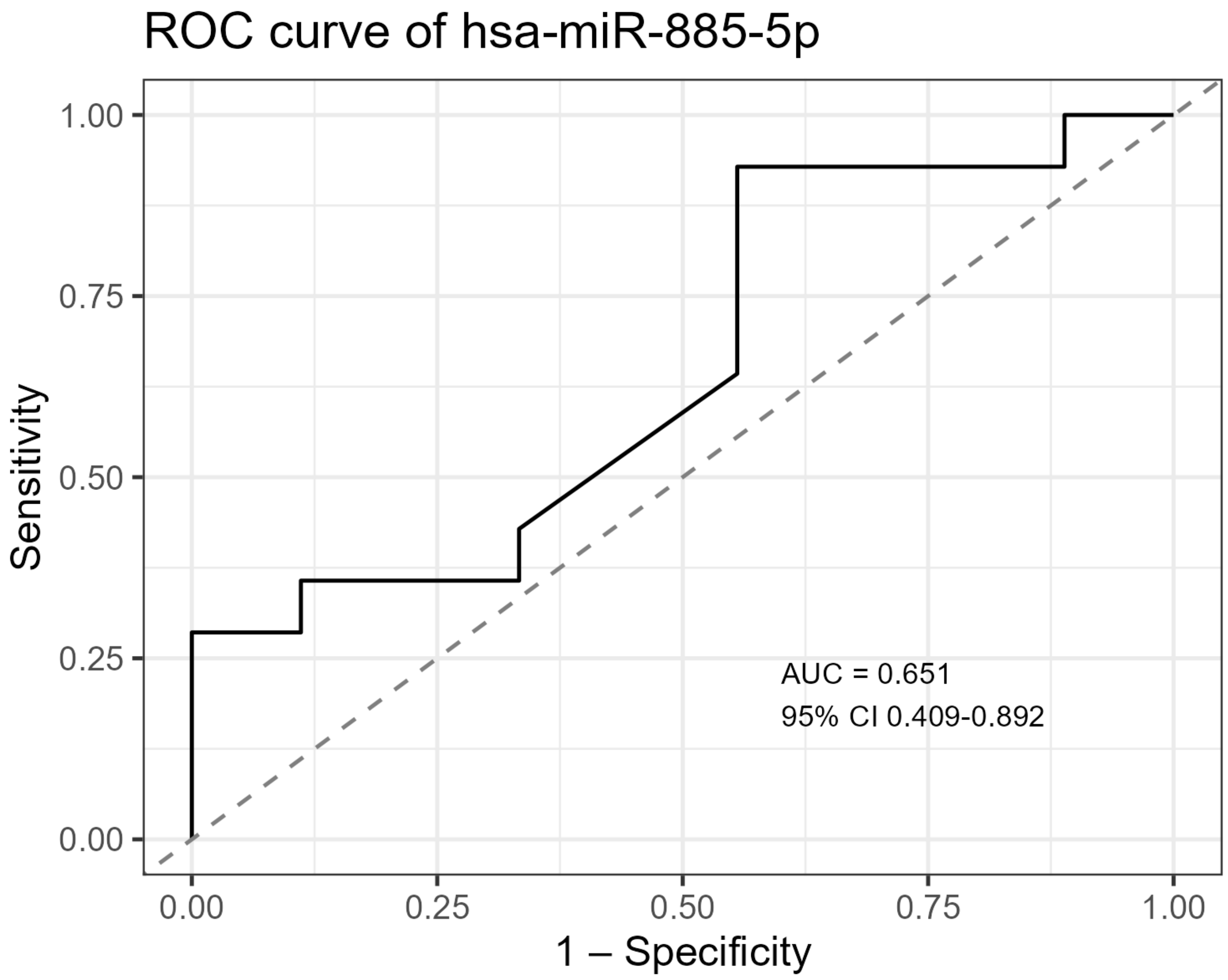
ROC curve of hsa-miR-885-5p
1.00
0.75
0.50
0.25
0.00
Sensitivity
AUC = 0.651
95% CI 0.409-0.892
0.00
0.25
0.50
0.75
1.00
1 – Specificity

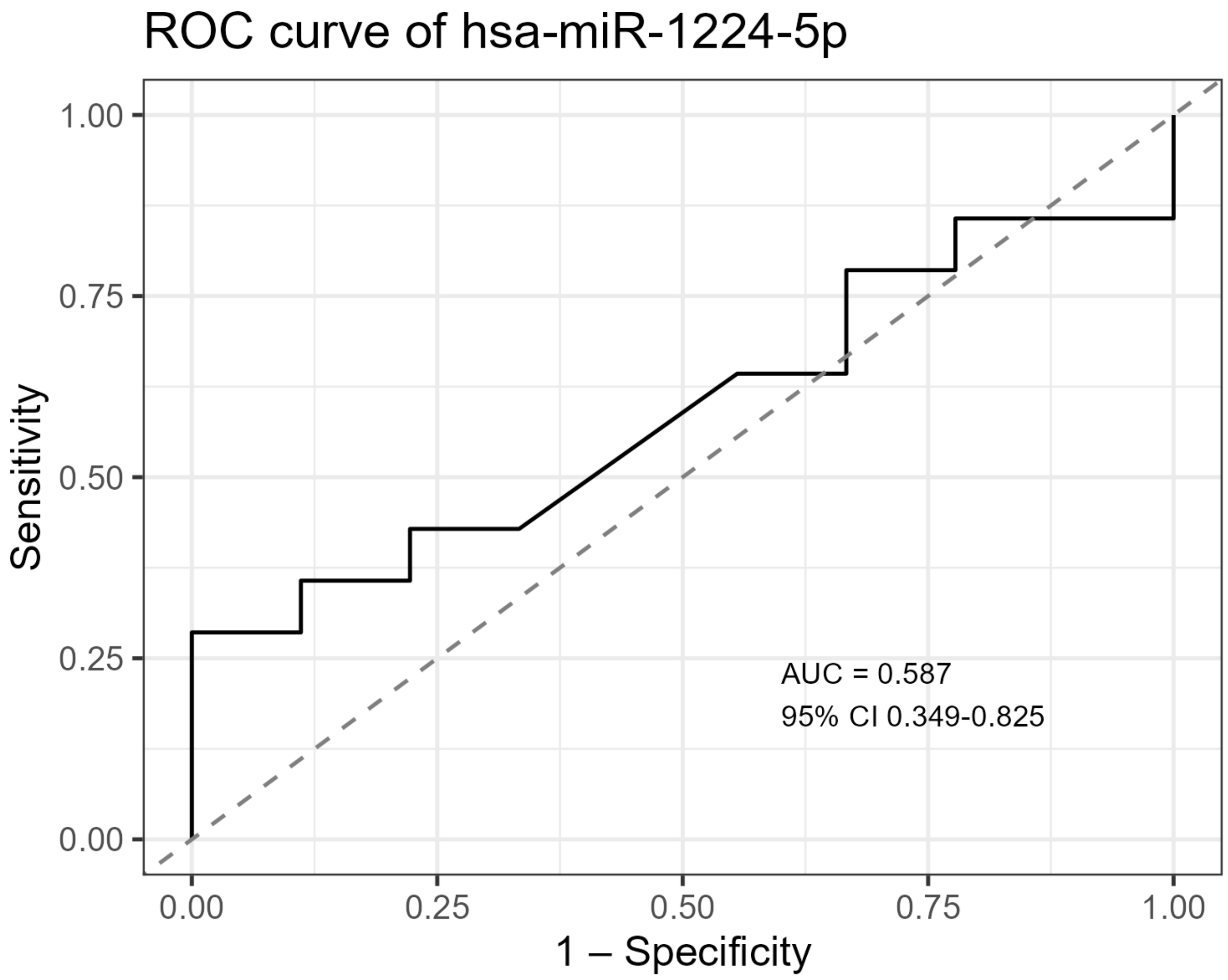
ROC curve of hsa-miR-1224-5p
1.00
0.75
0.50
0.25
0.00
Sensitivity
0.00
0.25
0.50
0.75
1.00
1 – Specificity
AUC = 0.587
95% CI 0.349-0.825

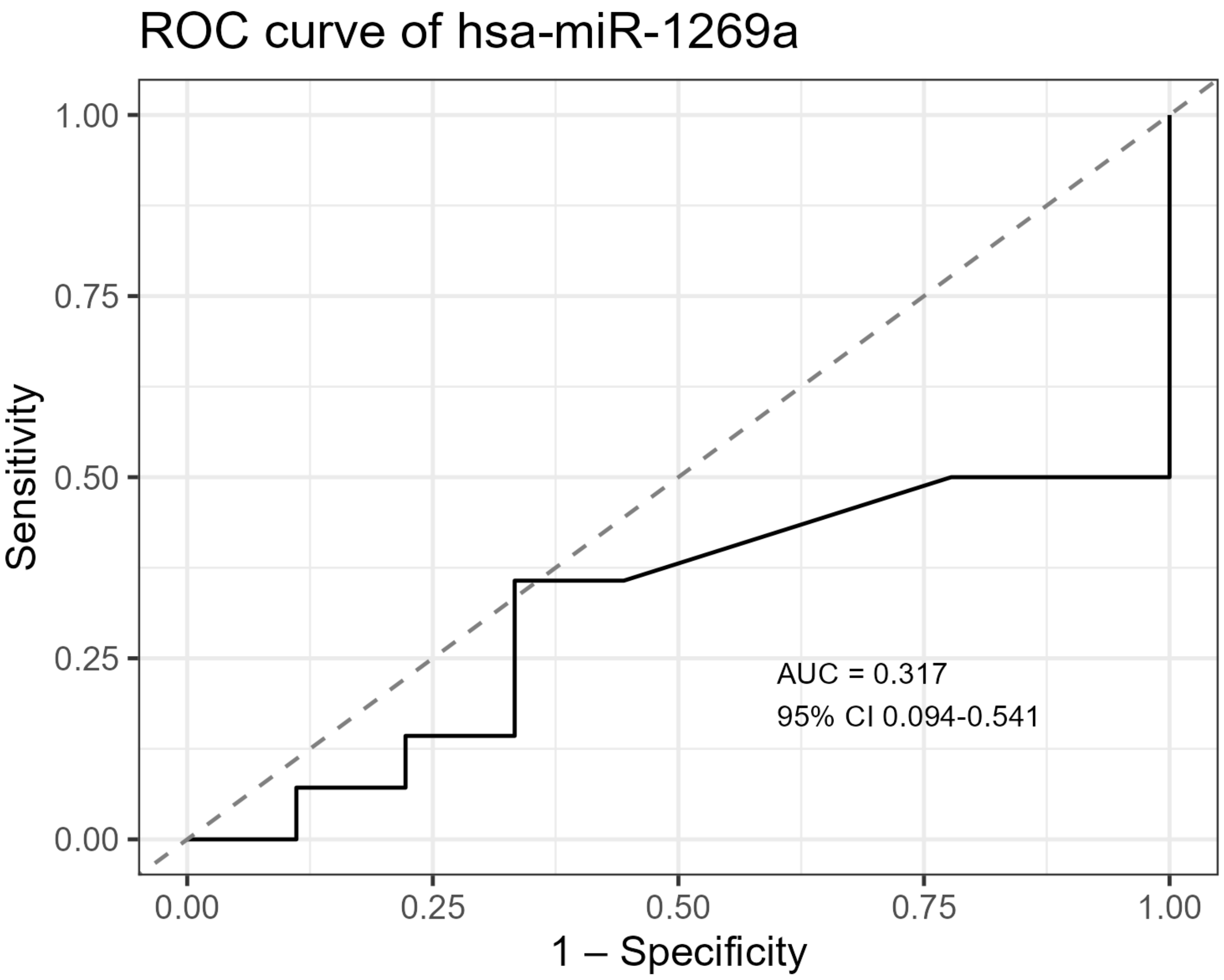

ROC curve of hsa-miR-1269a
Sensitivity
1 – Specificity
0.00
0.25
0.50
0.75
1.00
0.00
0.25
0.50
0.75
1.00
AUC = 0.317
95% CI 0.094-0.541

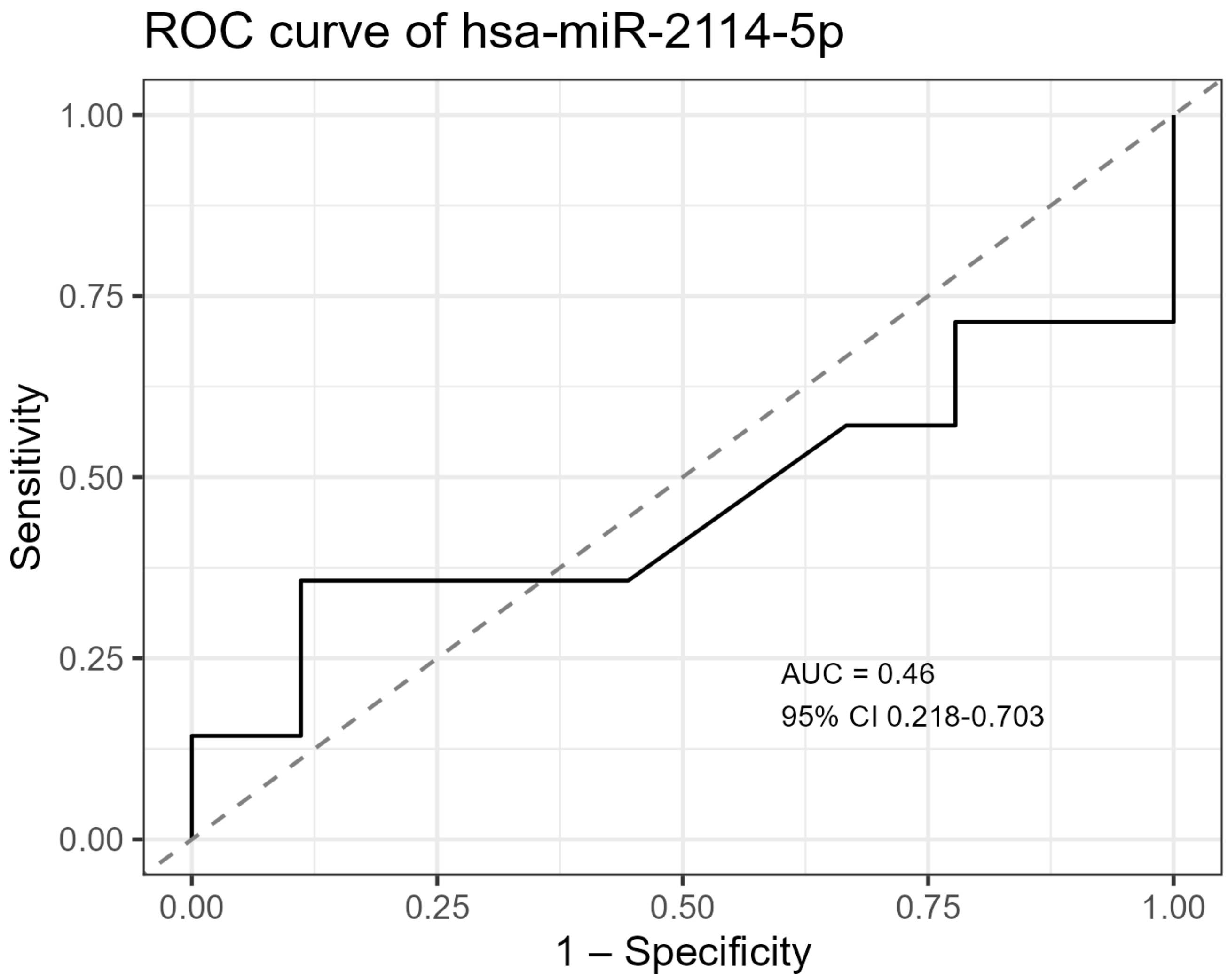
ROC curve of hsa-miR-2114-5p
Sensitivity
1.00
0.75
0.50
0.25
0.00
0.00
0.25
0.50
0.75
1.00
1 – Specificity
AUC = 0.46
95% CI 0.218-0.703

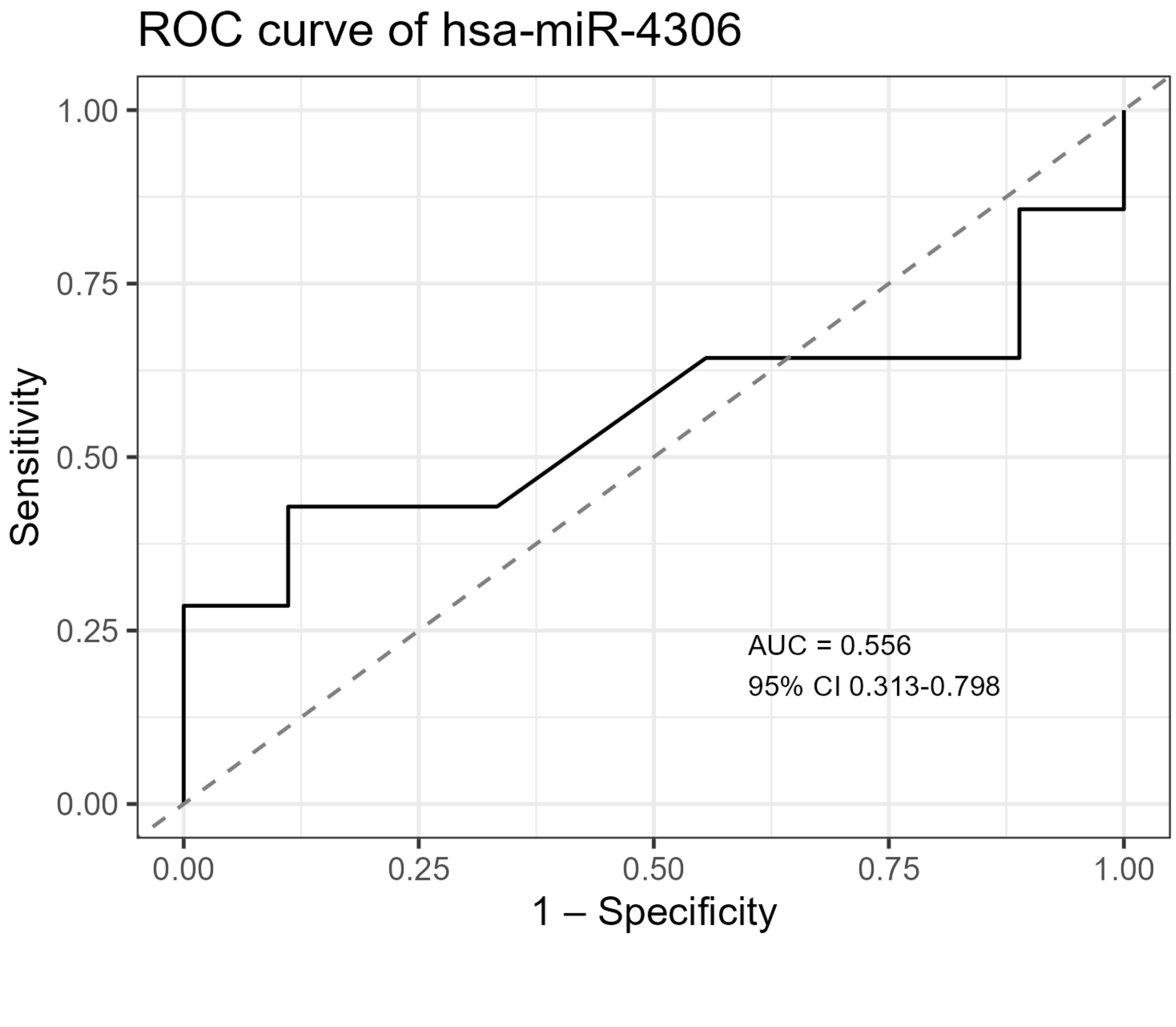
ROC curve of hsa-miR-4306
Sensitivity
1.00
0.75
0.50
0.25
0.00
0.00
0.25
0.50
0.75
1.00
1 – Specificity
AUC = 0.556
95% CI 0.313-0.798
