# Supplementary material for: Optimization of extracellular vesicle extraction from hepatic tissue interstitial fluid and analysis of their ncRNA expression profiles
Source: PLoS One. 2026 Aug 3;21(8):e0355303. doi: 10.1371/journal.pone.0355303 (PMC13432105; doi:10.1371/journal.pone.0355303)
Supplement: S2 File — (PDF) [file pone.0355303.s003.pdf]

S1 Table. Patient Information.

ID	Gender	Group	CNLC	T	N	M	size（cm）	ALT	AST	TBIL	DBIL	TBA	ALB	ALP	r-GGT	NA	Cr	PTA	INR	WBC	N%	PLT	AFP
2246	female	HCC	IIIA	3	0	0	4.3×3.0	18	21	15.5	2.5	4.1	35.3	84	30	138.4	86	92	1.05	8.46	68	1.59	>1210
2247	male	FNH					8.7×10.4	49	27	8.9	1.2	2	47.9	106	224	140.6	69	133	0.87	8.15	62.3	266	2.03
2248	female	HCC	IA	1	0	0	3.5	22	26	15.5	1	10.6	46.9	118	29	135.8	48	131	0.87	8.46	40	184	-
2250	male	HCC	IIIA	3	0	0	9.3×9.2	34	58	11.7	2.1	3.3	38.4	119	106	136.2	67	86	1.09	5.64	60.5	157	3.52
2254	male	HCC	IB	2	0	0	7.6×6.0	131	79	13	2.2	13.2	41.6	131	79	139.2	55	95	1.03	3.35	63.4	110	147
2256	male	HCC	IA	1	0	0	4.7×3.8	18	27	23	1.7	6.4	44.1	65	33	140.1	66	77	1.17	3.61	67.8	62	15.9
2257	male	CH					10.4×13.8	16	18	18.2	2.8	1.4	45.5	54	58	138.1	109	92	1.05	2.76	61.4	77	2.22
2258	male	ICC	IB	1	0	0	5.8×4.6	11	16	21.6	2.6	3.8	43.8	72	25	136.1	75	103	0.98	6.27	67.7	204	49.6
2259	female	HCC	IB	2	0	0	6.7×7.3	28	23	9.7	1.1	5.8	38.3	102	43	140.8	63	116	0.92	5.55	50.8	211	142
2260	male	HCCA	IIIC	3	0	0		47	43	126.8	68.5	8.7	43.4	220	123	141.7	79	105	0.97	7.3	74.2	243	5.57
2262	female	HCC	IIIB	3	0	0	11.0×7.4	208	198	41.7	5.4	1.7	39.3	124	103	139.3	55	78	1.16	7.62	85.7	41	-
2263	female	ICC	IB	1	0	0	4.3×5.0	741	467	60.4	44	233.3	47.2	315	815	142.6	69	81	1.13	8.26	77.8	174	-
2264	female	HCC	IIIA	4	0	0	8.7×7.3	40	155	23	5.1	9.7	46.1	107	159	141.1	55	99	1	3.74	66.9	104	>1210
2266	female	IPT					1.7	10	15	16.6	1.2	1	49.9	95	13	142.8	53	109	0.96	4.61	59.4	188	1.57
2268	male	HCC	IA	2	x	0	3.5×2.7	15	21	17.2	1.6	4	48.4	70	35	140.1	61	68	1.25	2.7	76.5	76	15.9
2269	male	HCC	IIIA	4	0	0	2.7×2.4	27	30	16.5	2.3	20.1	40.6	86	57	139	79	68	1.26	4.63	65.3	103	6.82
2270	male	ICC	IA	1	0	0	2.9×2.5	19	18	12.8	1.4	3.9	45.3	61	95	138.3	45	92	1.04	6.56	66	135	13.8
2272	female	ICC	IIB	2	0	0	6.2×3.7×9	23	25	20.7	2.3	4.5	49.2	91	29	142.5	57	113	0.94	-	-	-	1.75
2274	female	HCC	IIA	3	0	0	6.2×5.0	19	36	9	0.9	21.7	35.2	191	82	138.2	72	86	1.09	2.96	66.3	42	12.5
2275	female	HCC	IB	1	0	0	3.5×3.3	70	89	35	6.7	32.6	36.9	86	53	142.1	-	75	1.19	3.7	49.9	72	9.49
2276	male	HCC	IIIB	4	x	1	9.1×6.7×7.3	12	28	13.2	2.7	1.3	38.8	90	60	144.3	73	97	1.01	6.44	59.8	233	6.99
2279	female	CH						9	14	12.9	5.1	1.3	46.4	50	13	137.1	53	114	0.93	4.22	63.7	266	2.54
2280	female	HCC	IIA	2	0	0	7.6×5.0＆2.0×1.8	90	58	13.4	6.1	1.2	44.6	93	123	3.26	56	120	0.91	4.48	68.1	93	565

Caption. HCC: Hepatocellular Carcinoma; FNH: Focal Nodular Hyperplasia; CH: Cavernosum Hemangioma; HCCA: Hilarcholangiocarcinoma; ICC: Intrahepatic Cholangiocarcinoma; IPT: Inflammatory Pseudotumor;

S2 Table. Antibody Information

Antigen	Antibody	Clonality	Catalog No	Dilution	Species	Source
**CD81**	Anti-CD81 Antibody	Monoclonal	ab109201	1:700	Rabbit	Abcam,Cam,UK
**CD63**	Anti-CD63 Antibody	Monoclonal	ab134045	1:1000	Rabbit	Abcam,Cam,UK
**CD9**	Anti-CD9 Antibody	Monoclonal	ab263019	1:1000	Rabbit	Abcam,Cam,UK
**TSG101**	Anti-TSG101 Antibody	Monoclonal	ab125011	1:1000	Rabbit	Abcam,Cam,UK
**HSP70**	Anti-Hsp70 Antibody	Monoclonal	ab181606	1:1000	Rabbit	Abcam,Cam,UK
**ALIX**	Anti-ALIX Antibody	Monoclonal	ab275377	1:1000	Rabbit	Abcam,Cam,UK
**ASGPR**	Anti-ASGR2 Antibody	Monoclonal	ab200196	1:1000	Rabbit	Abcam,Cam,UK
**CD34**	Anti-CD34 Antibody	Monoclonal	ab81289	1:1000	Rabbit	Abcam,Cam,UK
**ALB**	Serum Albumin/ALB Antibody	Monoclonal	HY-P78077	1:1000	Rabbit	MCE
**CK19**	Cytokeratin 19 Antibody	Monoclonal	HY-P81183	1:1000	Rabbit	MCE

S3 Table. lncRNA Primer Information.

lncRNA	Primer Names	Forward/Reverse(5´-3´)	amplicon(bp)	Reference Sequence Number (transcriptvariant)	Primer Location(nt)	
GAPDH	3F	GGGCATCCTGGGCTACACTGA	143	NM_002046	Exon 8	889-909
	145R	CAAATTCGTTGTCATACCAGGAAATG			Exon 8/9	1031-1006
AL031985.3	831F	AGACCCACTGATGAATGTGTGC	81	ENSG0000026092	Single Exon	831-852
	911R	CTTGAGCCAAACGAAACCTAAC				911-890
AL158166	432F	AAGAGAGATGAATTTGCAGGCCTT	149	ENSG00000232259	Exon 1/2	432 455
	580R	TGTGACGTTTCCAGAGGGTT			Exon 2	580-561
CERNA2	343F	CATCTACAACTTGGCCCAACCAC	161	NR_134505	Exon 1/2	343-365
	503R	CCATGCGTGAACCAGTCAGCTTC			Exon 2	503-481
GAS5	18F	CGACTCCTGTGAGGTATGGTGCTG	79	NR_152522 (tv 3)	Exon 1/2	18-41
	96R	CTTGGGGACACAACTGTCCATAAG			Exon 3/2	96-73
H19	1317F	GAGACGAGGCCAGGTCTCCA	161	NR_002196.3(tv1)	Exon 1/2	1317-1336
	1477R	CACCAGCCTAAGGTGTTCAGG			Exon 3/2	1477-1457
LINC00622	232F	GCCCTCCTCCCATGTTGAAA	199	NR_036540	Single Exon	232-51
	430R	GCAGGCAAAGGTGTGTGATG				430-411
LINC00839	525F	ACCTGTGGCATCCATCTCTG	116	NR_026827	Exon 2	525-544
	640R	GTTTGAGCCCCTTGTTGGAC			Exon 3	640-621
LINC03067	609F	ACCAATAATCGTTACCAGGTACTGAG	116	ENST00000605200_1	Exon 1/2	609-634
	724R	AGAGGGTGTCACCTGGGAGCA			Exon 3/2	724-704
SNHG1	23F	TTGGCTCAAAGGGCCAGCAC	103	NR_003098(tv1)	Exon 7/8	23-42
	125R	ATTTGCTCAGACCTGTAACTTCAG			Exon 10/9	125-102
ST8SIA6-AS1	84F	CATGCTCCTCCTTGCTCCAA	148	NR_034129	Exon 1	84-103
	231R	TGGCACAGATATCAGGTGAAGG			Exon 2	231-210
TMCC1-AS1	47F	AGTTGCTCCAGTGCCACTGGGTTA	177	NR_037893	Exon 1/2	47-70
	223R	GTTGATTTGGTCCACGGGGATTGC			Exon 3/2	223-200

S4 Table. miRNA Primer Information.

miRNA	Primer code
U6	miRAN0002-1-200
miR-122-3p	miRA1000396-1-200
miR-122-5p	miRA0000421-1-200
miR-142-5p	miRA1000133-1-200
miR-148b-5p	miRA1001754-1-200
miR-192-5p	miRA1000028-1-200
miR-194-5p	miRA1000344-1-200
miR-483-5p	miRA1000846-1-200
miR-885-3p	miRACM001-22
miR-885-5p	miRA1001552-1-200
miR-4306	miRA1000244-1-200
miR-16-5p	miRA0000069-1-200
miR-21-3p	miRA1000357-1-200
miR-21-5p	miRA0000076-1-200
miR-130a-3p	miRA0000425-1-200
miR-197-3p	miRA1000142-1-200
miR-214-3p	miRA0000271-1-200
miR-451a	miRA0001631-1-200
miR-452-5p	miRA1000690-1-200
miR-628-5p	miRA1001643-1-200
miR-1269a	miRA1001468-1-200
miR-1224-5p	miRA1001542-1-200
miR-2114-5p	miRACM001-22
