# Supplementary material for: Optimization of extracellular vesicle extraction from hepatic tissue interstitial fluid and analysis of their ncRNA expression profiles
Source: PLoS One. 2026 Aug 3;21(8):e0355303. doi: 10.1371/journal.pone.0355303 (PMC13432105; doi:10.1371/journal.pone.0355303)
Supplement: S3 File — (PDF) [file pone.0355303.s004.pdf]

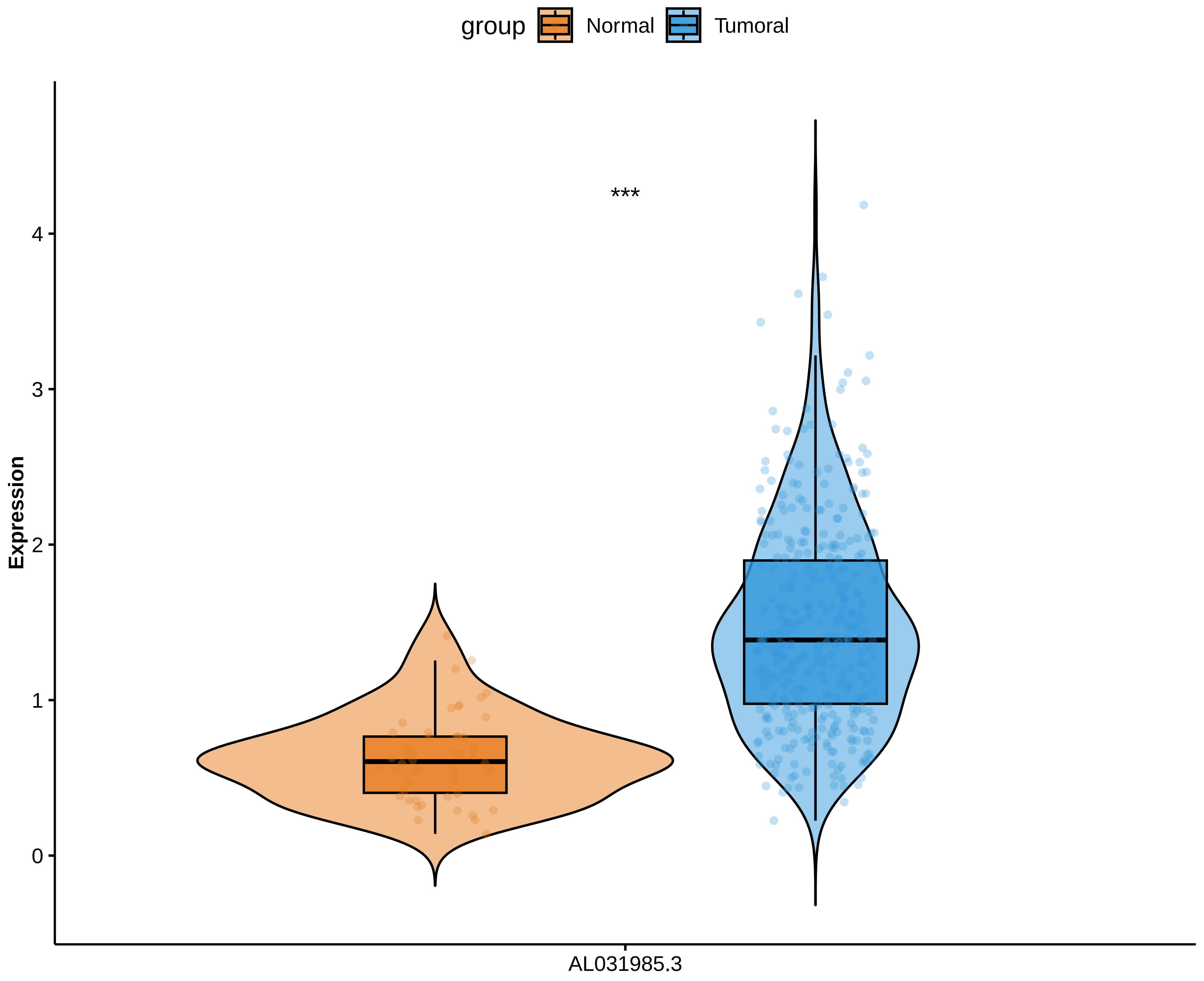
group
Normal
Tumoral
***
Expression
0
1
2
3
4
AL031985.3

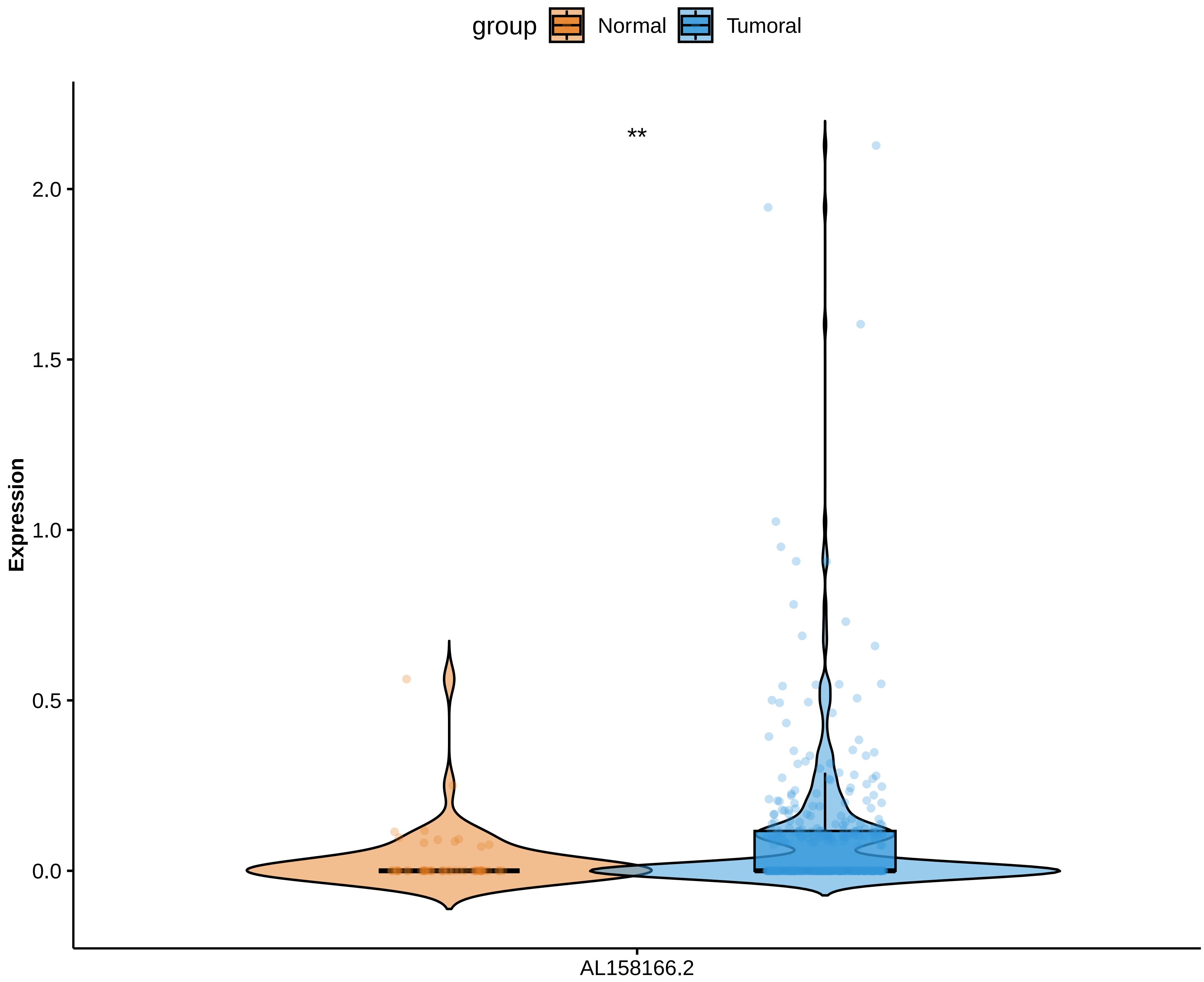

group
Normal
Tumoral
**
Expression
2.0
1.5
1.0
0.5
0.0
AL158166.2

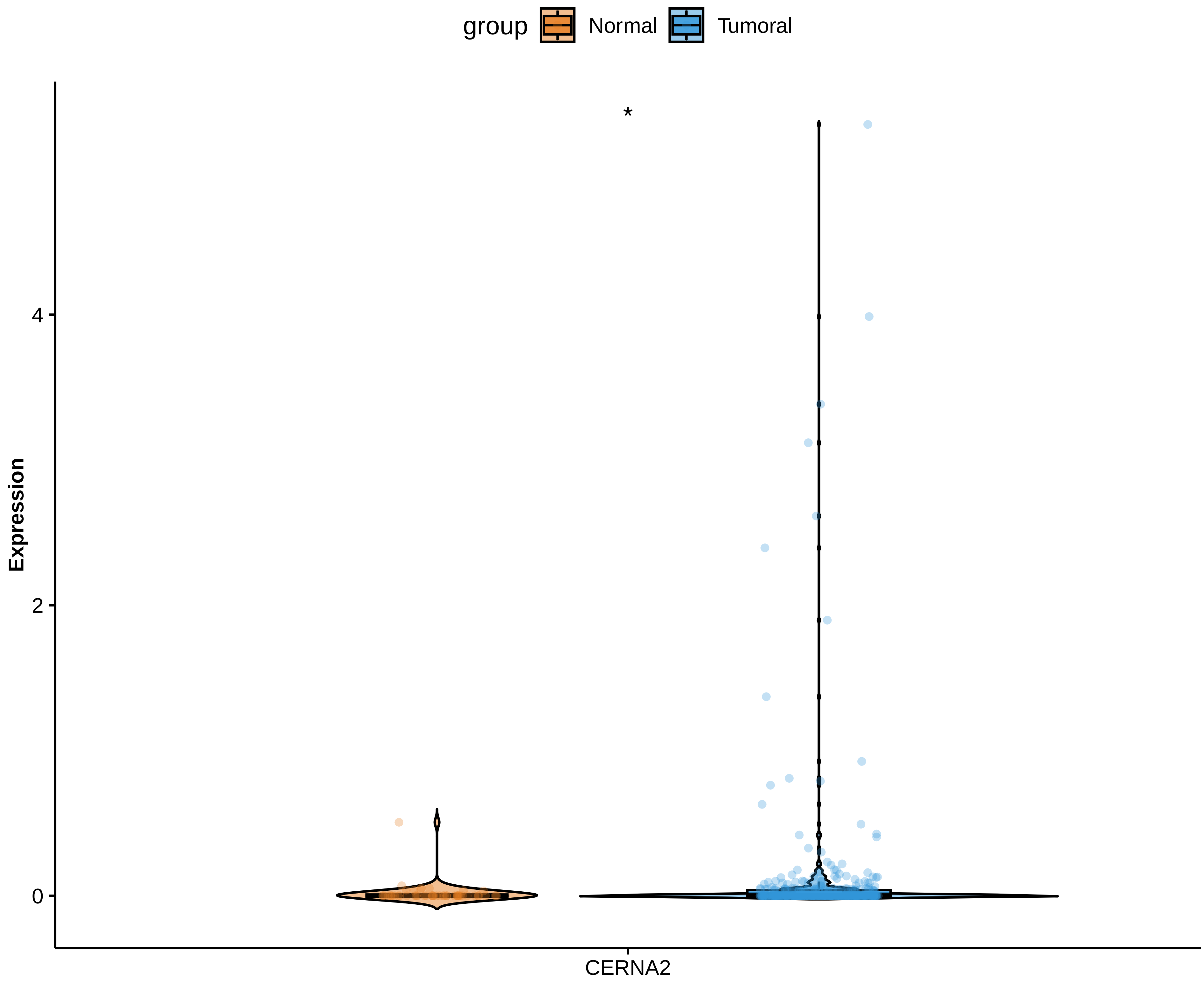
group
Normal
Tumoral
*
Expression
0
2
4
CERNA2

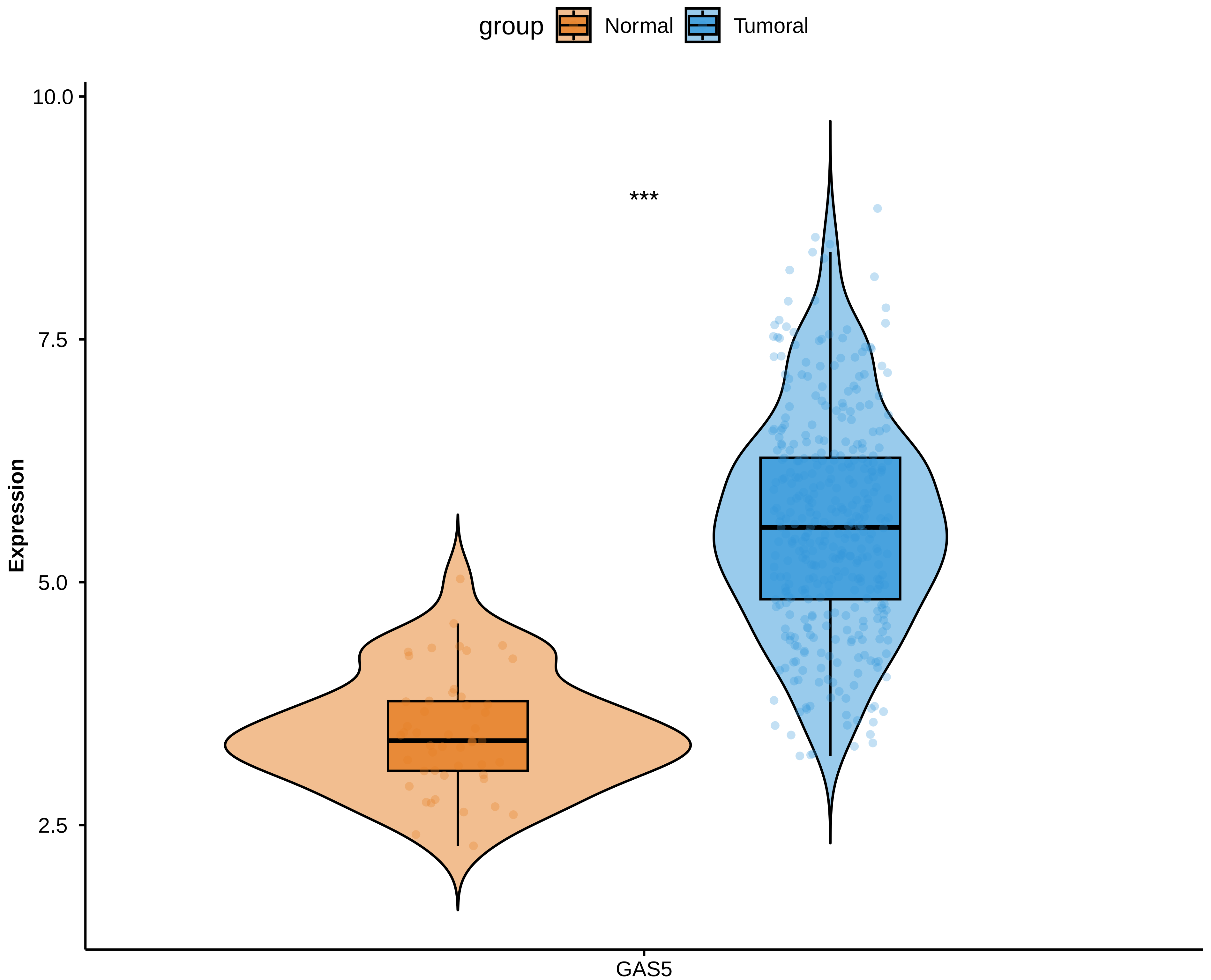
group
Normal
Tumoral
10.0
7.5
5.0
2.5
Expression
***
GAS5

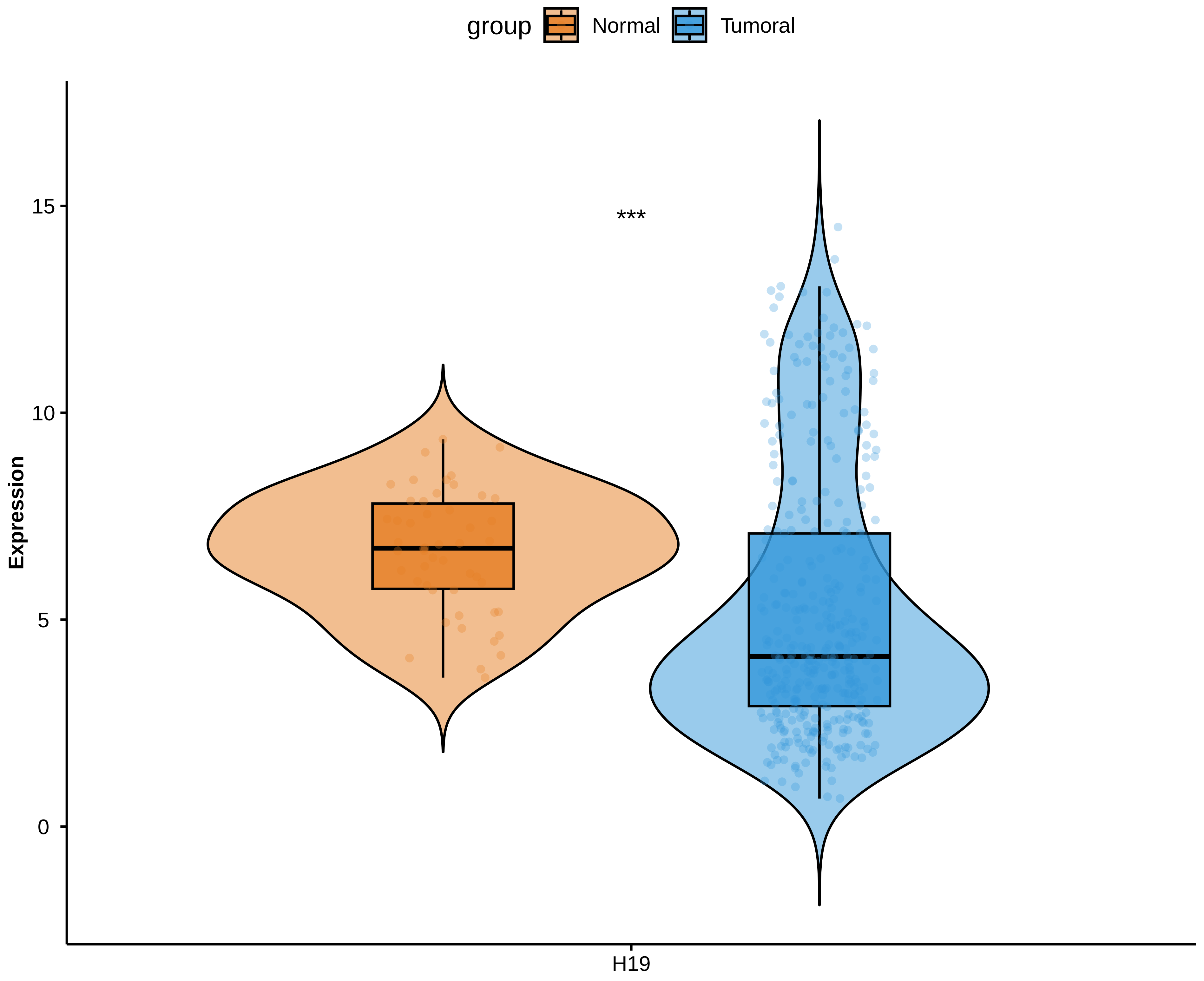
group
Normal
Tumoral
Expression
15
10
5
0
***
H19

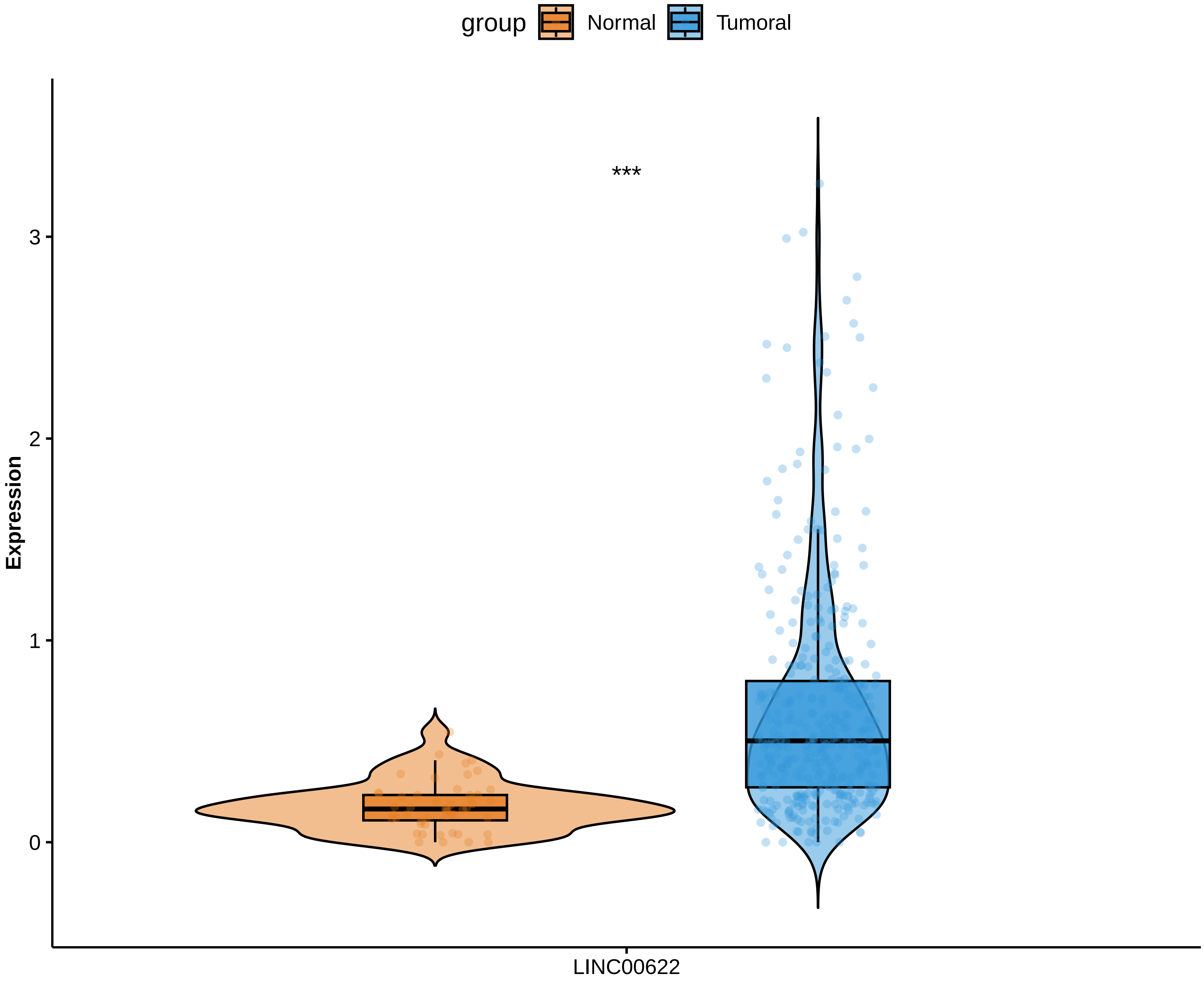
group
Normal
Tumoral
***
3
2
1
0
Expression
LINC00622

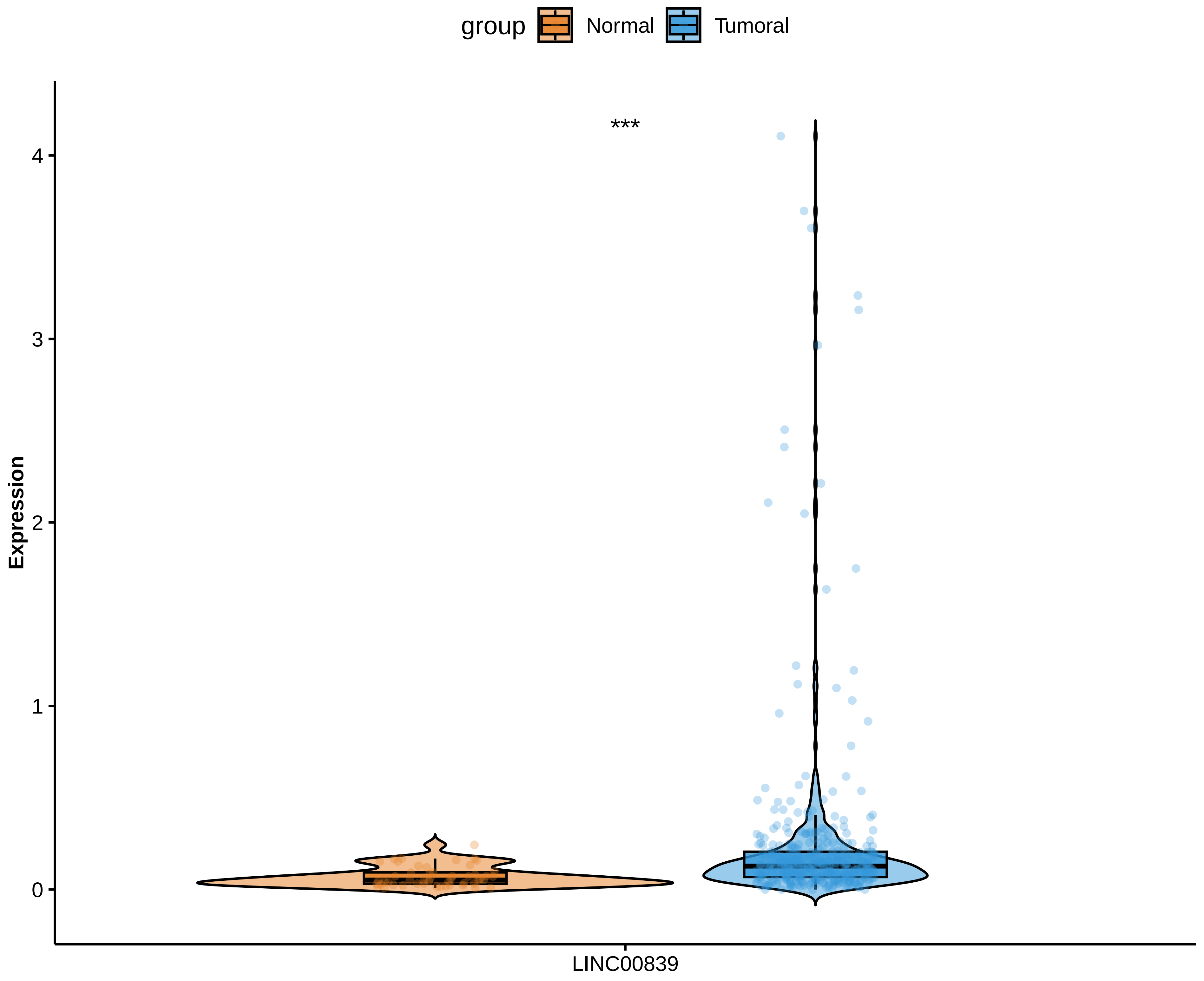
group
Normal
Tumoral
***
Expression
4
3
2
1
0
LINC00839

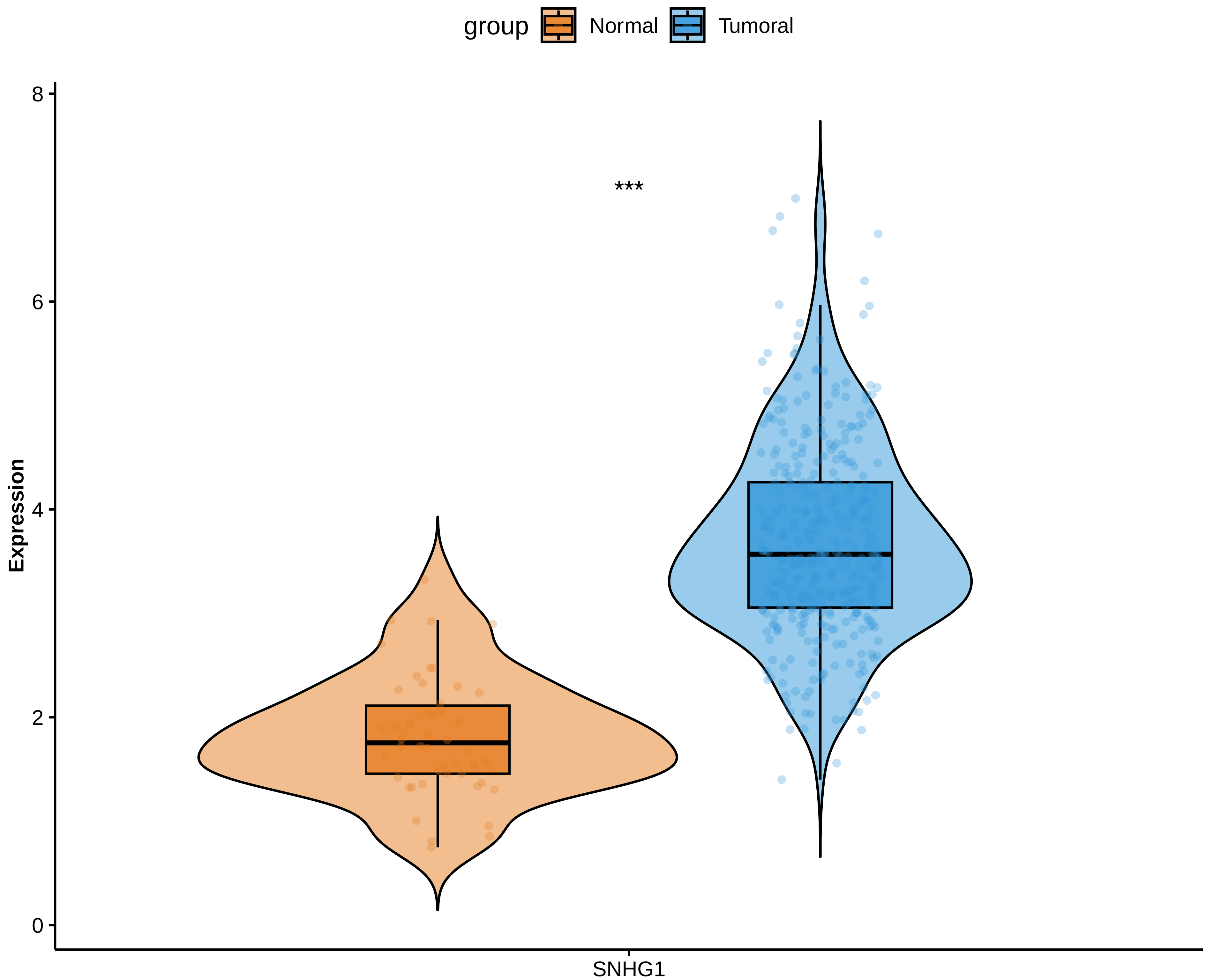
group
Normal
Tumoral
***
8
6
4
2
0
Expression
SNHG1

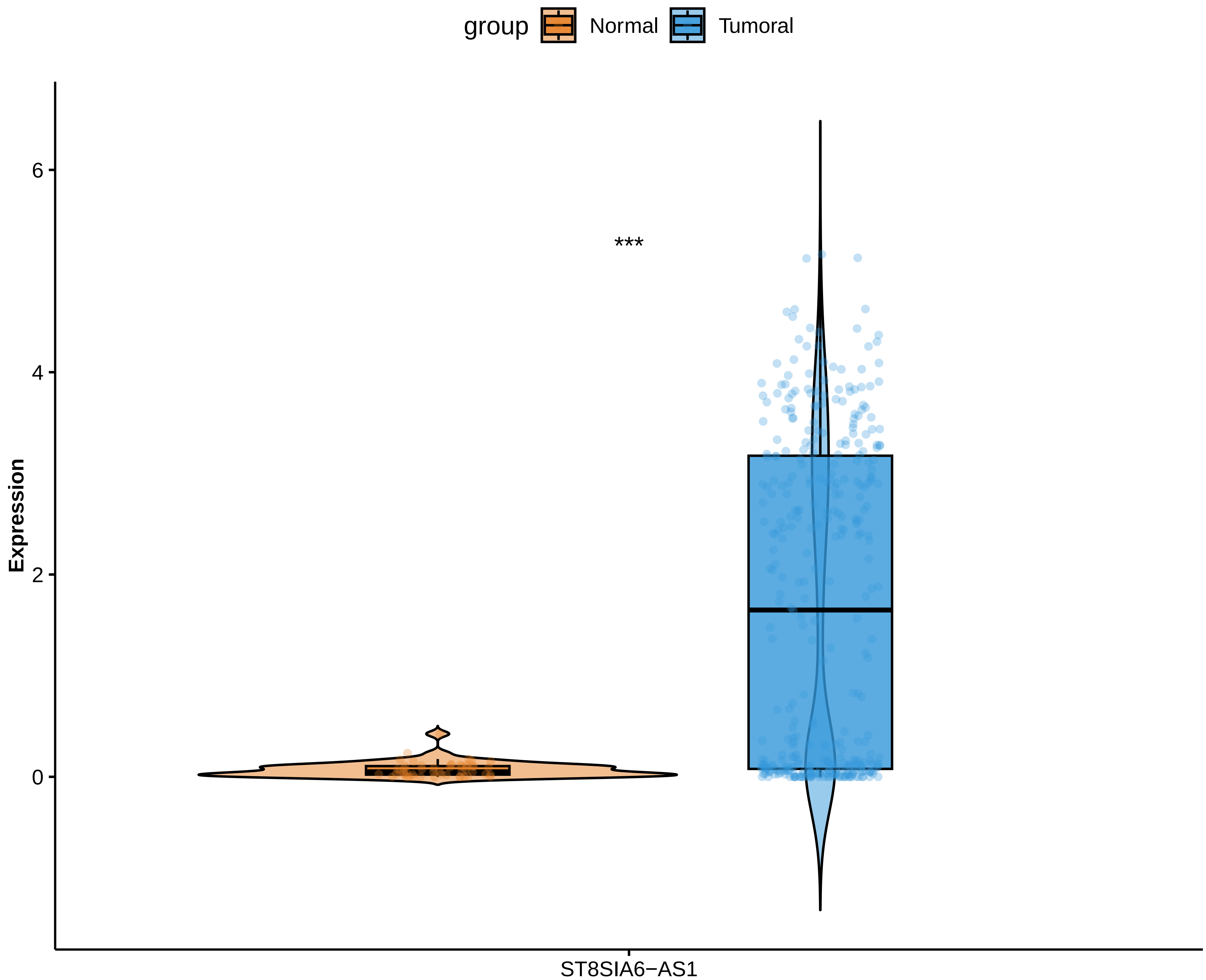
group
Normal
Tumoral
Expression
6
4
2
0
***
ST8SIA6−AS1

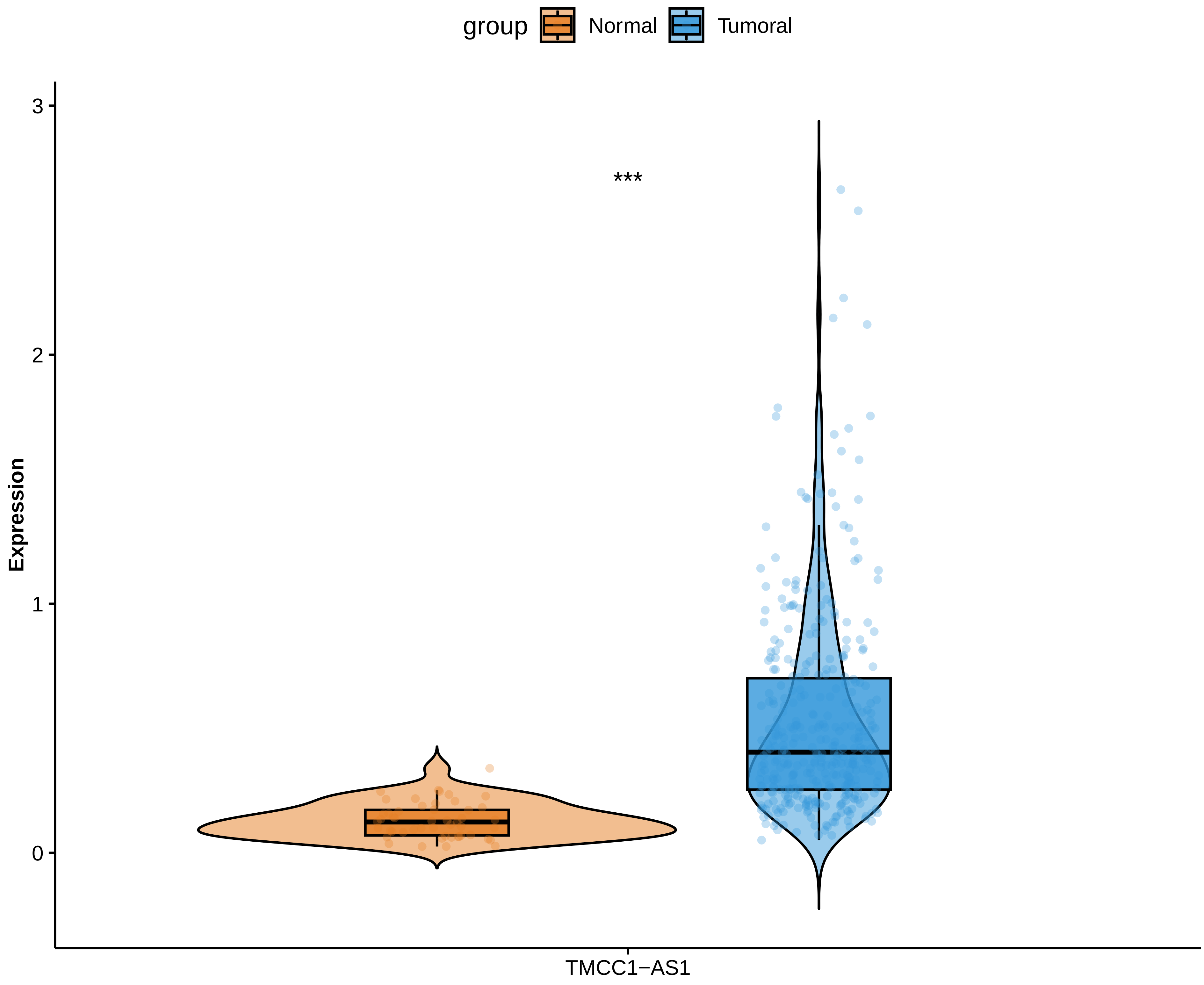
group
Normal
Tumoral
***
Expression
3
2
1
0
TMCC1−AS1

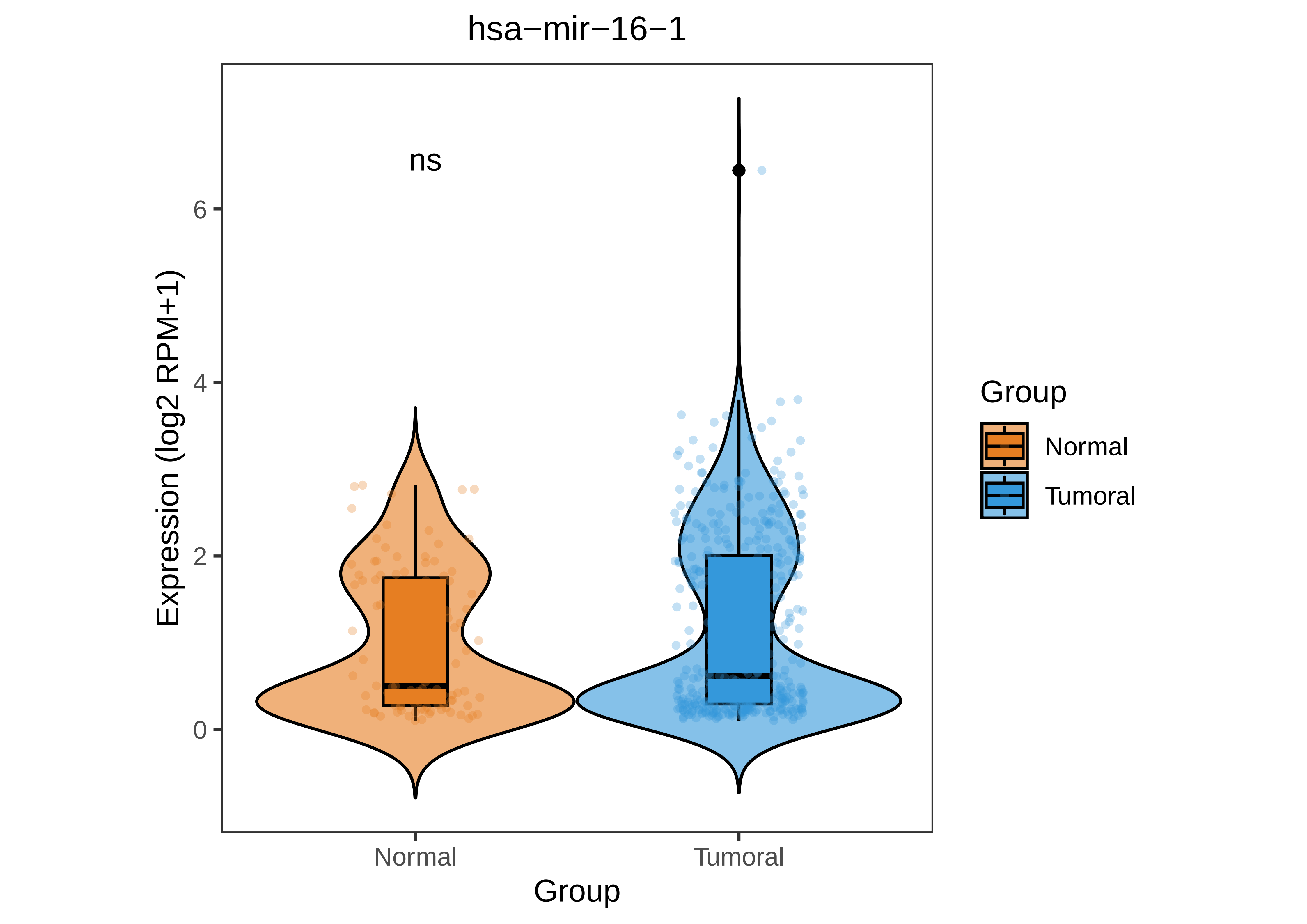

hsa−mir−16−1
ns
Expression (log2 RPM+1)
0
2
4
6
Normal
Tumoral
Group
Group
Normal
Tumoral

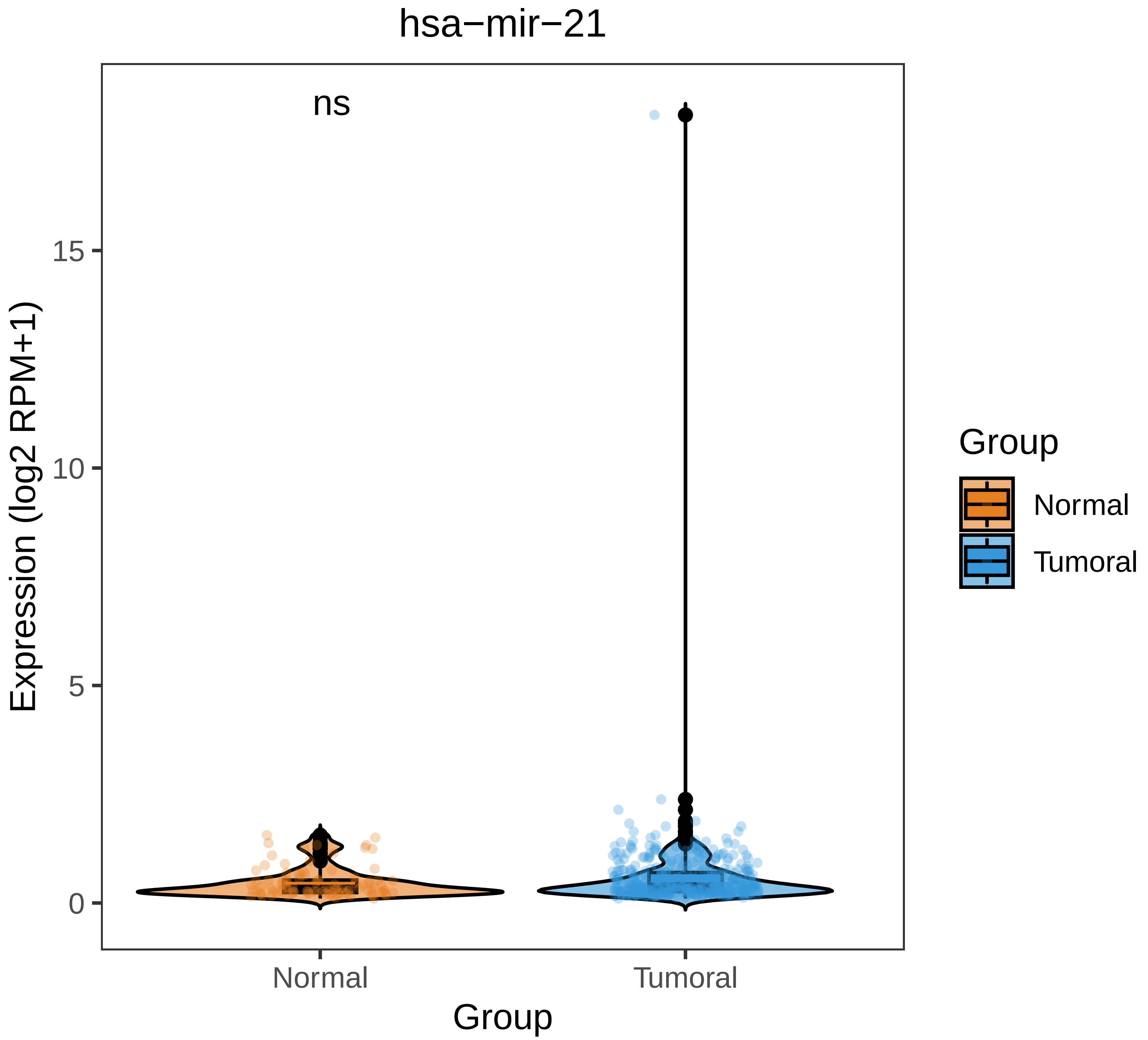
hsa−mir−21
ns
Expression (log2 RPM+1)
15
10
5
0
Normal
Tumoral
Group
Group
Normal
Tumoral

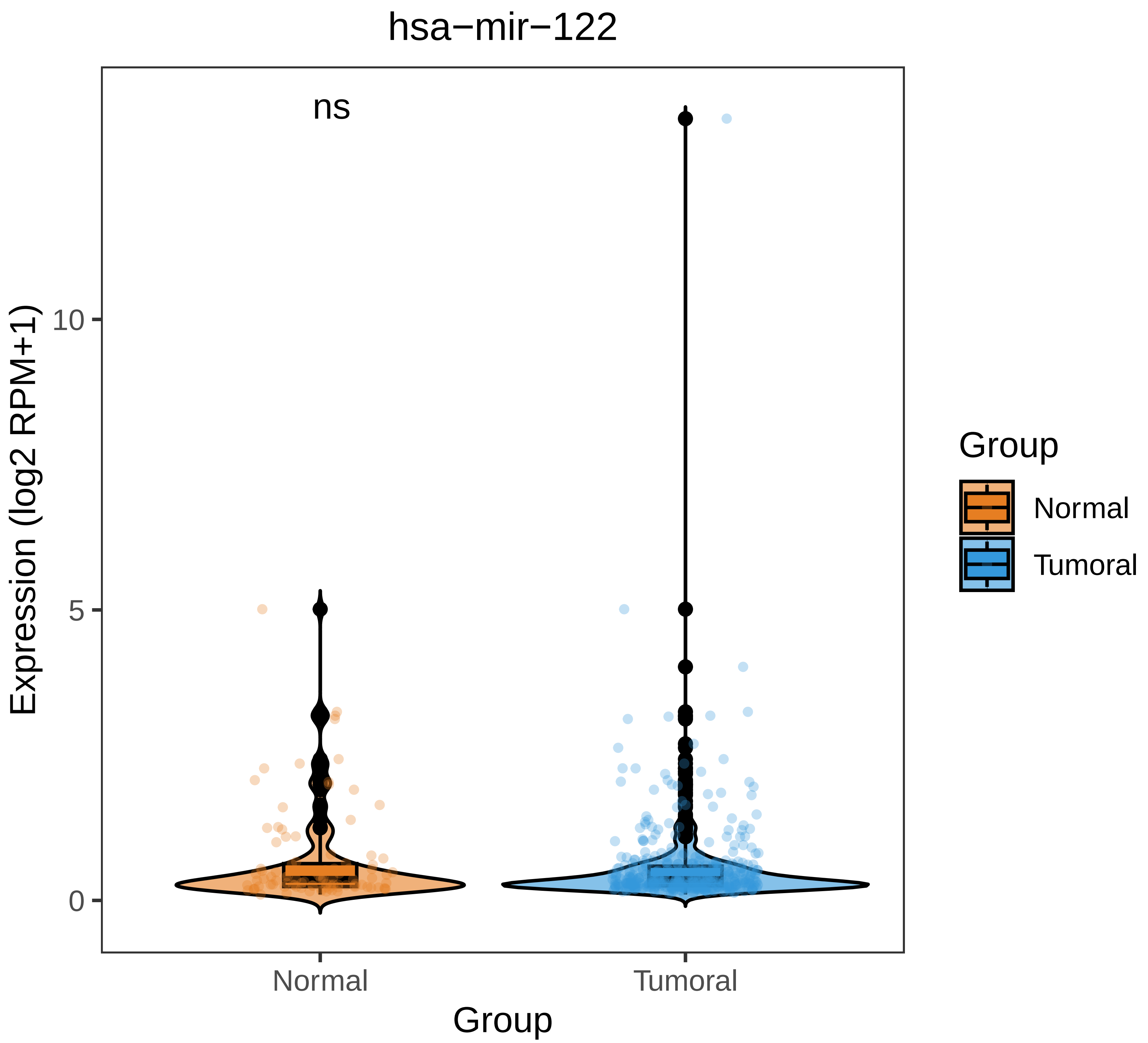
hsa−mir−122
ns
Expression (log2 RPM+1)
10
5
0
Normal
Tumoral
Group
Group
Normal
Tumoral

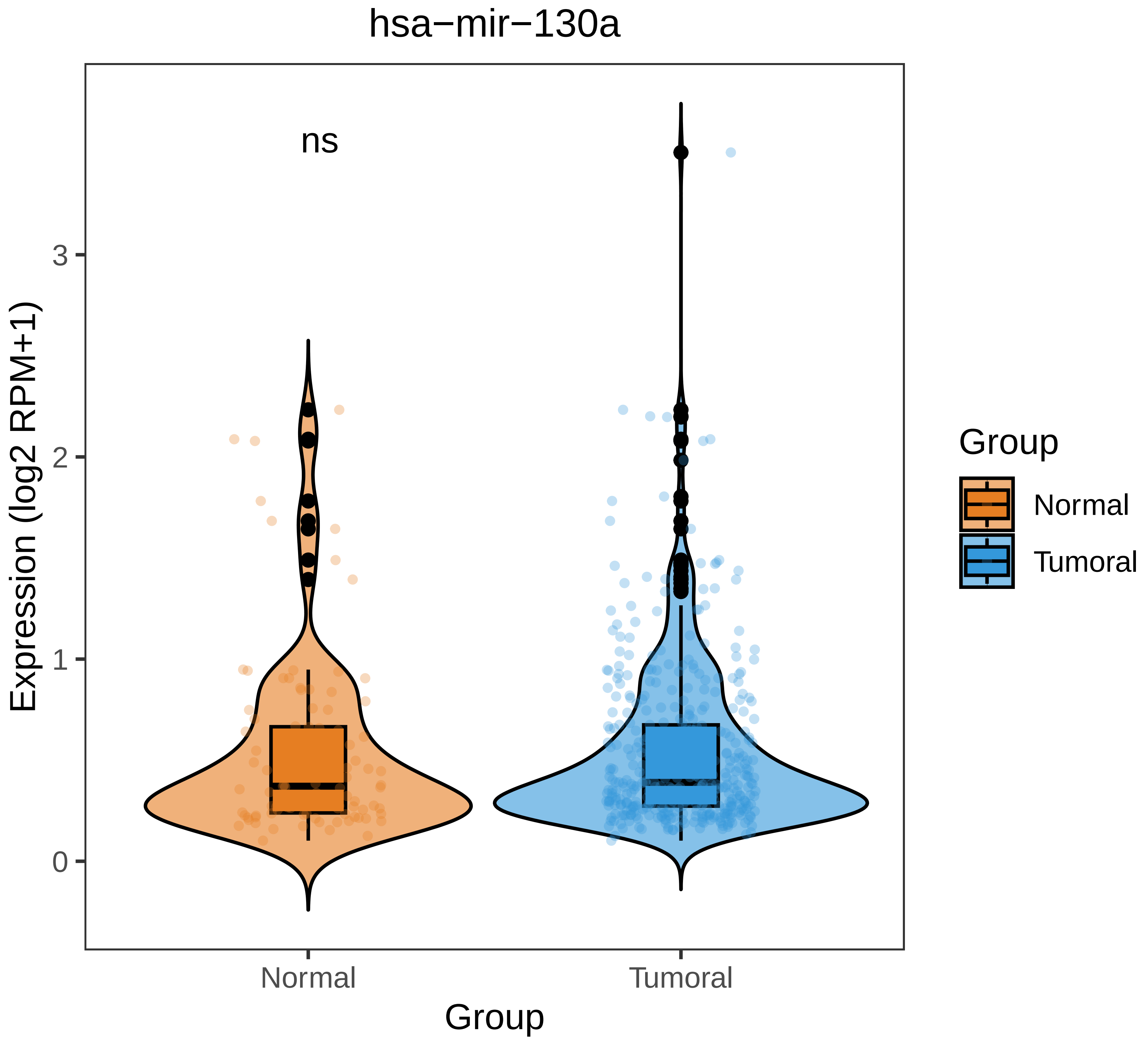
hsa−mir−130a
ns
Expression (log2 RPM+1)
0
1
2
3
Normal
Tumoral
Group
Group
Normal
Tumoral

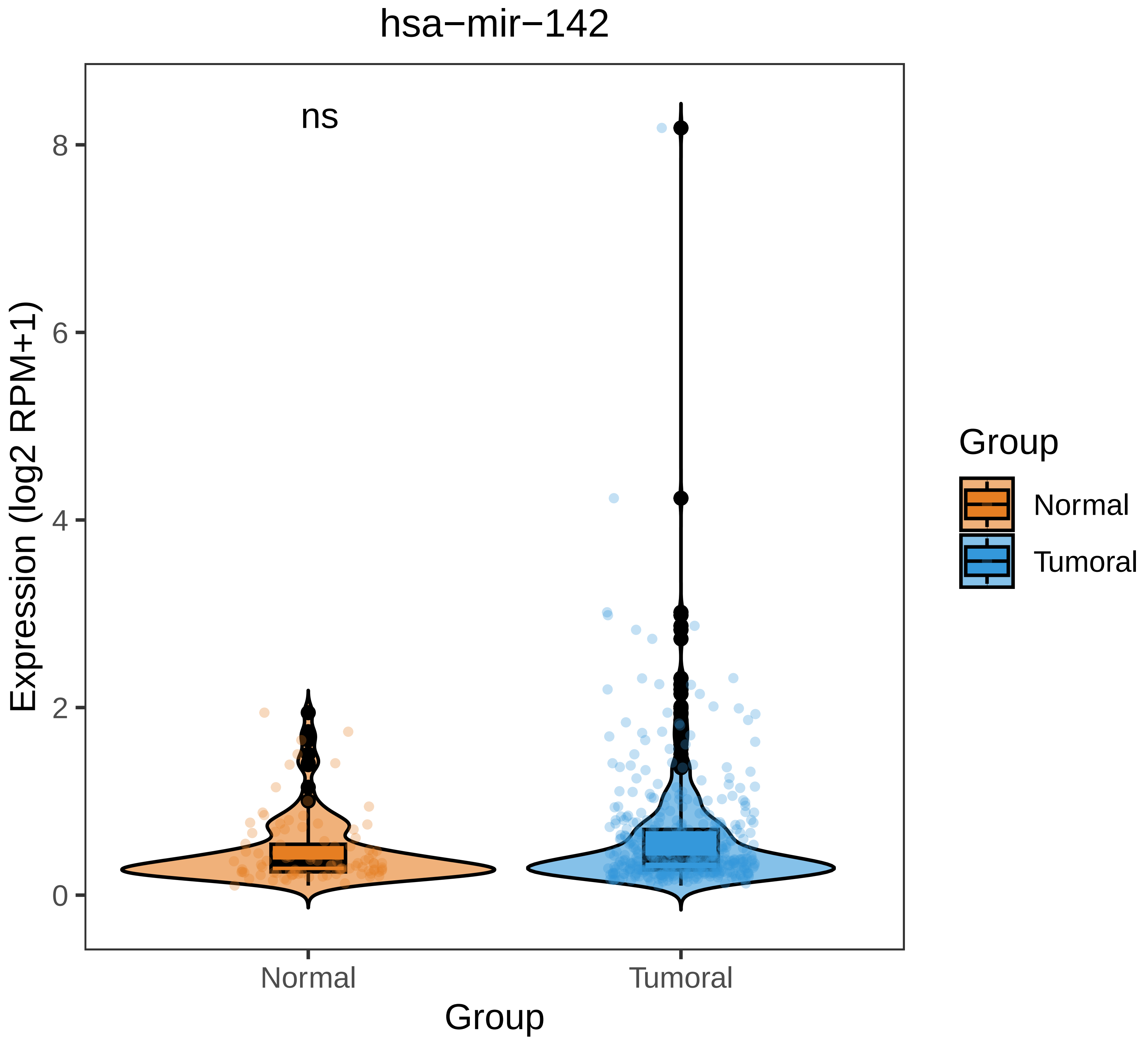
hsa−mir−142
ns
Expression (log2 RPM+1)
8
6
4
2
0
Normal
Tumoral
Group
Group
Normal
Tumoral

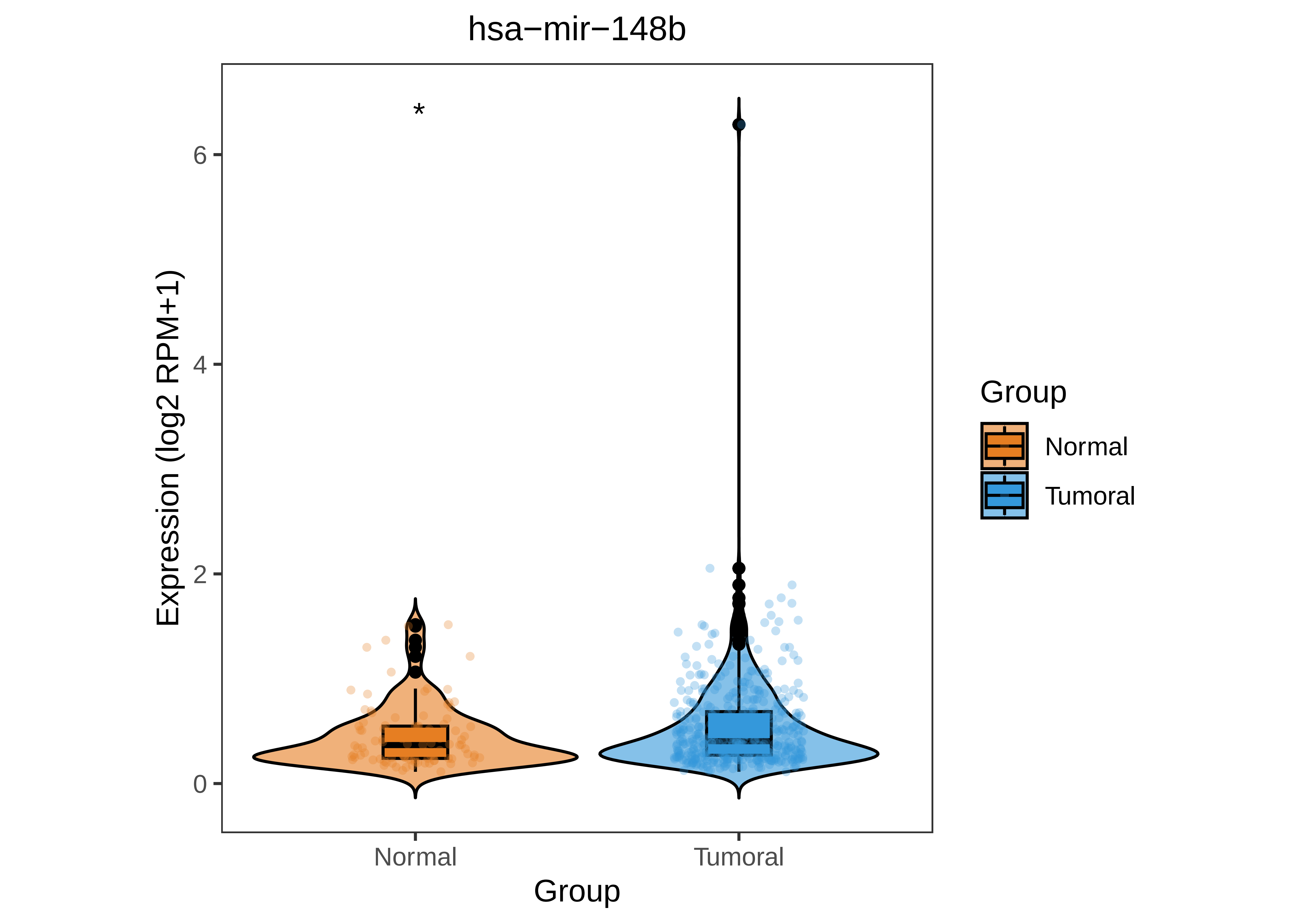

hsa−mir−148b
*
Expression (log2 RPM+1)
0
2
4
6
Normal
Tumoral
Group
Group
Normal
Tumoral

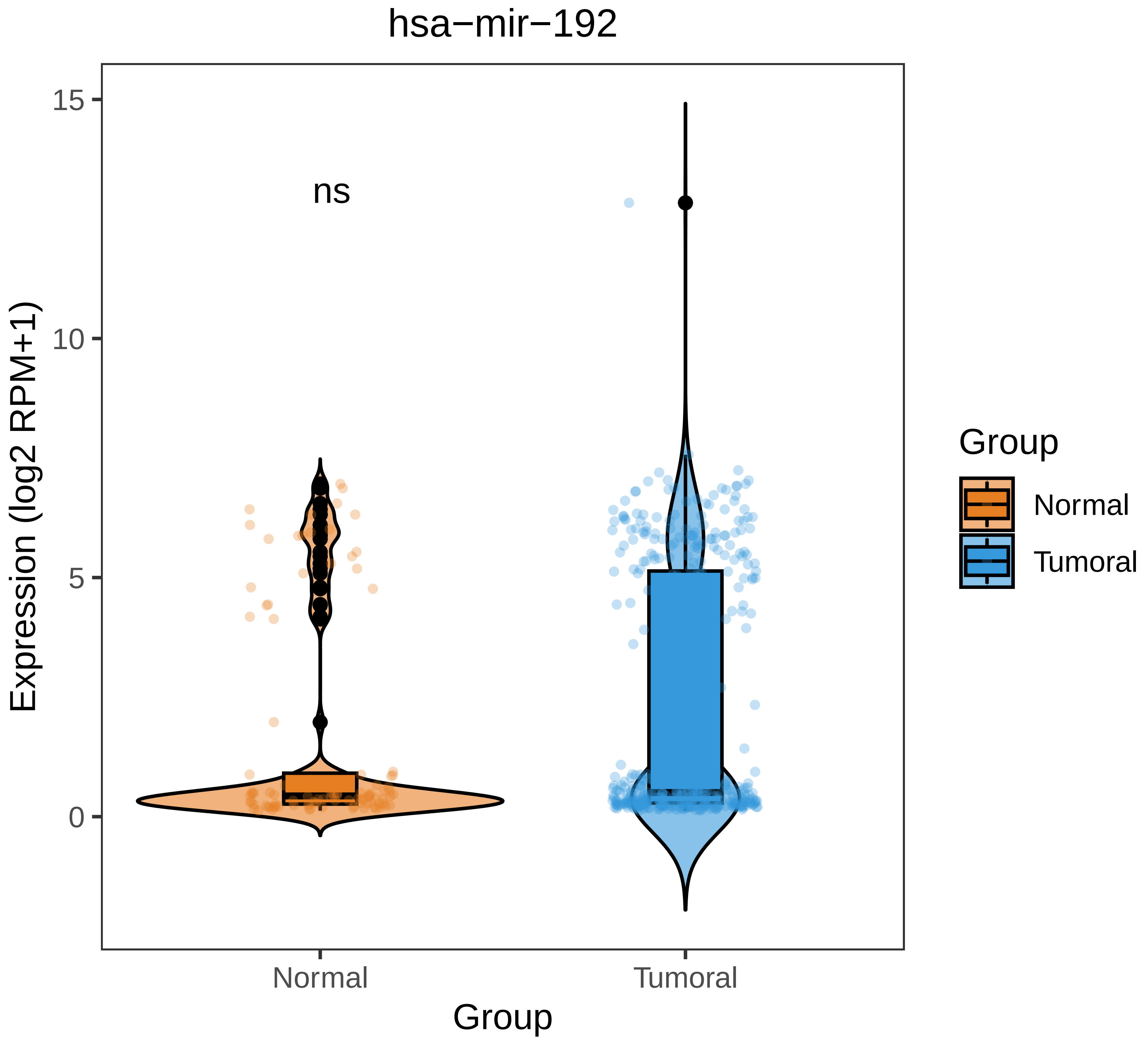
hsa−mir−192
ns
Expression (log2 RPM+1)
15
10
5
0
Normal
Tumoral
Group
Group
Normal
Tumoral

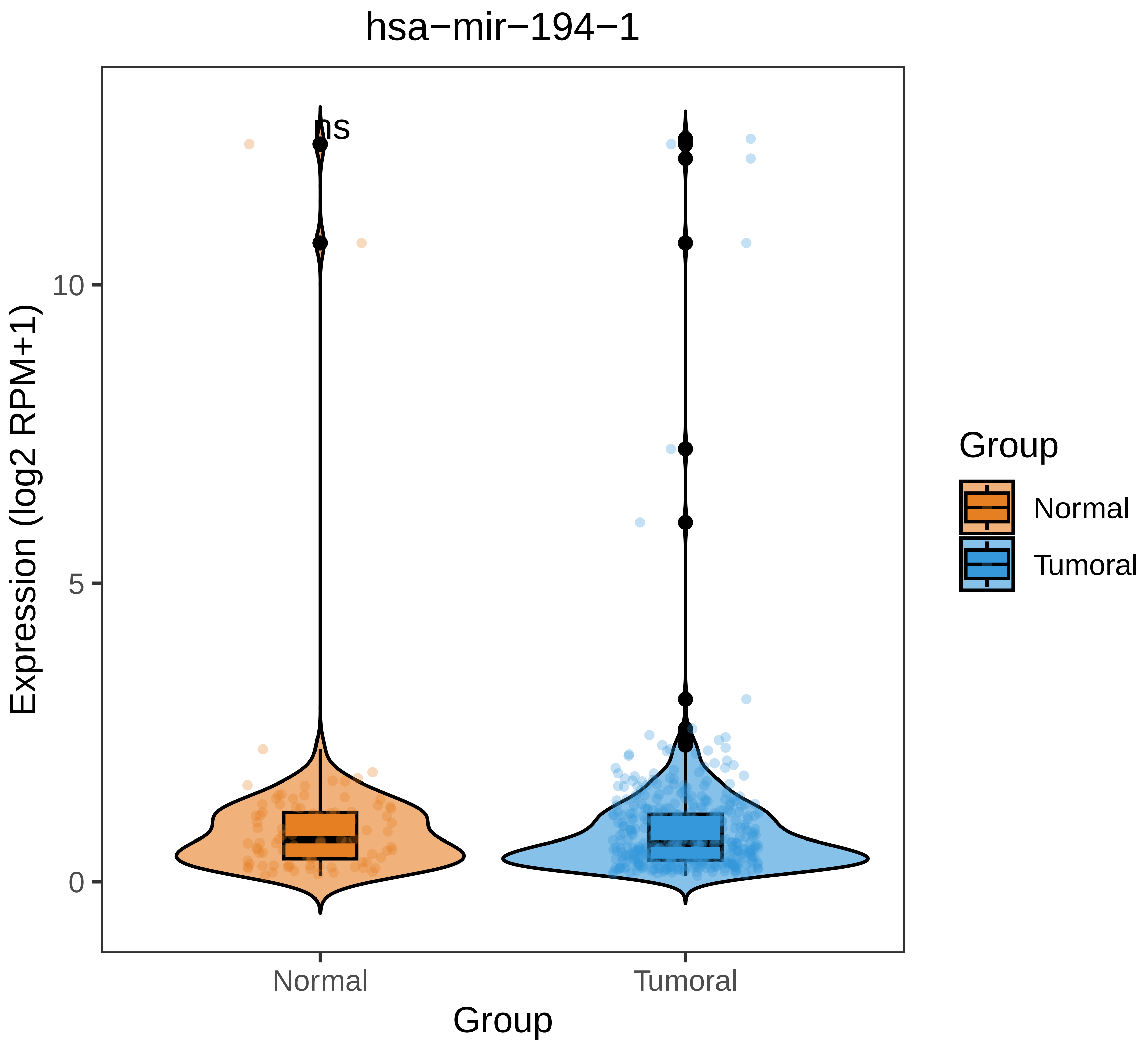

hsa−mir−194−1
ns
10
5
0
Expression (log2 RPM+1)
Normal
Tumoral
Group
Group
Normal
Tumoral

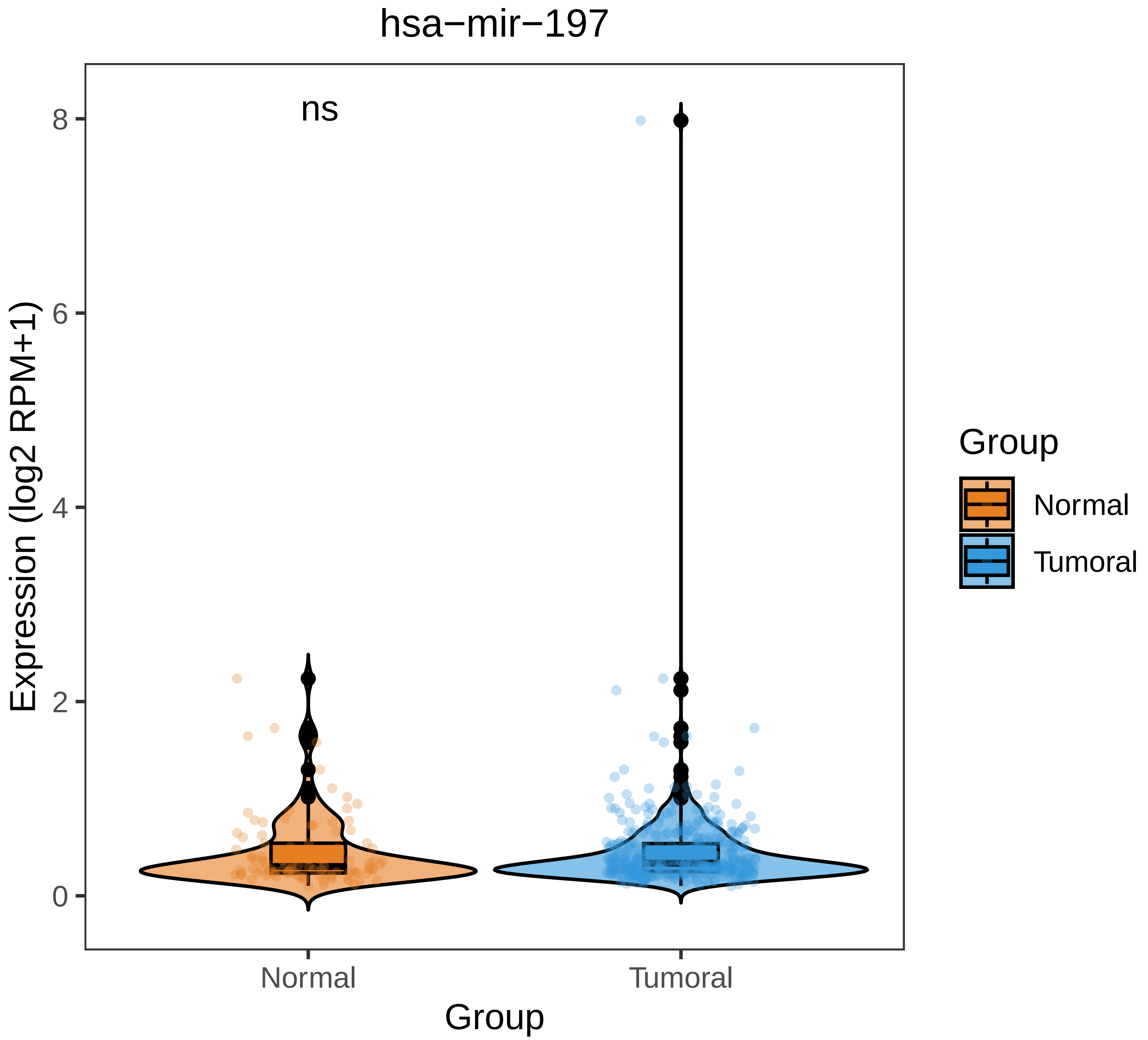

hsa−mir−197
ns
8
6
4
2
0
Expression (log2 RPM+1)
Normal
Tumoral
Group
Group
Normal
Tumoral

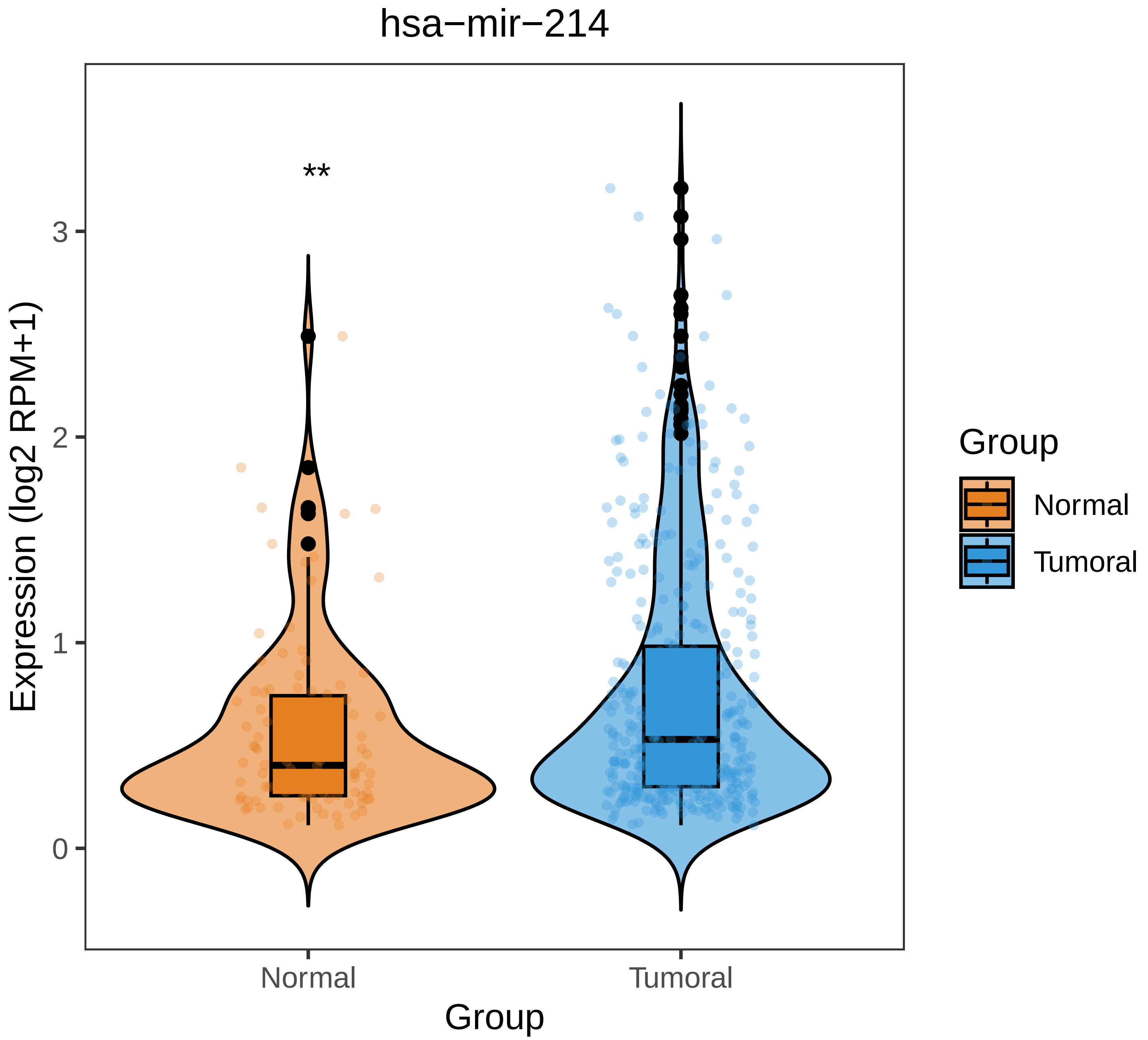
hsa−mir−214
**
Expression (log2 RPM+1)
0
1
2
3
Normal
Tumoral
Group
Group
Normal
Tumoral

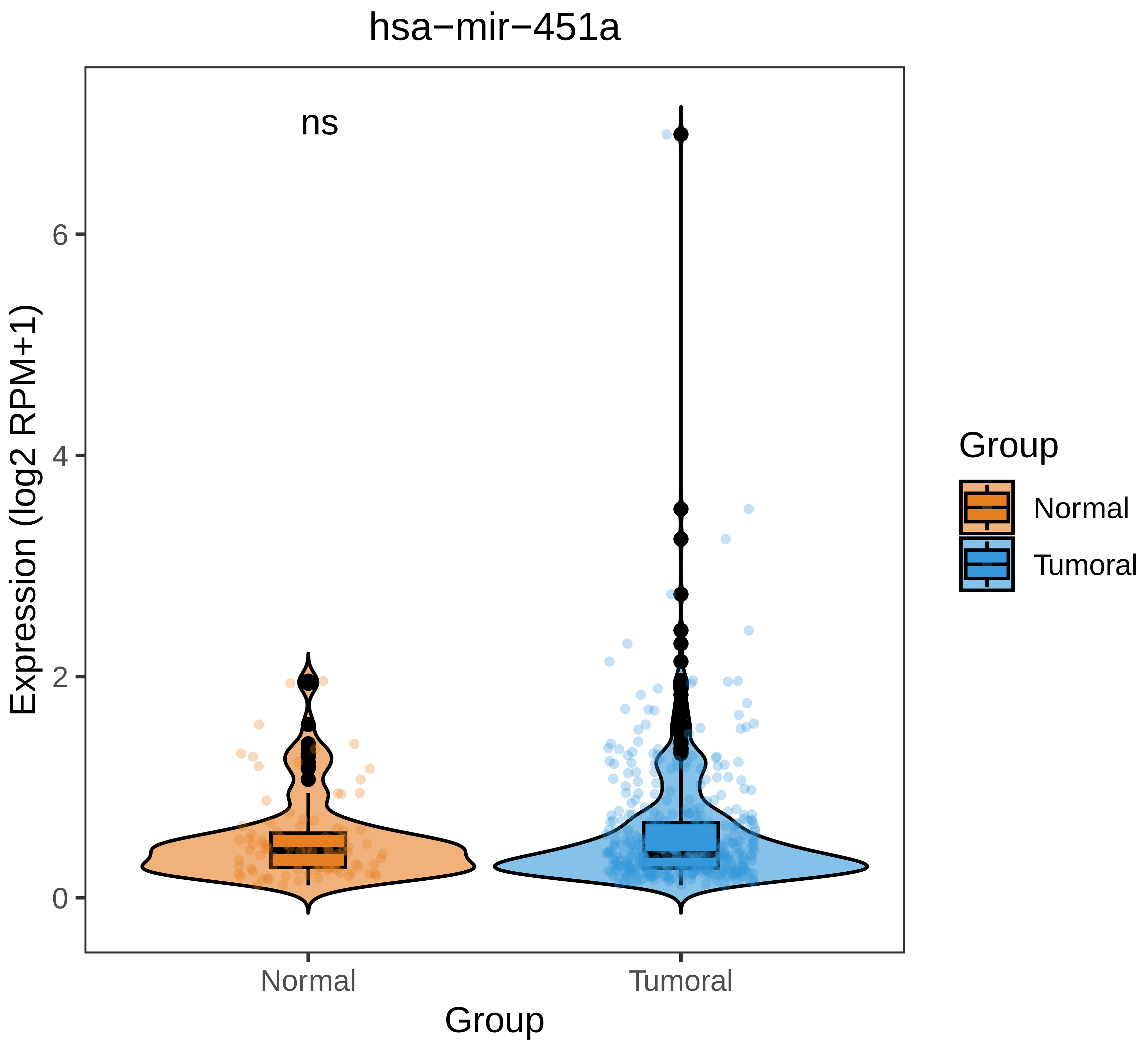

hsa−mir−451a
ns
Expression (log2 RPM+1)
0
2
4
6
Normal
Tumoral
Group
Group
Normal
Tumoral

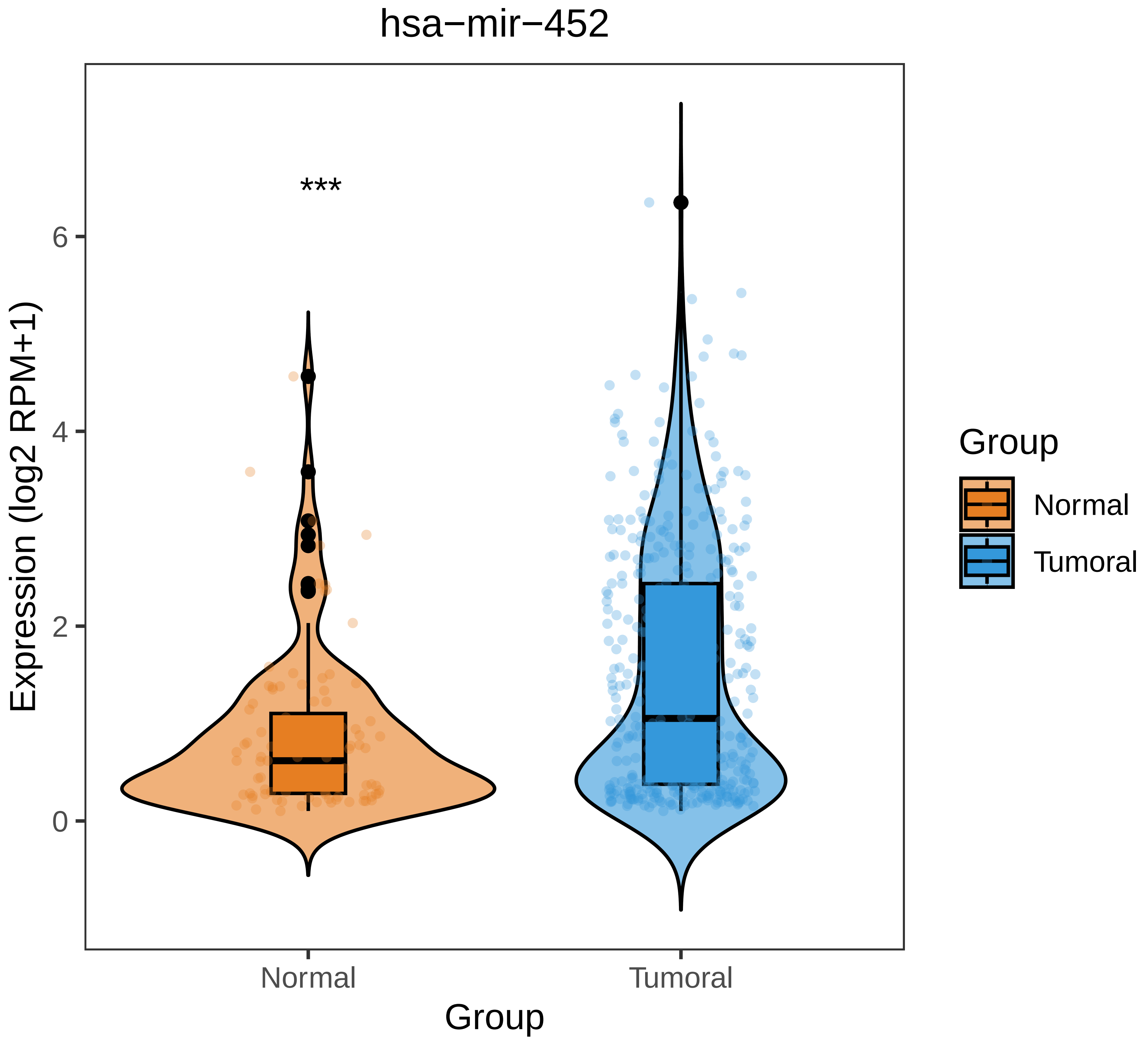
hsa−mir−452
***
Expression (log2 RPM+1)
0
2
4
6
Normal
Tumoral
Group
Group
Normal
Tumoral

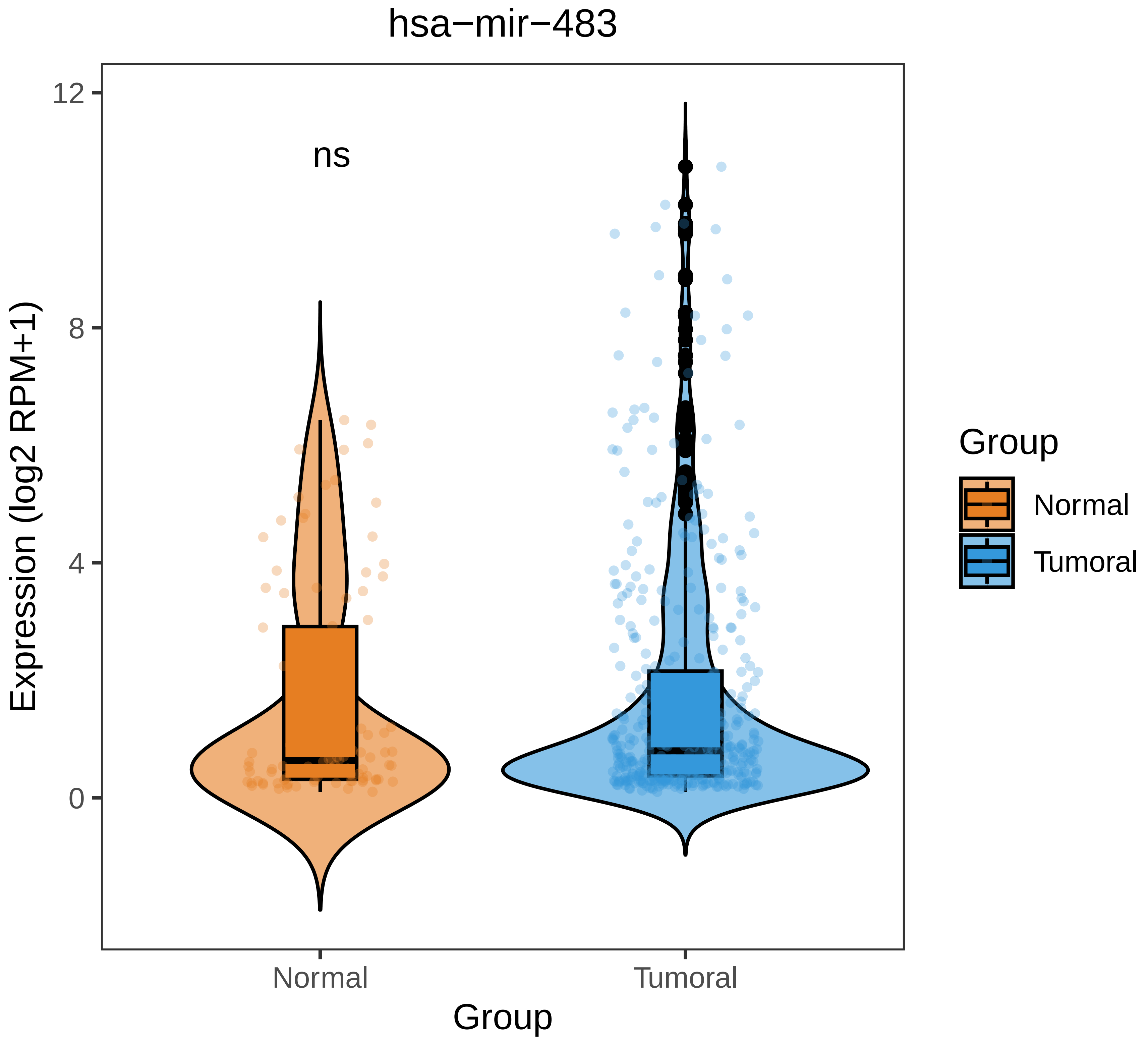

hsa−mir−483
ns
Expression (log2 RPM+1)
12
8
4
0
Normal
Tumoral
Group
Group
Normal
Tumoral

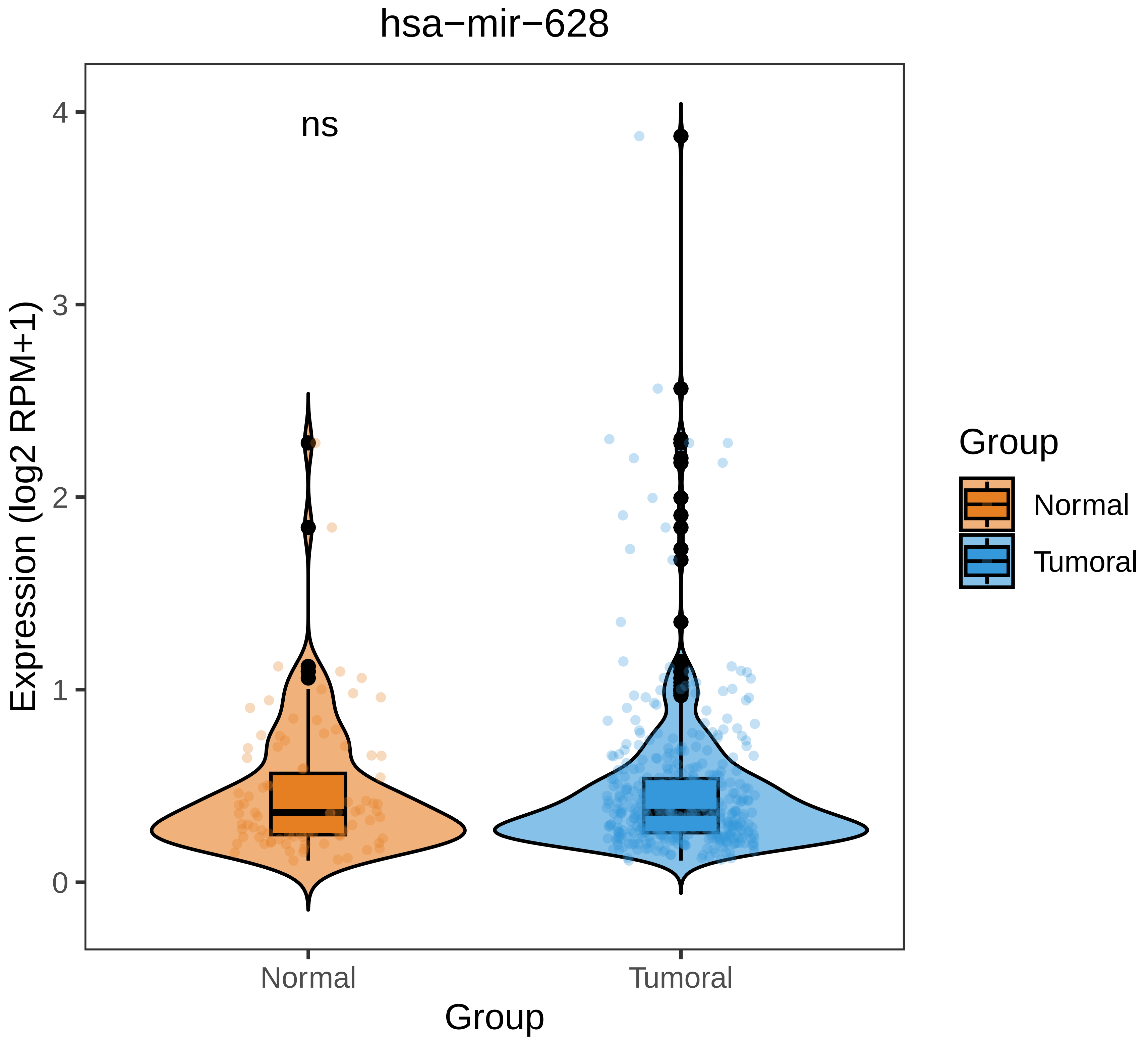
hsa−mir−628
ns
Expression (log2 RPM+1)
0
1
2
3
4
Normal
Tumoral
Group
Group
Normal
Tumoral

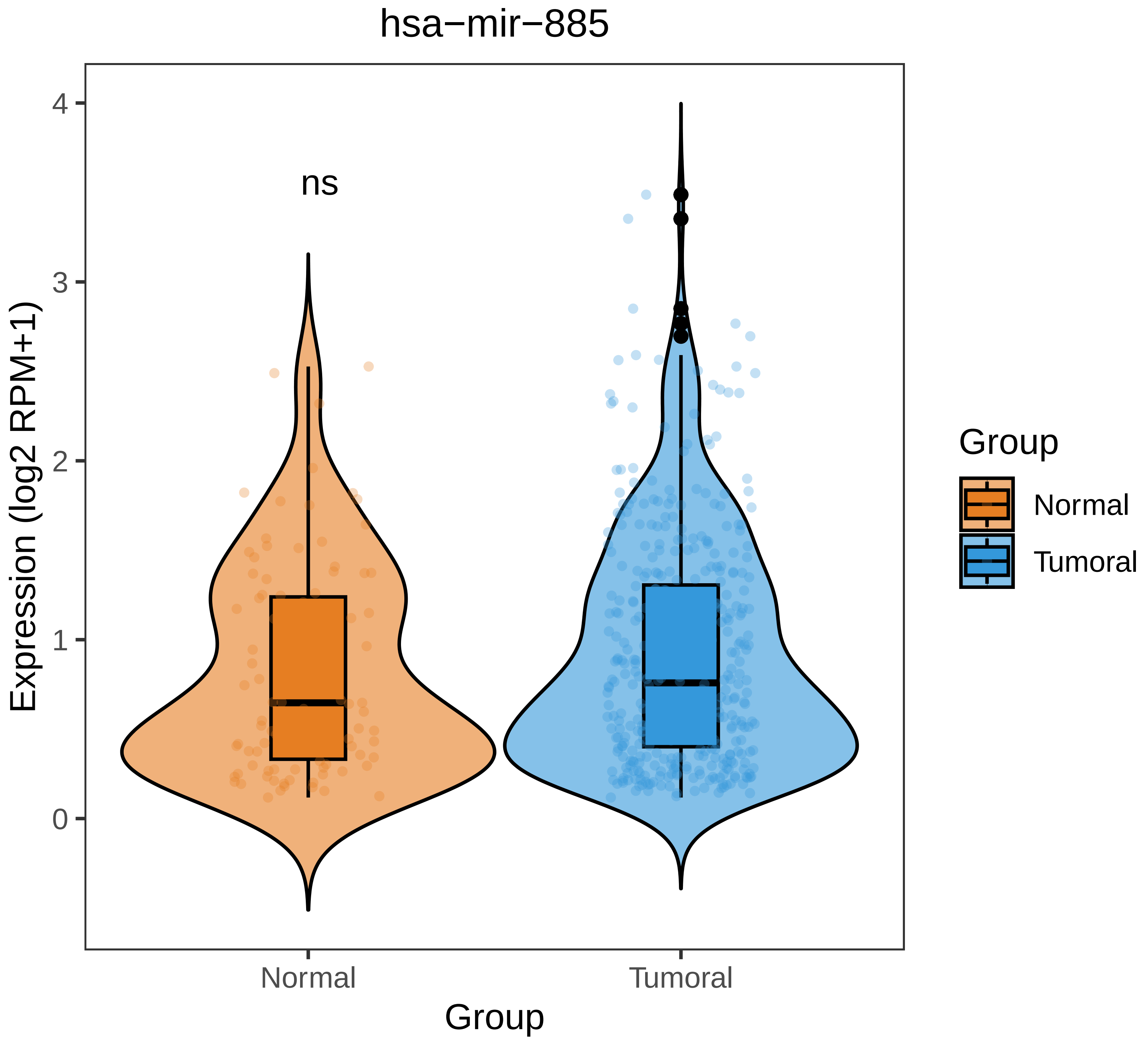

hsa−mir−885
ns
Expression (log2 RPM+1)
0
1
2
3
4
Normal
Tumoral
Group
Group
Normal
Tumoral

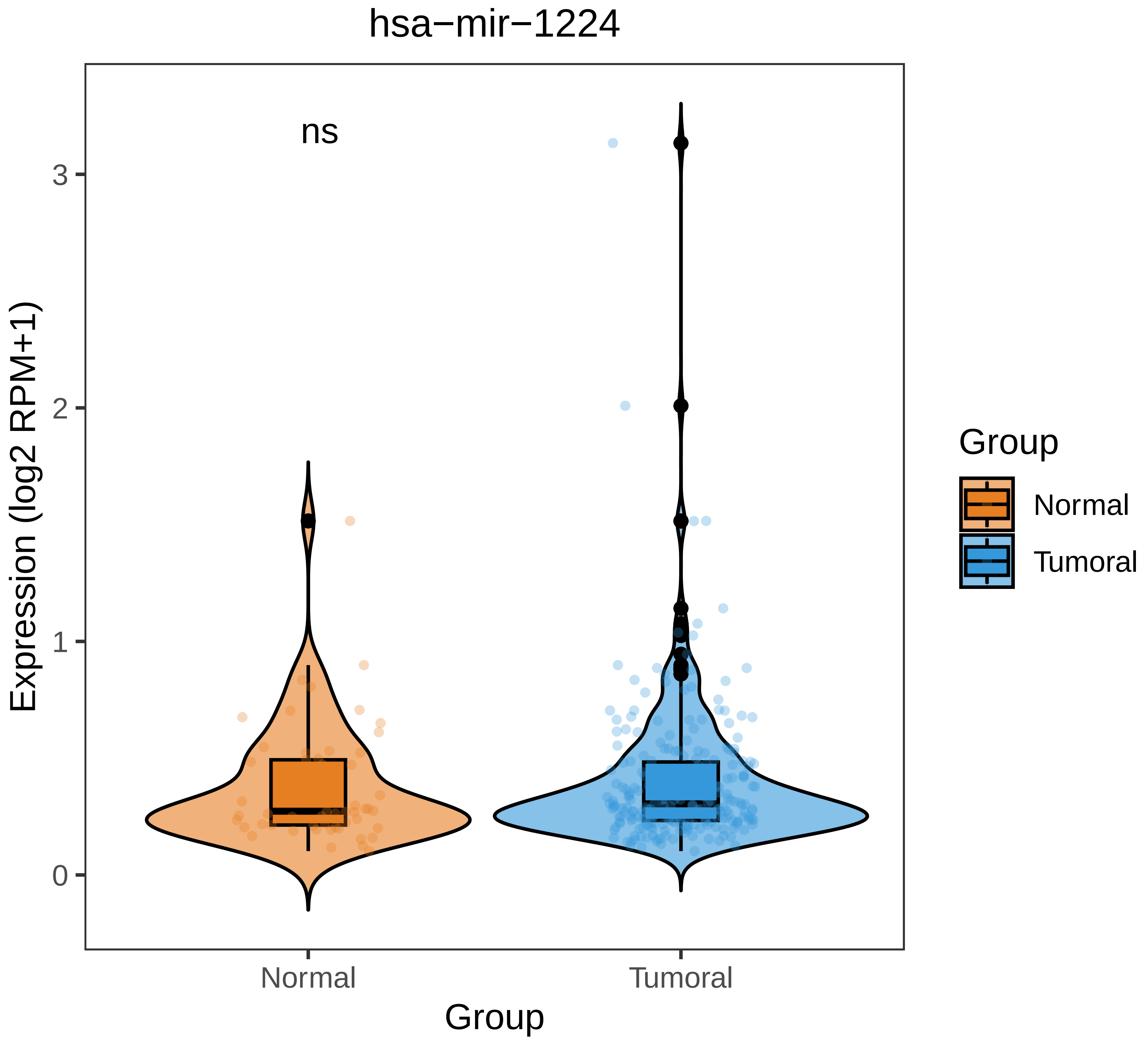
hsa−mir−1224
ns
Expression (log2 RPM+1)
0
1
2
3
Normal
Tumoral
Group
Group
Normal
Tumoral

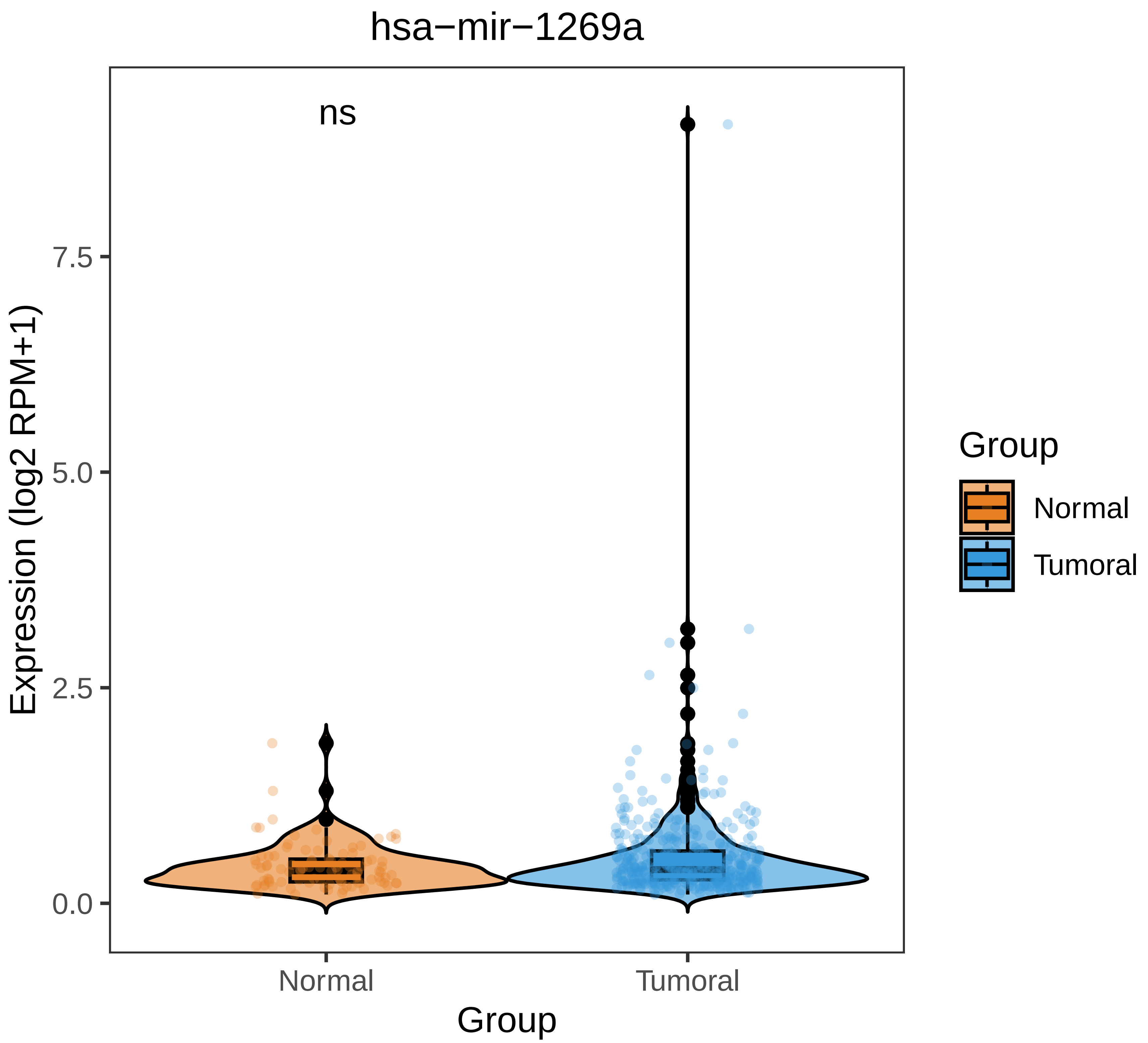
hsa−mir−1269a
ns
Expression (log2 RPM+1)
7.5
5.0
2.5
0.0
Normal
Tumoral
Group
Group
Normal
Tumoral

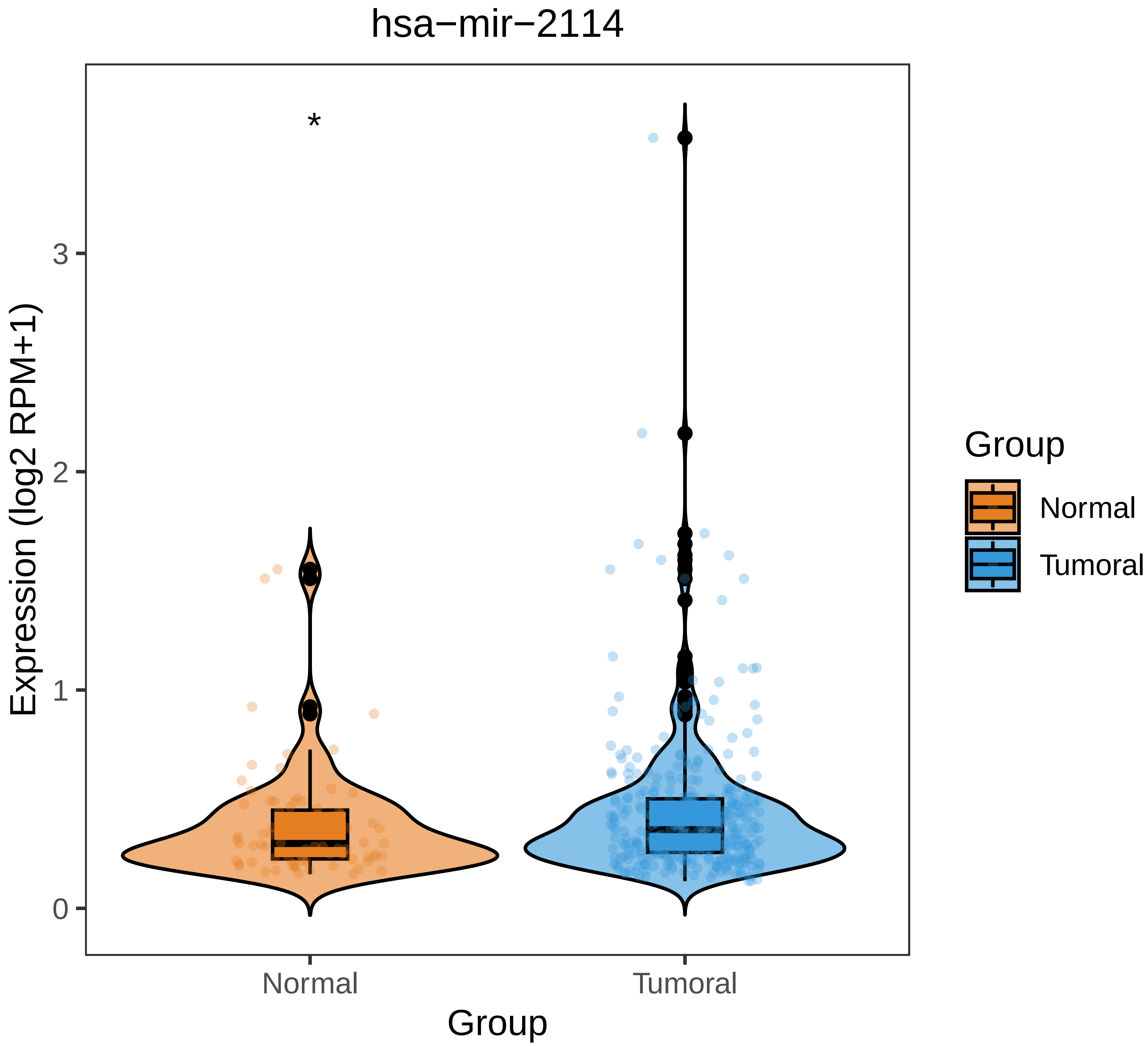

hsa−mir−2114
*
Expression (log2 RPM+1)
0
1
2
3
Normal
Tumoral
Group
Group
Normal
Tumoral
