# Supplementary material for: Optimization of extracellular vesicle extraction from hepatic tissue interstitial fluid and analysis of their ncRNA expression profiles
Source: PLoS One. 2026 Aug 3;21(8):e0355303. doi: 10.1371/journal.pone.0355303 (PMC13432105; doi:10.1371/journal.pone.0355303)
Supplement: S4 File — (PDF) [file pone.0355303.s005.pdf]

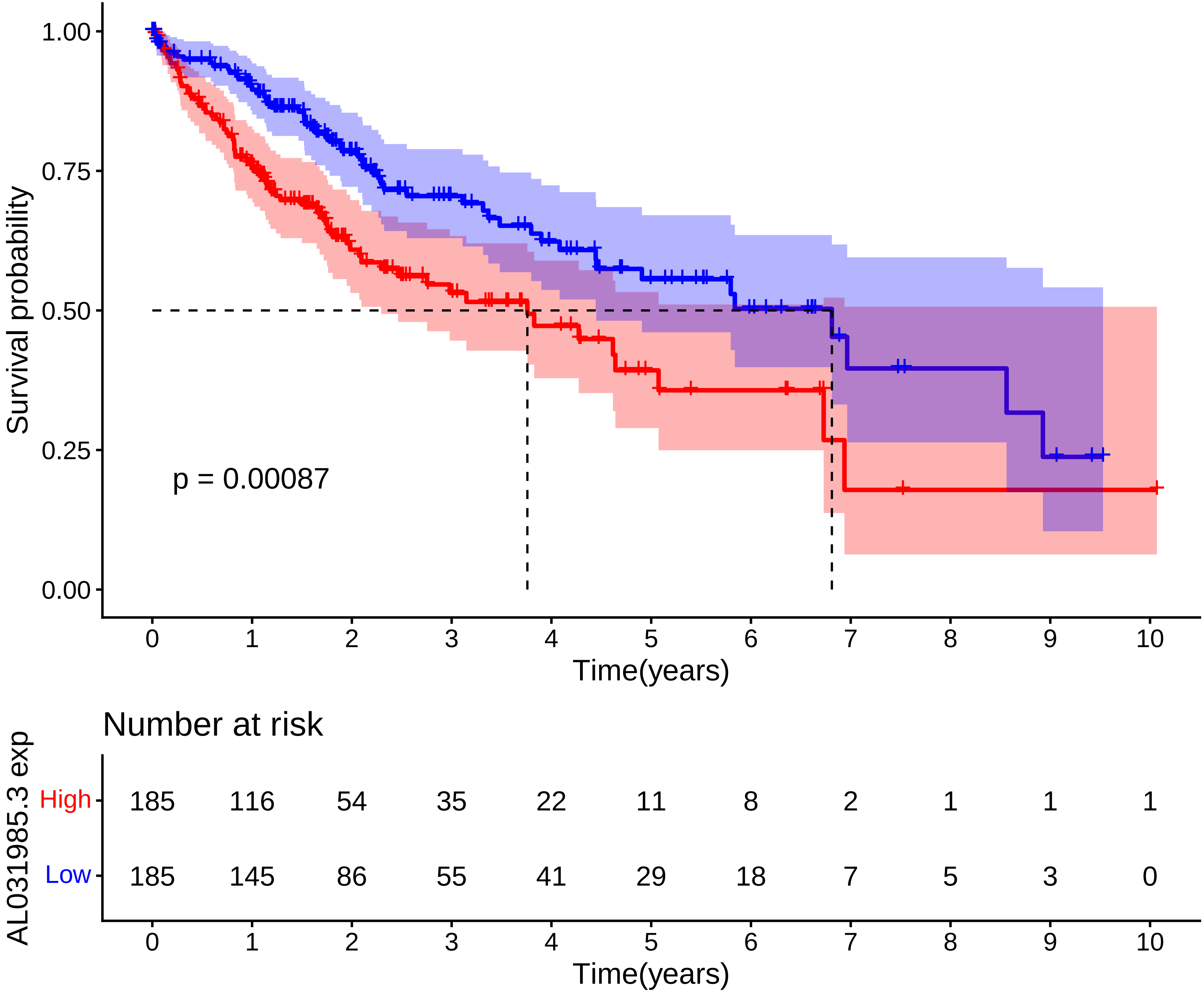

Survival probability
1.00
0.75
0.50
0.25
0.00
p = 0.00087
0
1
2
3
4
5
6
7
8
9
10
Time(years)
Number at risk
AL031985.3 exp
High
Low
185 116 54 35 22 11 8 2 1 1 1
185 145 86 55 41 29 18 7 5 3 0
Time(years)

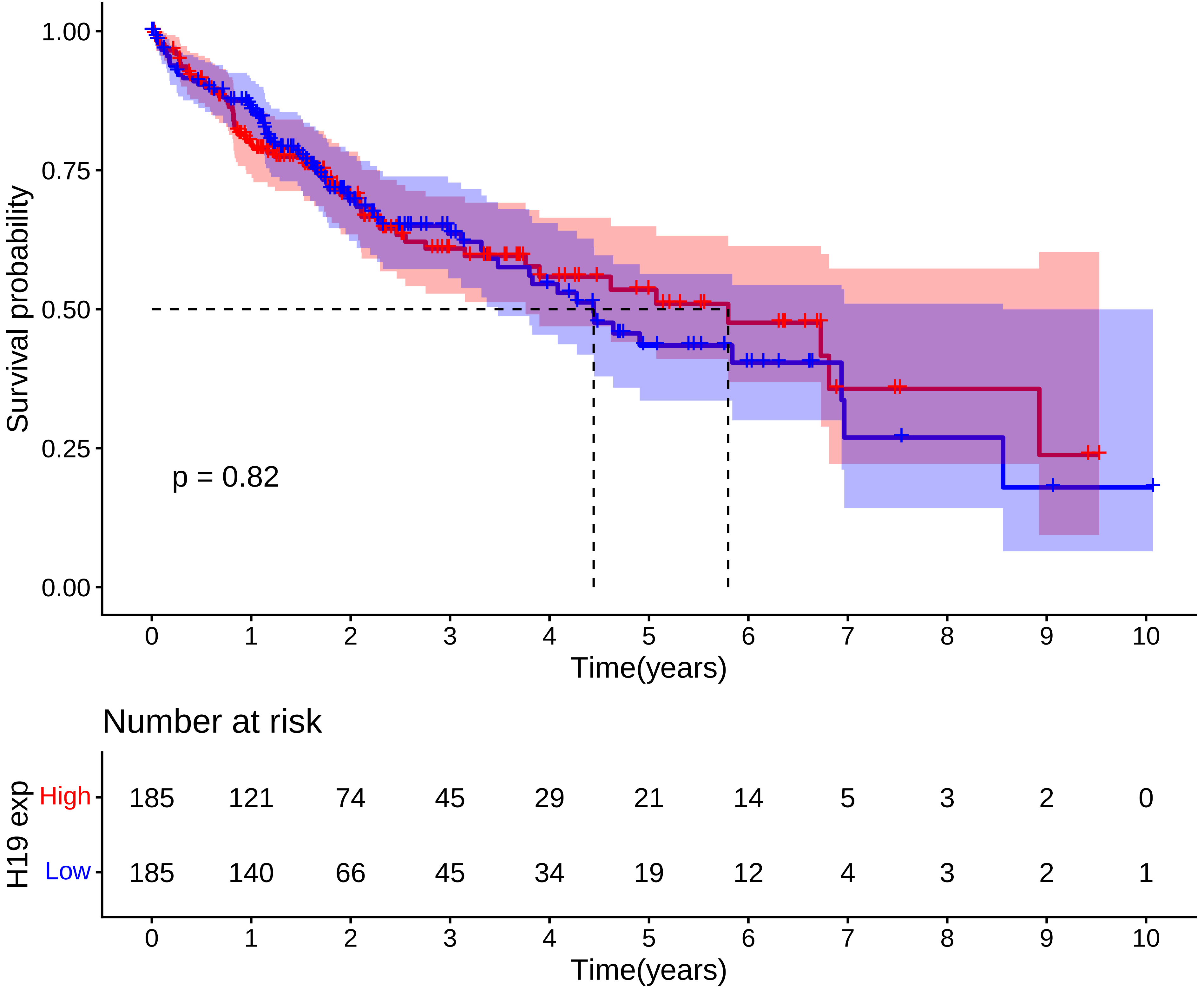

1.00
0.75
0.50
0.25
0.00
Survival probability
p = 0.82
0 1 2 3 4 5 6 7 8 9 10
Time(years)
Number at risk
H19 exp
High 185 121 74 45 29 21 14 5 3 2 0
Low 185 140 66 45 34 19 12 4 3 2 1
0 1 2 3 4 5 6 7 8 9 10
Time(years)

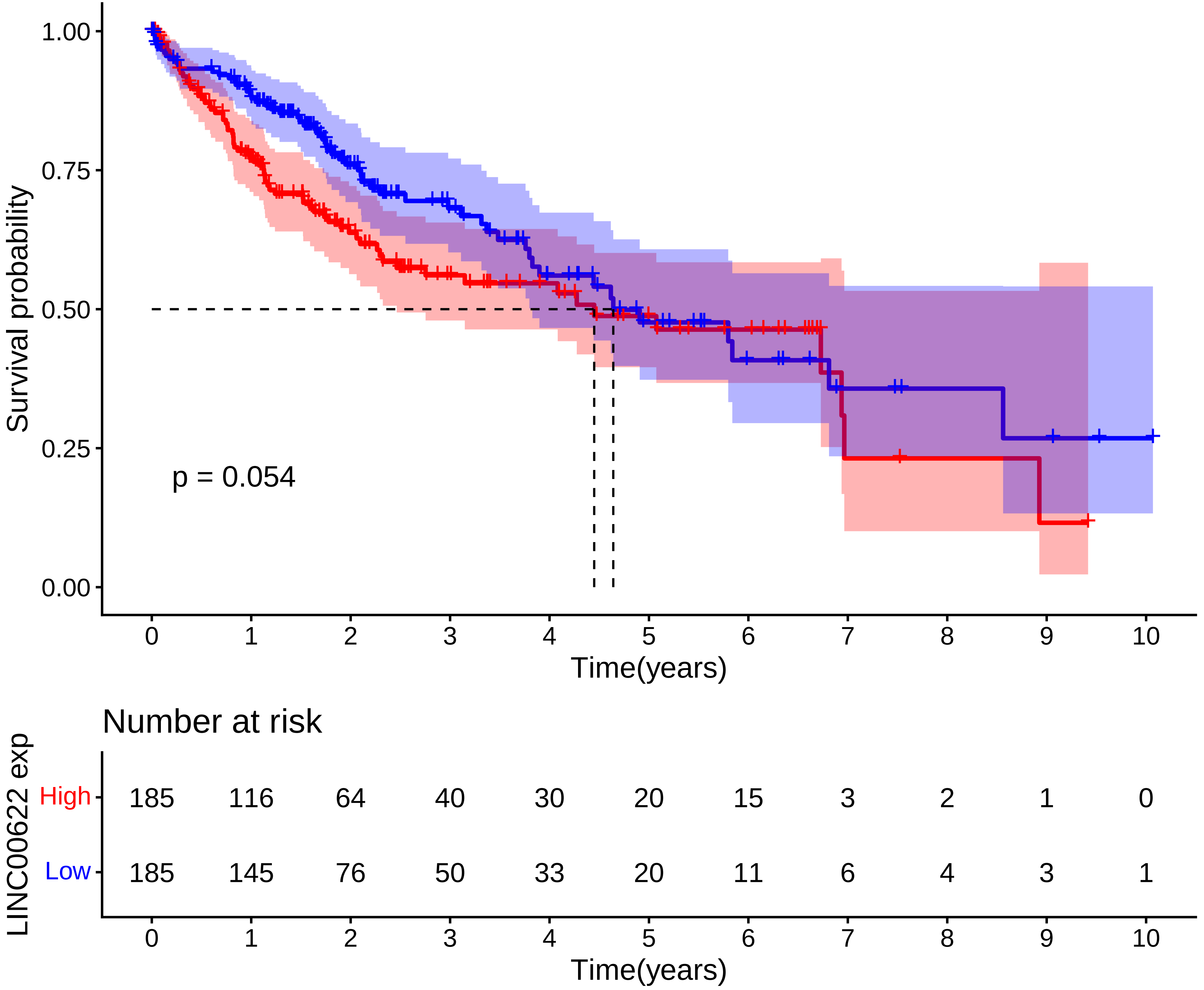
Survival probability
1.00
0.75
0.50
0.25
0.00
p = 0.054
0 1 2 3 4 5 6 7 8 9 10
Time(years)
Number at risk
LINC00622 exp
High 185 116 64 40 30 20 15 3 2 1 0
Low 185 145 76 50 33 20 11 6 4 3 1
0 1 2 3 4 5 6 7 8 9 10
Time(years)

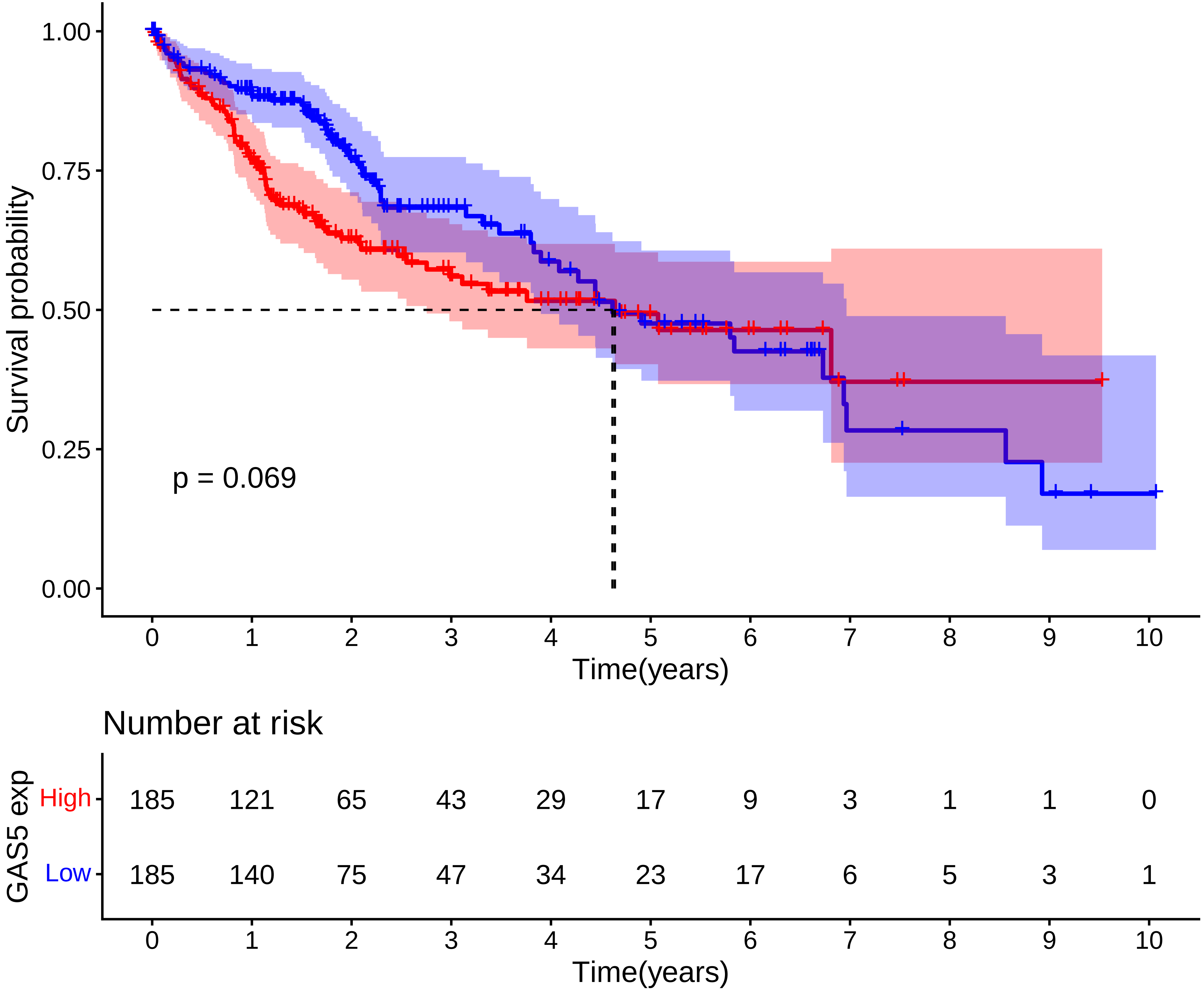

Survival probability
1.00
0.75
0.50
0.25
0.00
p = 0.069
0 1 2 3 4 5 6 7 8 9 10
Time(years)
Number at risk
GAS5 exp
High 185 121 65 43 29 17 9 3 1 1 0
Low 185 140 75 47 34 23 17 6 5 3 1
0 1 2 3 4 5 6 7 8 9 10
Time(years)

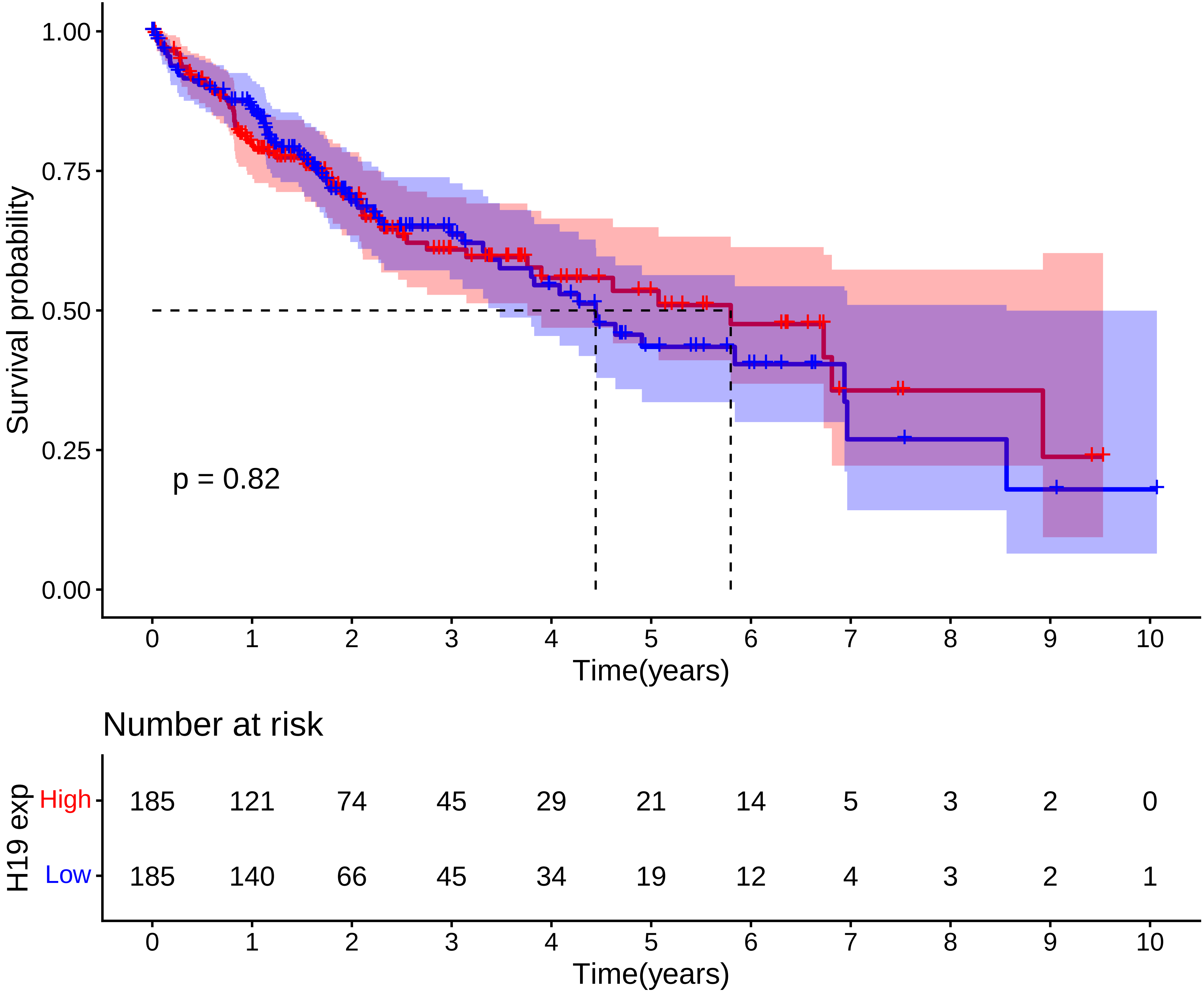

Survival probability
1.00
0.75
0.50
0.25
0.00
p = 0.82
0
1
2
3
4
5
6
7
8
9
10
Time(years)
Number at risk
H19 exp
High
185 121 74 45 29 21 14 5 3 2 0
Low
185 140 66 45 34 19 12 4 3 2 1
0
1
2
3
4
5
6
7
8
9
10
Time(years)

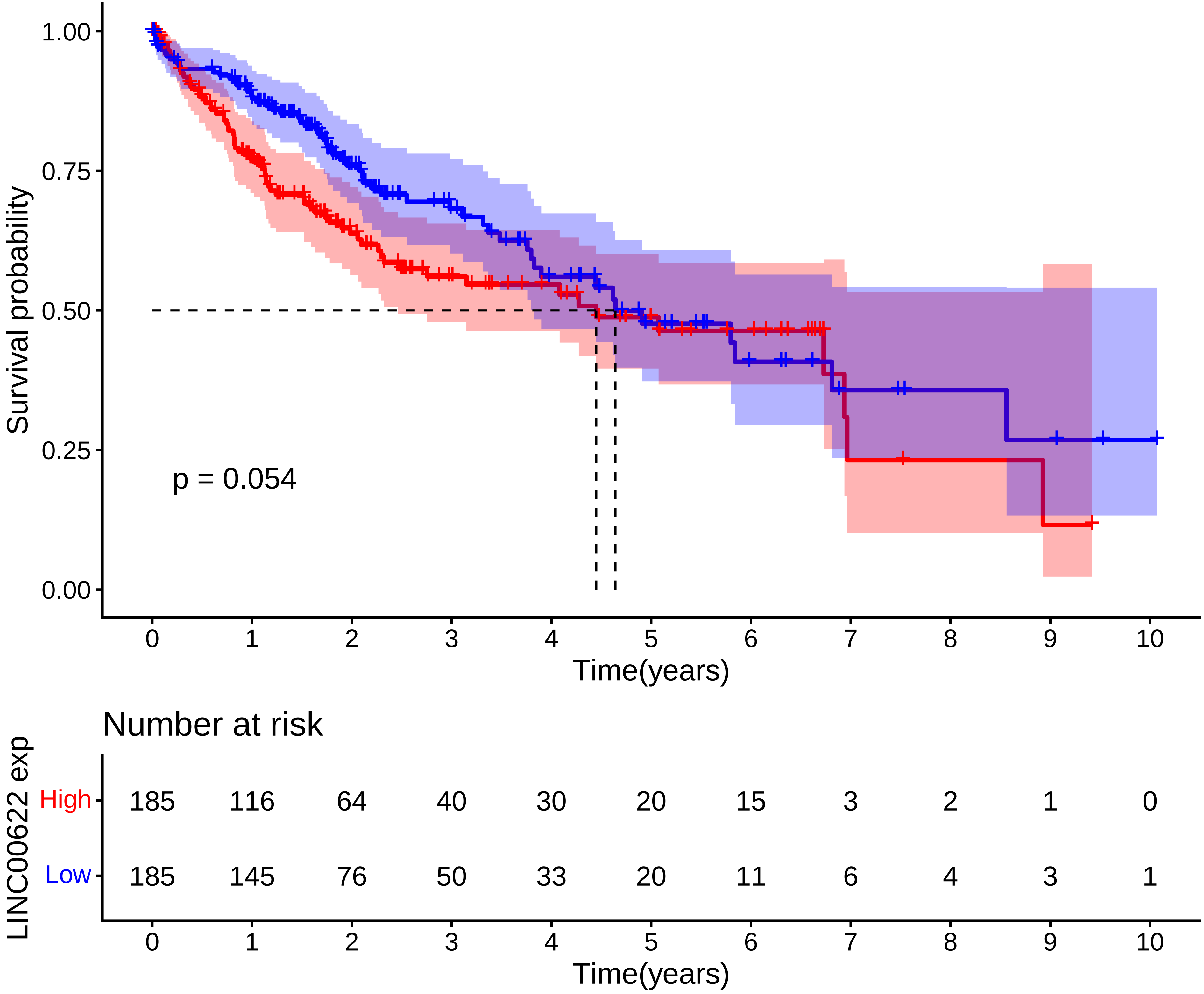
Survival probability
1.00
0.75
0.50
0.25
0.00
p = 0.054
0 1 2 3 4 5 6 7 8 9 10
Time(years)
Number at risk
LINC00622 exp
High 185 116 64 40 30 20 15 3 2 1 0
Low 185 145 76 50 33 20 11 6 4 3 1
0 1 2 3 4 5 6 7 8 9 10
Time(years)

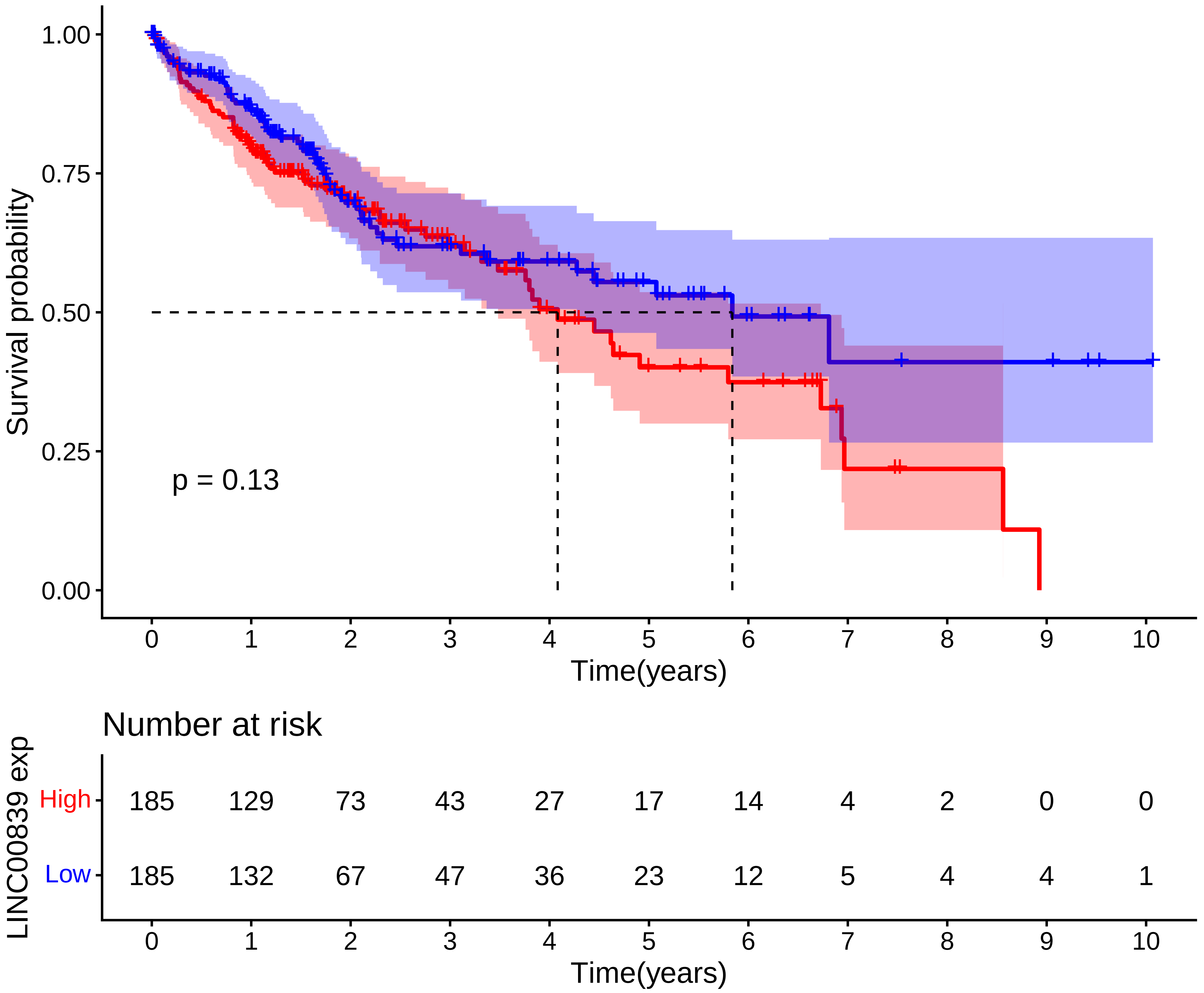

Survival probability
1.00
0.75
0.50
0.25
0.00
p = 0.13
0 1 2 3 4 5 6 7 8 9 10
Time(years)
Number at risk
LINC00839 exp
High 185 129 73 43 27 17 14 4 2 0 0
Low 185 132 67 47 36 23 12 5 4 4 1
0 1 2 3 4 5 6 7 8 9 10
Time(years)

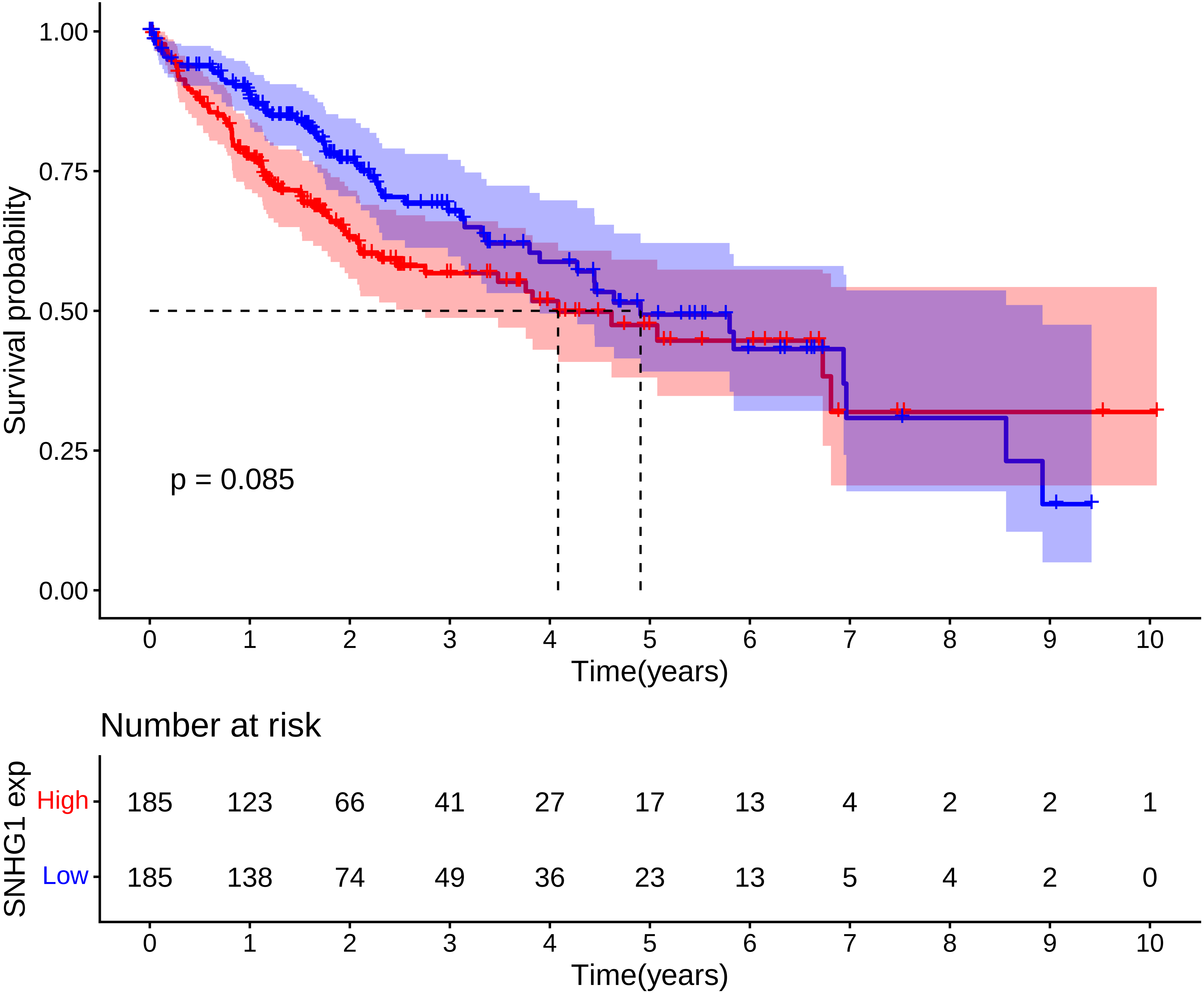
Survival probability
1.00
0.75
0.50
0.25
0.00
p = 0.085
0 1 2 3 4 5 6 7 8 9 10
Time(years)
Number at risk
SNHG1 exp
High 185 123 66 41 27 17 13 4 2 2 1
Low 185 138 74 49 36 23 13 5 4 2 0
0 1 2 3 4 5 6 7 8 9 10
Time(years)

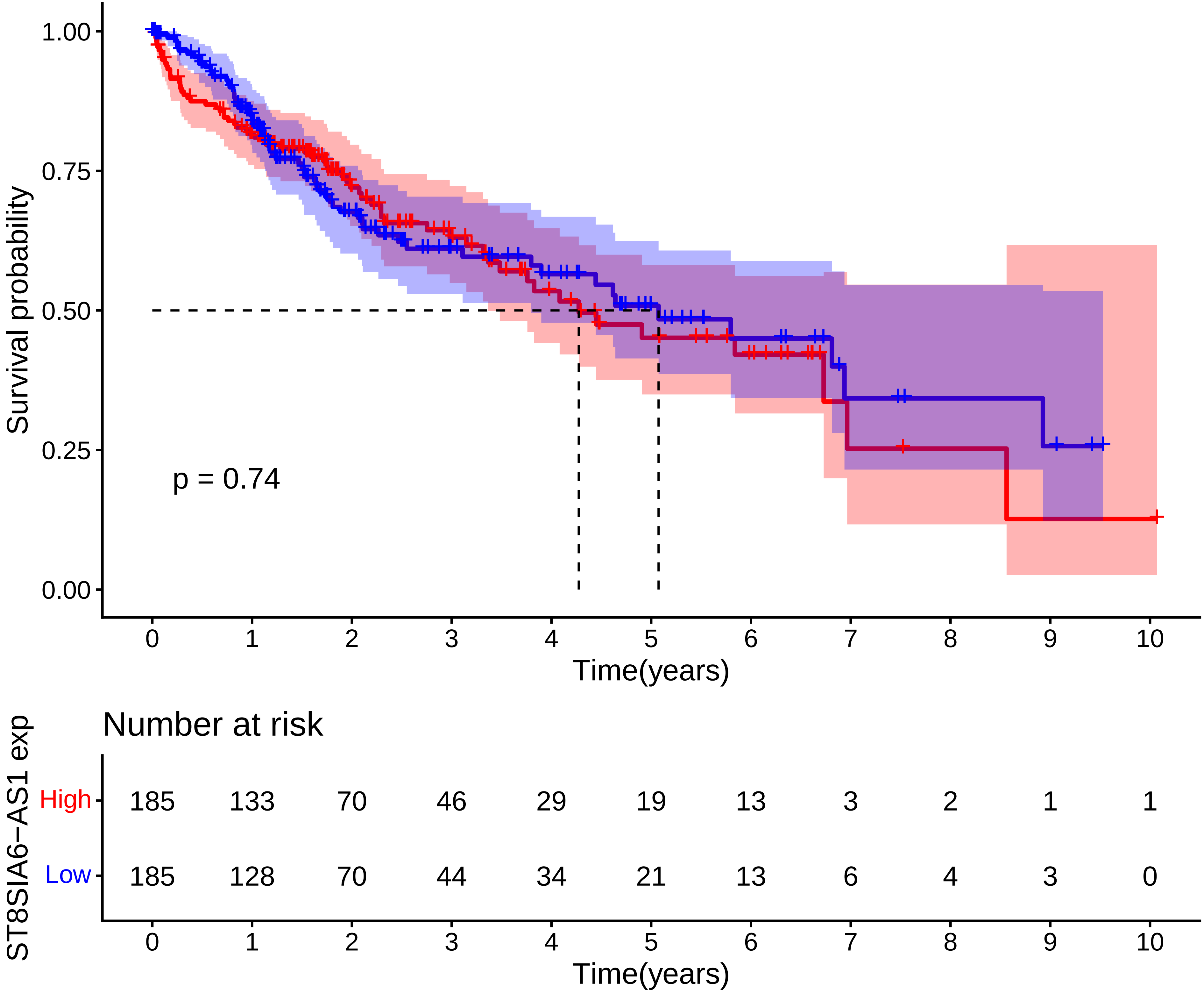

Survival probability
1.00
0.75
0.50
0.25
0.00
p = 0.74
0
1
2
3
4
5
6
7
8
9
10
Time(years)
Number at risk
ST8SIA6−AS1 exp
High
185 133 70 46 29 19 13 3 2 1 1
Low
185 128 70 44 34 21 13 6 4 3 0
Time(years)

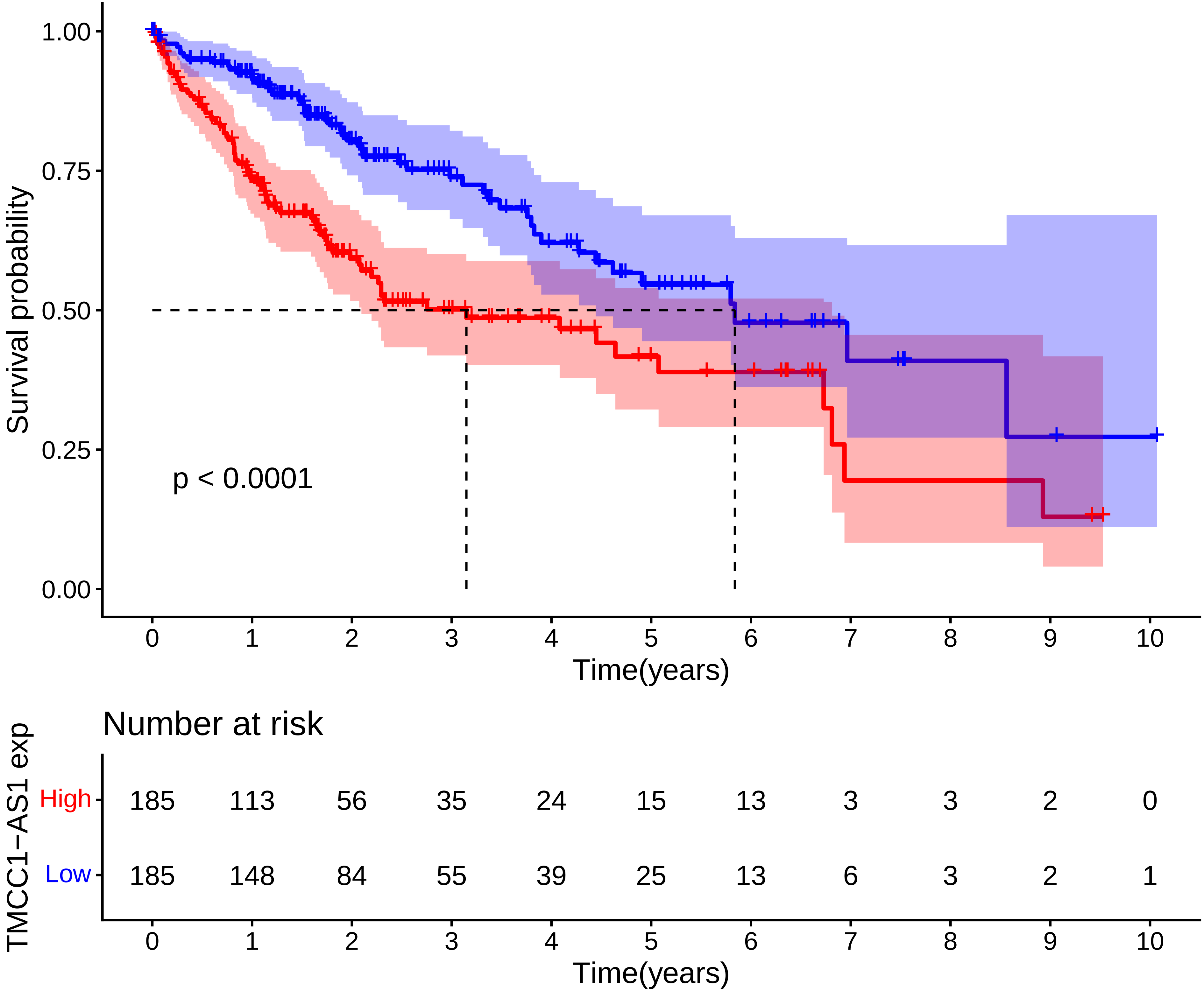

Survival probability
1.00
0.75
0.50
0.25
0.00
p < 0.0001
0 1 2 3 4 5 6 7 8 9 10
Time(years)
Number at risk
TMCC1−AS1 exp
High 185 113 56 35 24 15 13 3 3 2 0
Low 185 148 84 55 39 25 13 6 3 2 1
0 1 2 3 4 5 6 7 8 9 10
Time(years)

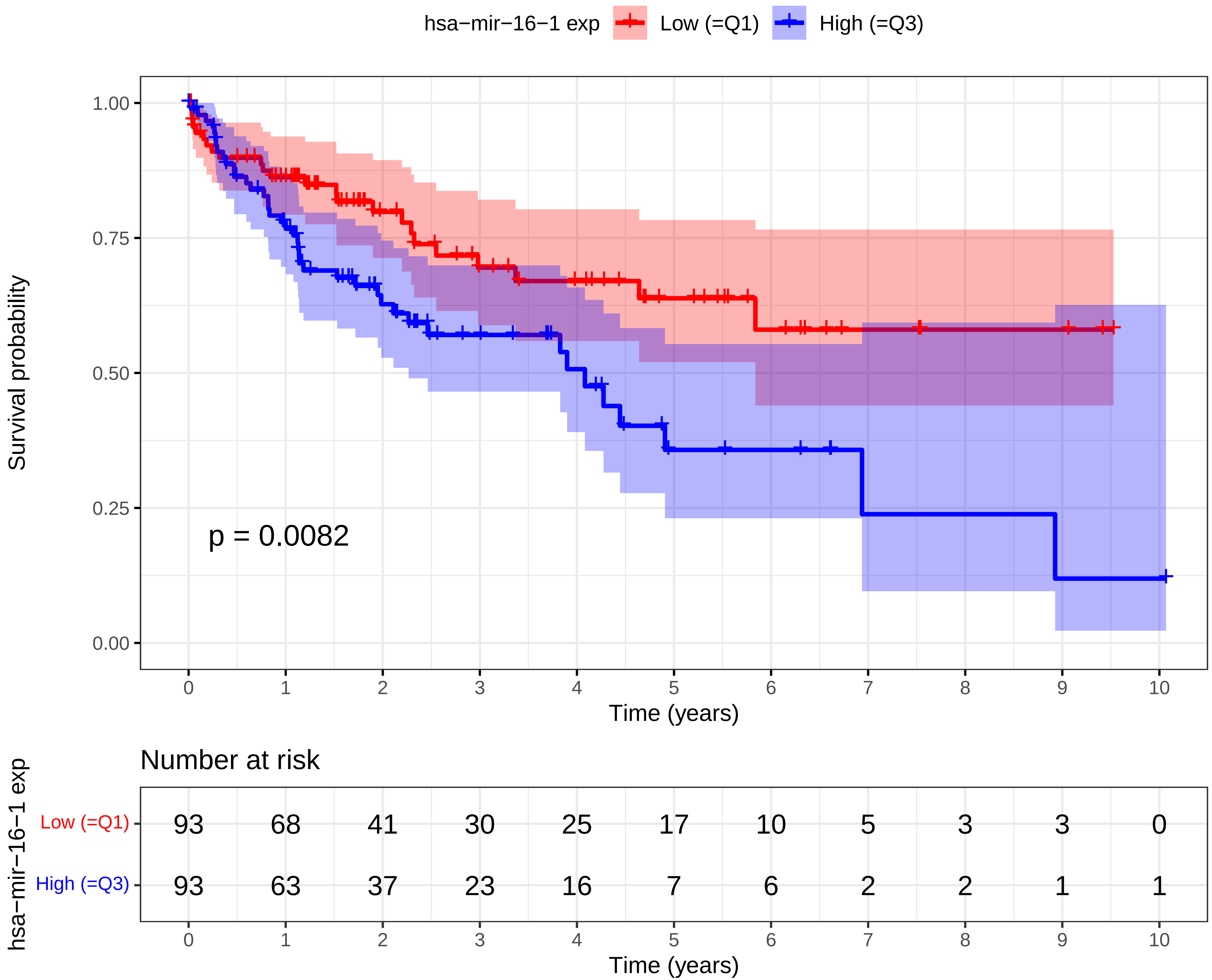
hsa−mir−16−1 exp
Low (=Q1)
High (=Q3)
Survival probability
1.00
0.75
0.50
0.25
0.00
p = 0.0082
0 1 2 3 4 5 6 7 8 9 10
Time (years)
Number at risk
hsa−mir−16−1 exp
Low (=Q1) 93 68 41 30 25 17 10 5 3 3 0
High (=Q3) 93 63 37 23 16 7 6 2 2 1 1
0 1 2 3 4 5 6 7 8 9 10
Time (years)

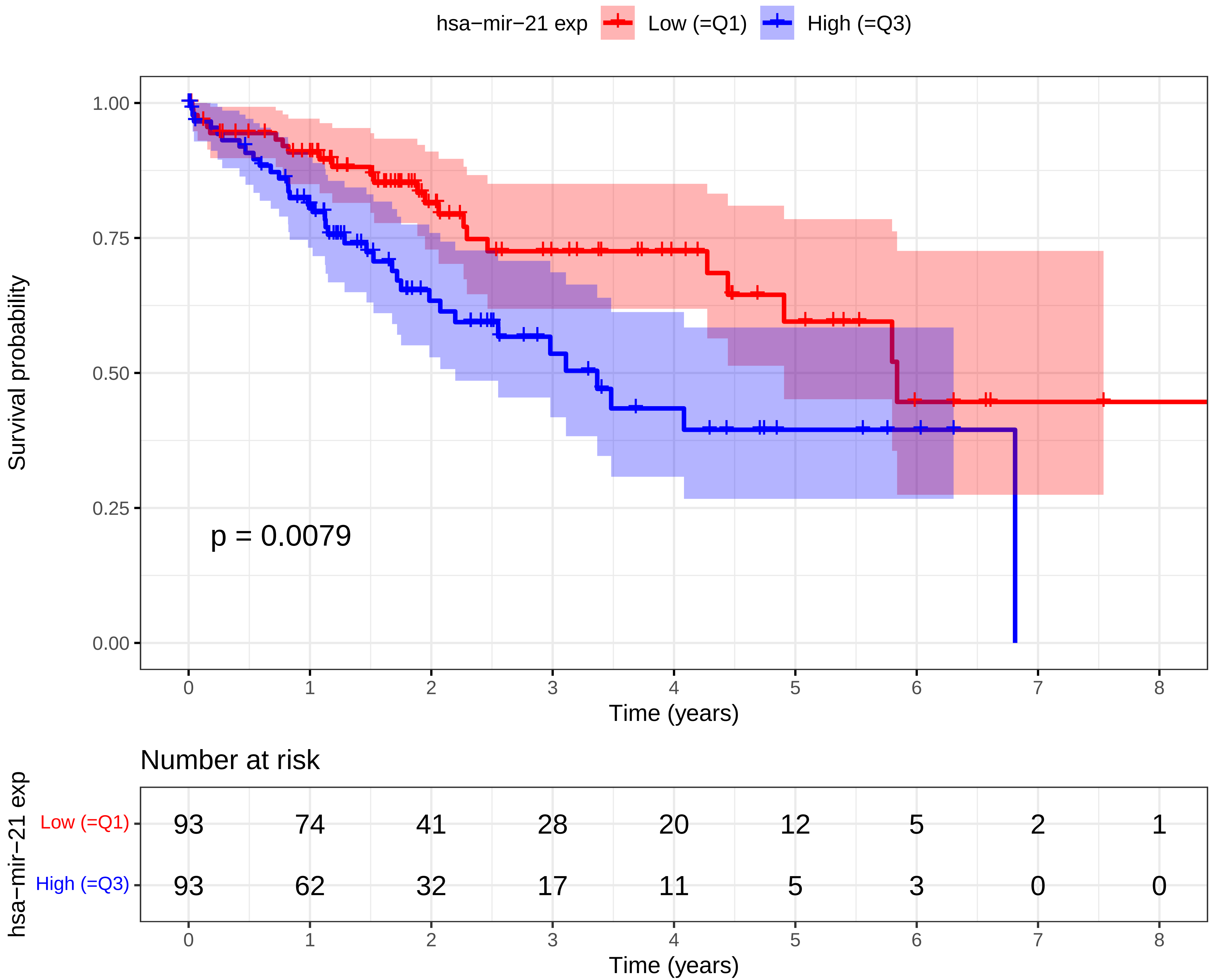
hsa−mir−21 exp
Low (=Q1)
High (=Q3)
Survival probability
1.00
0.75
0.50
0.25
0.00
p = 0.0079
0
1
2
3
4
5
6
7
8
Time (years)
Number at risk
hsa−mir−21 exp
Low (=Q1) 93 74 41 28 20 12 5 2 1
High (=Q3) 93 62 32 17 11 5 3 0 0
Time (years)

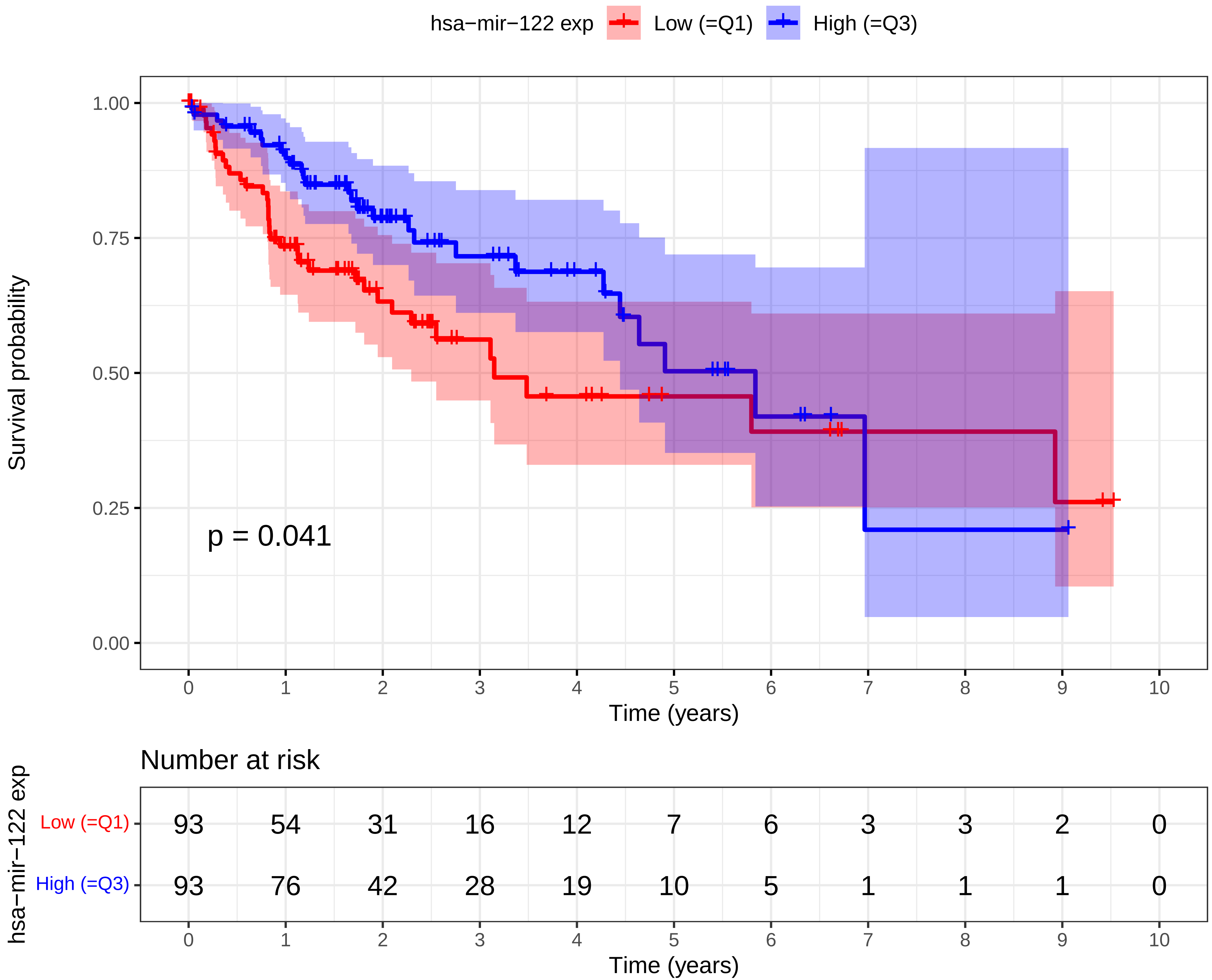

hsa−mir−122 exp
Low (=Q1)
High (=Q3)
1.00
0.75
0.50
0.25
0.00
Survival probability
p = 0.041
0 1 2 3 4 5 6 7 8 9 10
Time (years)
Number at risk
hsa−mir−122 exp
Low (=Q1) 93 54 31 16 12 7 6 3 3 2 0
High (=Q3) 93 76 42 28 19 10 5 1 1 1 0
0 1 2 3 4 5 6 7 8 9 10
Time (years)

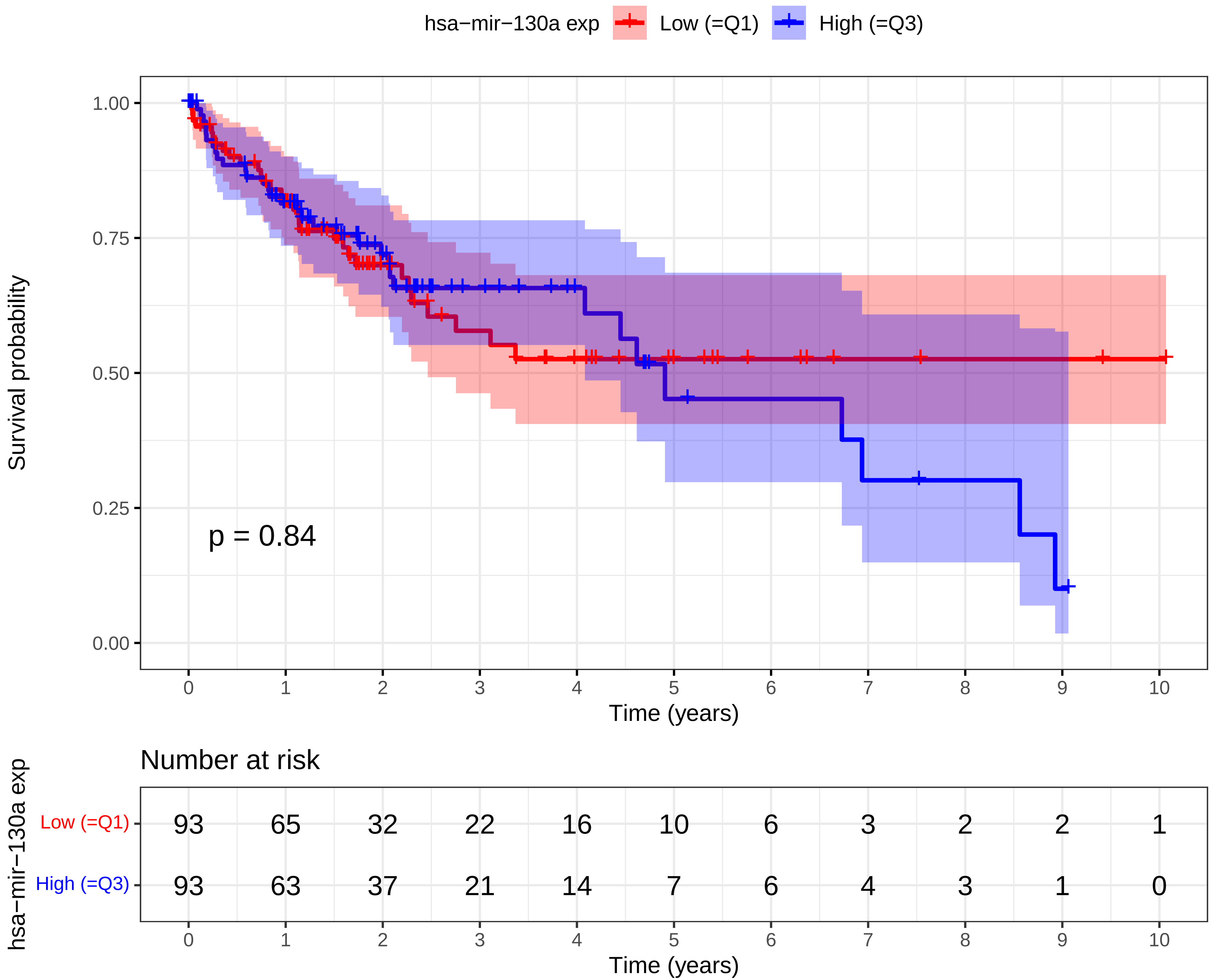
hsa−mir−130a exp
Low (=Q1)
High (=Q3)
Survival probability
1.00
0.75
0.50
0.25
0.00
p = 0.84
Time (years)
0 1 2 3 4 5 6 7 8 9 10
Number at risk
hsa−mir−130a exp
Low (=Q1) 93 65 32 22 16 10 6 3 2 2 1
High (=Q3) 93 63 37 21 14 7 6 4 3 1 0
Time (years)

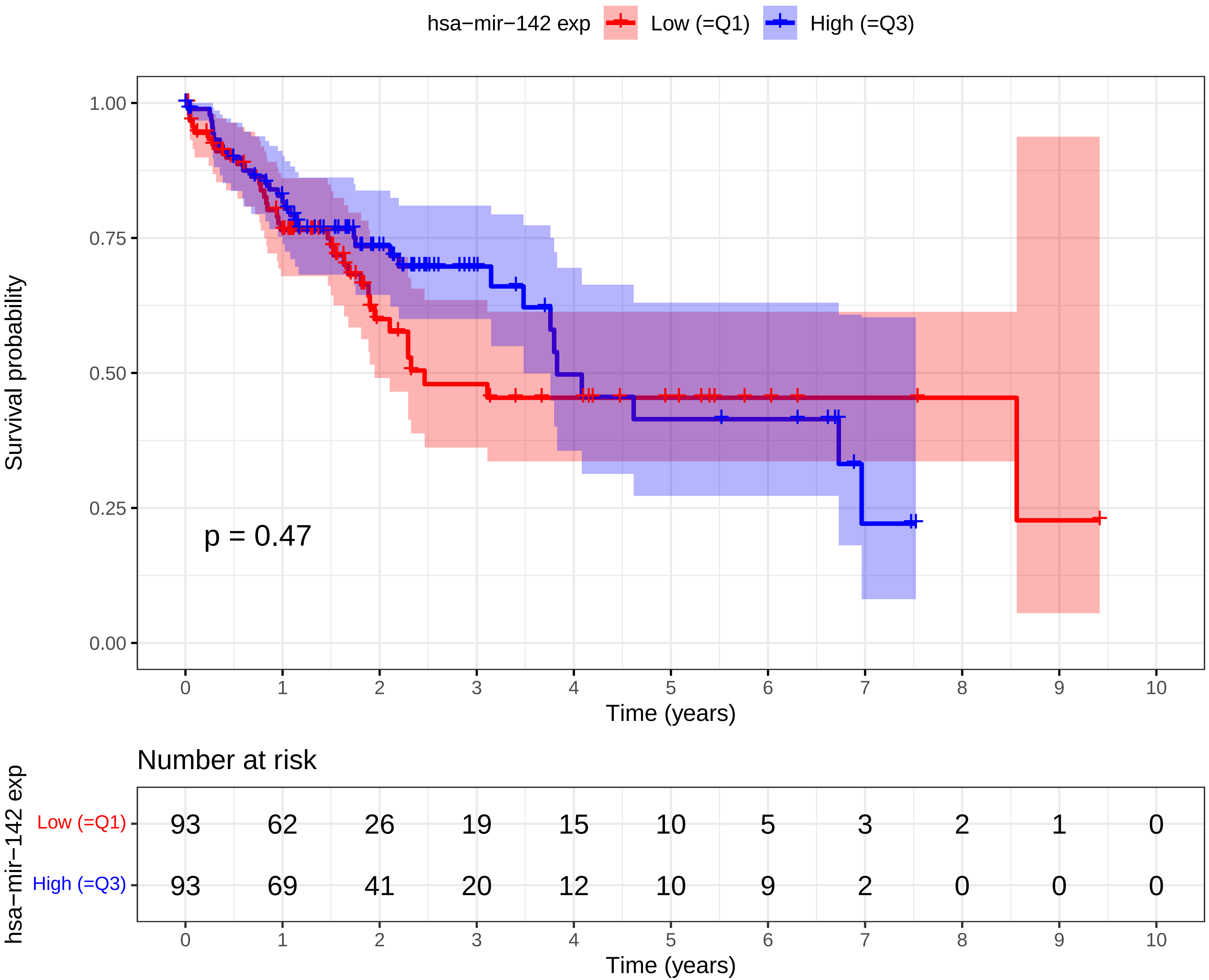
hsa−mir−142 exp
Low (=Q1)
High (=Q3)
Survival probability
1.00
0.75
0.50
0.25
0.00
p = 0.47
0
1
2
3
4
5
6
7
8
9
10
Time (years)
Number at risk
hsa−mir−142 exp
Low (=Q1)
93 62 26 19 15 10 5 3 2 1 0
High (=Q3)
93 69 41 20 12 10 9 2 0 0 0
0
1
2
3
4
5
6
7
8
9
10
Time (years)

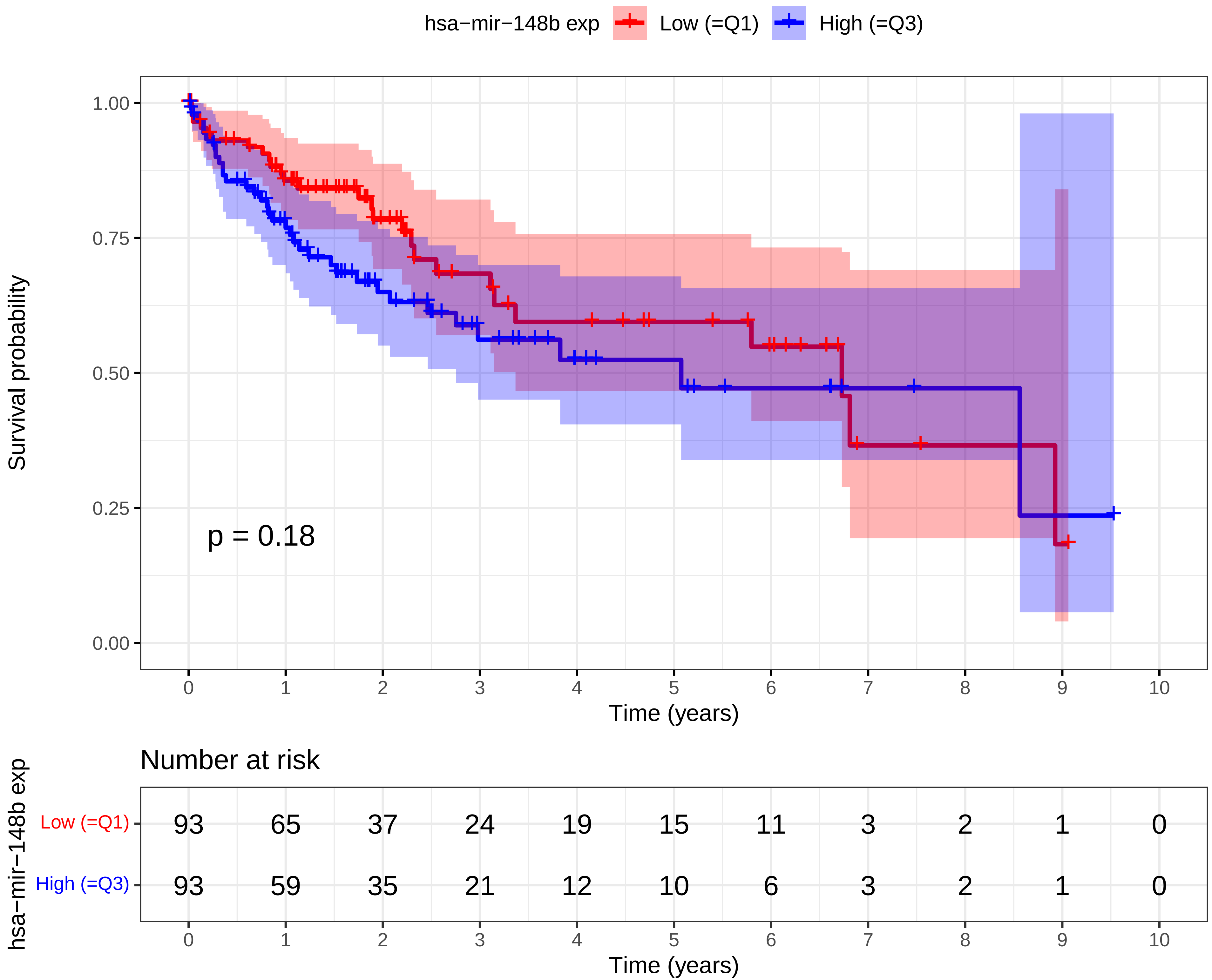
hsa−mir−148b exp
Low (=Q1)
High (=Q3)
Survival probability
1.00
0.75
0.50
0.25
0.00
p = 0.18
0 1 2 3 4 5 6 7 8 9 10
Time (years)
Number at risk
hsa−mir−148b exp
Low (=Q1) 93 65 37 24 19 15 11 3 2 1 0
High (=Q3) 93 59 35 21 12 10 6 3 2 1 0
0 1 2 3 4 5 6 7 8 9 10
Time (years)

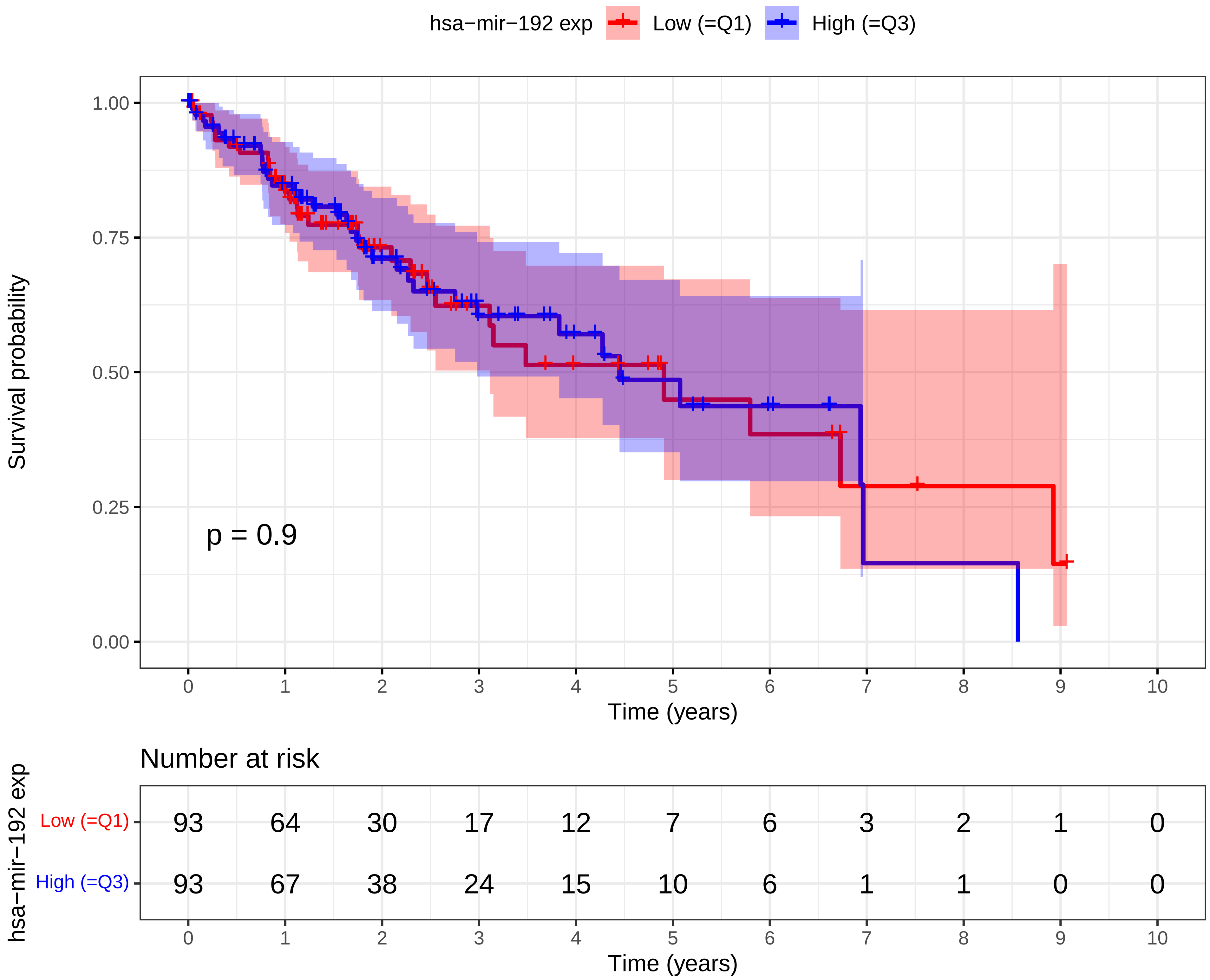
hsa−mir−192 exp
Low (=Q1)
High (=Q3)
Survival probability
1.00
0.75
0.50
0.25
0.00
p = 0.9
0 1 2 3 4 5 6 7 8 9 10
Time (years)
Number at risk
hsa−mir−192 exp
Low (=Q1) 93 64 30 17 12 7 6 3 2 1 0
High (=Q3) 93 67 38 24 15 10 6 1 1 0 0
0 1 2 3 4 5 6 7 8 9 10
Time (years)

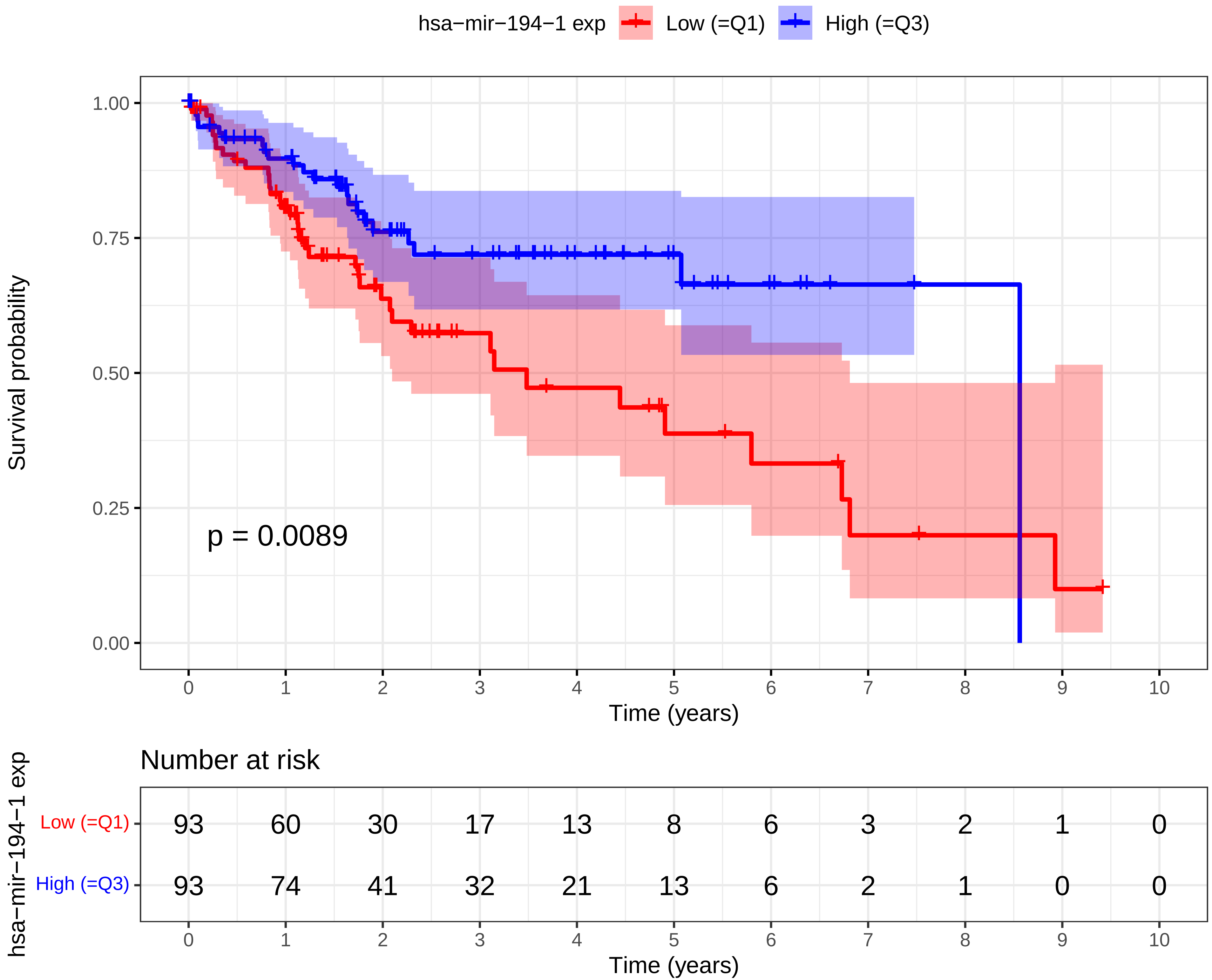

hsa−mir−194−1 exp
Low (=Q1)
High (=Q3)
Survival probability
1.00
0.75
0.50
0.25
0.00
p = 0.0089
0 1 2 3 4 5 6 7 8 9 10
Time (years)
Number at risk
hsa−mir−194−1 exp
Low (=Q1) 93 60 30 17 13 8 6 3 2 1 0
High (=Q3) 93 74 41 32 21 13 6 2 1 0 0
0 1 2 3 4 5 6 7 8 9 10
Time (years)

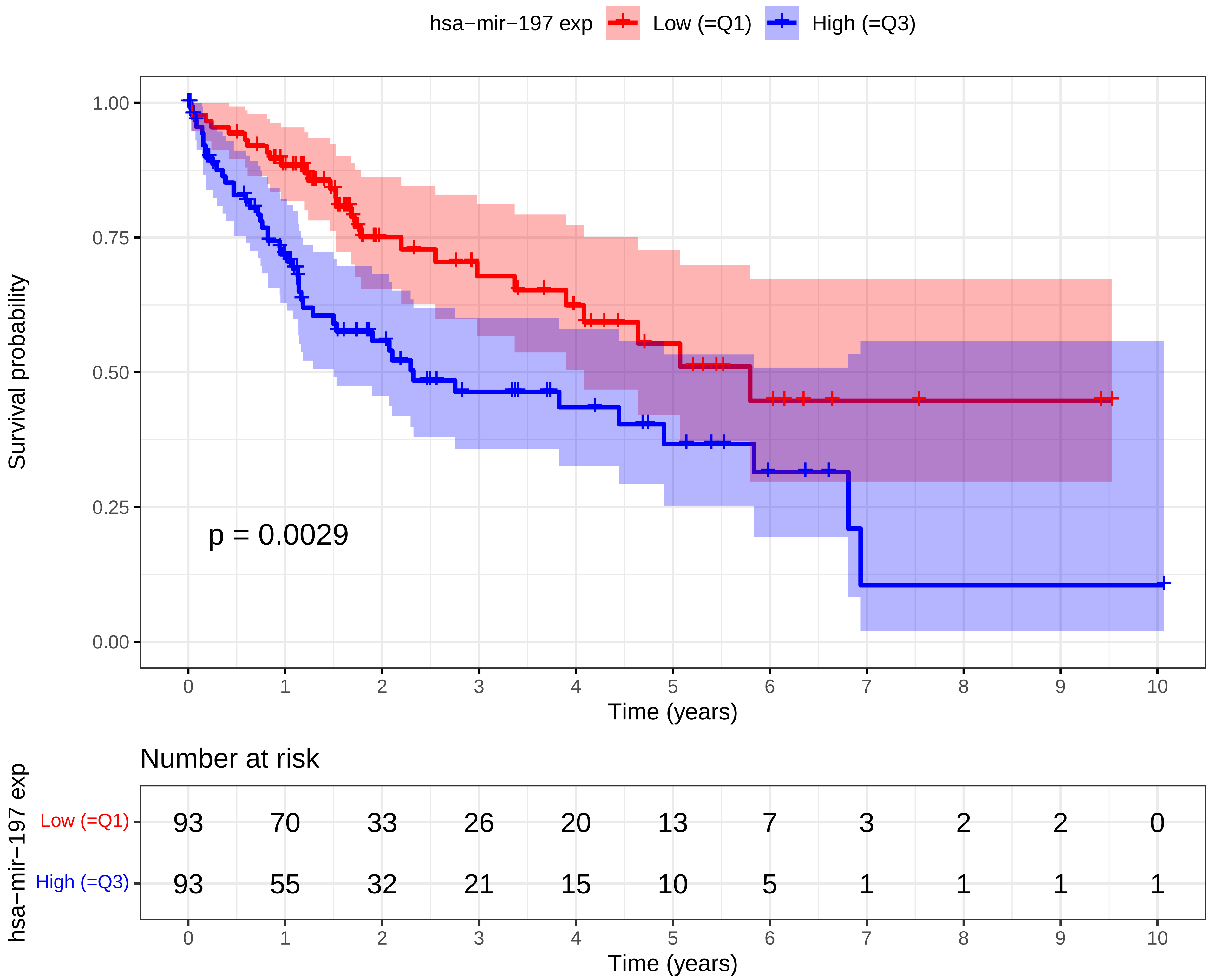
hsa−mir−197 exp
Low (=Q1)
High (=Q3)
Survival probability
1.00
0.75
0.50
0.25
0.00
p = 0.0029
0
1
2
3
4
5
6
7
8
9
10
Time (years)
Number at risk
hsa−mir−197 exp
Low (=Q1)
High (=Q3)
93 70 33 26 20 13 7 3 2 2 0
93 55 32 21 15 10 5 1 1 1 1
0
1
2
3
4
5
6
7
8
9
10
Time (years)

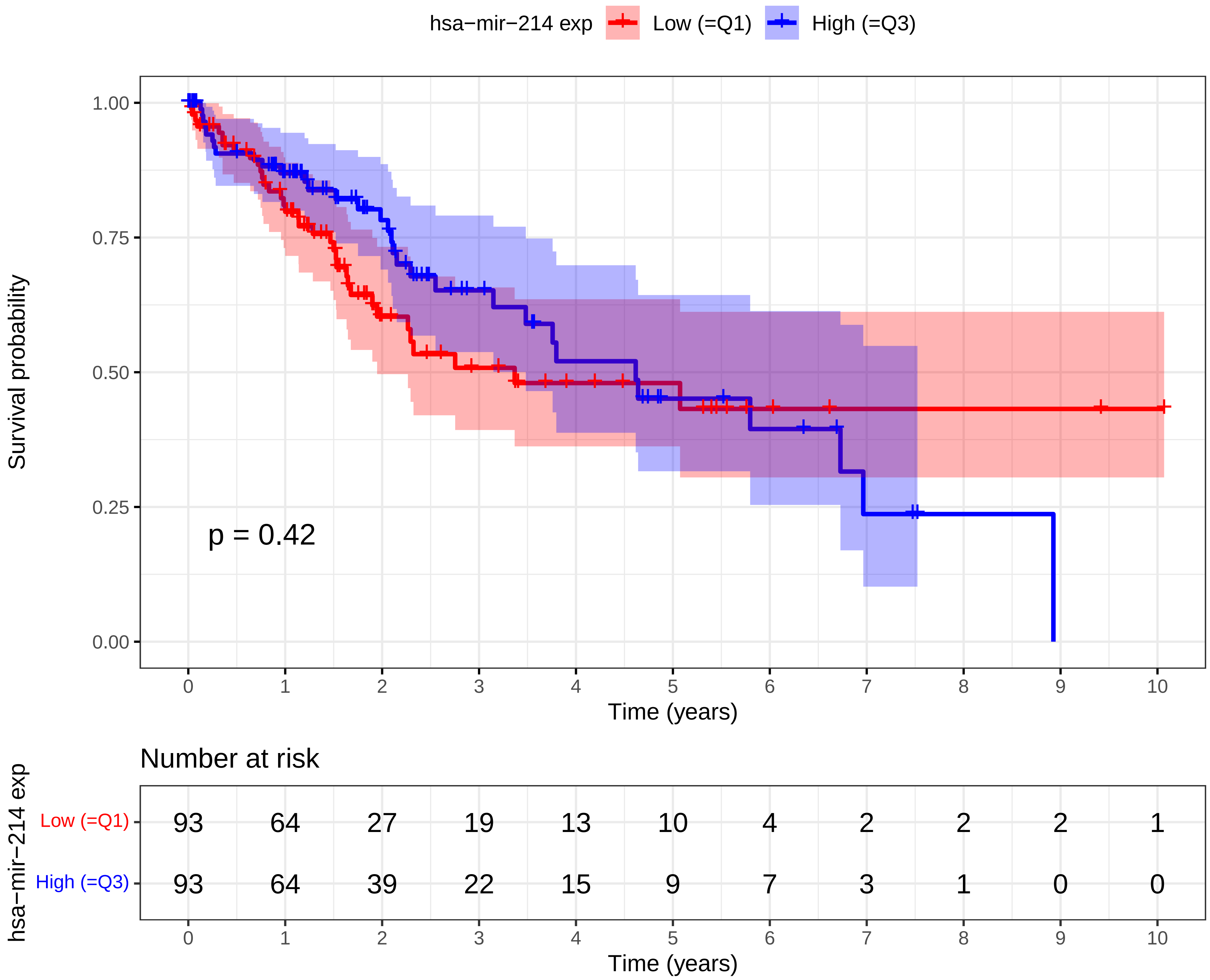
hsa−mir−214 exp
Low (=Q1)
High (=Q3)
Survival probability
1.00
0.75
0.50
0.25
0.00
p = 0.42
0 1 2 3 4 5 6 7 8 9 10
Time (years)
Number at risk
hsa−mir−214 exp
Low (=Q1) 93 64 27 19 13 10 4 2 2 2 1
High (=Q3) 93 64 39 22 15 9 7 3 1 0 0
0 1 2 3 4 5 6 7 8 9 10
Time (years)

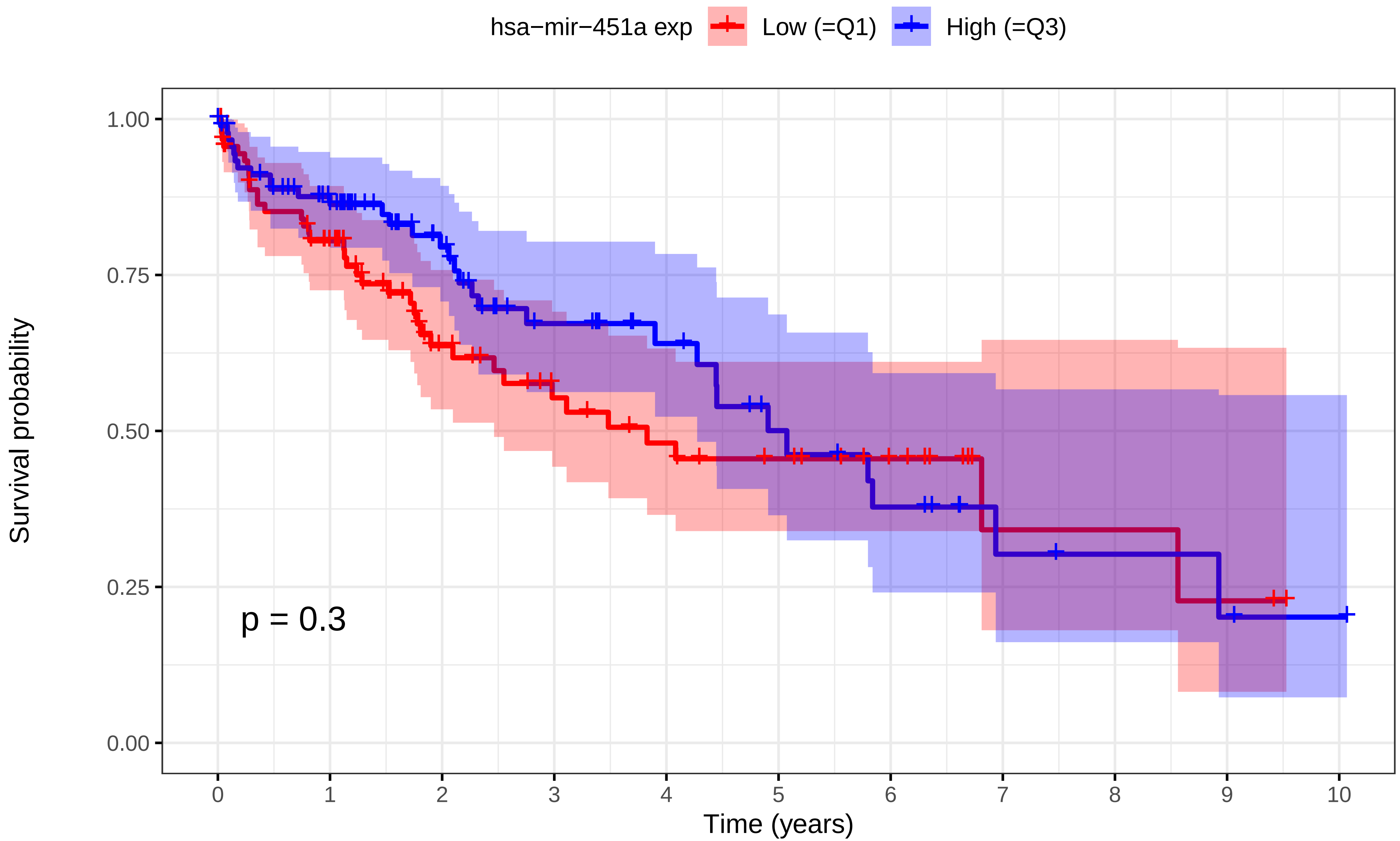

hsa−mir−451a exp	0	1	2	3	4	5	6	7	8	9	10
Low (=Q1)	93	64	34	24	19	15	10	3	3	2	0
High (=Q3)	93	69	43	27	20	13	9	4	3	2	1

Time (years)

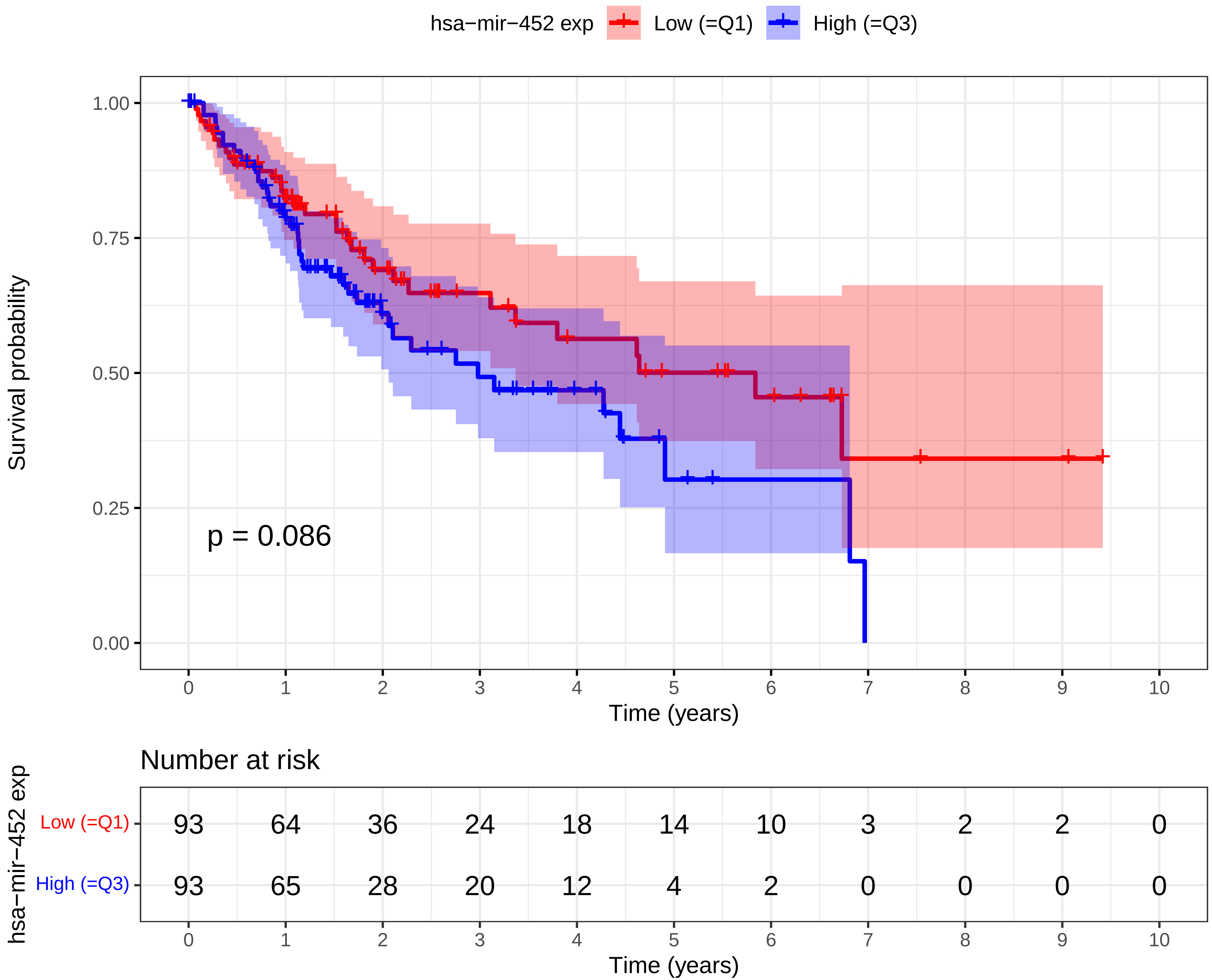
hsa−mir−452 exp
Low (=Q1)
High (=Q3)
Survival probability
1.00
0.75
0.50
0.25
0.00
p = 0.086
Time (years)
0 1 2 3 4 5 6 7 8 9 10
Number at risk
hsa−mir−452 exp
Low (=Q1) 93 64 36 24 18 14 10 3 2 2 0
High (=Q3) 93 65 28 20 12 4 2 0 0 0 0
Time (years)

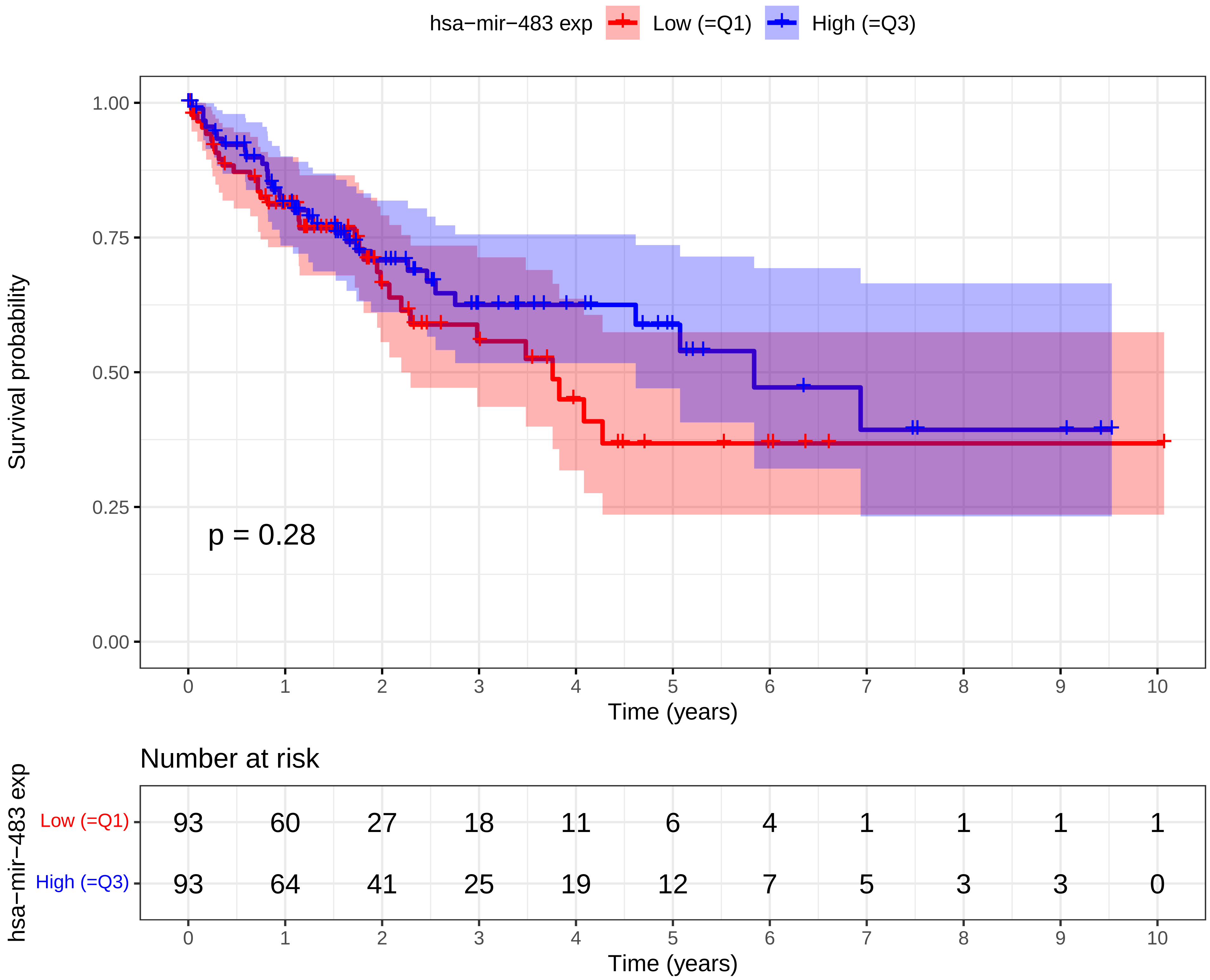
hsa−mir−483 exp
Low (=Q1)
High (=Q3)
Survival probability
1.00
0.75
0.50
0.25
0.00
p = 0.28
0 1 2 3 4 5 6 7 8 9 10
Time (years)
Number at risk
hsa−mir−483 exp
Low (=Q1) 93 60 27 18 11 6 4 1 1 1 1
High (=Q3) 93 64 41 25 19 12 7 5 3 3 0
0 1 2 3 4 5 6 7 8 9 10
Time (years)

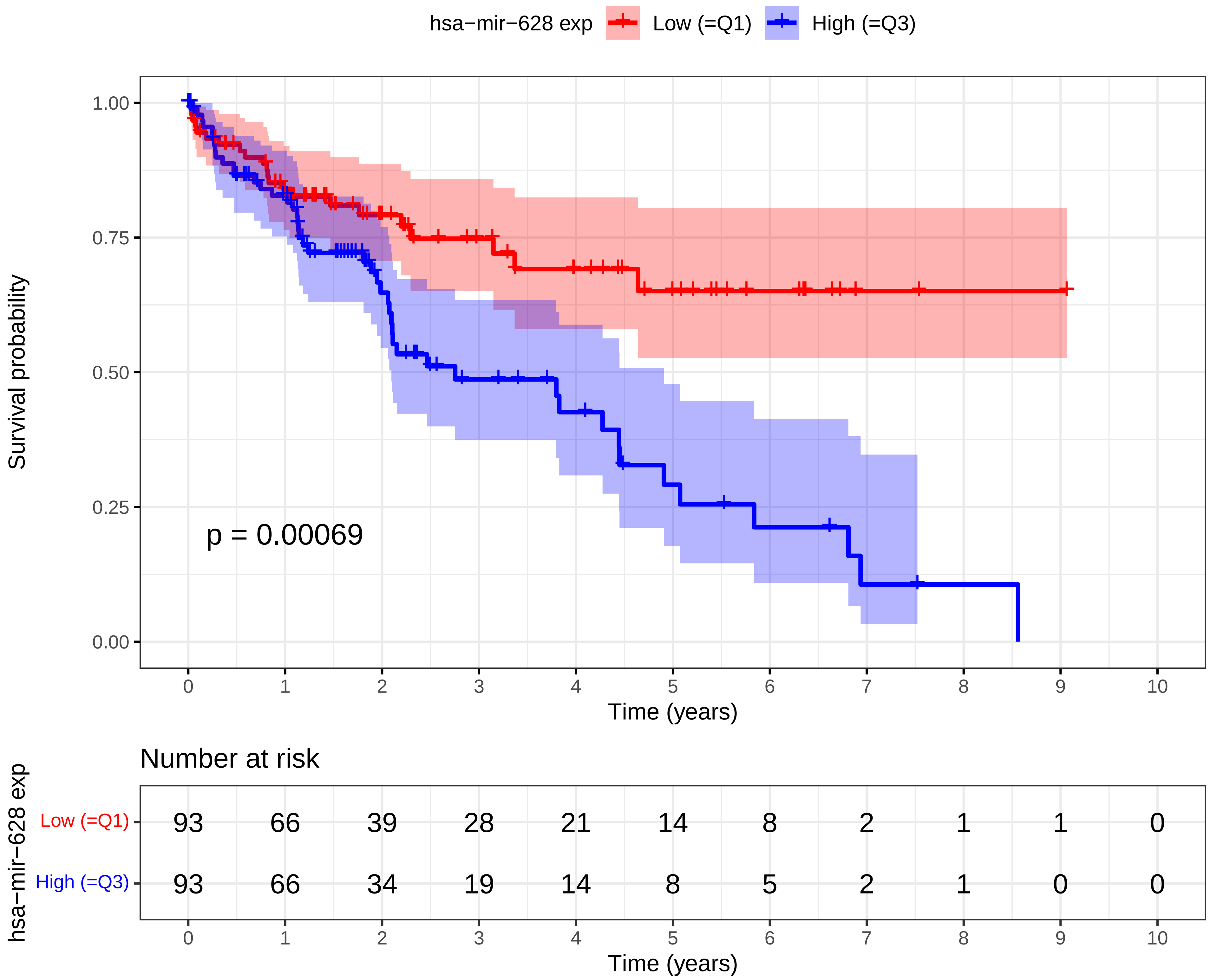
hsa−mir−628 exp
Low (=Q1)
High (=Q3)
Survival probability
1.00
0.75
0.50
0.25
0.00
p = 0.00069
0 1 2 3 4 5 6 7 8 9 10
Time (years)
Number at risk
hsa−mir−628 exp
Low (=Q1) 93 66 39 28 21 14 8 2 1 1 0
High (=Q3) 93 66 34 19 14 8 5 2 1 0 0
0 1 2 3 4 5 6 7 8 9 10
Time (years)

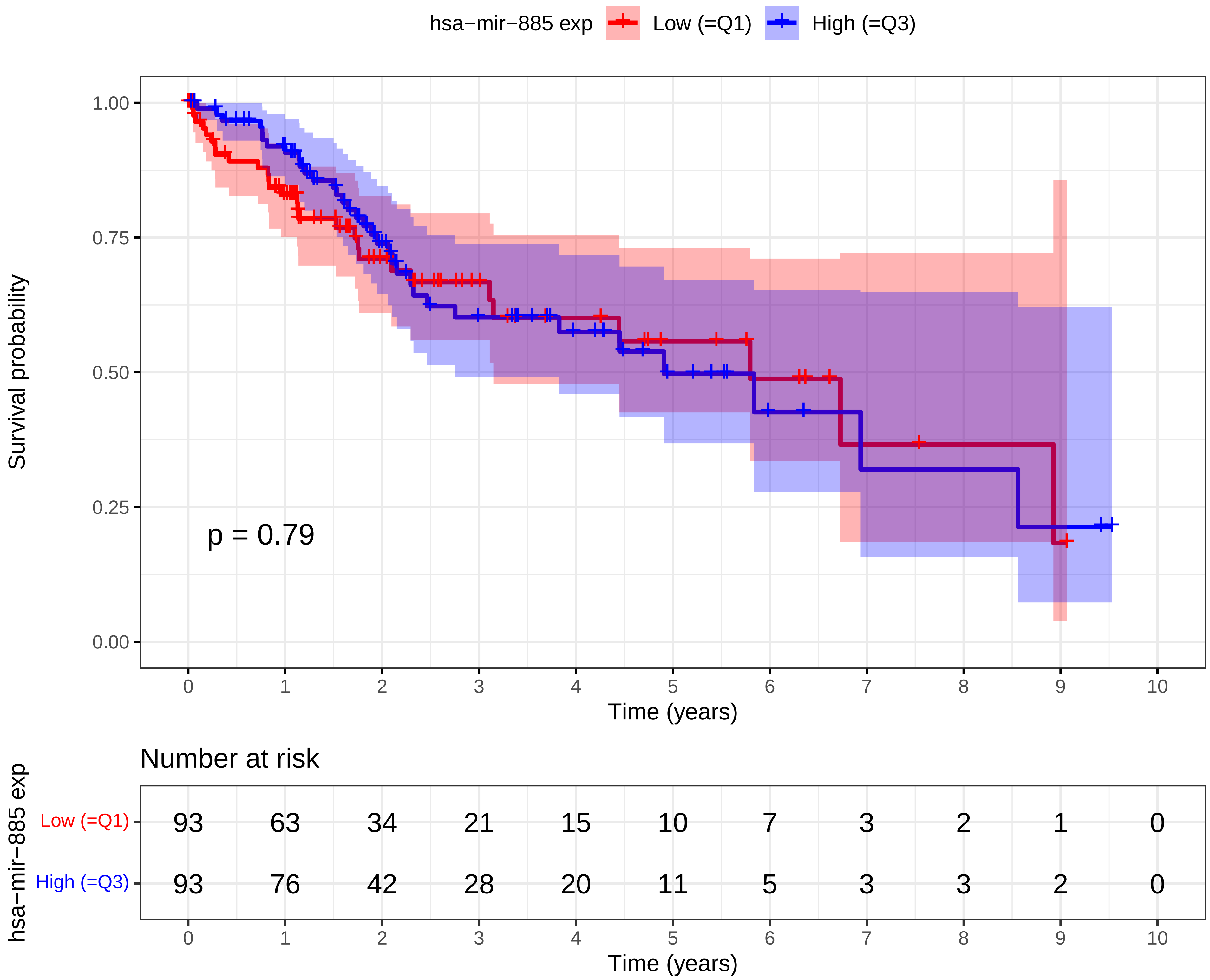

hsa−mir−885 exp
Low (=Q1)
High (=Q3)
Survival probability
1.00
0.75
0.50
0.25
0.00
p = 0.79
0 1 2 3 4 5 6 7 8 9 10
Time (years)
Number at risk
hsa−mir−885 exp
Low (=Q1) 93 63 34 21 15 10 7 3 2 1 0
High (=Q3) 93 76 42 28 20 11 5 3 3 2 0
0 1 2 3 4 5 6 7 8 9 10
Time (years)

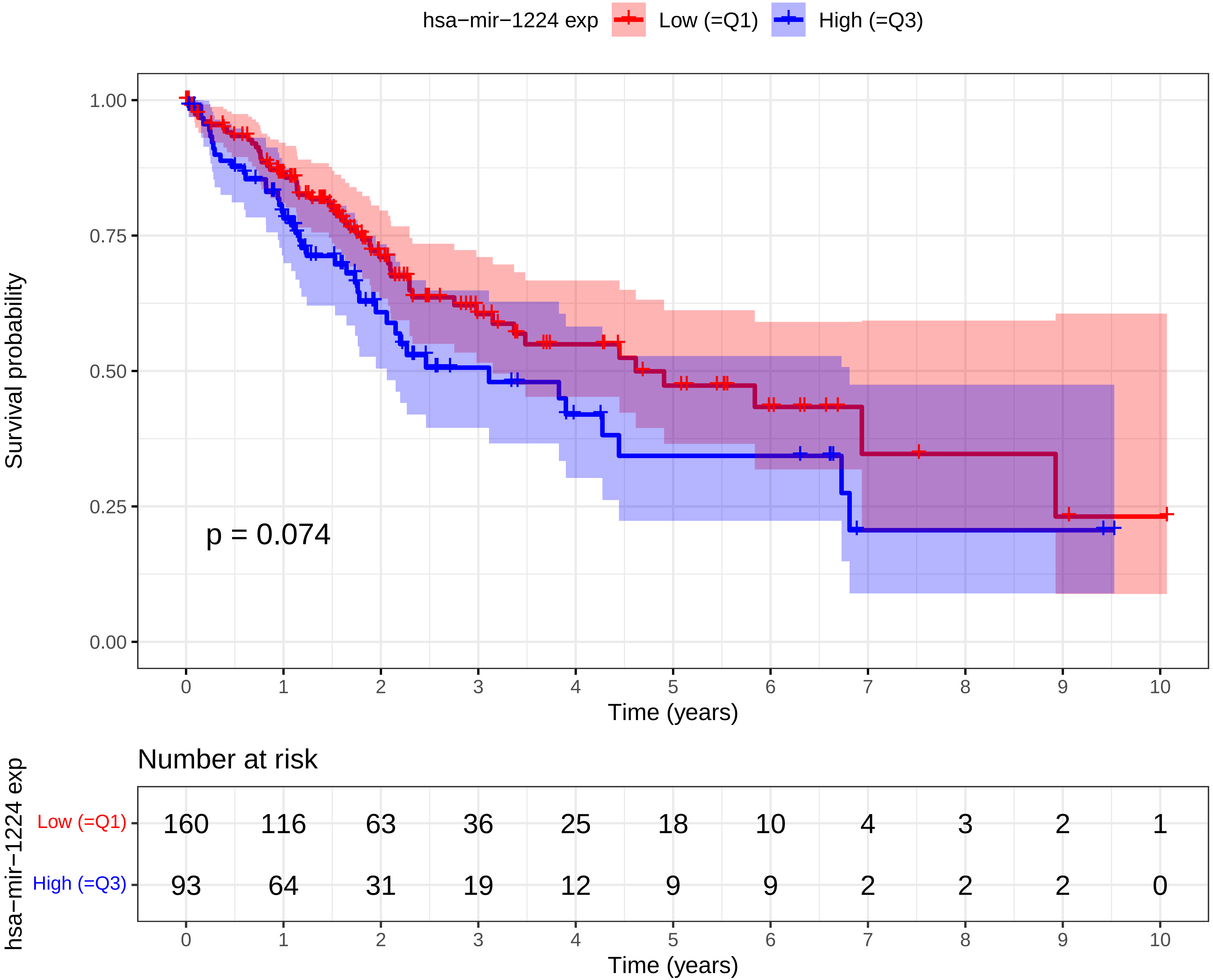
hsa−mir−1224 exp
Low (=Q1)
High (=Q3)
Survival probability
1.00
0.75
0.50
0.25
0.00
p = 0.074
0
1
2
3
4
5
6
7
8
9
10
Time (years)
Number at risk
hsa−mir−1224 exp
Low (=Q1) 160 116 63 36 25 18 10 4 3 2 1
High (=Q3) 93 64 31 19 12 9 9 2 2 2 0
Time (years)

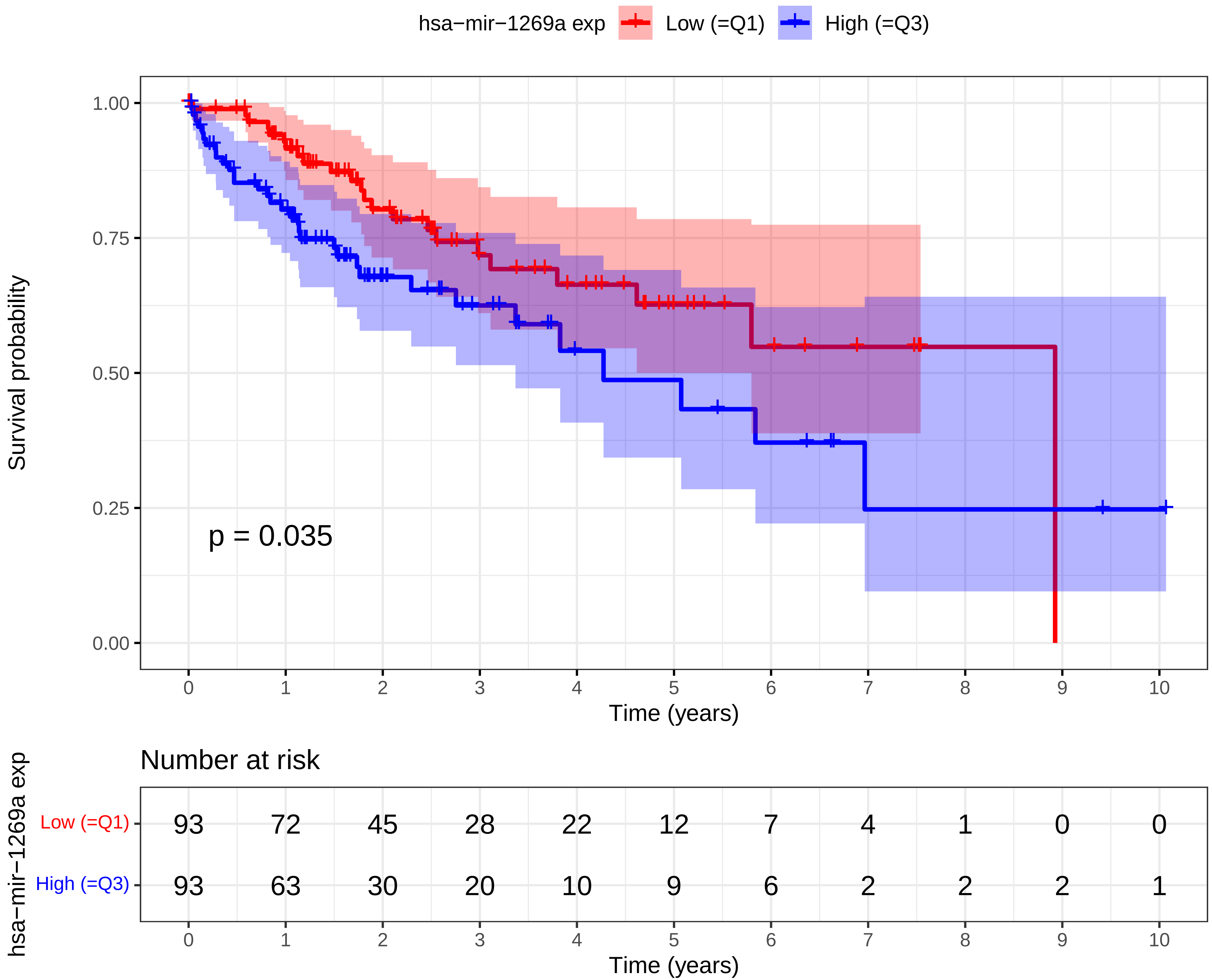
hsa−mir−1269a exp
Low (=Q1)
High (=Q3)
Survival probability
1.00
0.75
0.50
0.25
0.00
p = 0.035
0 1 2 3 4 5 6 7 8 9 10
Time (years)
Number at risk
hsa−mir−1269a exp
Low (=Q1) 93 72 45 28 22 12 7 4 1 0 0
High (=Q3) 93 63 30 20 10 9 6 2 2 2 1
0 1 2 3 4 5 6 7 8 9 10
Time (years)

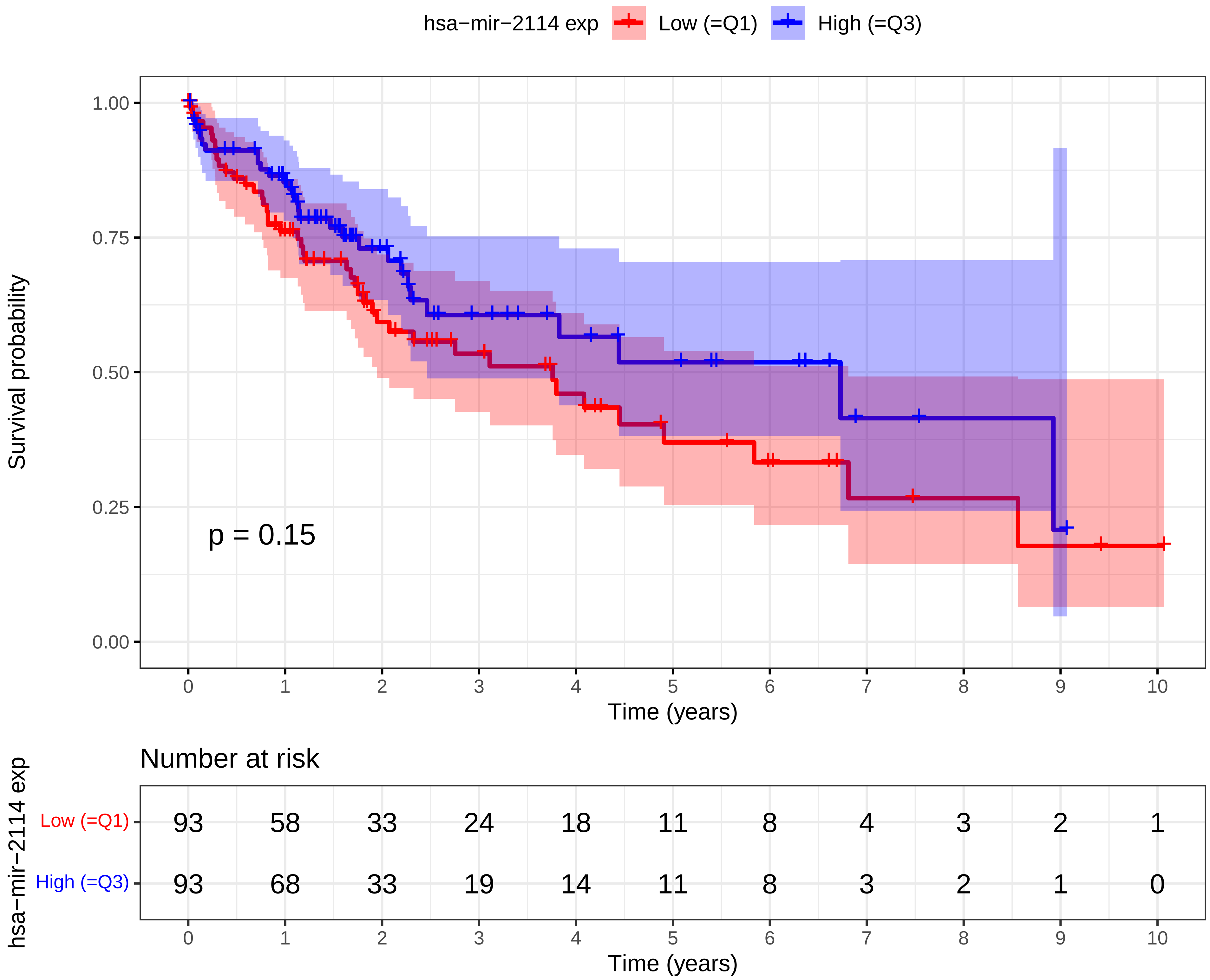

hsa−mir−2114 exp
Low (=Q1)
High (=Q3)
Survival probability
1.00
0.75
0.50
0.25
0.00
p = 0.15
0 1 2 3 4 5 6 7 8 9 10
Time (years)
Number at risk
hsa−mir−2114 exp
Low (=Q1) 93 58 33 24 18 11 8 4 3 2 1
High (=Q3) 93 68 33 19 14 11 8 3 2 1 0
0 1 2 3 4 5 6 7 8 9 10
Time (years)
