# Supplementary material for: Optimization of extracellular vesicle extraction from hepatic tissue interstitial fluid and analysis of their ncRNA expression profiles
Source: PLoS One. 2026 Aug 3;21(8):e0355303. doi: 10.1371/journal.pone.0355303 (PMC13432105; doi:10.1371/journal.pone.0355303)

Fig 1D Alix

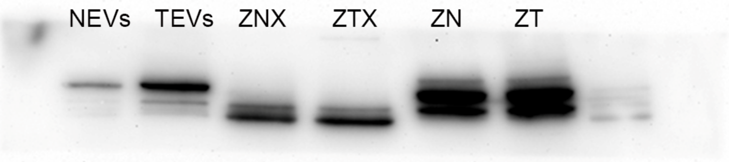

Fig 1D Alixmarker

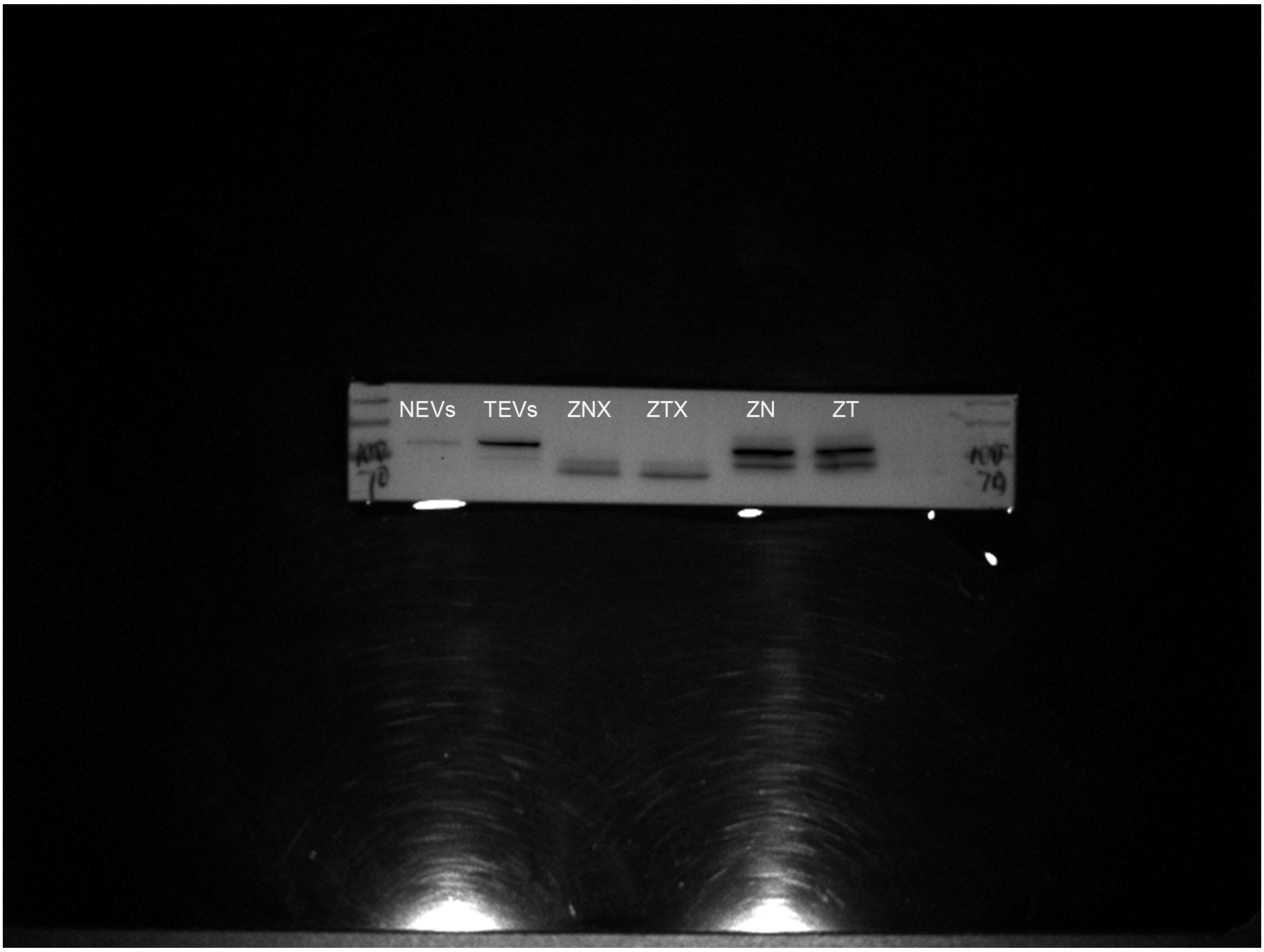

Fig 1D ASGPR

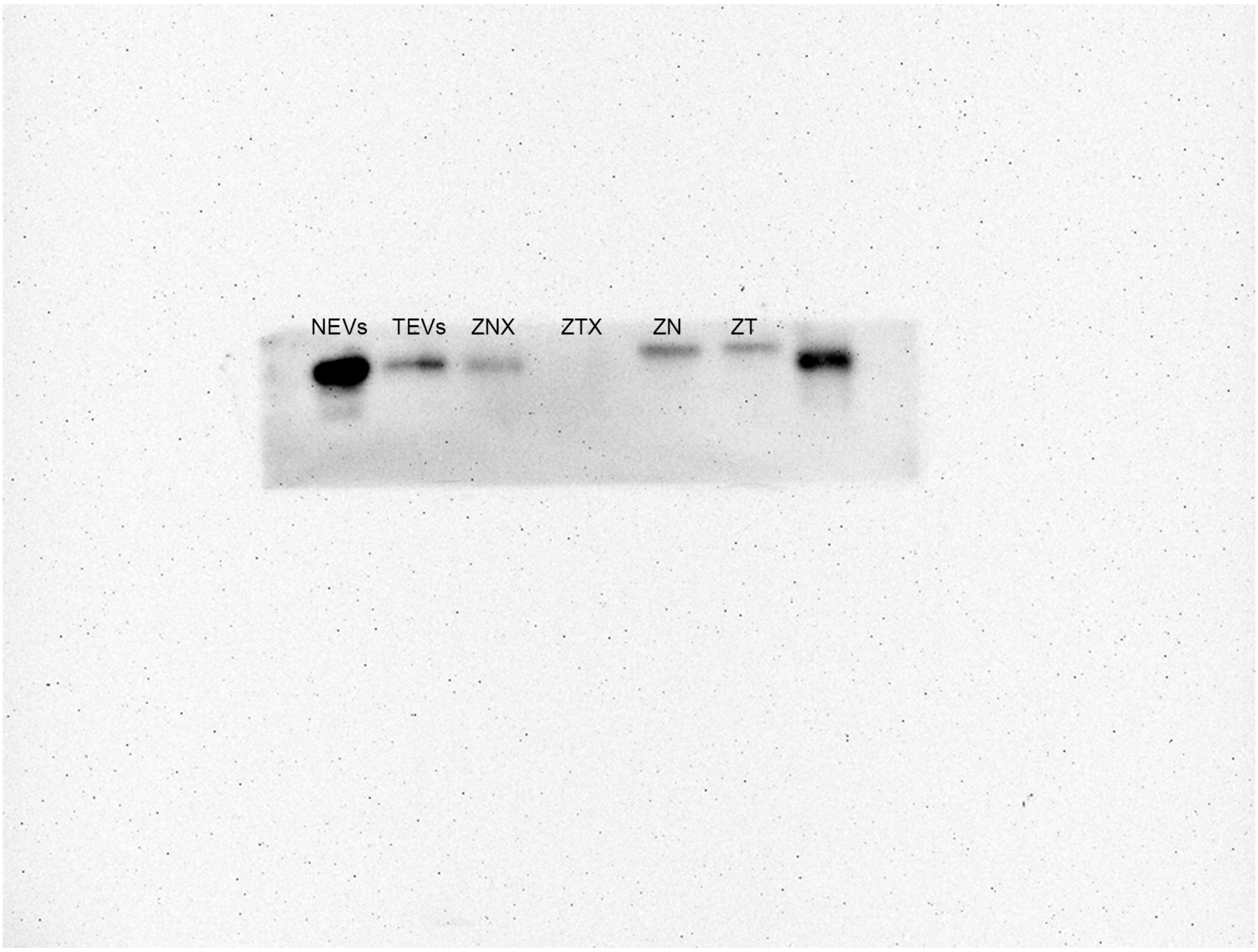

Fig 1D ASGPRmarker

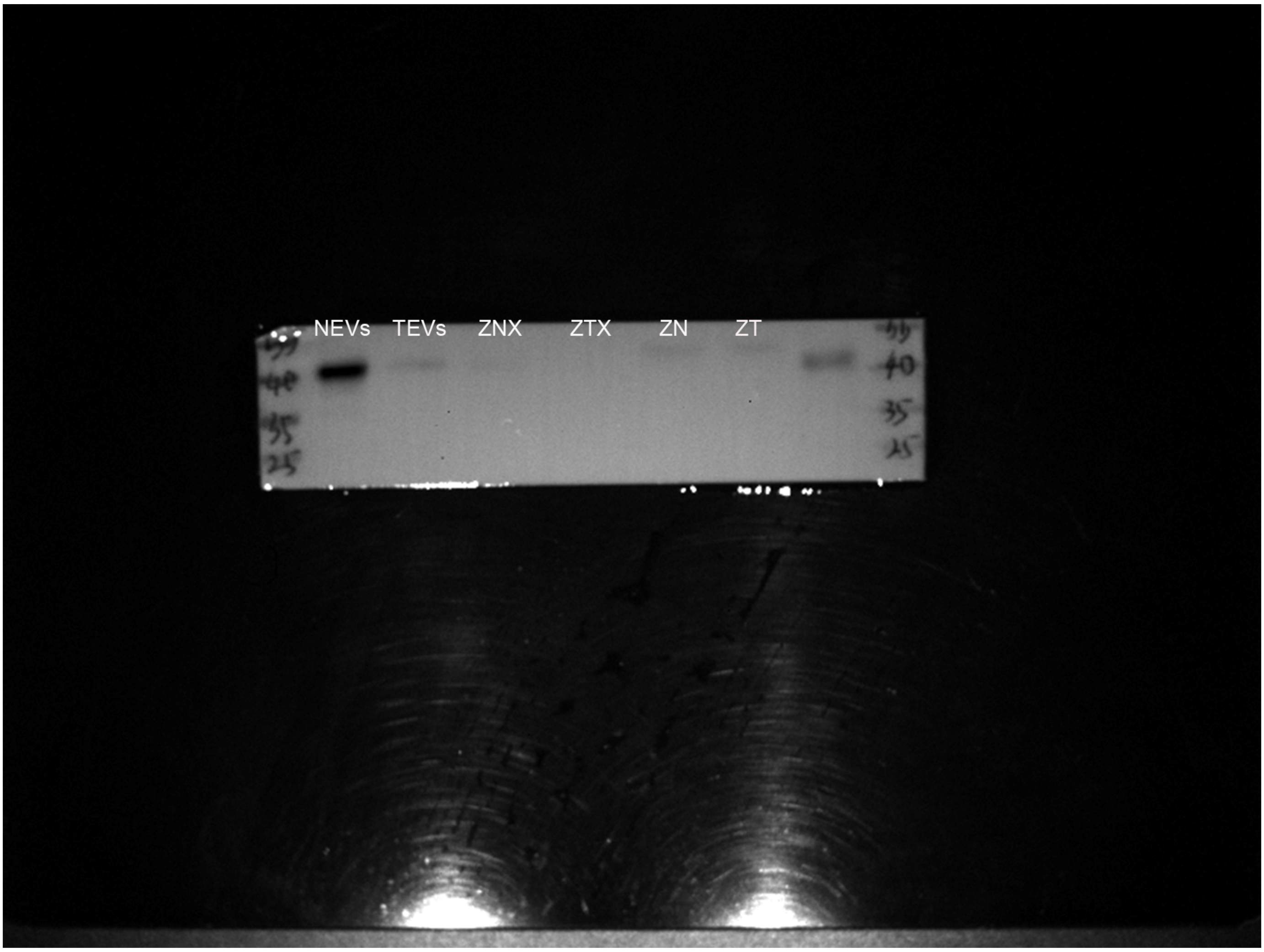

Fig 1D CD63

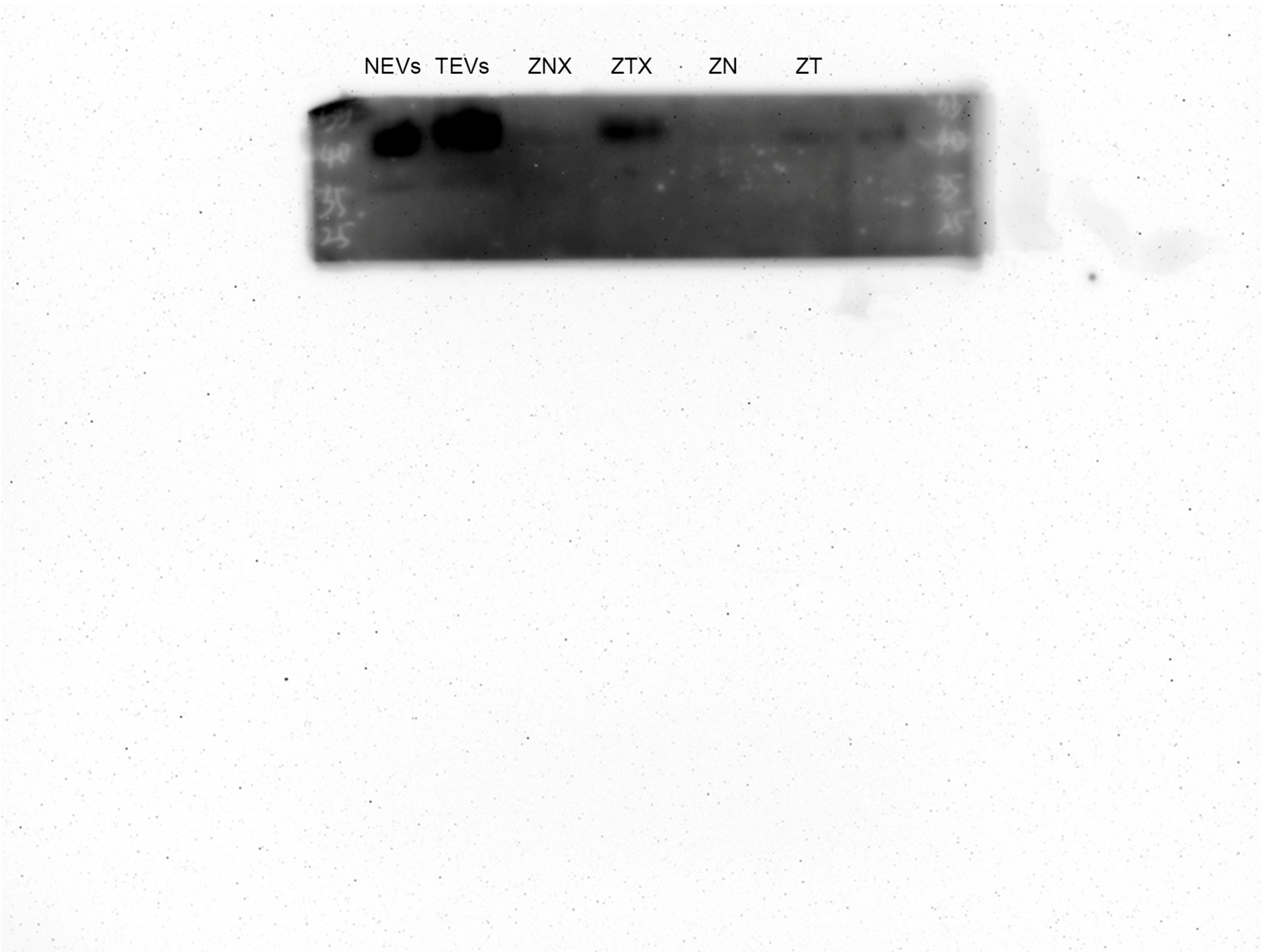

Fig 1D CD63marker

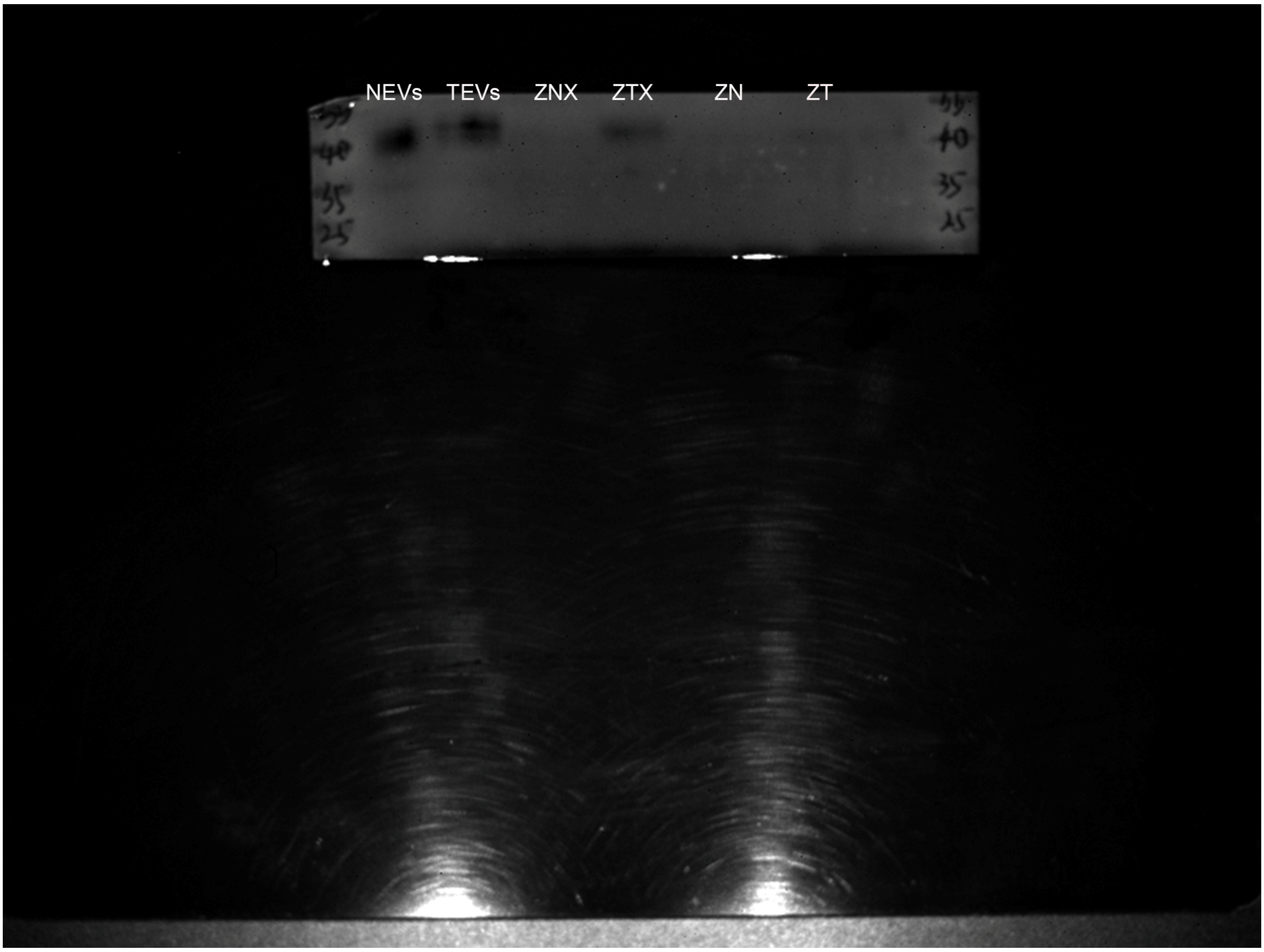

Fig 1D HSP70

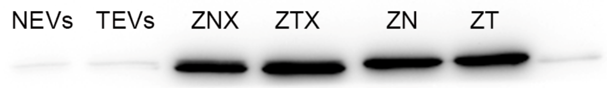

Fig 1D HSP70marker

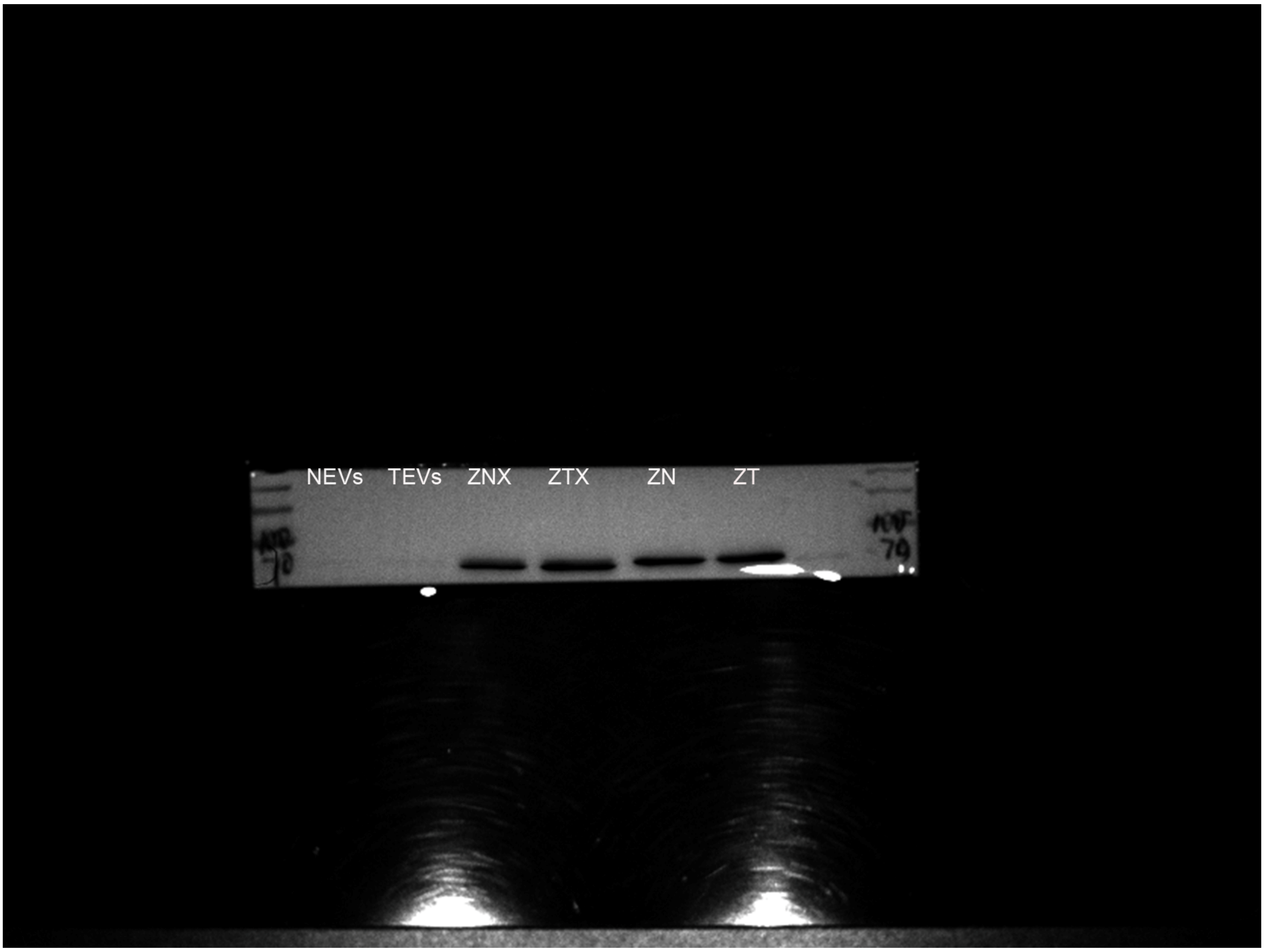

Fig 1D TSG101

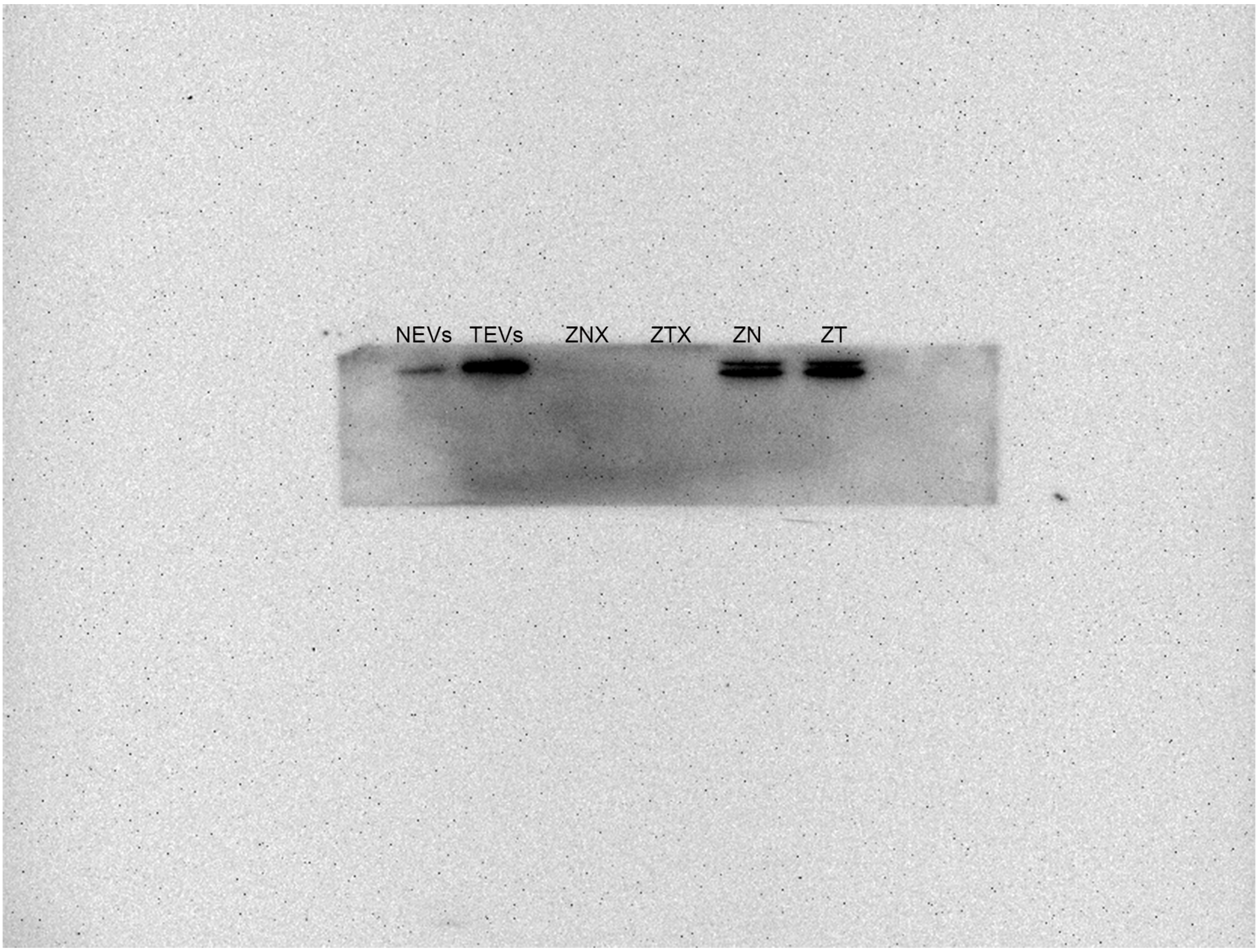

Fig 1D TSG101marker

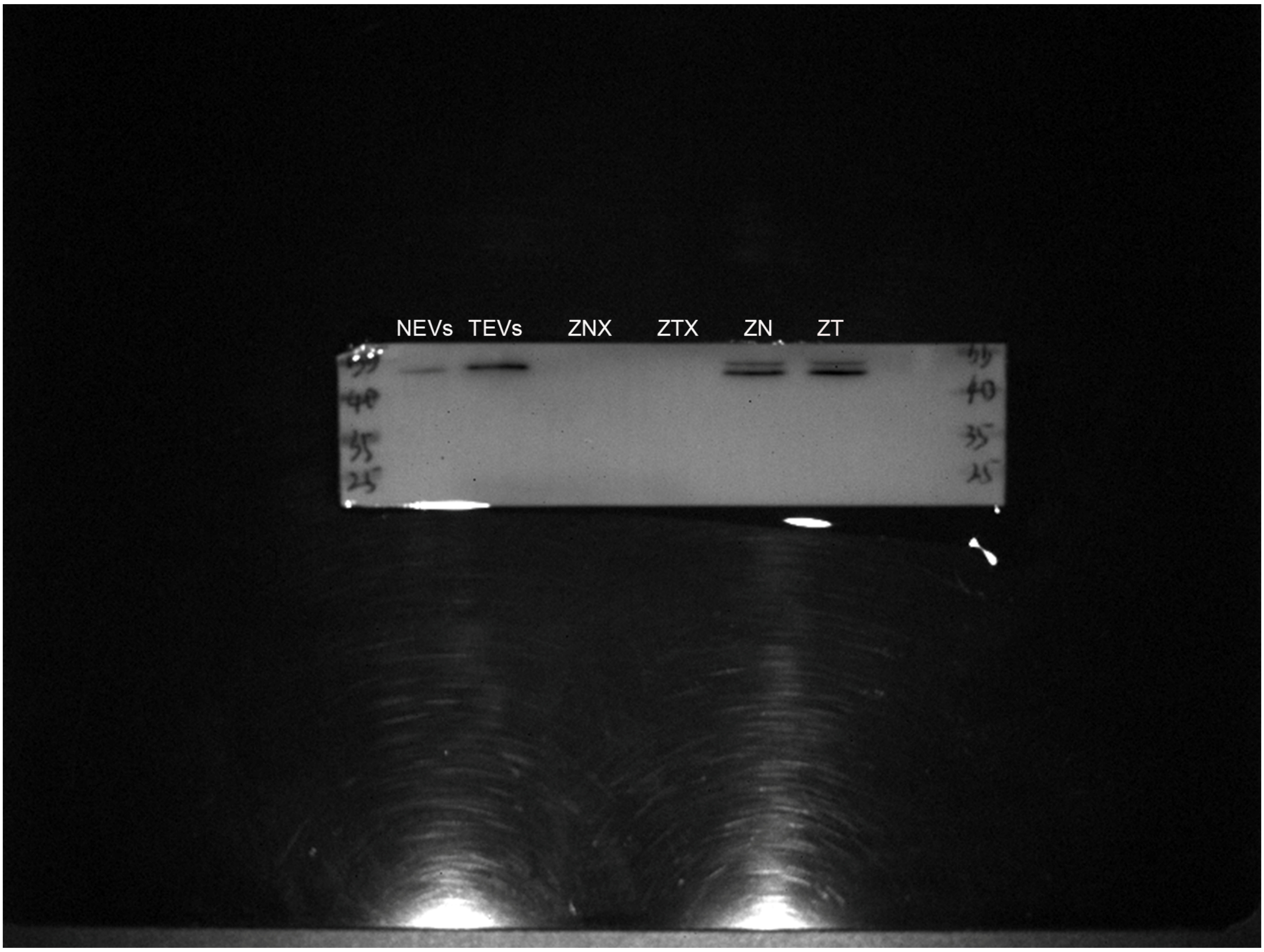

Fig 1E Alix

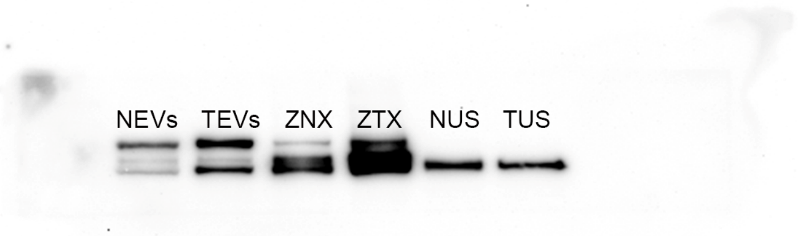

Fig 1E Alixmarker

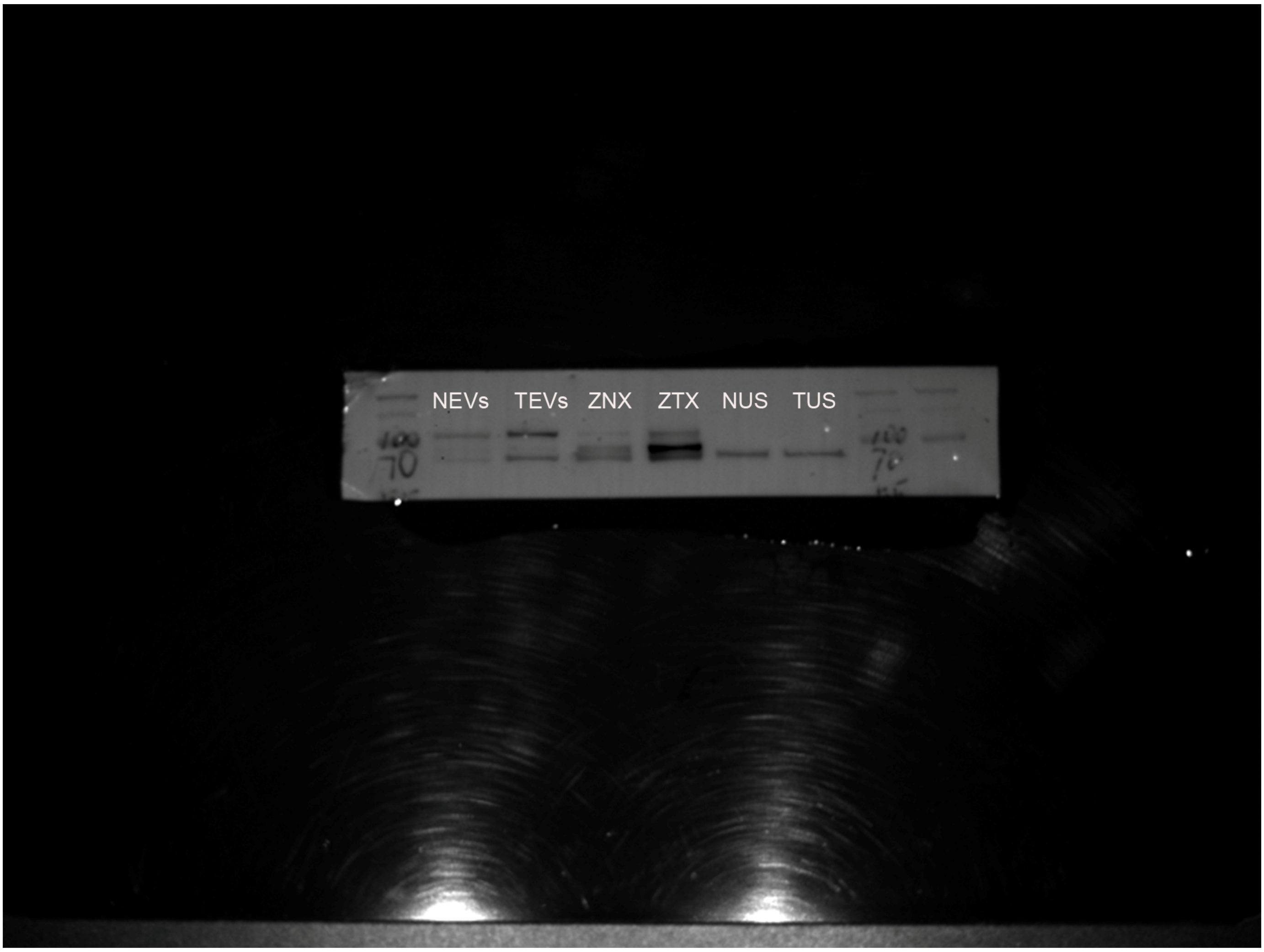

Fig 1E ASGPR

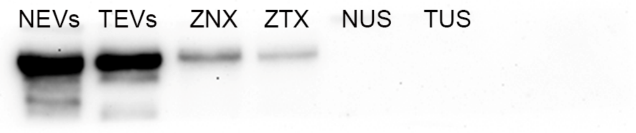

Fig 1E ASGPRmarker

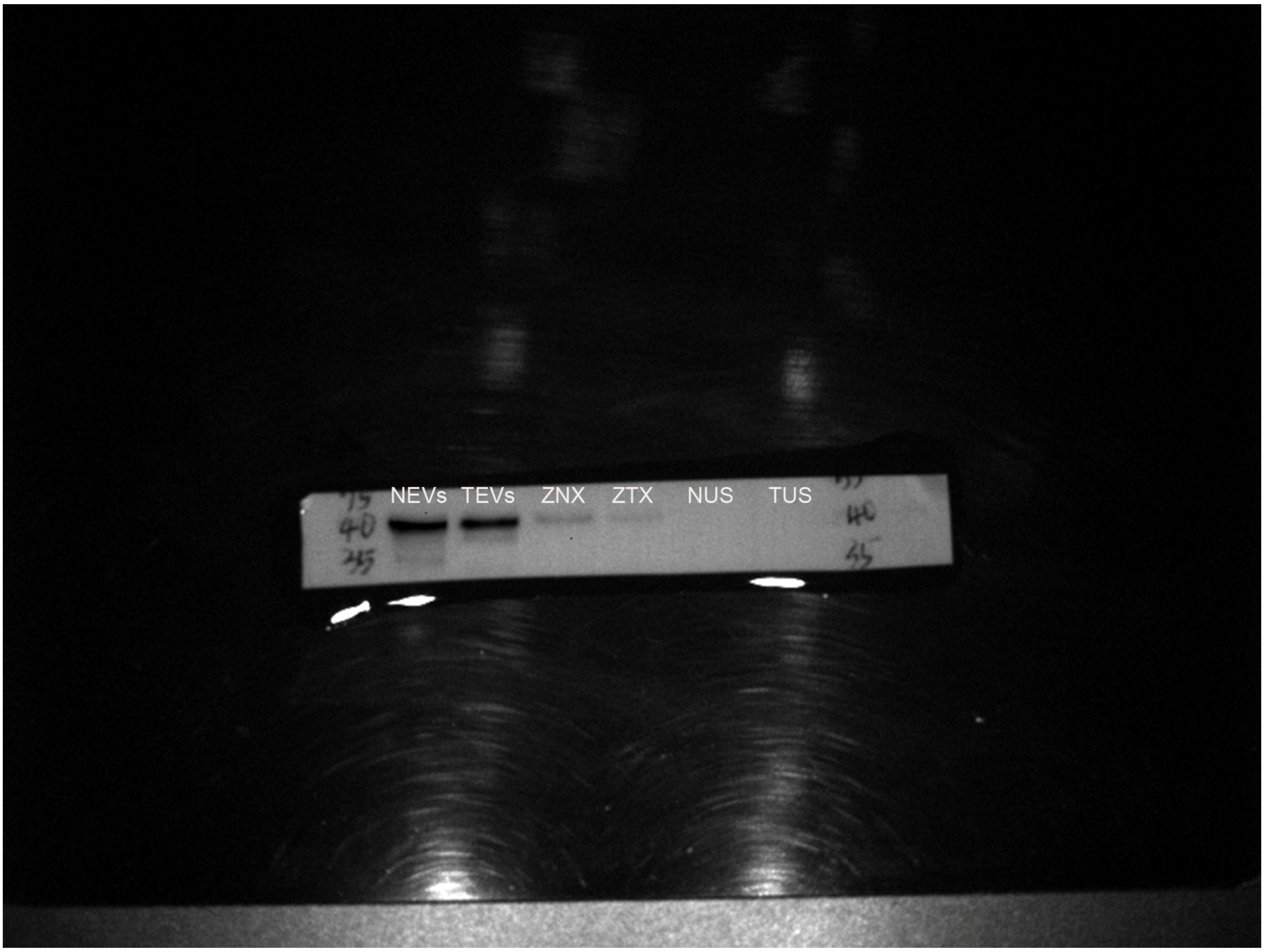

Fig 1E CD63

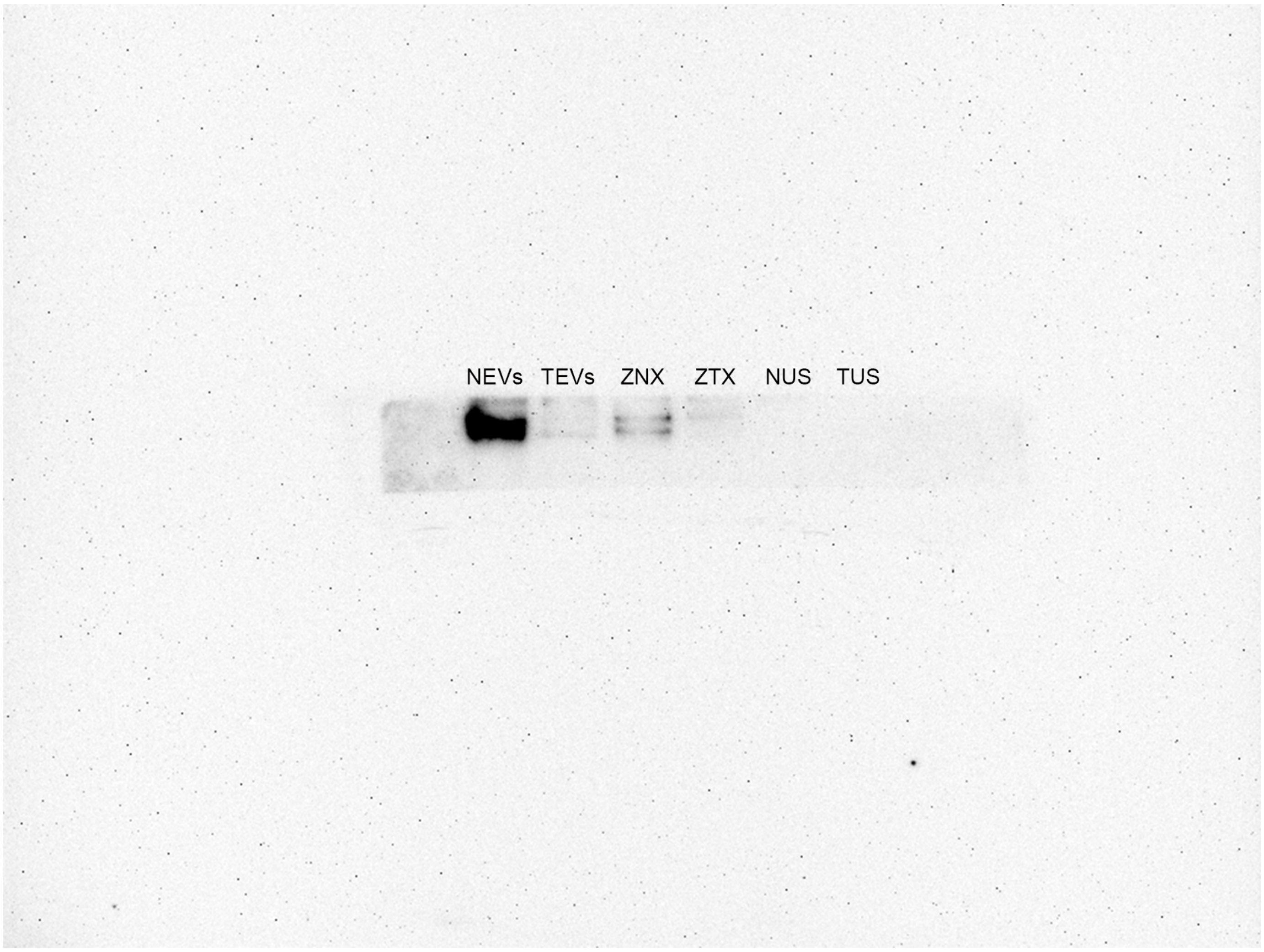

Fig 1E CD63marker

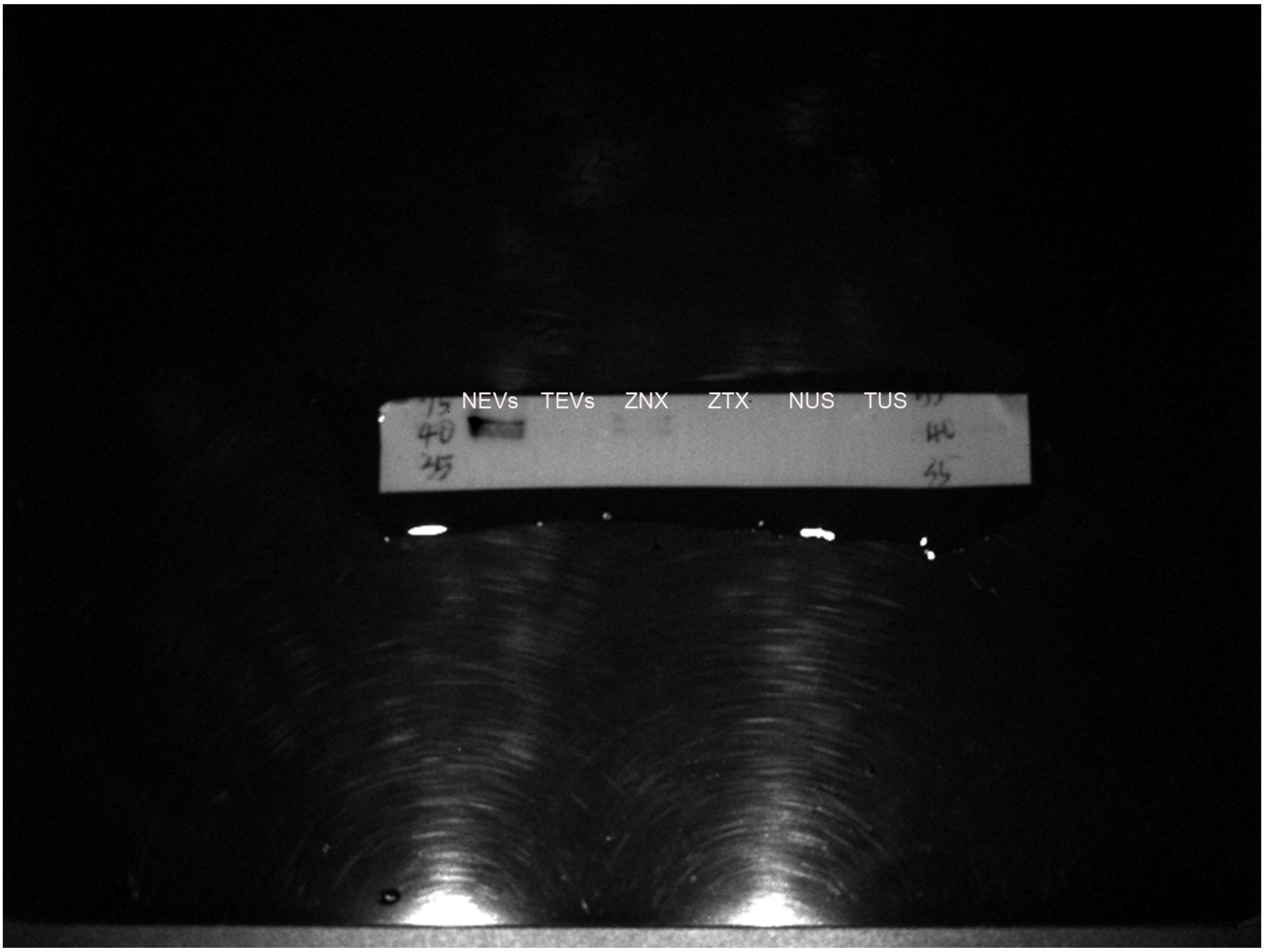

Fig 1E HSP70

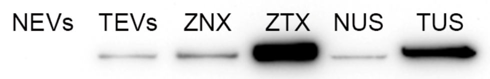

Fig 1E HSP70marker

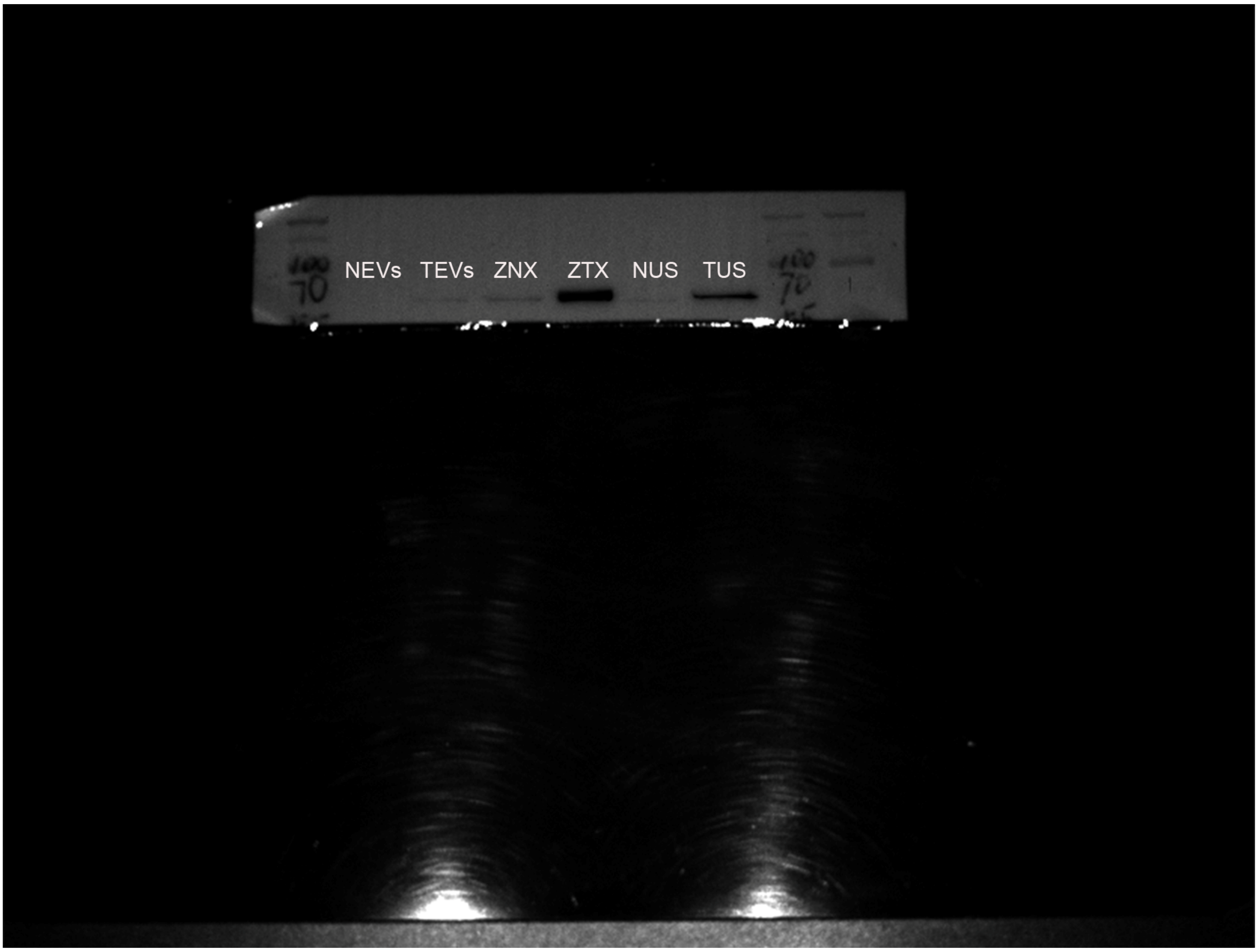

S1 Fig1E CD34+CD9 及 marker

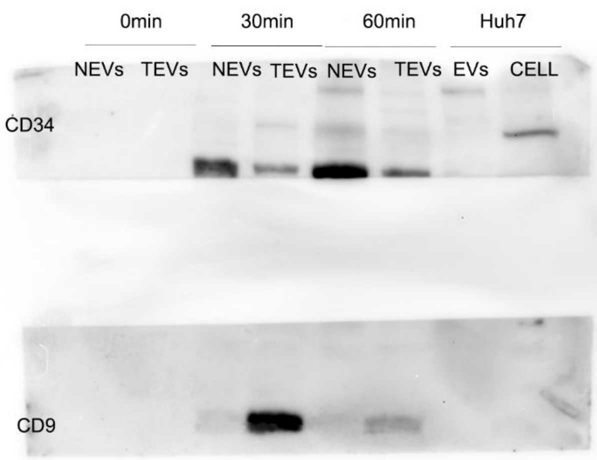


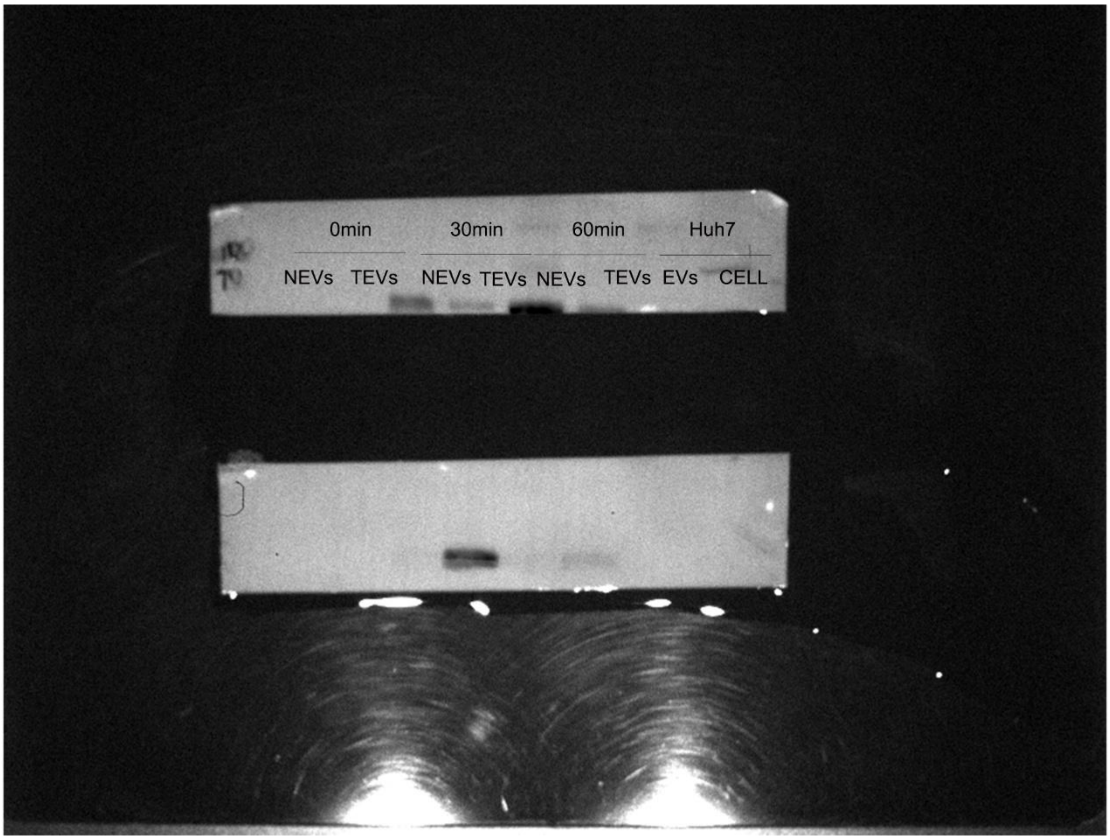

S1 Fig1E ALIX 及 marker

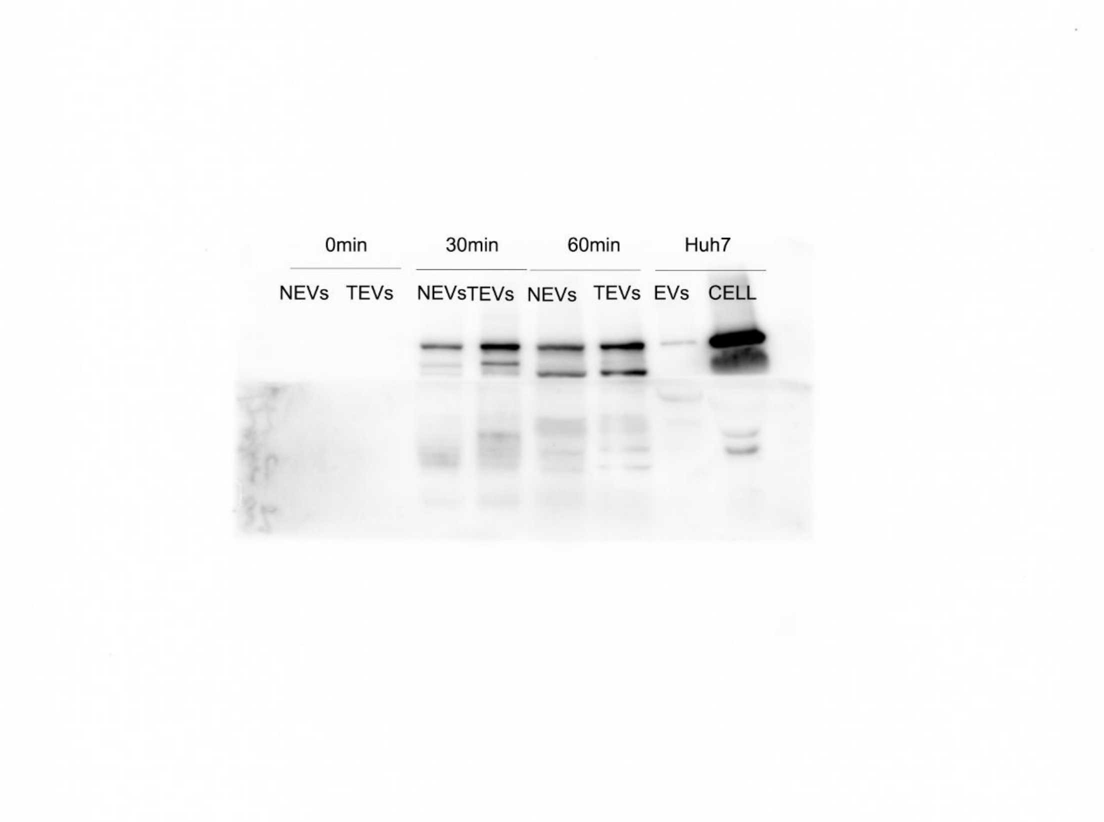


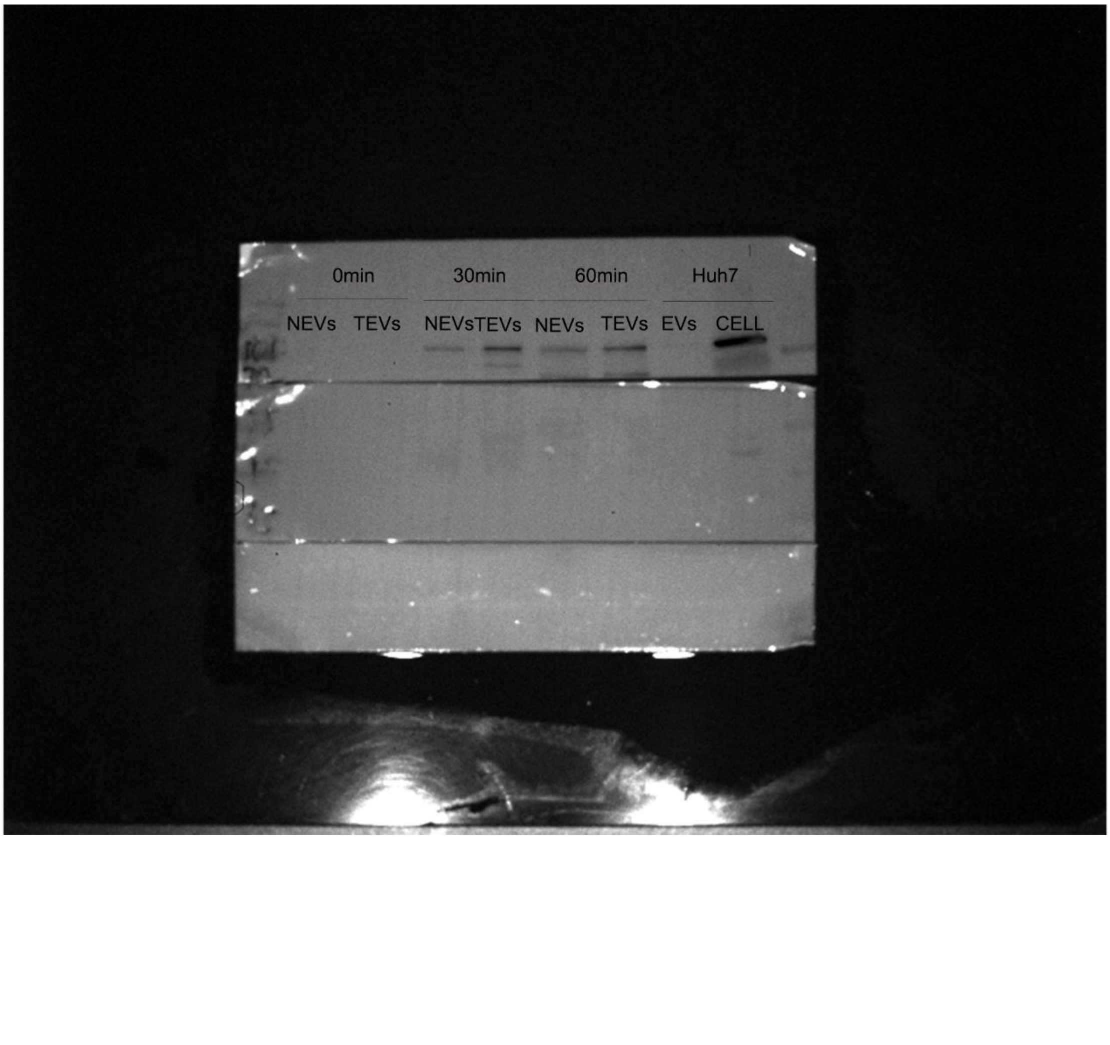

S1 Fig1E Calnexin 和 ASGPR 及 marker

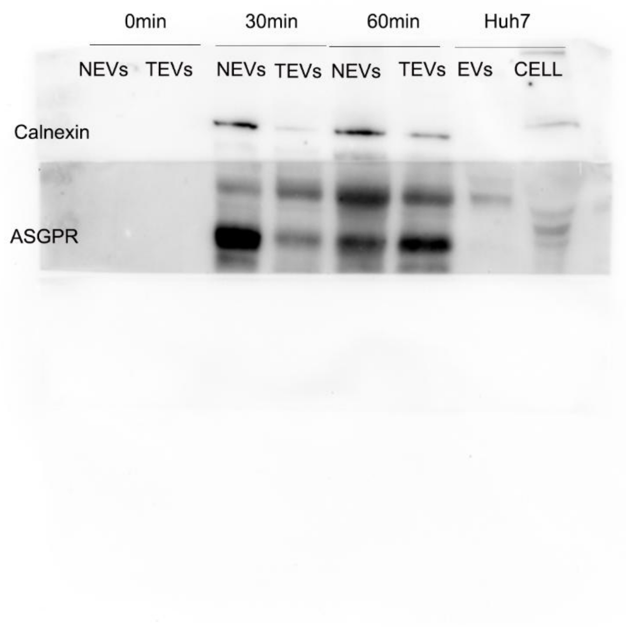


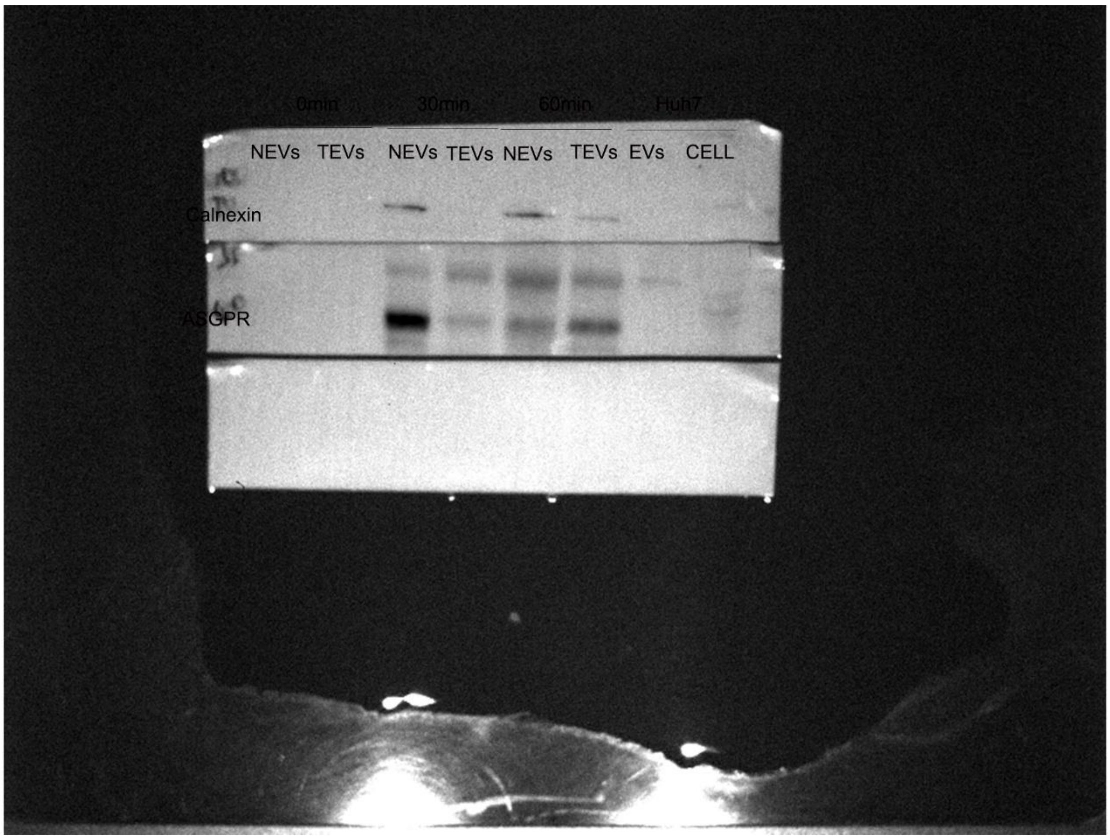

S1 Fig1E ALB 及 marker

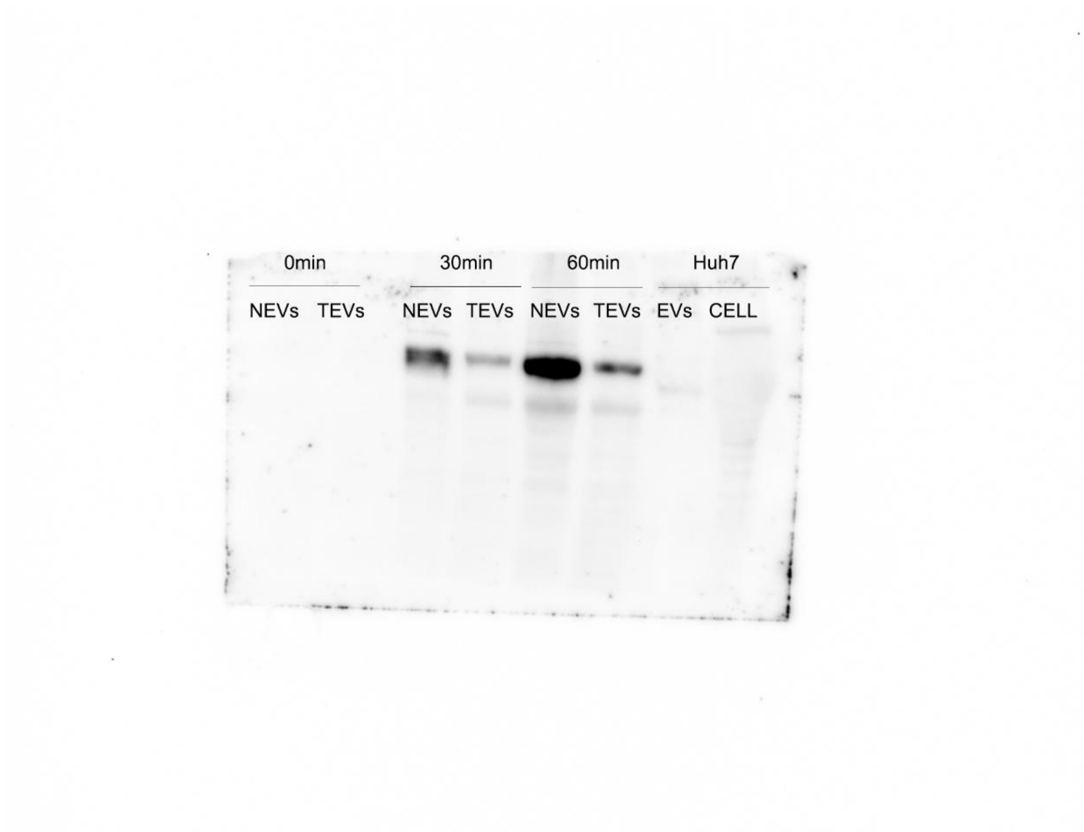


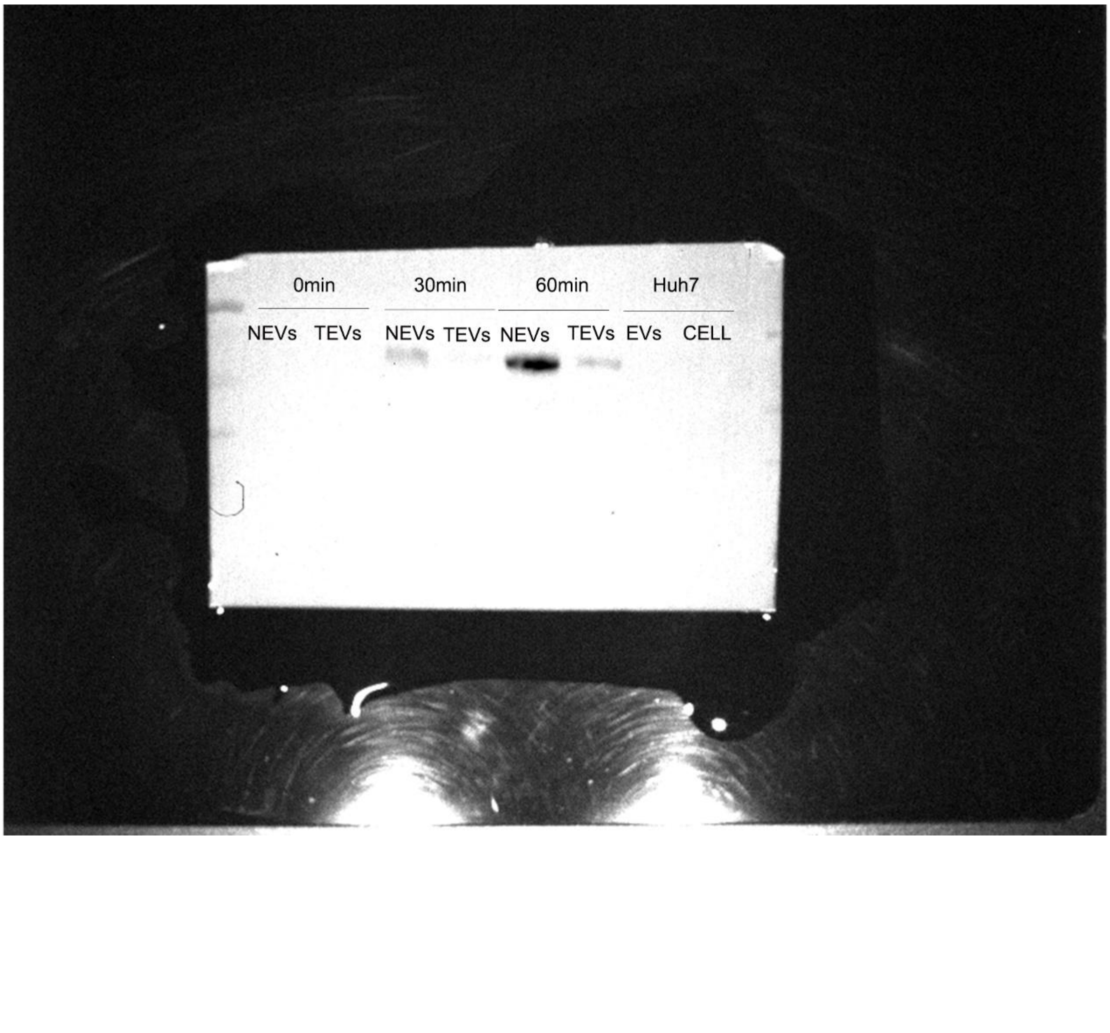

S1 Fig1E CD63 及 marker

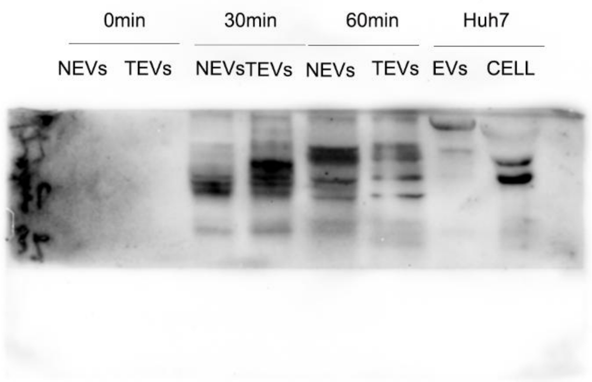


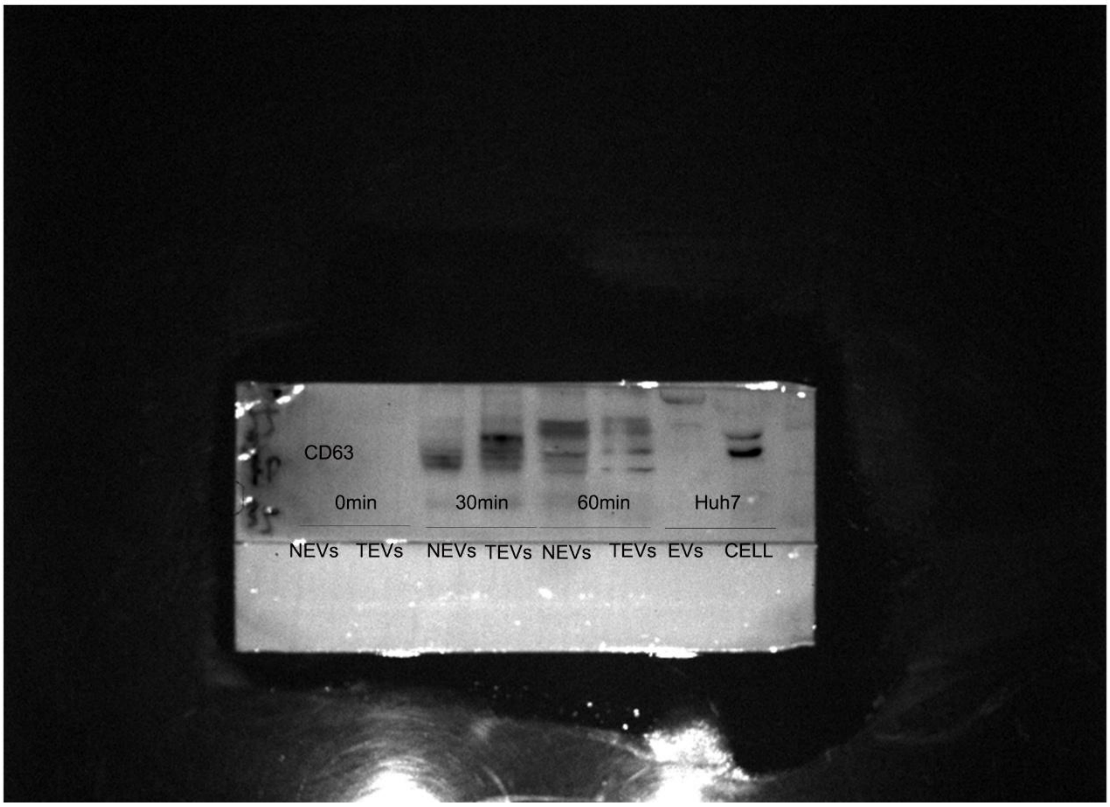

S1 Fig1E TSG101 及 marker

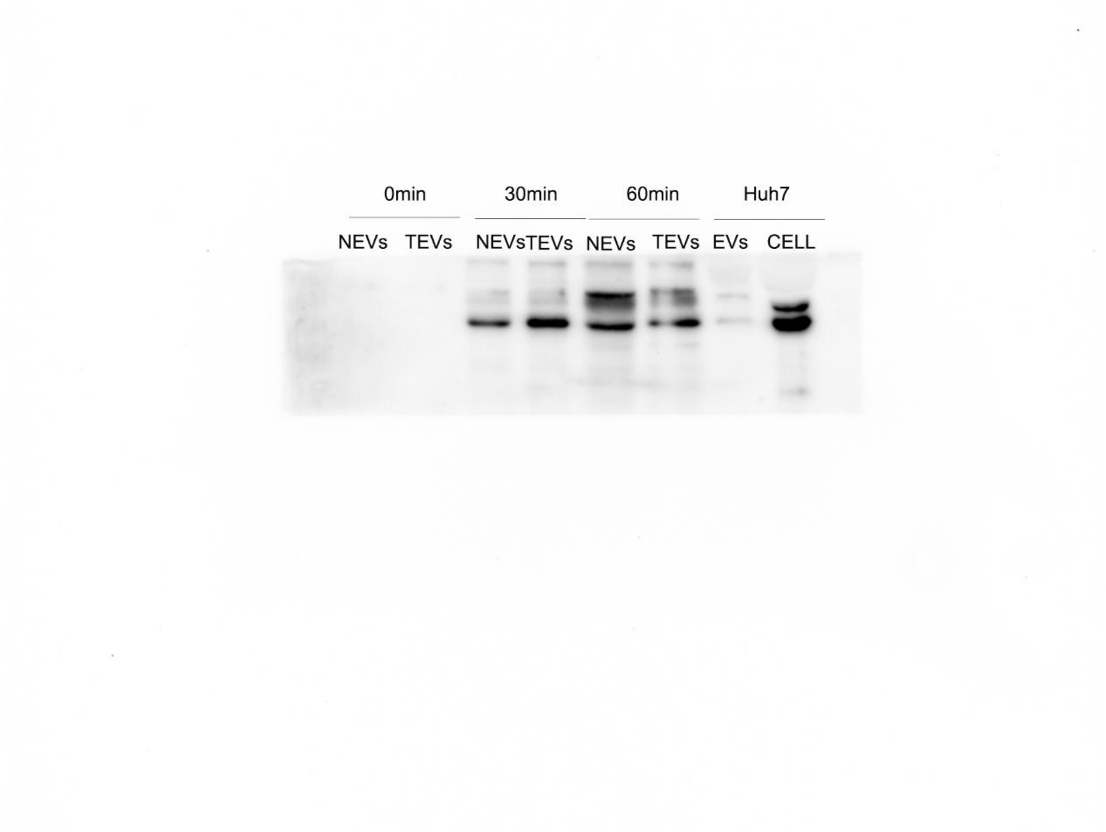


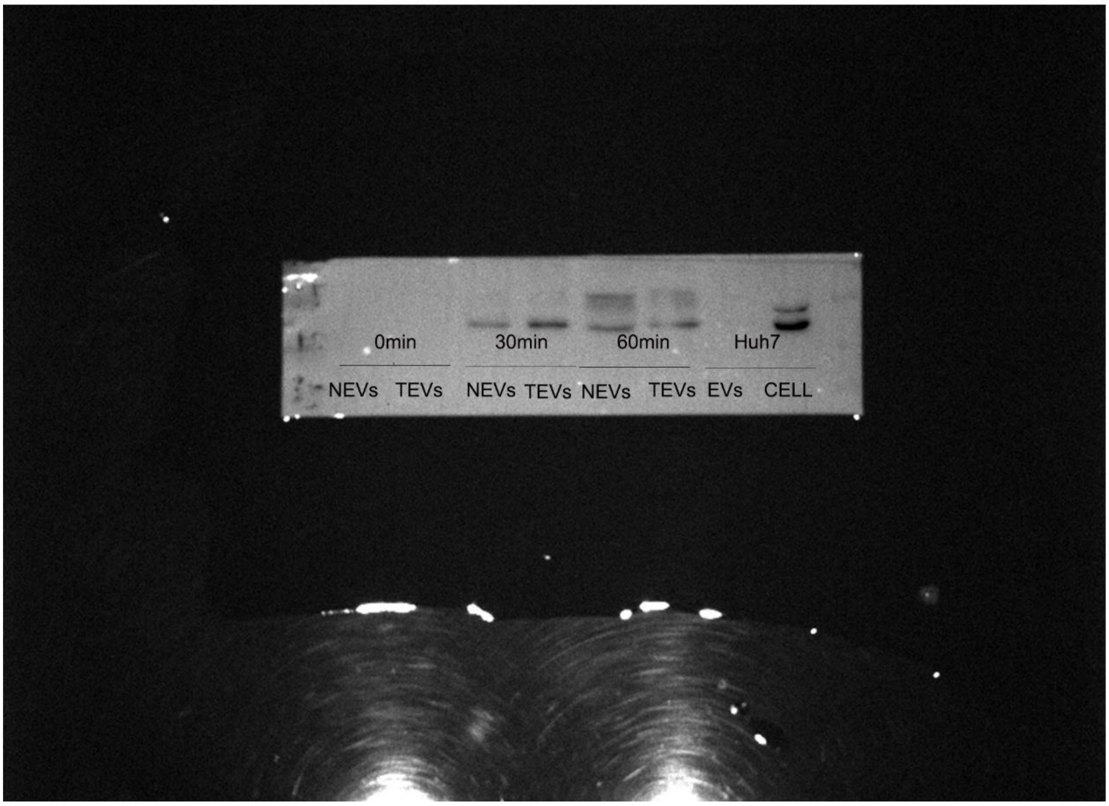

S1 Fig1E CK19 及 marker

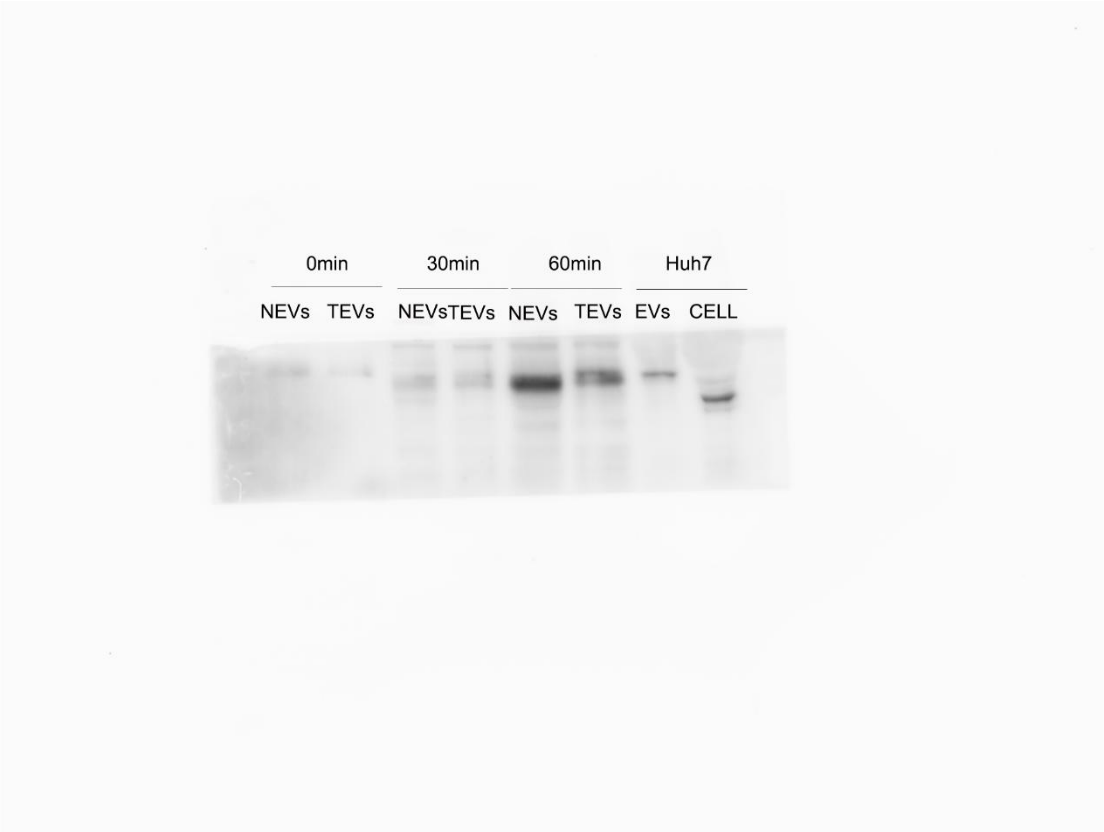


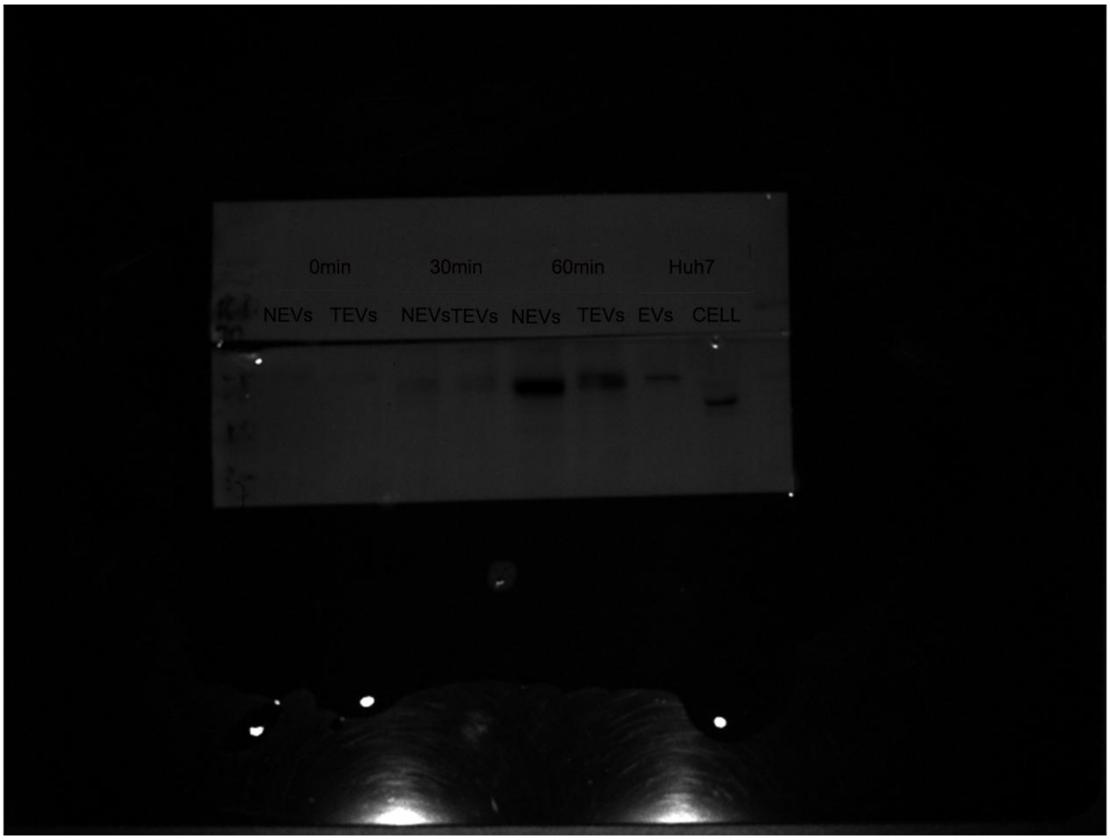

Supplement: S1 Text — (PDF) [file pone.0355303.s006.pdf]
