# Supplementary material for: Optimization of extracellular vesicle extraction from hepatic tissue interstitial fluid and analysis of their ncRNA expression profiles
Source: PLoS One. 2026 Aug 3;21(8):e0355303. doi: 10.1371/journal.pone.0355303 (PMC13432105; doi:10.1371/journal.pone.0355303)
Supplement: Attachment — Submitted filename: Reviewer comment.docx [file pone.0355303.s007.docx]

**Optimization of Extracellular Vesicle Extraction from Hepatic Tissue Interstitial Fluid**

**and Analysis of Their ncRNA Expression Profiles**

**Summary**

This manuscript examines a method for isolating liver tissue–derived TIF-sEVs and explores the non-coding RNAs they contain, with the aim of identifying potential biomarkers for the early diagnosis and precision intervention of hepatocellular carcinoma. However, several points in the manuscript require further clarification..

**Comment:**

**Introduction**

1. Please clarify the potential of TIF-sEVs as biomarkers for early diagnosis in cancer and other diseases, and support this statement with additional recent references.

**Materials and methods**

1. Please provide more detailed methodological information on the ROC analysis.

**Results**

1. Please clarify why six lncRNAs (e.g., AL031985, TMCC1-AS1) and eleven miRNAs (e.g., miR-1224-5p, miR-483-5p) were examined. Were these non-coding RNAs selected from the array results or from the literature?
2. Please improve the figure quality, particularly the western blot bands and font, which appear blurry.
3. The ROC analysis should also present separate curves for the lncRNA GAS5 and the miRNA hsa-miR-194-5p. Please also report the corresponding sensitivity and specificity (at the selected cutoff).
4. ROC analyses for the other non-coding RNAs should be reported either in the Results section or in the Supplementary Materials.

**Discussion**

1. Because the study cohort included only HCC and non-HCC groups, without stratification by HCC stage, the current analysis may not adequately support the stated objective of “providing potential biomarkers for early diagnosis.” The authors should therefore discuss this limitation and clarify how their findings relate to early-stage HCC detection.
