# Supplementary material for: Optimization of extracellular vesicle extraction from hepatic tissue interstitial fluid and analysis of their ncRNA expression profiles
Source: PLoS One. 2026 Aug 3;21(8):e0355303. doi: 10.1371/journal.pone.0355303 (PMC13432105; doi:10.1371/journal.pone.0355303)
Supplement: Attachment — Submitted filename: Response to Reviewers.docx [file pone.0355303.s010.docx]

Response to the Reviewer's Comments

Dear Editors and Reviewers,

We appreciate the opportunity to revise our manuscript and are grateful for the insightful comments provided by the reviewers. Those comments are all valuable and very helpful for revising and improving our paper, as well as the important guiding significance to our researches. In the following, we have provided detailed responses to each of the reviewers' comments. Revised portion are marked in red in the paper. Additionally, we have conducted a comprehensive revision of the entire manuscript. In this response letter, the reviewers' comments are laid out below in *italicized font*, and our responses are in red text. We have tried our best to make all the revisions clear, and we hope that the revised manuscript meets the requirements for publication.

Reviewer#1

*Comment1: The authors isolated hepatic tissue interstitial fluid using an enzymatic digestion method. While the Discussion compares this protocol with previously reported methods, enzymatic digestion may induce cellular disruption and artificial release of extracellular vesicles during tissue processing. Therefore, it remains unclear whether the isolated vesicles truly represent EVs present in the native interstitial space or vesicles released as a consequence of tissue digestion. The authors should clarify how they minimized potential contamination from intracellular components and discuss this methodological limitation more explicitly.*

Response 1: Thank you for raising the critical concern that enzymatic digestion may cause cell damage, leading to the artificial release of vesicles and intracellular components. We have carefully examined this risk and would like to address it from the following perspectives.

First, tissue interstitial fluid itself constitutes a dynamic microenvironment in which cells continuously secrete extracellular vesicles (EVs) under physiological conditions. The so-called "native" EVs are inherently a mixture of pre-existing vesicles and those freshly released. Any isolation method would struggle to strictly distinguish between the two at the single-particle level. In this study, both para-tumor and tumor tissues were processed under identical conditions; therefore, even if digestion-induced components were present, they would be evenly distributed between the two groups and would not introduce confounding bias for comparative analysis.

Second, we incorporated multiple levels of purification into the experimental workflow. After enzymatic digestion, the tissue homogenate was sequentially subjected to differential centrifugation (300 × g, 2,000 × g) to remove cells and large debris, followed by ultracentrifugation (120,000 × g) and 0.22 μm filtration to obtain the vesicle pellet. Protease and phosphatase inhibitors were added immediately upon termination of digestion.

Third, to evaluate whether enzymatic digestion induces the artificial release of vesicles, we established a 0-minute control group (mechanical mincing only, without enzyme treatment) and compared it in parallel with the 30-minute and 60-minute enzymatic digestion groups. The Western blot results presented in S1 Fig E indicate that the 0-minute control was essentially negative for all examined EV markers, confirming that mechanical fragmentation alone does not release detectable EVs from solid liver tissue and does not introduce significant contamination from intracellular components. The comparison between the 30-minute and 60-minute groups revealed tissue-specific differentiation patterns, which are described in detail in the Results section (lines 142–157 and S1 Fig E) .

In summary, we have explicitly addressed the limitations of the enzymatic digestion method in the Discussion section of the revised manuscript: the potential for trace release of vesicles induced by procedural stress cannot be completely excluded, and it remains challenging to distinguish between "in situ pre-existing" and "procedure-generated" EVs at the single-particle level. This method is suitable for the rapid enrichment of vesicle-associated nucleic acids from small tissue samples (especially those exhibiting fibrosis) for differential screening. The discussion of limitations has been integrated into the revised manuscript (lines 390–394).

*Comment 2: The current study mainly provides a descriptive analysis of ncRNA expression profiles in extracellular vesicles derived from hepatic tissue interstitial fluid. Although the authors discuss the potential diagnostic value of several ncRNAs based on previous literature, the biological significance of the findings in this study remains largely unexplored. Without functional validation or mechanistic investigation, it is difficult to determine the role of these ncRNAs in hepatocellular carcinoma biology. Additional biological interpretation or functional validation would strengthen the conclusions of the study.*

Response 2: Thank you for your suggestion. We fully agree on the importance of functional validation. However, it is important to note that the primary focus of this paper is on the establishment of methodology and preliminary discoveries. First, we developed an optimized protocol for the preparation of TIF-sEVs from liver tissue. Second, we conducted an initial screening that identified 11 differentially expressed lncRNAs and 22 differentially expressed miRNAs. Mechanistic investigations and target gene validations have been incorporated into our follow-up research plan. The current findings provide a methodological foundation and a basis of candidate molecules for subsequent functional studies.

*Comment 3: The manuscript first identifies differentially expressed lncRNAs and subsequently analyzes miRNAs. However, the rationale for presenting the relative expression relationship between these ncRNAs is unclear. It may be more informative to first analyze the biological pathways or functional processes associated with the differentially expressed lncRNAs and miRNAs separately. Subsequently, investigating whether these ncRNAs are linked to shared pathways or regulatory mechanisms could provide a more biologically meaningful basis for comparing their relative expression patterns.*

Response 3: Thank you for your suggestion. We recognize the importance of analysis from the perspective of shared pathways or co-regulatory networks. However, the primary focus of this paper is on methodological validation and differential expression screening. The main objective of this study is to verify whether non-coding RNAs (ncRNAs) can be stably detected in tumor-derived small extracellular vesicles (TIF-sEVs), which led to the adoption of a parallel screening strategy. Additionally, using TMCC1-AS1 as an example, predictions from Starbase indicate that it contains potential binding sites for several differentially expressed microRNAs (miRNAs), such as miR-16-5p, suggesting a possible competing endogenous RNA (ceRNA) regulatory mechanism. Furthermore, these sequence-based predictions have yet to be experimentally validated, and an integrative analysis at the pathway level is planned for future investigation.

*Comment 4: There appears to be an inconsistency between the text and the cited figure numbers in lines 118–146. The authors should carefully review and correct the figure references to ensure that they correspond appropriately to the described results.*

Response 4: Thank you for your careful review. We have meticulously checked the result descriptions in lines 118–146 against the cited figure numbers and have made the following corrections: "Fig 2B–C" on line 125 has been corrected to "Fig 1B–C," and "Fig 2D–E" on line 142 has been amended to "Fig 1D–E."

*Comment 5: The manuscript suggests that EVs isolated from TIF may originate from liver tissue. However, it is generally difficult to determine the precise cellular origin of EVs present in interstitial fluid, as vesicles from multiple cellular sources may coexist in this compartment. Therefore, interpretations implying a definitive hepatic origin should be made with caution.*

Response 5: Thank you for your reminder. We agree that extracellular vesicles (EVs) present in tissue interstitial fluid may originate from various cell types, and our isolation method does not permit the assignment of individual EVs to a specific cell of origin. Therefore, we have revised the manuscript to avoid any implication of a definitive or exclusive hepatocellular origin. Specifically, we now state that the isolated vesicles are enriched for hepatocyte-associated markers (ASGPR and ALB) and that hepatocytes represent one of the major sources, rather than the sole source, of the TIF-sEV population. Furthermore, the detection of weaker signals for CK19 and CD34 (Supplementary Fig 1E) confirms the anticipated co-presence of EVs from other liver-resident cell types.

*Comment 6: Although the authors detected the hepatocyte-associated membrane protein ASGPR, additional characterization using other liver-associated EV markers could further strengthen the interpretation regarding the potential hepatic origin of the isolated vesicles.*

Response 6: Thank you for your suggestion. We have conducted further characterization by detecting ALB (positive), along with CK19 and CD34, both of which exhibited weaker signals compared to ASGPR/ALB. These results, illustrated in Figure S1E, provide additional support for the hepatic origin of the isolated vesicles. This revision has been incorporated into the revised manuscript.

*Comment 7: The characterization of extracellular vesicles should also follow established guidelines such as the Minimal Information for Studies of Extracellular Vesicles (MISEV). The authors should clarify whether their EV characterization fully meets these criteria and consider including additional validation markers, particularly negative markers for non-vesicular contaminants.*

Response 7: Thank you for the reminder regarding compliance with the MISEV2023 guidelines. We have systematically evaluated our characterization against the MISEV2023 recommendations item by item (see Fig 1 and Supplementary Fig 2). Transmission electron microscopy confirmed the typical cup-shaped morphology of the vesicles. Nanoparticle tracking analysis (NTA) indicated a peak particle size within the range of 50–200 nm. Additionally, Western blot analysis clearly detected the positive markers CD63, CD9, TSG101, and Alix. Although the negative marker calnexin was present in the isolated vesicles, indicating trace contamination from the endoplasmic reticulum, this level of contamination is challenging to eliminate entirely when isolating extracellular vesicles (EVs) from tissue via enzymatic digestion. Given that the downstream application is quantitative reverse transcription polymerase chain reaction (qRT-PCR), this level of contamination does not impede RNA detection. Furthermore, the treatment conditions were consistent across all groups, ensuring that they do not affect the comparative analysis.

Reviewer#2

Introduction

*Comment 1: Please clarify the potential of TIF-sEVs as biomarkers for early diagnosis in cancer and other diseases, and support this statement with additional recent references.*

Response 1: Thank you for your suggestion. We have incorporated four recent references (2023–2025) that address hepatocellular carcinoma, melanoma, burn depth assessment, and non-small cell lung cancer. These additions further substantiate the potential of TIF-sEVs for early diagnosis. The revisions have been made in the Introduction section (lines 62–70).

Materials and methods

*Comment 2: Please provide more detailed methodological information on the ROC analysis.*

Response 2: Thank you for your valuable suggestion regarding the inclusion of detailed methodological information for the ROC analysis. We have now incorporated a comprehensive description of the analysis workflow in the revised manuscript, specifically in section 4.10, titled 'ROC Analysis.'

Results

*Comment 3: Please clarify why six lncRNAs (e.g., AL031985, TMCC1-AS1) and eleven miRNAs (e.g., miR-1224-5p, miR-483-5p) were examined. Were these non-coding RNAs selected from the array results or from the literature?*

Response 3: Thank you for your question. First, I would like to correct a numerical discrepancy: this study examined 11 long non-coding RNAs (lncRNAs) and 22 microRNAs (miRNAs). The lncRNAs, including AL031985 and TMCC1-AS1, were selected based on differential expression analysis of The Cancer Genome Atlas (TCGA) hepatocellular carcinoma data. The miRNAs, such as miR-1224-5p and miR-483-5p, were derived partially from TCGA and partially from high-throughput sequencing of serum/plasma extracellular vesicles, with the raw data deposited in the Gene Expression Omnibus (GEO) under accession number GSE302990. All differential expression results and survival analysis plots are provided in Supplementary File 3, titled "ncRNA differential expression survival analysis."

*Comment 4: Please improve the figure quality, particularly the western blot bands and font, which appear blurry.*

Response 4: Thank you for pointing out the image quality issues. We have improved all figures and re-uploaded them, uniformly adopting Arial 12pt font and submitting in TIF format to ensure clarity.

*Comment 5:* The ROC analysis should also present separate curves for the lncRNA GAS5 and the miRNA hsa-miR-194-5p. Please also report the corresponding sensitivity and specificity (at the selected cutoff).

Response 5: Thank you for your valuable suggestion. In response to the reviewer's comment, we have redrawn Figure 4, which illustrates the ROC curves of the combined diagnostic model. This updated figure now includes the individual ROC curves for each variable within the combined model, specifically lncRNA GAS5 and miRNA hsa-miR-194-5p. Furthermore, we have provided additional details regarding the diagnostic performance of the combined model at a cutoff value of 0.628 in Section 2.4 (ROC Curve Analysis), noting a sensitivity of 0.786 and a specificity of 1.000. These revisions have been incorporated into the revised manuscript.

*Comment 6: ROC analyses for the other non-coding RNAs should be reported either in the Results section or in the Supplementary Materials.*

Response 6: Thank you for your suggestion. We have compiled the results of the ROC analysis, including the Area Under the Curve (AUC), Lower Confidence Interval (LCI), and Upper Confidence Interval (UCI), along with corresponding figures for all remaining non-coding RNAs (ncRNAs) in the Supplementary Materials for the readers' reference.

Discussion

*Comment 6: Because the study cohort included only HCC and non-HCC groups, without stratification by HCC stage, the current analysis may not adequately support the stated objective of “providing potential biomarkers for early diagnosis.” The authors should therefore discuss this limitation and clarify how their findings relate to early-stage HCC detection.*

Response 7: Thank you for your insightful comment. We agree that our cohort was not stratified according to the stages of hepatocellular carcinoma (HCC), and the current data do not substantiate a claim of 'early diagnosis.' Consequently, we have uniformly revised the conclusion throughout the manuscript, changing 'early diagnosis' to 'differential diagnosis.' This revision has been applied in the Abstract (lines 43), Introduction (lines 89), and Discussion (lines 265和414). Additionally, we have explicitly addressed the limitations in the revised Discussion section.

We have tried our best to improve the manuscript. We appreciate the editors and reviewers for their earnest work, and hope that the revisions will meet with your approval.

Sincerely,

Yingtang Gao
