# Supplementary material for: Optimization of extracellular vesicle extraction from hepatic tissue interstitial fluid and analysis of their ncRNA expression profiles
Source: PLoS One. 2026 Aug 3;21(8):e0355303. doi: 10.1371/journal.pone.0355303 (PMC13432105; doi:10.1371/journal.pone.0355303)
Supplement: Attachment — Submitted filename: summary R1.docx [file pone.0355303.s009.docx]

**Optimization of Extracellular Vesicle Extraction from Hepatic Tissue Interstitial Fluid**

**and Analysis of Their ncRNA Expression Profiles**

**Summary**

The authors have addressed the reviewer’s comment and made the appropriate revisions in the manuscript. However, several aspects of the manuscript require further clarification and improvement before it can be considered for publication.

**Comment:**

1. The source of the lncRNA and miRNA data derived from The Cancer Genome Atlas (TCGA) hepatocellular carcinoma dataset and the Gene Expression Omnibus (GEO) dataset under accession number GSE302990 should be clearly described in the Materials and Methods section.

2. The images in Fig. 1C–E are not sufficiently clear, and the graph labels and protein names in the western blot panels appear blurred. The figure should be improved to ensure that all labels are clearly legible and of publication quality.

3. In the ROC curve analysis, please clarify why hsa-miR-194-5p was included, given that it was not identified among the eleven miRNAs and does not appear to have been reported as significantly different in the prior study. The rationale for selecting this miRNA for ROC analysis should be clearly explained.

4. If hsa-miR-194-5p and GAS5 are proposed as potential biomarkers for hepatocellular carcinoma, the authors should expand the Discussion to include their previously reported biological roles and relevance to hepatocellular carcinoma. This additional context would strengthen the rationale for their biomarker potential and better support the interpretation of the findings.

5. Sections 4.9 and 4.10 appear to have duplicate headings, both titled “qRT-PCR Detection of miRNAs.” Please revise the section titles to avoid duplication and to clearly distinguish the methods described in lines 502 and 519.
