# Supplementary material for: Optimization of extracellular vesicle extraction from hepatic tissue interstitial fluid and analysis of their ncRNA expression profiles
Source: PLoS One. 2026 Aug 3;21(8):e0355303. doi: 10.1371/journal.pone.0355303 (PMC13432105; doi:10.1371/journal.pone.0355303)
Supplement: Attachment — Submitted filename: Response_to_Reviewers_auresp_2.docx [file pone.0355303.s011.docx]

Response to the Reviewer's Comments

Dear Editors and Reviewers,

We appreciate the opportunity to revise our manuscript and are grateful for the insightful comments provided by the reviewers. Those comments are all valuable and very helpful for revising and improving our paper, as well as the important guiding significance to our researches. In the following, we have provided detailed responses to each of the reviewers' comments. Revised portion are marked in red in the paper. Additionally, we have conducted a comprehensive revision of the entire manuscript. In this response letter, the reviewers' comments are laid out below in *italicized font*, and our responses are in red text. We have tried our best to make all the revisions clear, and we hope that the revised manuscript meets the requirements for publication.

*Comment 1: The source of the lncRNA and miRNA data derived from The Cancer Genome Atlas (TCGA) hepatocellular carcinoma dataset and the Gene Expression Omnibus (GEO) dataset under accession number GSE302990 should be clearly described in the Materials and Methods section.*

Response 1: Thank you for your valuable suggestion to describe the lncRNA and miRNA data sources in detail within the Materials and Methods section. We have added the corresponding description of the TCGA hepatocellular carcinoma dataset and the GEO dataset (GSE302990) (lines 416–438).

*Comment 2: The images in Fig. 1C–E are not sufficiently clear, and the graph labels and protein names in the western blot panels appear blurred. The figure should be improved to ensure that all labels are clearly legible and of publication quality.*

Response 2: Thank you for your careful review. The apparent blurring of Fig. 1C–E is likely due to PDF compression during manuscript processing. The original figure was submitted as a high-resolution TIFF file, which can be viewed by clicking "Click here to access/download; Figure; Fig 1.tif" at the top of the figure page. We have verified that all labels and protein names are clearly legible in the original file.

*Comment 3: In the ROC curve analysis, please clarify why hsa-miR-194-5p was included, given that it was not identified among the eleven miRNAs and does not appear to have been reported as significantly different in the prior study. The rationale for selecting this miRNA for ROC analysis should be clearly explained.*

Response 3: Thank you for raising this important question. We apologize for the lack of clarity in our original description. hsa-miR-194-5p was identified from our prior serum sEV sequencing data (GEO: GSE302990), which profiled the HBV-related liver disease spectrum including chronic hepatitis B, cirrhosis, hepatic adenoma, and HCC. It was included as one of 22 candidate miRNAs submitted to LASSO regression, together with 11 candidate lncRNAs and clinical parameters. The LASSO algorithm applies L1 regularization that shrinks coefficients of less predictive variables toward zero; hsa-miR-194-5p was retained with a non-zero coefficient, indicating its contribution to the joint predictive model beyond univariate significance. We have revised the manuscript to clarify this: the Abstract now states in the Methods that LASSO was applied to "all 33 candidate ncRNAs and clinical parameters" and in the Results that the five-variable signature was "selected by LASSO from the full candidate panel" (lines 33–42). The Results section notes that "LASSO regression was performed on 33 candidate ncRNAs and clinical parameters" (lines 241–242), and the Materials and Methods section details the dual-source origin of the 33 candidates (lines 416–438).

*Comment 4: If hsa-miR-194-5p and GAS5 are proposed as potential biomarkers for hepatocellular carcinoma, the authors should expand the Discussion to include their previously reported biological roles and relevance to hepatocellular carcinoma. This additional context would strengthen the rationale for their biomarker potential and better support the interpretation of the findings.*

Response 4: We thank the reviewer for this constructive suggestion. We have expanded the Discussion [lines 384–397] to include the reported biological roles of GAS5 and hsa-miR-194-5p in HCC. GAS5 is a tumor-suppressive lncRNA downregulated in HCC and associated with poor prognosis; hsa-miR-194-5p has been implicated in HBV-related hepatocarcinogenesis and tumor immune regulation. These additions provide biological context for their biomarker potential.

*Comment 5:* Sections 4.9 and 4.10 appear to have duplicate headings, both titled “qRT-PCR Detection of miRNAs.” Please revise the section titles to avoid duplication and to clearly distinguish the methods described in lines 502 and 519.

Response 5: Thank you for noting this duplication. Due to the insertion of Section 4.2 (Data Sources), the original Section 4.9 is now renumbered as 4.10 "qRT-PCR Detection of miRNAs" (lines 542–558), and the original Section 4.10 is now 4.11 "LASSO Regression and Diagnostic Model Development" (lines 559–568). The two sections are now clearly distinguished.

We have tried our best to improve the manuscript. We appreciate the editors and reviewers for their earnest work, and hope that the revisions will meet with your approval.

Sincerely,

Yingtang Gao
