# Supplementary material for: Optimization of extracellular vesicle extraction from hepatic tissue interstitial fluid and analysis of their ncRNA expression profiles
Source: PLoS One. 2026 Aug 3;21(8):e0355303. doi: 10.1371/journal.pone.0355303 (PMC13432105; doi:10.1371/journal.pone.0355303)
Supplement: Peer review history

# Peer review: Decision Letter 0

**DOI:** 10.1371/journal.pone.0355303.r001

**Authors:** Boyan Grigorov

3 Apr 2026

PONE-D-26-05376

Optimization of Extracellular Vesicle Extraction from Hepatic Tissue Interstitial Fluid and Analysis of Their ncRNA Expression Profiles

PLOS One

Dear Dr. Gao,

Thank you for submitting your manuscript to PLOS ONE. After careful consideration, we feel that it has merit but does not fully meet PLOS ONE’s publication criteria as it currently stands. Therefore, we invite you to submit a revised version of the manuscript that addresses the points raised during the review process.

Please submit your revised manuscript by May 18 2026 11:59PM. If you will need more time than this to complete your revisions, please reply to this message or contact the journal office at plosone@plos.org. When you're ready to submit your revision, log on to https://www.editorialmanager.com/pone/ and select the 'Submissions Needing Revision' folder to locate your manuscript file.

Please include the following items when submitting your revised manuscript:

- A letter that responds to each point raised by the academic editor and reviewer(s). You should upload this letter as a separate file labeled 'Response to Reviewers'.
- A marked-up copy of your manuscript that highlights changes made to the original version. You should upload this as a separate file labeled 'Revised Manuscript with Track Changes'.
- An unmarked version of your revised paper without tracked changes. You should upload this as a separate file labeled 'Manuscript'.

If you would like to make changes to your financial disclosure, please include your updated statement in your cover letter. Guidelines for resubmitting your figure files are available below the reviewer comments at the end of this letter.

If applicable, we recommend that you deposit your laboratory protocols in protocols.io to enhance the reproducibility of your results. Protocols.io assigns your protocol its own identifier (DOI) so that it can be cited independently in the future. For instructions see: https://journals.plos.org/plosone/s/submission-guidelines#loc-laboratory-protocols. Additionally, PLOS ONE offers an option for publishing peer-reviewed Lab Protocol articles, which describe protocols hosted on protocols.io. Read more information on sharing protocols at https://plos.org/protocols?utm_medium=editorial-email&utm_source=authorletters&utm_campaign=protocols.

As the corresponding author, your ORCID iD is verified in the submission system and will appear in the published article. PLOS supports the use of ORCID, and we encourage all coauthors to register for an ORCID iD and use it as well. Please encourage your coauthors to verify their ORCID iD within the submission system before final acceptance, as unverified ORCID iDs will not appear in the published article. *Only*  the individual author can complete the verification step; PLOS staff *cannot*  verify ORCID iDs on behalf of authors.

We look forward to receiving your revised manuscript.

Kind regards,

Boyan Grigorov

Academic Editor

PLOS One

Journal Requirements:

When submitting your revision, we need you to address these additional requirements.

1. Please ensure that your manuscript meets PLOS ONE's style requirements, including those for file naming. The PLOS ONE style templates can be found at

https://journals.plos.org/plosone/s/file?id=wjVg/PLOSOne_formatting_sample_main_body.pdf and

https://journals.plos.org/plosone/s/file?id=ba62/PLOSOne_formatting_sample_title_authors_affiliations.pdf

2. We note that the grant information you provided in the ‘Funding Information’ and ‘Financial Disclosure’ sections do not match.

When you resubmit, please ensure that you provide the correct grant numbers for the awards you received for your study in the ‘Funding Information’ section.

3. Thank you for stating the following financial disclosure:

“The APC was funded by Natural Science Foundation of Tianjin Science and Technology Bureau (No. 21JCZDJC01050), the Key Research Project of Tianjin Health Commission (No.TJWJ2021ZD003)”

Please state what role the funders took in the study.  If the funders had no role, please state: "The funders had no role in study design, data collection and analysis, decision to publish, or preparation of the manuscript."

If this statement is not correct you must amend it as needed.

Please include this amended Role of Funder statement in your cover letter; we will change the online submission form on your behalf.

4. PLOS ONE now requires that authors provide the original uncropped and unadjusted images underlying all blot or gel results reported in a submission’s figures or Supporting Information files. This policy and the journal’s other requirements for blot/gel reporting and figure preparation are described in detail at https://journals.plos.org/plosone/s/figures#loc-blot-and-gel-reporting-requirements and https://journals.plos.org/plosone/s/figures#loc-preparing-figures-from-image-files. When you submit your revised manuscript, please ensure that your figures adhere fully to these guidelines and provide the original underlying images for all blot or gel data reported in your submission. See the following link for instructions on providing the original image data: https://journals.plos.org/plosone/s/figures#loc-original-images-for-blots-and-gels.

In your cover letter, please note whether your blot/gel image data are in Supporting Information or posted at a public data repository, provide the repository URL if relevant, and provide specific details as to which raw blot/gel images, if any, are not available. Email us at plosone@plos.org if you have any questions.

5. We notice that your supplementary tables are included in the manuscript file. Please remove them and upload them with the file type 'Supporting Information'. Please ensure that each Supporting Information file has a legend listed in the manuscript after the references list.

6. If the reviewer comments include a recommendation to cite specific previously published works, please review and evaluate these publications to determine whether they are relevant and should be cited. There is no requirement to cite these works unless the editor has indicated otherwise.

[Note: HTML markup is below. Please do not edit.]

Reviewers' comments:

Reviewer's Responses to Questions

**Comments to the Author**

1. Is the manuscript technically sound, and do the data support the conclusions?

The manuscript must describe a technically sound piece of scientific research with data that supports the conclusions. Experiments must have been conducted rigorously, with appropriate controls, replication, and sample sizes. The conclusions must be drawn appropriately based on the data presented.

Reviewer #1: Yes

Reviewer #2: Partly

**********

2. Has the statistical analysis been performed appropriately and rigorously?

Reviewer #1: Yes

Reviewer #2: Yes

**********

3. Have the authors made all data underlying the findings in their manuscript fully available?

The PLOS Data policy requires authors to make all data underlying the findings described in their manuscript fully available without restriction, with rare exception (please refer to the Data Availability Statement in the manuscript PDF file). The data should be provided as part of the manuscript or its supporting information, or deposited to a public repository. For example, in addition to summary statistics, the data points behind means, medians and variance measures should be available. If there are restrictions on publicly sharing data—e.g. participant privacy or use of data from a third party—those must be specified.

Reviewer #1: Yes

Reviewer #2: Yes

**********

4. Is the manuscript presented in an intelligible fashion and written in standard English?

PLOS ONE does not copyedit accepted manuscripts, so the language in submitted articles must be clear, correct, and unambiguous. Any typographical or grammatical errors should be corrected at revision, so please note any specific errors here.

Reviewer #1: Yes

Reviewer #2: Yes

**********

5. Review Comments to the Author

Please use the space provided to explain your answers to the questions above. You may also include additional comments for the author, including concerns about dual publication, research ethics, or publication ethics. (Please upload your review as an attachment if it exceeds 20,000 characters)

Reviewer #1: Shubo Liu and colleagues investigated extracellular vesicles (EVs) isolated from hepatic tissue interstitial fluid (TIF) and analyzed the expression patterns of several lncRNAs and miRNAs in hepatocellular carcinoma. The attempt to explore ncRNA signatures in EVs derived from TIF is interesting, and the study may provide useful information for identifying potential biomarkers related to HCC. However, several issues should be addressed to strengthen the manuscript.

1. The authors isolated hepatic tissue interstitial fluid using an enzymatic digestion method. While the Discussion compares this protocol with previously reported methods, enzymatic digestion may induce cellular disruption and artificial release of extracellular vesicles during tissue processing. Therefore, it remains unclear whether the isolated vesicles truly represent EVs present in the native interstitial space or vesicles released as a consequence of tissue digestion. The authors should clarify how they minimized potential contamination from intracellular components and discuss this methodological limitation more explicitly.

2. The current study mainly provides a descriptive analysis of ncRNA expression profiles in extracellular vesicles derived from hepatic tissue interstitial fluid. Although the authors discuss the potential diagnostic value of several ncRNAs based on previous literature, the biological significance of the findings in this study remains largely unexplored. Without functional validation or mechanistic investigation, it is difficult to determine the role of these ncRNAs in hepatocellular carcinoma biology. Additional biological interpretation or functional validation would strengthen the conclusions of the study.

3. The manuscript first identifies differentially expressed lncRNAs and subsequently analyzes miRNAs. However, the rationale for presenting the relative expression relationship between these ncRNAs is unclear. It may be more informative to first analyze the biological pathways or functional processes associated with the differentially expressed lncRNAs and miRNAs separately. Subsequently, investigating whether these ncRNAs are linked to shared pathways or regulatory mechanisms could provide a more biologically meaningful basis for comparing their relative expression patterns.

4. There appears to be an inconsistency between the text and the cited figure numbers in lines 118–146. The authors should carefully review and correct the figure references to ensure that they correspond appropriately to the described results.

5. The manuscript suggests that EVs isolated from TIF may originate from liver tissue. However, it is generally difficult to determine the precise cellular origin of EVs present in interstitial fluid, as vesicles from multiple cellular sources may coexist in this compartment. Therefore, interpretations implying a definitive hepatic origin should be made with caution.

6. Although the authors detected the hepatocyte-associated membrane protein ASGPR, additional characterization using other liver-associated EV markers could further strengthen the interpretation regarding the potential hepatic origin of the isolated vesicles.

7. The characterization of extracellular vesicles should also follow established guidelines such as the Minimal Information for Studies of Extracellular Vesicles (MISEV). The authors should clarify whether their EV characterization fully meets these criteria and consider including additional validation markers, particularly negative markers for non-vesicular contaminants.

Reviewer #2: Summary

This manuscript examines a method for isolating liver tissue–derived TIF-sEVs and explores the non-coding RNAs they contain, with the aim of identifying potential biomarkers for the early diagnosis and precision intervention of hepatocellular carcinoma. However, several points in the manuscript require further clarification.

Comment:

Introduction

1. Please clarify the potential of TIF-sEVs as biomarkers for early diagnosis in cancer and other diseases, and support this statement with additional recent references.

Materials and methods

1. Please provide more detailed methodological information on the ROC analysis.

Results

1. Please clarify why six lncRNAs (e.g., AL031985, TMCC1-AS1) and eleven miRNAs (e.g., miR-1224-5p, miR-483-5p) were examined. Were these non-coding RNAs selected from the array results or from the literature?

2. Please improve the figure quality, particularly the western blot bands and font, which appear blurry.

3. The ROC analysis should also present separate curves for the lncRNA GAS5 and the miRNA hsa-miR-194-5p. Please also report the corresponding sensitivity and specificity (at the selected cutoff).

4. ROC analyses for the other non-coding RNAs should be reported either in the Results section or in the Supplementary Materials.

Discussion

1. Because the study cohort included only HCC and non-HCC groups, without stratification by HCC stage, the current analysis may not adequately support the stated objective of “providing potential biomarkers for early diagnosis.” The authors should therefore discuss this limitation and clarify how their findings relate to early-stage HCC detection.

**********

6. PLOS authors have the option to publish the peer review history of their article (what does this mean?). If published, this will include your full peer review and any attached files.

If you choose “no”, your identity will remain anonymous but your review may still be made public.

**Do you want your identity to be public for this peer review?**  For information about this choice, including consent withdrawal, please see our Privacy Policy.

Reviewer #1: No

Reviewer #2: No

**********

[NOTE: If reviewer comments were submitted as an attachment file, they will be attached to this email and accessible via the submission site. Please log into your account, locate the manuscript record, and check for the action link "View Attachments". If this link does not appear, there are no attachment files.]

To ensure your figures meet our technical requirements, please review our figure guidelines: https://journals.plos.org/plosone/s/figures

You may also use PLOS’s free figure tool, NAAS, to help you prepare publication quality figures: https://journals.plos.org/plosone/s/figures#loc-tools-for-figure-preparation.

NAAS will assess whether your figures meet our technical requirements by comparing each figure against our figure specifications.

Attachment Submitted filename: Reviewer comment.docx

# Author comment: Author response to Decision Letter 1

**DOI:** 10.1371/journal.pone.0355303.r002

19 May 2026

Dear Editor,

We thank the academic editor and reviewers for their constructive comments. All revisions have been completed.

The following journal requirements have been addressed:

Style requirements: The manuscript has been formatted to meet PLOS ONE's style guidelines, including file naming.

Funding information and Financial Disclosure: We regret that an incorrect version of the Financial Disclosure statement was included in the initial submission. Since we are unable to edit this section directly in the submission system at this stage, please find the correct and complete statement below:

“This work was supported by the Tianjin Municipal Health Commission (Grant No. TJWJ2021ZD003, received by Y. Gao) and the Natural Science Foundation of Tianjin Science and Technology Bureau (Grant No. 21JCZDJC01050, received by Y. Gao). The funders had no role in study design, data collection and analysis, decision to publish, or preparation of the manuscript.”

Original blot/gel images: The original uncropped and unadjusted images underlying all blot/gel data have been uploaded as Supporting Information.

Supplementary tables: All supplementary tables have been removed from the manuscript file and uploaded separately as “Supporting Information” files, with legends listed after the references.

A detailed point-by-point response to all reviewer comments has been uploaded as a separate file titled “Response to Reviewers.”

Thank you for handling our manuscript. We look forward to your final decision.

Sincerely,

Yingtang Gao

Attachment Submitted filename: Response to Reviewers.docx

# Peer review: Decision Letter 1

**DOI:** 10.1371/journal.pone.0355303.r003

**Authors:** Boyan Grigorov

14 Jun 2026

PONE-D-26-05376R1

Optimization of Extracellular Vesicle Extraction from Hepatic Tissue Interstitial Fluid and Analysis of Their ncRNA Expression Profiles

PLOS One

Dear Dr. Gao,

Thank you for submitting your manuscript to PLOS ONE. After careful consideration, we feel that it has merit but does not fully meet PLOS ONE’s publication criteria as it currently stands. Therefore, we invite you to submit a revised version of the manuscript that addresses the points raised during the review process.

- **Reviewers appreciated your revised manuscript, however to improve the quality of the future article, you need to introduce the changed asked by the second reviewer.**

-->Please submit your revised manuscript by Jul 29 2026 11:59PM. If you will need more time than this to complete your revisions, please reply to this message or contact the journal office at plosone@plos.org. When you're ready to submit your revision, log on to https://www.editorialmanager.com/pone/ and select the 'Submissions Needing Revision' folder to locate your manuscript file.

Please include the following items when submitting your revised manuscript:

- A letter that responds to each point raised by the academic editor and reviewer(s). You should upload this letter as a separate file labeled 'Response to Reviewers'.
- A marked-up copy of your manuscript that highlights changes made to the original version. You should upload this as a separate file labeled 'Revised Manuscript with Track Changes'.
- An unmarked version of your revised paper without tracked changes. You should upload this as a separate file labeled 'Manuscript'.

If you would like to make changes to your financial disclosure, please include your updated statement in your cover letter. Guidelines for resubmitting your figure files are available below the reviewer comments at the end of this letter.

If applicable, we recommend that you deposit your laboratory protocols in protocols.io to enhance the reproducibility of your results. Protocols.io assigns your protocol its own identifier (DOI) so that it can be cited independently in the future. For instructions see: https://journals.plos.org/plosone/s/submission-guidelines#loc-laboratory-protocols. Additionally, PLOS ONE offers an option for publishing peer-reviewed Lab Protocol articles, which describe protocols hosted on protocols.io. Read more information on sharing protocols at https://plos.org/protocols?utm_medium=editorial-email&utm_source=authorletters&utm_campaign=protocols.

As the corresponding author, your ORCID iD is verified in the submission system and will appear in the published article. PLOS supports the use of ORCID, and we encourage all coauthors to register for an ORCID iD and use it as well. Please encourage your coauthors to verify their ORCID iD within the submission system before final acceptance, as unverified ORCID iDs will not appear in the published article. *Only*  the individual author can complete the verification step; PLOS staff *cannot*  verify ORCID iDs on behalf of authors.

We look forward to receiving your revised manuscript.

Kind regards,

Boyan Grigorov

Academic Editor

PLOS One

**Journal Requirements:**

1. If the reviewer comments include a recommendation to cite specific previously published works, please review and evaluate these publications to determine whether they are relevant and should be cited. There is no requirement to cite these works unless the editor has indicated otherwise.

2. Please review your reference list to ensure that it is complete and correct. If you have cited papers that have been retracted, please include the rationale for doing so in the manuscript text, or remove these references and replace them with relevant current references. Any changes to the reference list should be mentioned in the rebuttal letter that accompanies your revised manuscript. If you need to cite a retracted article, indicate the article’s retracted status in the References list and also include a citation and full reference for the retraction notice.

[Note: HTML markup is below. Please do not edit.]

Reviewers' comments:

Reviewer's Responses to Questions

**Comments to the Author**

1. If the authors have adequately addressed your comments raised in a previous round of review and you feel that this manuscript is now acceptable for publication, you may indicate that here to bypass the “Comments to the Author” section, enter your conflict of interest statement in the “Confidential to Editor” section, and submit your "Accept" recommendation.

Reviewer #1: All comments have been addressed

Reviewer #2: All comments have been addressed

**********

2. Is the manuscript technically sound, and do the data support the conclusions?

The manuscript must describe a technically sound piece of scientific research with data that supports the conclusions. Experiments must have been conducted rigorously, with appropriate controls, replication, and sample sizes. The conclusions must be drawn appropriately based on the data presented.

Reviewer #1: Yes

Reviewer #2: Partly

**********

3. Has the statistical analysis been performed appropriately and rigorously?

Reviewer #1: Yes

Reviewer #2: Yes

**********

4. Have the authors made all data underlying the findings in their manuscript fully available?

The PLOS Data policy requires authors to make all data underlying the findings described in their manuscript fully available without restriction, with rare exception (please refer to the Data Availability Statement in the manuscript PDF file). The data should be provided as part of the manuscript or its supporting information, or deposited to a public repository. For example, in addition to summary statistics, the data points behind means, medians and variance measures should be available. If there are restrictions on publicly sharing data—e.g. participant privacy or use of data from a third party—those must be specified.

Reviewer #1: Yes

Reviewer #2: Yes

**********

5. Is the manuscript presented in an intelligible fashion and written in standard English?

PLOS ONE does not copyedit accepted manuscripts, so the language in submitted articles must be clear, correct, and unambiguous. Any typographical or grammatical errors should be corrected at revision, so please note any specific errors here.

Reviewer #1: Yes

Reviewer #2: Yes

**********

6. Review Comments to the Author

Please use the space provided to explain your answers to the questions above. You may also include additional comments for the author, including concerns about dual publication, research ethics, or publication ethics. (Please upload your review as an attachment if it exceeds 20,000 characters)

**Reviewer #1:**  I appreciate the effort the authors have made to address the issues raised during the review process and to improve the manuscript. I hope that your future mechanistic and functional studies will build upon these interesting preliminary findings and provide further insights into the biological roles of TIF-derived extracellular vesicle ncRNAs.

**Reviewer #2:**  Summary

The authors have addressed the reviewer’s comment and made the appropriate revisions in the manuscript. However, several aspects of the manuscript require further clarification and improvement before it can be considered for publication.

Comment:

1. The source of the lncRNA and miRNA data derived from The Cancer Genome Atlas (TCGA) hepatocellular carcinoma dataset and the Gene Expression Omnibus (GEO) dataset under accession number GSE302990 should be clearly described in the Materials and Methods section.

2. The images in Fig. 1C–E are not sufficiently clear, and the graph labels and protein names in the western blot panels appear blurred. The figure should be improved to ensure that all labels are clearly legible and of publication quality.

3. In the ROC curve analysis, please clarify why hsa-miR-194-5p was included, given that it was not identified among the eleven miRNAs and does not appear to have been reported as significantly different in the prior study. The rationale for selecting this miRNA for ROC analysis should be clearly explained.

4. If hsa-miR-194-5p and GAS5 are proposed as potential biomarkers for hepatocellular carcinoma, the authors should expand the Discussion to include their previously reported biological roles and relevance to hepatocellular carcinoma. This additional context would strengthen the rationale for their biomarker potential and better support the interpretation of the findings.

5. Sections 4.9 and 4.10 appear to have duplicate headings, both titled “qRT-PCR Detection of miRNAs.” Please revise the section titles to avoid duplication and to clearly distinguish the methods described in lines 502 and 519.

**********

7. PLOS authors have the option to publish the peer review history of their article (what does this mean?). If published, this will include your full peer review and any attached files.

If you choose “no”, your identity will remain anonymous but your review may still be made public.

**Do you want your identity to be public for this peer review?**  For information about this choice, including consent withdrawal, please see our Privacy Policy.

Reviewer #1: No

Reviewer #2: No

**********

[NOTE: If reviewer comments were submitted as an attachment file, they will be attached to this email and accessible via the submission site. Please log into your account, locate the manuscript record, and check for the action link "View Attachments". If this link does not appear, there are no attachment files.]

To ensure your figures meet our technical requirements, please review our figure guidelines: https://journals.plos.org/plosone/s/figures

You may also use PLOS’s free figure tool, NAAS, to help you prepare publication quality figures: https://journals.plos.org/plosone/s/figures#loc-tools-for-figure-preparation.

NAAS will assess whether your figures meet our technical requirements by comparing each figure against our figure specifications.

Attachment Submitted filename: summary R1.docx

# Author comment: Author response to Decision Letter 2

**DOI:** 10.1371/journal.pone.0355303.r004

7 Jul 2026

Dear Editors and Reviewers,

We appreciate the opportunity to revise our manuscript and are grateful for the insightful comments provided by the reviewers. Those comments are all valuable and very helpful for revising and improving our paper, as well as the important guiding significance to our researches. In the following, we have provided detailed responses to each of the reviewers' comments. Revised portion are marked in red in the paper. Additionally, we have conducted a comprehensive revision of the entire manuscript. In this response letter, the reviewers' comments are laid out below in italicized font, and our responses are in red text. We have tried our best to make all the revisions clear, and we hope that the revised manuscript meets the requirements for publication.

Comment 1: The source of the lncRNA and miRNA data derived from The Cancer Genome Atlas (TCGA) hepatocellular carcinoma dataset and the Gene Expression Omnibus (GEO) dataset under accession number GSE302990 should be clearly described in the Materials and Methods section.

Response 1: Thank you for your valuable suggestion to describe the lncRNA and miRNA data sources in detail within the Materials and Methods section. We have added the corresponding description of the TCGA hepatocellular carcinoma dataset and the GEO dataset (GSE302990) (lines 416–438).

Comment 2: The images in Fig. 1C–E are not sufficiently clear, and the graph labels and protein names in the western blot panels appear blurred. The figure should be improved to ensure that all labels are clearly legible and of publication quality.

Response 2: Thank you for your careful review. The apparent blurring of Fig. 1C–E is likely due to PDF compression during manuscript processing. The original figure was submitted as a high-resolution TIFF file, which can be viewed by clicking "Click here to access/download; Figure; Fig 1.tif" at the top of the figure page. We have verified that all labels and protein names are clearly legible in the original file.

Comment 3: In the ROC curve analysis, please clarify why hsa-miR-194-5p was included, given that it was not identified among the eleven miRNAs and does not appear to have been reported as significantly different in the prior study. The rationale for selecting this miRNA for ROC analysis should be clearly explained.

Response 3: Thank you for raising this important question. We apologize for the lack of clarity in our original description. hsa-miR-194-5p was identified from our prior serum sEV sequencing data (GEO: GSE302990), which profiled the HBV-related liver disease spectrum including chronic hepatitis B, cirrhosis, hepatic adenoma, and HCC. It was included as one of 22 candidate miRNAs submitted to LASSO regression, together with 11 candidate lncRNAs and clinical parameters. The LASSO algorithm applies L1 regularization that shrinks coefficients of less predictive variables toward zero; hsa-miR-194-5p was retained with a non-zero coefficient, indicating its contribution to the joint predictive model beyond univariate significance. We have revised the manuscript to clarify this: the Abstract now states in the Methods that LASSO was applied to "all 33 candidate ncRNAs and clinical parameters" and in the Results that the five-variable signature was "selected by LASSO from the full candidate panel" (lines 33–42). The Results section notes that "LASSO regression was performed on 33 candidate ncRNAs and clinical parameters" (lines 241–242), and the Materials and Methods section details the dual-source origin of the 33 candidates (lines 416–438).

Comment 4: If hsa-miR-194-5p and GAS5 are proposed as potential biomarkers for hepatocellular carcinoma, the authors should expand the Discussion to include their previously reported biological roles and relevance to hepatocellular carcinoma. This additional context would strengthen the rationale for their biomarker potential and better support the interpretation of the findings.

Response 4: We thank the reviewer for this constructive suggestion. We have expanded the Discussion [lines 384–397] to include the reported biological roles of GAS5 and hsa-miR-194-5p in HCC. GAS5 is a tumor-suppressive lncRNA downregulated in HCC and associated with poor prognosis; hsa-miR-194-5p has been implicated in HBV-related hepatocarcinogenesis and tumor immune regulation. These additions provide biological context for their biomarker potential.

Comment 5: Sections 4.9 and 4.10 appear to have duplicate headings, both titled “qRT-PCR Detection of miRNAs.” Please revise the section titles to avoid duplication and to clearly distinguish the methods described in lines 502 and 519.

Response 5: Thank you for noting this duplication. Due to the insertion of Section 4.2 (Data Sources), the original Section 4.9 is now renumbered as 4.10 "qRT-PCR Detection of miRNAs" (lines 542–558), and the original Section 4.10 is now 4.11 "LASSO Regression and Diagnostic Model Development" (lines 559–568). The two sections are now clearly distinguished.

We have tried our best to improve the manuscript. We appreciate the editors and reviewers for their earnest work, and hope that the revisions will meet with your approval.

Sincerely,

Yingtang Gao

Attachment Submitted filename: Response_to_Reviewers_auresp_2.docx

# Editor report: Decision Letter 2

**DOI:** 10.1371/journal.pone.0355303.r005

**Authors:** Boyan Grigorov

20 Jul 2026

Optimization of Extracellular Vesicle Extraction from Hepatic Tissue Interstitial Fluid and Analysis of Their ncRNA Expression Profiles

PONE-D-26-05376R2

Dear Dr. Gao,

We’re pleased to inform you that your manuscript has been judged scientifically suitable for publication and will be formally accepted for publication once it meets all outstanding technical requirements.

Within one week, you’ll receive an e-mail detailing the required amendments. When these have been addressed, you’ll receive a formal acceptance letter and your manuscript will be scheduled for publication.

An invoice will be generated when your article is formally accepted. Please note, if your institution has a publishing partnership with PLOS and your article meets the relevant criteria, all or part of your publication costs will be covered. Please make sure your user information is up-to-date by logging into Editorial Manager at Editorial Manager® and clicking the ‘Update My Information' link at the top of the page. For questions related to billing, please contact billing support.

If your institution or institutions have a press office, please notify them about your upcoming paper to help maximize its impact. If they’ll be preparing press materials, please inform our press team as soon as possible -- no later than 48 hours after receiving the formal acceptance. Your manuscript will remain under strict press embargo until 2 pm Eastern Time on the date of publication. For more information, please contact onepress@plos.org.

Kind regards,

Boyan Grigorov

Academic Editor

PLOS One

Additional Editor Comments (optional):

Reviewers' comments:

# Editor report: Acceptance letter

**DOI:** 10.1371/journal.pone.0355303.r006

**Authors:** Boyan Grigorov

PONE-D-26-05376R2

PLOS One

Dear Dr. Gao,

I'm pleased to inform you that your manuscript has been deemed suitable for publication in PLOS One. Congratulations! Your manuscript is now being handed over to our production team.

At this stage, our production department will prepare your paper for publication. This includes ensuring the following:

* All references, tables, and figures are properly cited

* All relevant supporting information is included in the manuscript submission,

* There are no issues that prevent the paper from being properly typeset

You will receive further instructions from the production team, including instructions on how to review your proof when it is ready. Please keep in mind that we are working through a large volume of accepted articles, so please give us a few days to review your paper and let you know the next and final steps.

Lastly, if your institution or institutions have a press office, please let them know about your upcoming paper now to help maximize its impact. If they'll be preparing press materials, please inform our press team within the next 48 hours. Your manuscript will remain under strict press embargo until 2 pm Eastern Time on the date of publication. For more information, please contact onepress@plos.org.

You will receive an invoice from PLOS for your publication fee after your manuscript has reached the completed accept phase. If you receive an email requesting payment before acceptance or for any other service, this may be a phishing scheme. Learn how to identify phishing emails and protect your accounts at https://explore.plos.org/phishing.

If we can help with anything else, please email us at customercare@plos.org.

Thank you for submitting your work to PLOS ONE and supporting open access.

Kind regards,

PLOS ONE Editorial Office Staff

on behalf of

Dr. Boyan Grigorov

Academic Editor

PLOS One
